# Macrocyclic shape-persistency of cyclo[6]aramide results in enhanced multipoint recognition for the highly efficient template-directed synthesis of rotaxanes†
†Electronic supplementary information (ESI) available: Further details of the synthesis, characterization, ^1^H and 2D NMR experiments and computational modelling. CCDC 1475246 and 1475247. For ESI and crystallographic data in CIF or other electronic format see DOI: 10.1039/c6sc04714a



**DOI:** 10.1039/c6sc04714a

**Published:** 2016-11-22

**Authors:** Xiaowei Li, Xiangyang Yuan, Pengchi Deng, Lixi Chen, Yi Ren, Chengyuan Wang, Lixin Wu, Wen Feng, Bing Gong, Lihua Yuan

**Affiliations:** a College of Chemistry , Key Laboratory for Radiation Physics and Technology of Ministry of Education , Analytical & Testing Center , Sichuan University , Chengdu 610064 , Sichuan , China . Email: lhyuan@scu.edu.cn ; Fax: +86-28-85418755 ; Tel: +86-28-85412890; b State Key Laboratory of Supramolecular Structure and Materials , Jilin University , Changchun 130012 , China; c Department of Chemistry , The State University of New York , Buffalo , New York 14260 , USA

## Abstract

Examples of using two-dimensional shape-persistent macrocycles, *i.e.* those having noncollapsible and geometrically well-defined skeletons, for constructing mechanically interlocked molecules are scarce, which contrasts the many applications of these macrocycles in molecular recognition and functional self-assembly. Herein, we report the crucial role played by macrocyclic shape-persistency in enhancing multipoint recognition for the highly efficient template-directed synthesis of rotaxanes. Cyclo[6]aramides, with a near-planar conformation, are found to act as powerful hosts that bind bipyridinium salts with high affinities. This unique recognition module, composed of two macrocyclic molecules with one bipyridinium ion thread through the cavity, is observed both in the solid state and in solution, with unusually high binding constants ranging from ∼10^13^ M^–2^ to ∼10^15^ M^–2^ in acetone. The high efficacy of this recognition motif is embodied by the formation of compact [3]rotaxanes in excellent yields based on either a “click-capping” (91%) or “facile one-pot” (85%) approach, underscoring the great advantage of using H-bonded aromatic amide macrocycles for the highly efficient template-directed synthesis of mechanically interlocked structures. Furthermore, three cyclo[6]aramides bearing different peripheral chains **1–3** demonstrate high specificity in the synthesis of a [3]rotaxane from **1** and **2**, and a [2]rotaxane from **3** *via* a “facile one-pot” approach, in each case as the only isolated product. Analysis of the crystal structure of the [3]rotaxane reveals a highly compact binding mode that would be difficult to access using other macrocycles with a flexible backbone. Leveraging this unique recognition motif, resulting from the shape-persistency of these oligoamide macrocycles, in the template-directed synthesis of compact rotaxanes may open up new opportunities for the development of higher order interlocked molecules and artificial molecular machines.

## Abstract 2

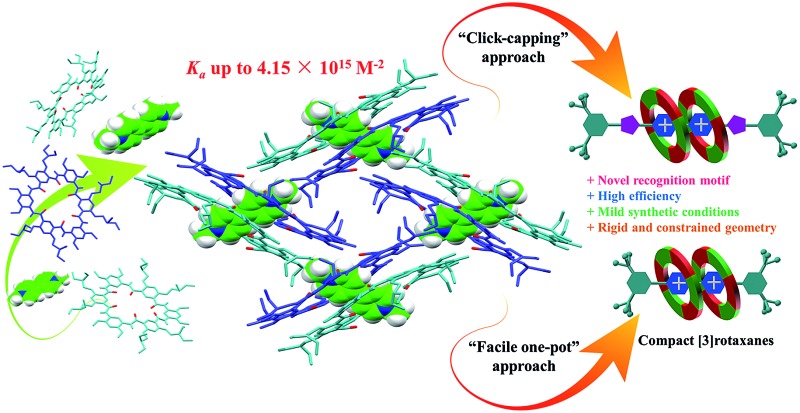
The importance of macrocyclic shape-persistency in novel host–guest systems for the highly efficient template-directed synthesis of rotaxanes has been revealed.

## Introduction

The development of mechanically interlocked molecules (MIMs) has invariably been accompanied by emerging recognition motifs and template-directed synthetic protocols.^1^ Among various factors, the combination of a macrocycle and a thread component with sufficient binding affinity constitutes a crucial determinant to create such motifs. In this regard, many elegant examples have been reported. These include the use of macrocyclic hosts such as crown ethers,^2^ tetracationic cyclophanes,^3^ calixarenes,^4^ cucurbiturils,^5^ cyclodextrins,^6^ and pillarenes^7^ to interact with the chosen thread components. However, uncovering simple and efficient recognition motifs still represents a grand challenge for the synthesis of MIMs, especially those involving non-metal coordination for extremely compact interlocked molecules, with high atom economy.

Thus far, most known examples of highly efficient, template-directed synthesis of [3]rotaxanes have relied on metal–ligand coordination.^8^ In all these cases, the “wheels” employed are based on macrocyclic molecules that are nonplanar and fairly flexible conformationally. In recent years, shape-persistent macrocycles, with noncollapsible and geometrically well-defined skeletons, such as phenylacetylene macrocycles,^9^ have attracted considerable attention due to their intriguing functions, including supramolecular gelation,^10^ channelized transportation,^11^ organic catalysis,^12^ molecular recognition^13^ and multifunctional self-assembly.^14^ Many shape-persistent macrocycles have well-defined surface topography and nanosized cavities with preorganized binding sites, and thus may exhibit enhanced complexation.^15^ Inspired by the pioneering work on synthesizing MIMs using flexible aromatic amide macrocycles^16^ or H-bonded oligoamide foldamers,^17^ we reasoned that a shape-persistent macrocycle, when serving as a wheel component for a MIM, may overcome the limit of known systems by engaging effectively in cooperative recognition interactions. However, introducing two-dimensional (2D) shape-persistent macrocycles into interlocked structures still presents a significant challenge. To the best of our knowledge, few examples of MIMs based on these macrocycles are known,^18^ especially [3]rotaxanes. In fact, there is only one report, based on a pentagonal cyanostar macrocycle, on forming [3]rotaxanes.^18*a*^

**Scheme 1:**
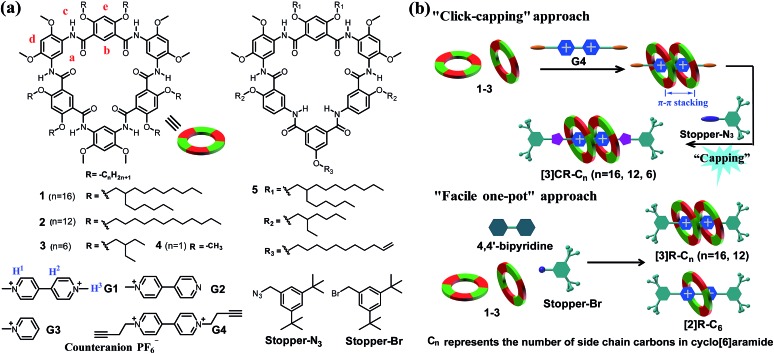
(a) Chemical structures of cyclo[6]aramides **1–5**, guests **G1–G4**, **Stopper-N_3_** and **Stopper-Br**. (b) Schematic illustration of approaches for constructing compact [3]rotaxanes based on cyclo[6]aramides **1–3**.

H-Bonded aromatic amide macrocycles^19^ are a large class of recently emerged 2D shape-persistent cyclic compounds featuring full amide linkages with rigid backbones enforced through intramolecular hydrogen bonds. Among them, cyclo[6]aramides, the smallest member of the aromatic oligoamide macrocycles^20^ with the amide carbonyl oxygens pointing inwards, are particularly noteworthy. They resemble a crown ether in their oxygen-rich cavities, but differ considerably in their conformational rigidity. In particular, the highly favorable intramolecular H-bond assisted macrocyclization strategy makes numerous geometrically well-defined macrocycles readily available, which facilitates their further applications. With a well-defined cavity of *ca.* 8 Å in diameter, large π-surfaces and precisely positioned binding sites, these macrocycles have displayed rich host–guest (H–G) chemistry. Our recent studies revealed that cyclo[6]aramides could bind organic cations and hydrogen bond donors.^21^ Especially notable is the complex consisting of such macrocycles and diquat, an isomer of paraquat, with 2 : 1 stoichiometry, in which a diquat molecule resides between two macrocyclic molecules rather than threads into their cavities due to electronic repulsion and steric hindrance.^22^ Paraquat is one of the most widely used guests for studying H–G interactions.^23^ Despite numerous reports on paraquat recognition, threading two rings on a single paraquat molecule with high binding affinity both in solution and in the solid state is still very difficult to achieve. The cryptand reported by Gibson and co-workers could form a 2 : 1 H–G complex only observed in the crystal structure and the highest binding constant for 1 : 1 stoichiometry achieved so far is 5.0 × 10^6^ M^–1^ in acetone.^24^ A crown ether, bis-*p*-xylyl[26]crown-6, was used by Chiu and co-workers to form a [3]pseudorotaxane-like complex both in solution and in the solid state. However, only a very low binding constant (*K*_1_ = 700 M^–1^ and *K*_2_ = 60 M^–1^) was observed in acetonitrile.^25^ So far no successful examples have been reported on creating a tight 2 : 1 binding motif for constructing constrained [3]rotaxanes based on a single paraquat unit. Herein we report that cyclo[6]aramides, with their persistent shape and geometrically well-defined electron-rich cavity (Scheme 1a), could act as powerful hosts for the tight binding of bipyridinium salts in a 2 : 1 binding mode both in the solid state and in solution with exceedingly high binding constants. More importantly, this unique recognition motif has led to the template-directed synthesis of compact [3]rotaxanes in excellent yields based on either a “click-capping” or “facile one-pot” approach (Scheme 1b).

## Results and discussion

### Evidence for 2 : 1 cooperative host–guest complexation

**Fig. 1:**
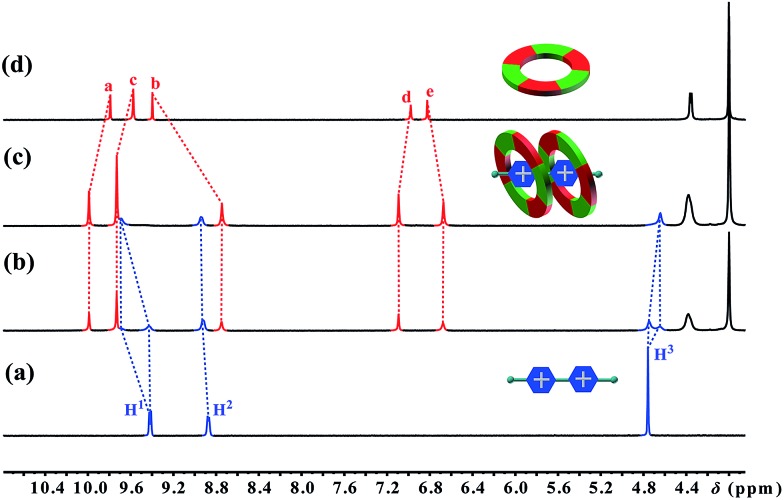
Partial ^1^H NMR spectra (400 MHz, acetone-d_6_, 298 K) of (a) 2.0 mM **G1**, (b) 2.0 mM **1** and **G1**, (c) 4.0 mM **1** and 2.0 mM **G1**, and (d) 2.0 mM **1**.

The first sign indicating the H–G interaction came from a color change upon adding paraquat **G1** to a solution of cyclo[6]aramide **1** in acetone. An abrupt change from clear to light yellow was observed, indicating that a charge transfer (CT) interaction happened between **1** and **G1**. A CT band in the UV-vis absorption spectrum confirms the formation of the H-G inclusion complex (Fig. S69†). Then, the formation of the H–G complex was further explored using ^1^H NMR spectroscopy (Fig. 1). When 1.0 equiv. of **1** was added to a 2.0 mM solution of **G1**, two sets of signals from the bipyridinium ion were observed, which evolved into one set of signals with 2 equiv. of **1**, indicating the slow exchange of the complex on the NMR time scale. Commensurate with this change is the appreciable shift for the aromatic and amide protons of the host upon guest binding. The binding event was supported with 2D nuclear overhauser effect spectroscopy (NOESY), which revealed through-space NOEs between bipyridinium protons and the internal aromatic protons H^a^ and H^b^ of **G1–G4** (Fig. S55–S64†). Such through-space NOE contacts can transpire only if these two macrocycles are mutually parallel but orthogonal to **G1**. Two-dimensional diffusion ordered spectroscopic (2D-DOSY) analysis provided additional evidence for the formation of very stable complexes between **1** and **G1–G4** (Fig. S65–S68†). For each complex, the protons of **1** and the guest have the same diffusion constant in solution. Furthermore, Job plots supplied information on the binding stoichiometry of **1** and **G1–G4** in solution (Fig. S71–S78†). For example, in the case of the complex **1**_2_ ⊃ **G1**, the maximum change in absorbency was observed at 0.67, indicating a macrocycle-cation ratio of 2 : 1. Examining the 2 : 1 mixture of **1** and **G1** using matrix-assisted laser ionization time of flight mass spectrometry (MALDI-TOF-MS) uncovered the peak with the highest intensity at *m*/*z* = 5005.219, corresponding to the complex [**1**_2_ + **G1** – PF_6_]^+^. The above results, taken in concert, clearly demonstrate the formation of a 2 : 1 complex **1**_2_ ⊃ **G1**. The same 2 : 1 stoichiometry was observed with guests **G2** and **G4**, but **G3** only shows 1 : 1 stoichiometry with **1** (Fig. S103–S109†). Another interesting observation made on complex **1**_2_ ⊃ **G1** is its reversible redox-responsiveness, which was realized by the addition and removal of Zn powder (Fig. S143†).

The binding constants *K*_1_ and *K*_2_ for the complexation of cyclo[6]aramide **1** with bipyridinium salts **G1–G4** were obtained using UV-vis titration methods (Table 1). The UV-vis titration experiments revealed surprisingly high binding constants, *K*_1_ = 3.49 × 10^7^ M^–1^ and *K*_2_ = 1.09 × 10^6^ M^–1^, for complex **1**_2_ ⊃ **G1**. The binding of **G1** with the second macrocycle is accompanied by a slightly negative cooperative effect (4*K*_2_/*K*_1_ < 1),^26^ which is probably caused by the remaining pyridinium unit that has become less electron deficient after the threading of the first macrocycle. To provide insight into the role of the second pyridinium moiety when the first site is occupied, **G2**, with one positive charge, was examined for binding to **1**. Results from UV-vis titration show that, as compared to those of **1**_2_ ⊃ **G1**, the binding constants for **G2** and **1** are drastically reduced to *K*_1_ = 3.62 × 10^4^ M^–1^ and *K*_2_ = 2.28 × 10^4^ M^–1^, which indicates the pivotal role played by the second positive charge of **G1** for the high stability of complex **1**_2_ ⊃ **G1**. In addition, the positive cooperativity (4*K*_2_/*K*_1_ = 2.51) observed for **1**_2_ ⊃ **G2**, along with the similar *K*_a_ values of complex **1** ⊃ **G3** to that of *K*_1_ for **1**_2_ ⊃ **G1**, suggests that additional inter-macrocycle π–π stacking may also assist the binding of the second macrocycle in the formation of **1**_2_ ⊃ **G2**. It is worth noting that **G4** gives the highest binding constants for binding **1** (*K*_1_ = 1.68 × 10^8^ M^–1^ and *K*_2_ = 2.47 × 10^7^ M^–1^), which should greatly facilitate the synthesis of rotaxanes (Fig. S83–S95 and Table S1†). A similar trend in binding constants was observed in a competitive solvent (acetone-d_6_/DMSO-d_6_, 9/1, v/v) (Table 1, Fig. S94 and S95†). Additional infrared experiments on the complexes prepared from pyridinium salts and macrocycle **1** show that the C=O stretching frequency shift induced by complex formation is in the order of **G4** > **G1** > **G2** > **G3** (Fig. S110–S113 and Table S1†), which agrees well with the difference in the binding affinities of these guests with macrocycle **1**. In order to demonstrate the crucial role played by shape persistency, cyclo[6]aramide **5**, which is partially H-bonded and bears two rotatable amide groups, was synthesized. Since the depletion of partial intramolecular H-bonds results in free rotation of the two amide groups, the shape-persistency as observed in **1** should be attenuated to a significant extent. Indeed, the results from the binding experiments show that it binds **G4** in a 2 : 1 binding mode (Fig. S81–S82†) with binding constants of *K*_1_ = 7.29 × 10^4^ M^–1^ and *K*_2_ = 2.50 × 10^3^ M^–1^ (Table 1 and Fig. S95 and S96†), which are four orders of magnitude lower than that of complex **1**_2_ ⊃ **G4**. This significantly reduced binding affinity as compared to **1** strongly suggests the importance of shape-persistency in the binding event. Despite the great advances made in the past decades, few recognition modules have shown such unusually strong binding in organic media.

**Table 1:** Binding constants of bipyridinium salts **G1–G4** with cyclo[6]aramide **1** ^*a*^

Complex	Solvent	Molar ratio^*b*^	*K*_1_ (M^–1^)	*K*_2_ (M^–1^)	*K*_12_ (M^–2^)	Cooperativity *α* (4*K*_2_/*K*_1_)^*c*^
**1**_2_ ⊃ **G1**	Acetone	2 : 1	3.49 × 10^7^	1.09 × 10^6^	3.80 × 10^13^	0.12
**1**_2_ ⊃ **G1**	Acetone/DMSO (9/1, v/v)	2 : 1	3.29 × 10^6^	3.72 × 10^5^	1.22 × 10^12^	0.45
**1**_2_ ⊃ **G2**	Acetone	2 : 1	3.62 × 10^4^	2.28 × 10^4^	8.25 × 10^8^	2.51
**1** ⊃ **G3**	Acetone	1 : 1	1.20 × 10^4^	—	—	—
**1**_2_ ⊃ **G4**	Acetone	2 : 1	1.68 × 10^8^	2.47 × 10^7^	4.15 × 10^15^	0.58
**1**_2_ ⊃ **G4**	Acetone/DMSO (9/1, v/v)	2 : 1	8.98 × 10^6^	2.14 × 10^6^	1.92 × 10^13^	0.95
**5**_2_ ⊃ **G4**	Acetone-d_6_	2 : 1	[7.29 × 10^4^]^*d*^	[2.50 × 10^3^]^*d*^	[1.82 × 10^8^]^*d*^	0.13

^*a*^The binding constant values were obtained through UV-vis titration experiments using a nonlinear fit curve calculated using Matlab 801. For each titration, at least 30 data points were collected. Typically, wavelength was monitored around the charge transfer absorption maxima for the complex formed, estimated errors for *K*_1_ and *K*_2_ = ±50%.

^*b*^The stoichiometry was determined using Job plots based on NMR experiments.

^*c*^The cooperativity with the interaction parameter a according to the equation *α* = 4*K*_2_/*K*_1_.

^*d*^The binding constants were obtained using NMR titration experiments. For full details, see the ESI.

### X-ray crystal structure of [3]pseudorotaxane **3**_2_ ⊃ **G1**

**Fig. 2:**
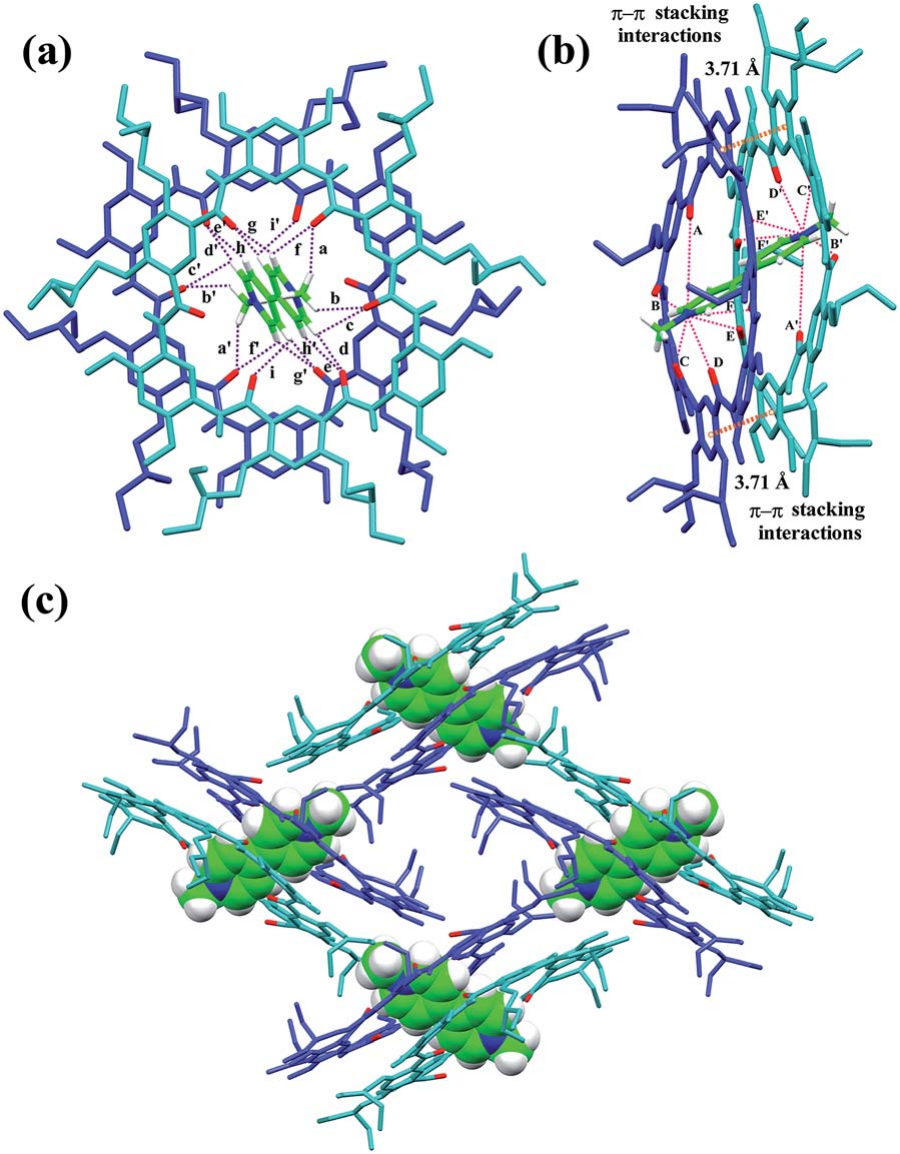
X-ray crystal structure of complex **3**_2_ ⊃ **G1** shown from (a) top and (b) side views. PF_6_^–^ counterions and hydrogen atoms except the ones involved in hydrogen bonding were omitted for clarity. The dashed purple lines indicate the C–H···O hydrogen bonds (a–i) and (a′–i′), parameters are as follows, H···O distance (Å), C–H···O angles (deg): (a and a′) 2.63, 133.7; (b and b′) 2.64, 116.3; (c and c′) 2.66, 106.7; (d and d′) 2.55, 125.2; (e and e′) 2.64, 120.8; (f and f′) 2.42, 109.1; (g and g′) 2.30, 128.9; (h and h′) 2.74, 110.8; (i and i′) 2.65, 111.1. The dashed pink lines indicate the N^+^···O interactions (A–F) and (A′–F′), parameters are as follows, N^+^···O distance (Å): (A and A′) 4.73; (B and B′) 4.20; (C and C′) 3.35; (D and D′) 3.32; (E and E′) 4.81; (F and F′) 4.46. Face to face π-stacking parameters: centroid–centroid distance (Å), 3.71; ring plane–ring plane inclination (deg), 4.42. (c) Crystal packing structure of complex **3**_2_ ⊃ **G1**. **G1** is shown in capped stick model or space filling model. Oxygen atoms in the cavity of the macrocycles are shown in red. PF_6_^–^ counterions and hydrogen atoms have been removed for clarity.

The slow evaporation of a solution containing **3** and **G1** in a mixed solvent of acetone/CHCl_3_/methanol (10/1/0.3) afforded red crystals in 7 weeks. The analysis of the resulting solid state structure using single-crystal X-ray diffraction (XRD) experiments reveals that **G1** is inserted into two macrocyclic molecules. In this complex, each of the pyridinium units is orientated orthogonally with respect to the two macrocycles and engages in multiple C–H···O H-bonding (Fig. 2a) and N^+^···O ion–dipole interactions that are reinforced by face to face π–π stacking interactions (3.71 Å) (Fig. 2b). These hydrogen bonds all have very short donor–acceptor distances ranging from 2.30 to 2.74 Å due to the rigid macrocyclic backbone constrained by intramolecular three-centre H-bonds. The length of the N^+^···O ion–dipole interactions varying from 3.32 to 4.73 Å is indicative of strong Coulomb interaction between the amide oxygen atoms and pyridinium cations. Therefore, the strong binding affinity between cyclo[6]aramide **1** and the bipyridinium guests is attributed to the result of the cooperative interplay of multipoint non-covalent forces. The geometrically well-defined and tightly packed solid-state structure of **3**_2_ ⊃ **G1** is also observed which is stabilized by multiple van der Waals forces between the side chains of macrocycle **3** and **G1** (Fig. 2c).

### “Click-capping” approach for the synthesis of rotaxanes

Click reactions, with their high efficiency and simplicity, are widely used for constructing MIMs.^27^ Buoyed by the observed tight binding, we anticipated that compact rotaxanes **[3]CR-C*_n_*** (*n* = 16, 12 and 6) might be attained by tethering **Stopper-N_3_** to the [3]pseudorotaxane formed from cyclo[6]aramides **1–3** and guest **G4** *via* a “click-capping” approach. Indeed, the templation reaction proceeded very well, leading to the specific formation of [3]rotaxanes (Table 2, entries 1–3). In the case of macrocycle **1**, which is decorated peripherally with sterically crowded alkyl groups, the reaction offers [3]rotaxane **[3]CR-C_16_** as the sole product in a yield of 86%. Using macrocycle **2**, which tends to severely aggregate in solution due to the presence of linear alkyl chains,^28^ the yield of [3]rotaxane **[3]CR-C_12_** was increased up to 91%. To the best of our knowledge, this is one of the rare examples of the synthesis of [3]rotaxanes in excellent yields reported hitherto using a non-metal coordination strategy.^29^ Due to its low solubility, macrocycle **3** forms **[3]CR-C_6_** in a yield of 64%. Varying the solvent polarity and the ratios of reactants resulted in the controlled generation of [3]rotaxanes as the favourable product (Table 2, entries 4 and 5). To probe which binding mode (1 : 1 or 2 : 1) is more favourable for forming rotaxanes, a mixture of 1 equiv. of **1** and 1 equiv. of guest **G4** was stirred in the presence of a catalyst. We speculated that the formation of [2]rotaxane should have dominated^25^ under this condition; however, besides [2]rotaxane in 34% yield, the yield of [3]rotaxane **[3]CR-C_16_** still reached 36%, indicating that the recognition event favours 2 : 1 stoichiometry, rather than 1 : 1, during the binding process. The highly selective formation of [3]rotaxanes with cyclo[6]aramides originates from the combination of exceedingly high binding affinities and the compact self-assembly mode. These features are rarely observed in other similar H–G systems. In addition, face to face π–π stacking interactions between the two near-planar macrocycles as observed in the crystal structure of **3**_2_ ⊃ **G1** could provide an additional driving force that contributes to the high efficiency. It is worth noting that the preparation of [3]rotaxanes with shape-persistent macrocycles as “wheels” in such a high yield (>90%) under non-metal coordination conditions is preceded by few examples and remains a formidable task.^18a,30^

**Table 2:** “Click-capping” approach for the synthesis of rotaxanes **[3]CR-C*_n_***

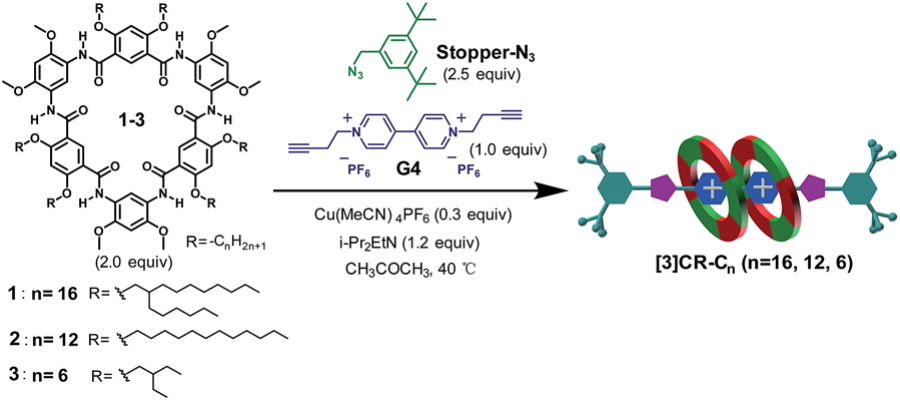					
Entry	Macrocycle	*n* ^*d*^	Yield^*e*^ (%)		[3]R : [2]R
			[3]R	[2]R	
1^*a*^	**1**	16	86	n.d.	>99 : 1
2^*a*^	**2**	12	91	n.d.	>99 : 1
3^*a*^	**3**	6	64	Trace^*f*^	>99 : 1
4^*a*^ ^,^ ^*b*^	**1**	16	36	34	35 : 65
5^*c*^	**1**	16	60	18	66 : 34

^*a*^Solvent is acetone, 40 °C, 24 h. Pre-mixing Cu(CH_3_CN)_4_PF_6_ and the macrocycle prior to the reaction did not lead to any difference in the rotaxane formation as compared to the common synthetic procedure in the Experimental section.

^*b*^Only 1.0 equiv. of macrocycle **1** was used.

^*c*^Solvent is acetone/acetonitrile (1/1, v/v), 40 °C, 48 h.

^*d*^ *n* is the number of side chain carbons of the macrocycle.

^*e*^Isolated yield after chromatography. The yield of [2]/[3]rotaxane was calculated based on the macrocycle.

^*f*^Observed using MALDI-TOF-MS. n.d. = not detected. For full details, see the ESI.

### “Facile one-pot” approach for the synthesis of rotaxanes

The “facile one-pot” reaction, characterized by simply mixing and heating, and the absence of metallocatalysts, is another useful approach in the synthesis of rotaxanes with high yields.^31^ The synthesis of [3]rotaxanes **[3]R-C*_n_*** (*n* = 16 and 12) or [2]rotaxane **[2]R-C_6_** based on this method was achieved through mixing 2.0 equiv. of cyclo[6]aramide, 2.5 equiv. of **Stopper-Br** and 1.0 equiv. of 4,4′-bipyridine in CHCl_3_/CH_3_CN (1/1, v/v) (Table 3, entries 1–3). Highly efficient template-directed synthesis with macrocycle **1** or **2** was achieved with an excellent yield of 85%, which is rare in the known synthesis of [3]rotaxanes.^32^ Particularly noticeable was the formation of a [2]rotaxane as the only product when macrocycle **3**, which bears short side chains, was used. This specificity in [2]rotaxane formation is rationalized according to the sparse dissolution of the macrocycle in the solution. Compound **3** alone was insoluble in CHCl_3_/CH_3_CN (1 : 1, v/v). However, gradual dissolution was observed to occur as the reaction progressed. This suggests a scanty concentration of macrocycle **3** in the reaction system as compared to that of macrocycle **1** or **2**. Since the efficiency of forming [3]rotaxanes depends predominantly on the effective molar ratio (2 : 1) of the macrocycle and cationic guest in solution, the limited concentration of macrocyclic molecules with respect to that of the cationic axle tends to facilitate a binding process that favours a 1 : 1 binding mode, thereby leading to the specific formation of a [2]rotaxane.

**Table 3:** “Facile one-pot” approach for the synthesis of rotaxanes **[3]R-C*_n_***

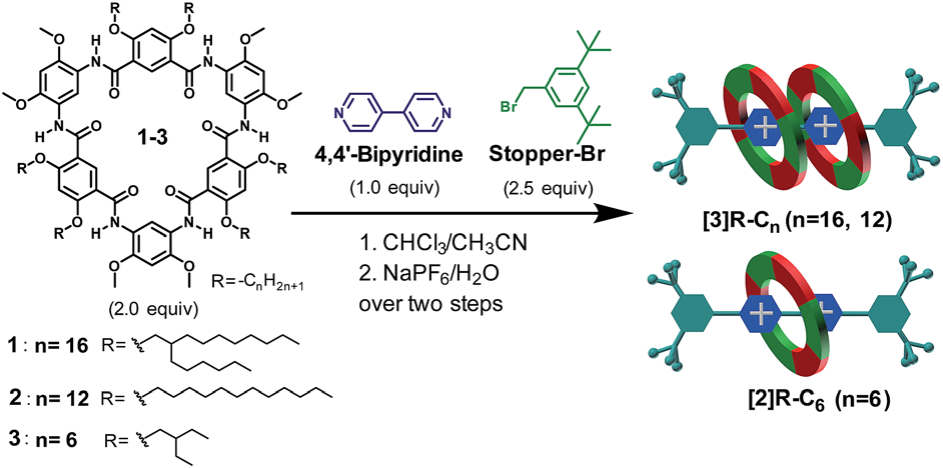					
Entry	Macrocycle	*n* ^*b*^	Yield^*c*^ (%)		[3]R : [2]R
			[3]R	[2]R	
1^*a*^	**1**	16	85	n.d.	>99 : 1
2^*a*^	**2**	12	85	n.d.	>99 : 1
3^*a*^	**3**	6	n.d.	71	>1 : 99

^*a*^Solvent is acetonitrile/CHCl_3_ (1/1, v/v), 40 °C, 24 h.

^*b*^ *n* is the number of side chain carbons of the macrocycle.

^*c*^Isolated yield after chromatography. The yield of [2]/[3]rotaxane was calculated based on the macrocycle. n.d. = not detected. For full details, see the ESI.

### Structural characterization of [2]/[3]rotaxanes

**Fig. 3:**
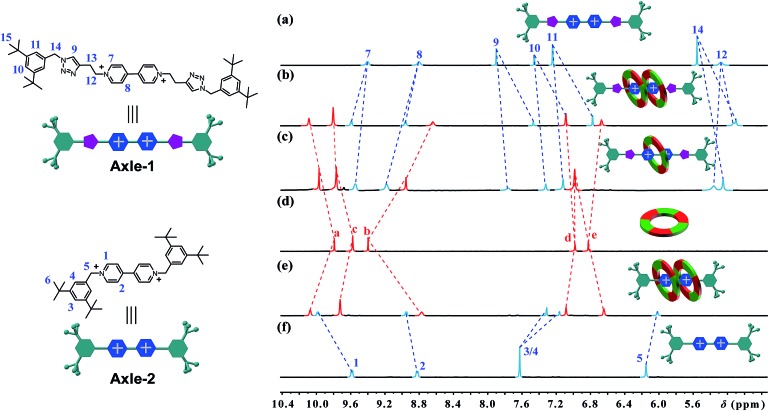
Partial ^1^H NMR spectra of (a) **Axle-1**, (b) **[3]CR-C_16_**, (c) **[2]CR-C_16_**, (d) cyclo[6]aramide **1**, (e) **[3]R-C_16_** and (f) **Axle-2** (400 MHz, acetone-d_6_, 298 K). The counterion is PF_6_^–^.

**Fig. 4:**
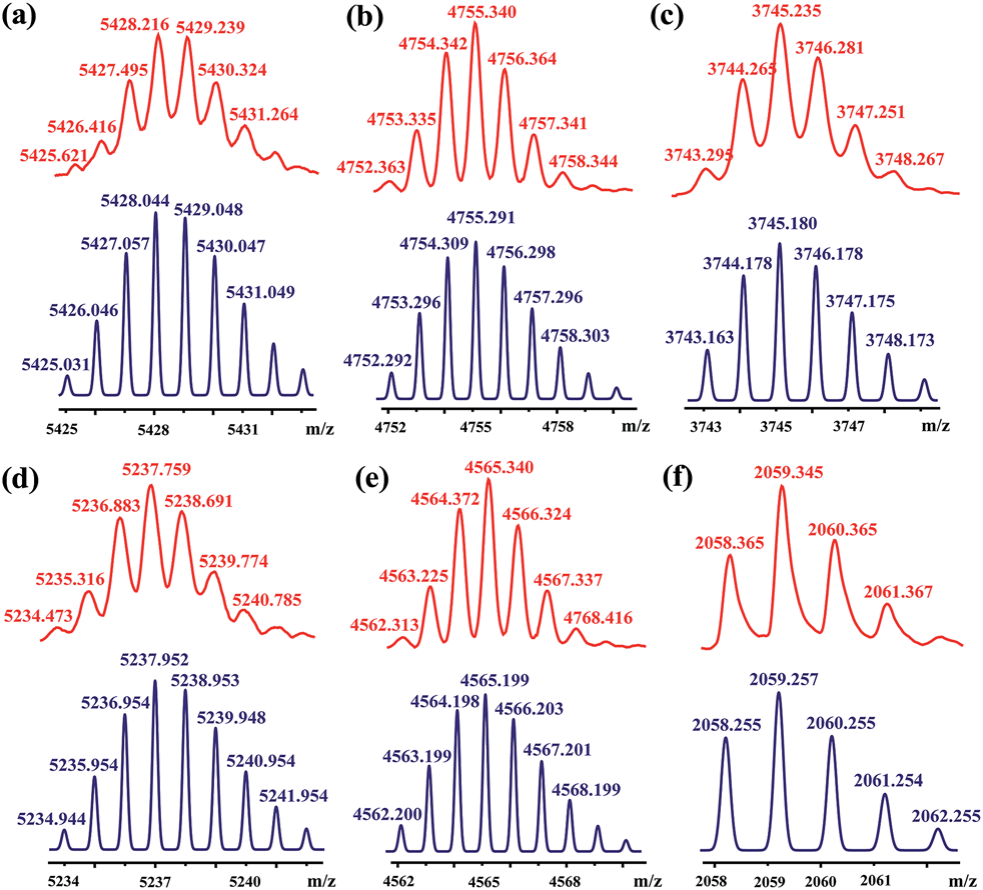
MALDI-TOF-MS spectra of rotaxanes (a) **[3]CR-C_16_**, (b) **[3]CR-C_12_**, (c) **[3]CR-C_6_**, (d) **[3]R-C_16_**, (e) **[3]R-C_12_** and (f) **[2]R-C_6_** (top, red), and their simulated spectra (bottom, blue).

[3]Rotaxanes **[3]CR-C*_n_*** (*n* = 6, 12 and 16) and **[3]R-C*_n_*** (*n* = 12 and 16), and [2]rotaxanes **[2]CR-C_16_** and **[2]R-C_6_**, were fully characterized using ^1^H and ^13^C NMR spectroscopy (Fig. S17–S32†) and mass spectrometry (Fig. S38–S44†). For example, the threaded structures of **[3]CR-C_16_**, **[2]CR-C_16_** and **[3]R-C_16_** are apparent by comparing their ^1^H NMR spectra (Fig. 3b, c and e) with those of the free threads **Axle-1** and **Axle-2** (Fig. 3a and f), and macrocycle **1** (Fig. 3d). The thread protons 7 and 8 in **[3]R-C_16_** are shifted downfield due to C–H···O H-bonding. The shielding of protons 9, 10, 11 and 14 on **Axle-1**, along with the results from 2D NOESY, HSQC, HMBC, and DOSY NMR experiments, indicates the relative positions of the components and the formation of mechanical bonds (Fig. S118–S141†). MALDI-TOF-MS provided additional evidence for the formation of mechanically interlocked structures. In the mass spectra of **[3]CR-C_16_**, **[3]CR-C_12_** and **[3]CR-C_6_**, the highest related peaks were observed at *m*/*z* = 5427.495, *m*/*z* = 4753.335 and *m*/*z* = 3744.265, which support the structural assignments for the formation of [3]rotaxanes (Fig. 4a–c). Furthermore, related peaks were also observed in the mass spectra of **[3]R-C_16_**, **[3]R-C_12_** and **[2]R-C_6_** at *m*/*z* = 5236.883, *m*/*z* = 4563.225 and *m*/*z* = 2058.365, pointing to the formation of a [3]rotaxane or [2]rotaxane (Fig. 4d–f). In all cases, all the peaks were isotopically resolved and fully agreed with their calculated theoretical distributions. It should be noted that all the rotaxanes are in red due to charge-transfer interactions between the macrocycles and axles, as indicated by the CT band observed in the UV-vis absorption spectra (Fig. S142†).

### X-ray crystal structure of **[3]CR-C_6_**

**Fig. 5:**
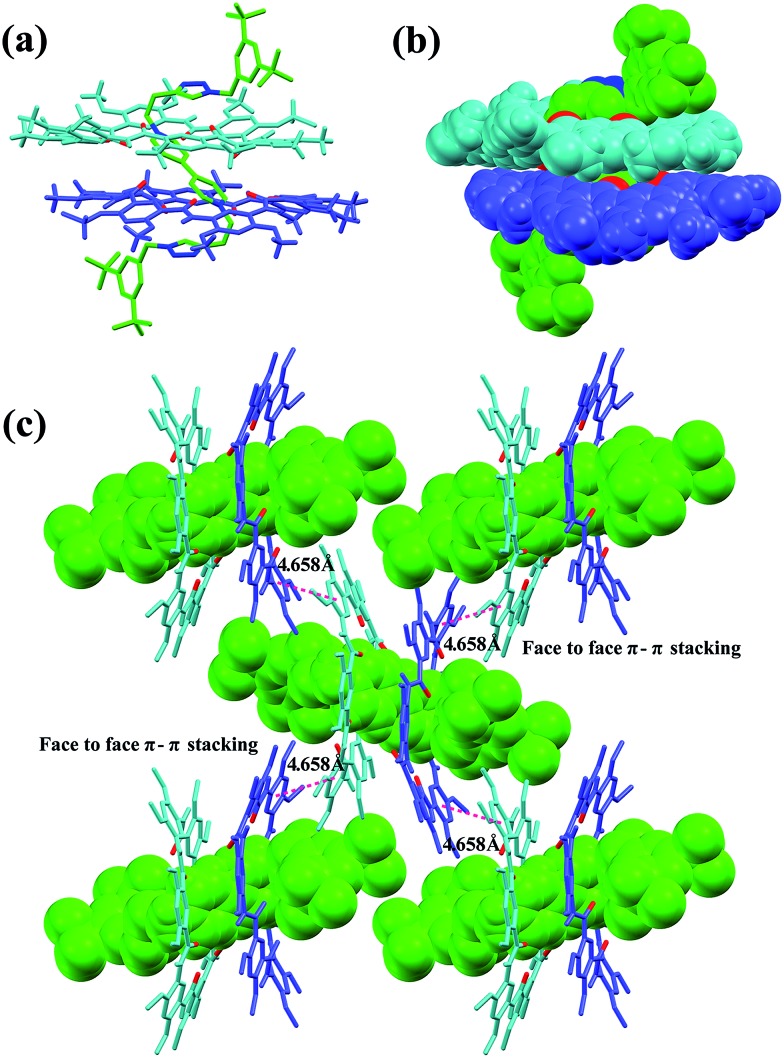
X-ray crystal structure of **[3]CR-C_6_** is shown in (a) capped stick model and (b) space-filling model. (c) Crystal packing structure of **[3]CR-C_6_**. Face to face π-stacking parameters are shown as dashed magenta lines: centroid–centroid distance (Å), 4.658; ring plane–ring plane inclination (deg), 2.96. Oxygen atoms in the cavity of the macrocycles are shown in red. PF_6_^–^ counterions, hydrogen atoms and side chains have been removed for clarity.

Single crystals of [3]rotaxane **[3]CR-C_6_** were obtained by slowly diffusing methanol into an acetone solution containing **[3]CR-C_6_** in about 8 weeks. The X-ray structure of **[3]CR-C_6_** clearly shows that the thread **Axle-1** penetrates through the cavities of the two neighboring macrocyclic molecules in a zigzag conformation (Fig. 5a and b). The mechanically interlocked structure is stabilized by twenty C–H···O H-bonds and twelve N^+^···O ion dipole interactions (Tables S4 and S5†). There are also a number of very weak face-to-face π–π stacking interactions (4.658 Å) between the macrocyclic molecules, resulting in a well-ordered and tightly packed solid-state structure (Fig. 5c). The numerous noncovalent forces revealed from the X-ray structure work cooperatively, leading to the surprisingly high stability of the [3]rotaxanes. In fact, when a mixture of **Axle-1** and macrocycle **1** was heated under reflux for 3 hours in acetone-d_6_/DMSO-d_6_ (9 : 1, v/v), the ^1^H NMR spectra did not show any signs of the presence of rotaxanes, indicating the fact that threading does not occur (Fig. S117†), and thus it can be inferred that [3]rotaxane **[3]CR-C_6_** is unlikely to experience a dethreading process under the conditions specified. The high stability is also demonstrated by the observation that complex **1**_2_ ⊃ **G1** (or **1**_2_ ⊃ **G4**) and free macrocycle **1** were clearly seen for each of them on a TLC plate. Upon the addition of 10.0 equiv. of diethylamine (DEA) to **[3]R-C_16_** or **[2]R-C_6_** in acetone, only **[2]R-C_6_** caused a color change from light yellow to blue, indicating the insensitivity of **[3]R-C_16_** to redox responsiveness. Interestingly, trifluoroacetic acid (TFA) can reverse the redox process of **[2]R-C_6_** (Fig. S144†).

### Computational simulation of **[3]R-C_1_**

Since the growth of single crystals of the [3]rotaxane **[3]R-C*_n_*** (*n* = 16, 12 and 6) synthesized according to the “facile one-pot” approach has proved to be extremely challenging, we resorted to molecular mechanics simulations to gain a better understanding of the noncovalent bonding interactions that direct rotaxane formation and stability. Further structural insights on [3]rotaxane **[3]R-C_1_** were obtained through computational simulations based on the DFT method. Our computational study indicated that [3]rotaxane **[3]R-C_1_** was built by assembling the two macrocyclic molecules of **4** in a near-planar conformation and the central motif **Axle-1** in an interlocked orthogonal binding arrangement, which is in good agreement with the structure obtained from the single crystal of **[3]CR-C_6_**. Furthermore, multiple C–H···O H-bonds and N^+^···O ion–dipole interactions are also observed in the modelling structure which direct the [3]rotaxane formation (Fig. S153†). It is worth noting that there are no face to face π–π stacking interactions formed between the two macrocycles, which is the same as the observation from the crystal structure of **[3]CR-C_6_**.

## Conclusions

In conclusion, we have demonstrated the unusually high binding affinity (*K*_a_ from ∼10^13^ M^–2^ to ∼10^15^ M^–2^ in acetone) of a novel threaded recognition motif comprising cyclo[6]aramides and bipyridinium salts in 2 : 1 (H : G) stoichiometry. The crystal structure of the [3]pseudorotaxane shows clear evidence of the high binding affinity, which is attributed as the result of the cooperative interplay of multipoint C–H···O H-bonds, N^+^···O ion–dipole interactions and π–π stacking interactions between the two neighboring macrocycles. Furthermore, the highly efficient synthesis of compact [3]rotaxanes achieved using either a “facile one-pot” or “click-capping” approach presents a rare example of constructing MIMs based on 2D shape-persistent macrocycles. The high efficiency of the formation of these rotaxanes highlights the unique advantage of macrocyclic shape-persistency, which results in enhanced multipoint recognition for the highly efficient synthesis of compact mechanically interlocked molecules. The concept of utilizing macrocyclic shape-persistency for boosting multipoint binding affinities for the template-directed synthesis of rotaxanes might be useful for the design of novel MIMs and the development of artificial molecular machines.

## Experimental section

### General experimental procedure for the “click-capping” approach for the synthesis of **[3]CR-C*_n_*** (*n* = 16, 12 and 6)

A mixture of cyclo[6]aramide (2.0 equiv.), guest **G4** (1.0 equiv.) and Cu(CH_3_CN)_4_PF_6_ (0.3 equiv.) was stirred in dry acetone at room temperature for 20 minutes under N_2_. Then a solution of **Stopper-N_3_** (2.5 equiv.) and *N*,*N*-diisopropylethylamine (DIPEA) (1.2 equiv.) was injected. The mixture was further stirred at 40 °C for 24 h. The resulting solution was washed with 16% aqueous EDTA tetra-sodium saturated ammonia solution (2 × 50 mL). The organic layer was retained and the aqueous layer extracted twice with CH_2_Cl_2_ (2 × 50 mL). The organic extracts were combined and washed with water, dried over Na_2_SO_4_ and dried *in vacuo*. Removal of the solvent afforded a red solid and the crude material was purified using flash column chromatography with silica gel (CHCl_3_/CH_3_OH, 20 : 1) to yield the corresponding red solid. The detailed synthetic procedures and full characterization of new compounds are provided in the ESI.†

### General experimental procedure for the “facile one-pot” approach for the synthesis of **[3]R-C_16_**, **[3]R-C_12_** and **[2]R-C_6_**

A mixture of cyclo[6]aramide (2 equiv.), 3,5-di-*tert*-butylbenzyl bromide **Stopper-Br** (2.5 equiv.) and 4,4′-bipyridine (1 equiv.) was stirred in 6 mL of CHCl_3_/CH_3_CN (1/1, v/v) under N_2_ at 40 °C for 48 h. Removal of solvents afforded a pale red solid, which was dissolved in acetone/H_2_O, followed by the addition of saturated aqueous NaPF_6_. After stirring for 30 min, the organic solvent was then evaporated under reduced pressure. The precipitate was collected and washed with H_2_O. Then the crude material was purified using flash column chromatography with silica gel (CHCl_3_/CH_3_OH, 30 : 1, then CHCl_3_/CH_3_OH, 10 : 1) to yield the corresponding red solid. The detailed synthetic procedures and full characterization of new compounds are provided in the ESI.†

### Single X-ray crystal data

Crystallographic data (excluding structure factors) for all the structures reported in this article have been deposited with the Cambridge Crystallographic Data Centre in CIF (see the ESI†). Crystal data for **3**_2_ ⊃ **G1** (CCDC-; 1475246): C_90_H_121_N_7_O_18_PF_6_, *M* = 1733.91, monoclinic, space group *P*2_1_/*c*, *a* = 22.630(8), *b* = 23.529(9), and *c* = 19.425(7) Å, *α* = 90, *β* = 112.979(5), and *γ* = 90°, *V* = 9522(6) Å^3^, *Z* = 4, *T* = 123(2) K, *μ*(Mo K_α_) = 0.107 mm^–1^, 54 751 reflections measured, 17 399 unique (*R*_int_ = 0.0938) which were used in all calculations. The final w*R*(*F*_2_) was 0.2667 (all data). Crystal data for **[3]CR-C_6_** (CCDC-; 1475247): C_216_H_292_N_20_O_36_P_2_F_12_, *M* = 4034.64, monoclinic, space group *P*2_1_/*n*, *a* = 22.928(2), *b* = 25.6042(12), and *c* = 23.2131(15) Å, *α* = 90, *β* = 111.030(9), and *γ* = 90°, *V* = 12 719.6(17) Å^3^, *Z* = 2, *T* = 150(2) K, *μ*(Mo K_α_) = 0.089 mm^–1^, 52 517 reflections measured, 23 345 unique (*R*_int_ = 0.0624) which were used in all calculations. The final w*R*(*F*_2_) was 0.3386 (all data).
