# Supplementary material for: Macrocyclic shape-persistency of cyclo[6]aramide results in enhanced multipoint recognition for the highly efficient template-directed synthesis of rotaxanes
Source: Chem Sci. 2016 Nov 22;8(3):2091–100. doi: 10.1039/c6sc04714a (PMC5399641; doi:10.1039/c6sc04714a)
Supplement: Supplementary file 1 [file SC-008-C6SC04714A-s001.pdf]

Electronic Supplementary Material (ESI) for Chemical Science.
This journal is © The Royal Society of Chemistry 2016

Electronic Supplementary Information

Macrocyclic shape-persistency of cyclo[6]aramide results in enhanced multipoint recognition for highly efficient template-directed synthesis of rotaxanes

Xiaowei Li,[a] Xiangyang Yuan,[a] Pengchi Deng,[a] Lixi Chen,[a] Yi Ren,[a] Chengyuan Wang,[a] Lixin Wu,[b] Wen Feng,[a] Bing Gong,[c] Lihua Yuan*[a]

[a] *College of Chemistry, Key Laboratory for Radiation Physics and Technology of Ministry of Education, Analytical & Testing Center, Sichuan University, Chengdu 610064, Sichuan, China*
[b] *State Key Laboratory of Supramolecular Structure and Materials, Jilin University, Changchun 130012, China*
[c] *Department of Chemistry, The State University of New York at Buffalo, Buffalo, New York 14260, United States*

E-mail: lhyuan@scu.edu.cn;
Fax: +86 28 85418755

Table of Contents

1. General Methods 3

2. Synthetic Protocols 4

3. Spectroscopic Characterization 18

3.1 ^{1}H and ^{13}C NMR Spectra of Novel Compounds 18

3.2 MALDI-TOF-MS or HRESI-MS Spectra of Novel Compounds 34

4. Host-Guest Complexation of 1 and G1-G4 40

4.1 NMR Spectra of Complexation 40

4.2 2D NMR Spectra of Host-Guest Complexes 45

4.2.1 2D-NOESY Spectra of Host-Guest Complexes 45

4.2.2 2D-DOSY Spectra of Host-Guest Complexes 50

4.3 UV-vis Spectra of $1_2 \supset$ G1 and $1_2 \supset$ G4 52

4.4 Job Plots of Host-Guest Complexes 53

4.5 Determination of the Stoichiometries and Binding Constants 59

4.6 MALDI-TOF-MS Spectra of Complexes 72

4.7 FT-IR Spectra of Complexes 76

5. Optimization for Synthesis of Rotaxanes 78

6. Stacked NMR Spectra of Rotaxanes 79

6.1 2D NOESY, HSQC and HMBC Spectra of Rotaxanes 81

6.2 2D DOSY Spectra of Rotaxanes 91

7. UV-vis Spectra of Rotaxanes 95

8. Redox-Responsive of Host-Guest Complexes and Rotaxanes 95

9. X-Ray Single Crystal Structures of $3_2 \supset$ G1 and [3]CR-C_6 97

10. Molecular Modeling 104

1. General Methods

All chemicals were obtained from commercial suppliers and were used as received unless other-wise noted. All reactions were conducted with oven-dried glassware under atmosphere or nitrogen. Solvents were dried and distilled following usual protocols. Column chromatography was carried out using silica gel (300-400 mesh). Solvents for extraction and chromatography were reagent grade. $CDCl_3$ and CD_3COCD_3 were from Cambridge Isotope Laboratories (CIL).

Analytical NMR spectra were recorded on Bruker AVANCE AV II-400 MHz or Bruker AVANCE AV II-600 MHz, at a constant temperature of 298 K. Chemical shifts are reported in δ values in ppm using tetramethylsilane (TMS) or residual solvent as internal standard and coupling constants (*J*) are denoted in Hz. Multiplicities are denoted as follows: s = singlet, d = doublet, t = triplet, dd = double doublet and m = multiplet. MALDI-TOF MS spectra were recorded on a Bruker Autoflex III MS spectrometer, matrix is 2,6-dihydroxyacetophenone (DHAP). ESI mass spectra were recorded on a Bruker Daltonics MicroTOF-Q II. ESI-MS were obtained on a Thermo-ITQ. UV-vis spectra were measured by SHIMADZU UV-2450. Fourier transform Infrared (FT-IR) data were collected by a Thermal Nicolet NEXUS 670 FT-IR spectrophotometer. Single crystal X-ray data were measured on a Xcalibur E diffractometer with graphite monochromated Mo-K_α radiation (λ = 0.7107 Å). Data collection and structure refinement details can be found in the CIF files or obtained free of charge via www.ccdc.cam.ac.uk/data_request/cif.

2. Synthetic Protocols

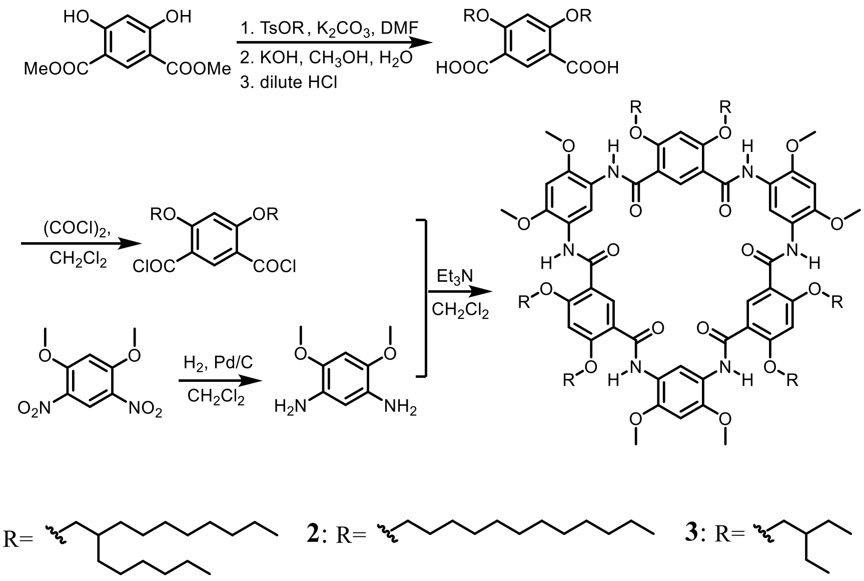

Cyclo[6]aramides **1-3** were prepared according to literature procedures.[1-3]

4,6-bis(2-ethylbutoxy)isophthalic acid was synthesized according to an analogous literature procedure.[1]

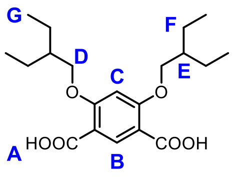

^{1}H NMR (400 MHz, $CDCl_3$): δ 10.49 (br, s, 2H, *H_A*), 8.94 (s, 1H, *H_B*), 6.58 (s, 1H, *H_C*), 4.17 (d, *J* = 5.2 Hz, 4H, *H_D*), 1.82 (m, 2H, *H_E*), 1.55 (m, 8H, *H_F*), 0.99 (m, 12H, *H_G*); ^{13}C NMR (100 MHz, $CDCl_3$, 298 K): δ 164.67, 162.89, 140.22, 111.64, 96.50, 72.45, 40.77, 23.43, 11.15; HRMS (ESI), m/z calcd for $[C_{20}H_{30}O_6+H]^+$ 367.2115; found: 367.2119; $[C_{20}H_{30}O_6+Na]^+$ 389.1935; found: 389.1936.

Synthesis of cyclo[6]aramide 3.

1,5-Dimethoxy-2,4-dinitrobenzene[1] (500 mg, 2.19 mmol) was hydrogenated in the presence of 20% Pd/C (100 mg) at 0.3 MPa for 10 h at room temperature. The solution was filtered in darkness as fast as possible followed by immediate removal of the solvent. The reduced diamine was used for the immediate coupling reaction. 4,6-Bis(2-ethylbutoxy)isophthaloyl dichloride (761 mg, 2.19 mmol) was dissolved in CH_2Cl_2 (80 mL) and added dropwise to a mixture of the above diamine and Et_3N (1.11 g, 10.95 mmol) in CH_2Cl_2 (200 mL) at 0 ℃. The solution was stirred at 0 ^{o}C under N_2 for 4 h. The organic layer was washed with water (20 mL × 3) and dried

over anhydrous Na_2SO_4 and filtered. Addition of acetone/CH_3OH to the filtrate caused a precipitation, which was filtered to give a white solid **3** (677 mg, 62%).

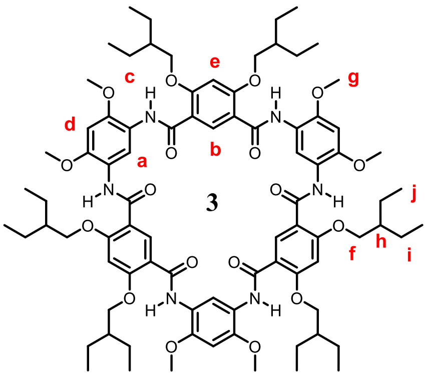

^{1}H NMR (400 MHz, $CDCl_3$, 298 K): δ 9.67 (s, 3H, H_a), 9.58 (s, 6H, H_c), 9.20 (s, 3H, H_b), 6.60 (s, 3H, H_d), 6.59 (s, 3H, H_e), 4.18 (d, J = 5.6 Hz, 12H, H_f), 3.94 (s, 18H, H_g), 2.01 (m, 6H, H_h), 1.62 (m, 24H, H_i), 1.01 (m, 36H, H_j); ^{13}C NMR (100 MHz, $CDCl_3$, 298 K): δ 162.65, 160.55, 146.07, 138.60, 120.10, 116.83, 115.20, 96.16, 94.87, 72.13, 55.93, 40.48, 23.13, 11.50; MALDI-TOF-MS, m/z calcd for $[C_{84}H_{114}N_6O_{18}+H]^+$ 1495.826; found: 1495.889.

Synthesis of heteroditopic cyclo[6]aramides 5.

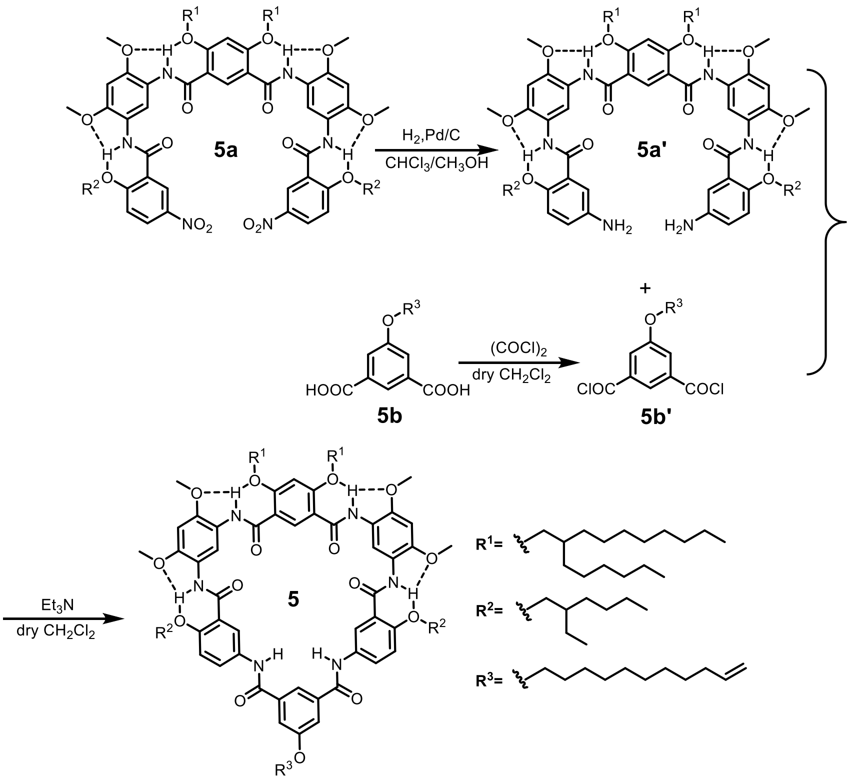

Compounds **5a**, **5b** and **5** were synthesized according to analogous literature procedures.[4] **5a** and **5b** were converted into **5a'** and **5b'** by catalytic hydrogenation, respectively. Compounds **5a'** and **5b'** were used directly in the subsequent reaction without further purification.

Pentamer **5a** (400 mg, 0.27 mmol) was hydrogenated in the presence of 20% Pd/C (80

mg) in $CHCl_3/CH_3OH$ (100 mL, v/v=5:1) for 10 h at 40 ℃. The solution was filtered in darkness as fast as possible followed by immediate removal of the solvent. The reduced diamine was used for the immediate coupling reaction. DMF (5 uL) was added to a suspension of compound **5b** (94 mg, 0.28 mmol) and oxalyl chloride (105 mg, 0.84 mmol) in CH_2Cl_2. The mixture was stirred for 40 min at room temperature. The solvent was evaporated and the resulting acid chloride was dried in vacuum at room temperature for 30 min to get compound **5b'**. Compound **5b'** was dissolved in CH_2Cl_2 (60 mL) and added dropwise to a mixture of the above **5a'** and Et_3N (162 mg, 1.60 mmol) in CH_2Cl_2 (20 mL) at 0 ℃. The solution was stirred under N_2 for 7 h. The organic layer was washed with water (20 mL × 3) and dried over anhydrous Na_2SO_4 and filtered. The crude product was purified by chromatography on silica gel (CH_2Cl_2/MeOH = 20: 1) to provide the product **5** as a light yellow solid (291 mg, 62%).

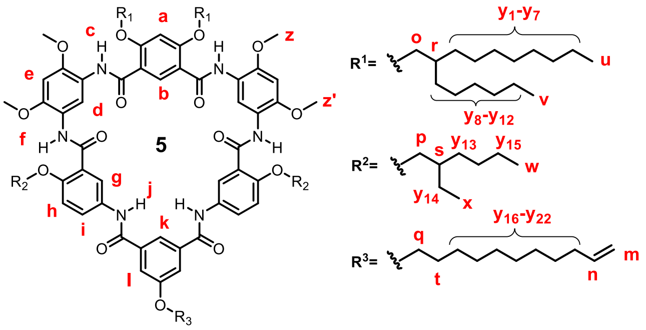

^{1}H NMR (400 MHz, $CDCl_3$, 298 K): δ = 10.20 (s, 2H, H_j), 9.42 (s, 2H, H_f), 9.35 (s, 2H, H_d), 9.16 (s, 2H, H_c), 9.15 (s, 1H, H_b), 8.49 (dd, J_1 = 8.8 Hz, J_2 = 2.4 Hz, 2H, H_i) 8.20 (s, 3H, H_k and H_g), 7.73 (s, 2H, H_l), 7.01 (d, J_1 = 8.8 Hz, 2H, H_h), 6.49 (s, 3H, H_a and H_e), 5.82 (m, 1H, H_n), 4.95 (m, 2H, H_m), 4.07 (m, 10H, H_o, H_p and H_q), 3.90 (s, 6H, H_z), 3.88 (s, 6H, $H_{z'}$), 2.04 (m, 2H, H_t), 1.82 (m, 4H, H_r and H_s), 1.54-1.25 (m, 78H, H_{y1}- H_{y22}), 0.94-0.84 (m, 24H, H_u, H_v, H_w and H_x); ^{13}C NMR (100 MHz, $CDCl_3$, 298 K): δ 165.02, 164.90, 163.08, 162.40, 161.48, 160.77, 159.87, 159.62, 159.54, 153.51, 146.64, 146.59, 145.95, 139.26, 139.21, 135.92, 135.17, 132.72, 132.40, 125.49, 124.15, 122.15, 120.95, 119.85, 118.01, 117.74, 116.11, 114.09, 113.20, 112.94, 94.87, 72.59, 72.31, 68.43, 55.89, 55.74, 38.72, 37.95, 33.83, 31.88, 31.85, 31.04, 30.05, 29.98, 29.62, 29.58, 29.47, 29.42, 29.34, 29.22, 29.16, 29.12, 28.95, 28.78, 26.70, 26.02, 25.94, 23.04, 23.08, 22.67, 14.09, 10.50. ESI-HRMS (m/z) calcd for $C_{105}H_{154}N_6O_{15}$ $[M+H]^+$ 1741.421, found $[M+H]^+$ 1741.426.

Synthesis of Guests G1-G4

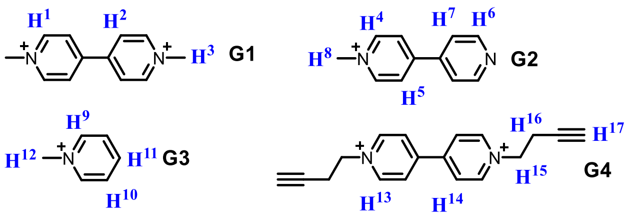

Guests **G1-G4** [5, 6] were prepared according to literature procedures.

G1 ^{1}H NMR (400 MHz, CD_3COCD_3, 298 K): δ 9.40 (d, *J* = 4.4 Hz, 4H, *H^1*), 8.85 (d, *J* = 4.4 Hz, 4H, *H^2*), 4.75 (s, 6H, *H^3*); **G2** ^{1}H NMR (400 MHz, CD_3COCD_3, 298 K): δ 9.29 (d, *J* = 6.4 Hz, 2H, *H^4*), 8.88 (d, *J* = 6.4 Hz, 2H, *H^5*), 8.70 (d, *J* = 6.4 Hz, 2H, *H^6*), 8.00 (d, *J* = 6.4 Hz, 2H, *H^7*), 4.72 (s, 3H, *H^8*); **G3** ^{1}H NMR (400 MHz, CD_3COCD_3, 298 K): δ 9.14 (d, *J* = 6.0 Hz, 2H, *H^9*), 8.75 (m, 1H, *H^{11}*), 8.29 (t, *J* = 6.8 Hz, 2H, *H^{10}*), 4.66 (s, 3H, *H^{12}*); **G4** ^{1}H NMR (400 MHz, CD_3COCD_3, 298 K): δ 9.53 (d, *J* = 4.8 Hz, 4H, *H^{13}*), 8.95 (d, *J* = 4.8 Hz, 4H, *H^{14}*), 5.16 (t, *J* = 6.0 Hz, 4H, *H^{15}*), 3.20 (m, 4H, *H^{16}*), 2.74 (t, *J* = 2.4 Hz, 2H, *H^{17}*).

Synthesis of Stopper-N_3

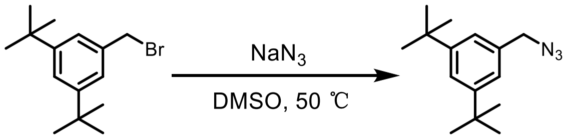

Stopper-N_3 was prepared according to the literature procedure [7].

3,5-Di-(tert-butyl)benzyl bromide (**Stopper-Br**) (500 mg, 1.77 mmol) and sodium azide (172 mg, 2.65 mmol) were mixed with DMSO (20 mL) at 50 ℃ under N_2 and the mixture was stirred for 4 h. Water (50 mL) was added to quench the reaction and the organic material was extracted with ether (3 × 50 mL), washed with brine (3 × 30 mL) and water (3 × 30 mL), dried (Na_2SO_4) and concentrated. The colorless oily residue was purified by column chromatography using silica gel (0-30% dichloromethane / petroleum ether) to give **Stopper-N_3** as colorless oil (352 mg, yield of 81%). **Stopper-N_3**

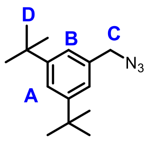

^{1}H NMR (400 MHz, $CDCl_3$, 298 K): δ 7.40 (t, *J* = 2.0 Hz, 1H, *H_A*), 7.14 (d, *J* = 2.0 Hz, 2H, *H_B*), 4.34 (s, 2H, *H_C*), 1.33 (s, 18H, *H_D*); ^{13}C NMR (100 MHz, $CDCl_3$, 298 K): δ 151.39, 134.52, 122.38, 122.31, 55.51, 34.85, 31.42; HRMS (ESI), m/z calcd for $[C_{15}H_{23}N_3\text{-}N_3]^+$ 203.1794; found: 203.1811; $[C_{15}H_{23}N_3+H]^+$ 246.1965; found: 246.2248.

Synthesis of dumbbell-shaped Axle-1

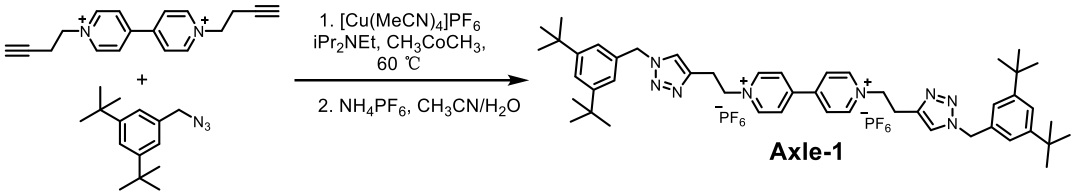

A solution of guest **G4** (40 mg, 0.072 mmol) and $Cu(MeCN)_4PF_6$ (8 mg, 0.021 mmol) in acetone (8 mL) was added under N_2 to a sealed CEM vial containing 3,5-di-(tert-butyl)benzyl azide (**Stopper-N$_3$**) (38 mg, 0.152 mmol) and N*i*Pr_2Et (11 mg, 0.086 mmol). The orange solution was stirred at 40 ℃ for 24 h. The mixture was washed with CH_2Cl_2/H_2O and dried over anhydrous Na_2SO_4, and the solvent was removed. The solid was dissolved in $CH_3CN–H_2O$ and saturated aqueous NH_4PF_6 was added. The organic solvent was then evaporated under reduced pressure. The precipitate was collected and washed with H_2O. Then the crude material was purified twice by column chromatography using silica gel (eluent: CH_3COCH_3 and then CH_3COCH_3 with 2% NH_4PF_6 (m / v)) and the main fraction was collected. Then, H_2O (200 mL) was added to the residue in order to remove excess NH_4PF_6, leaving the product as an orange precipitate. The solid was collected by filtration, further washed with excess H_2O and dried under high vacuum to afford the **Axle-1** as a dark red solid (69 mg, 92%).

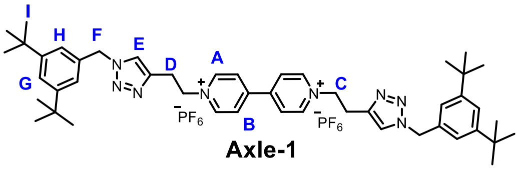

^{1}H NMR (400 MHz, CD_3COCD_3, 298 K): δ 9.45 (d, *J* = 6.4 Hz, 4H, H_A), 8.84 (d, *J* = 6.4 Hz, 4H, H_B), 7.92 (s, 2H, H_E), 7.46 (t, *J* = 2.0 Hz, 2H, H_G), 7.24 (d, *J* = 2.0 Hz, 4H, H_H) 5.56 (s, 4H, H_F), 5.30 (t, *J* = 6.4 Hz, 4H, H_C), 3.64 (m, 4H, H_D), 1.28 (s, 36H, H_I); ^{13}C NMR (100 MHz, $CDCl_3$, 298 K): δ 151.28, 150.10, 146.35, 141.92, 135.09, 126.97, 122.95, 122.41, 122.22, 61.13, 53.96, 34.53, 30.79, 26.94; HRMS (ESI), m/z calcd for $[C_{48}H_{64}N_8F_{12}P_2\text{-}2PF_6]^{2+}$ 376.2621; found: 376.2607 and $[C_{48}H_{64}N_8F_{12}P_2\text{-}2PF_6]^{+}$ 752.5248; found: 752.5246.

Synthesis of dumbbell-shaped Axle-2

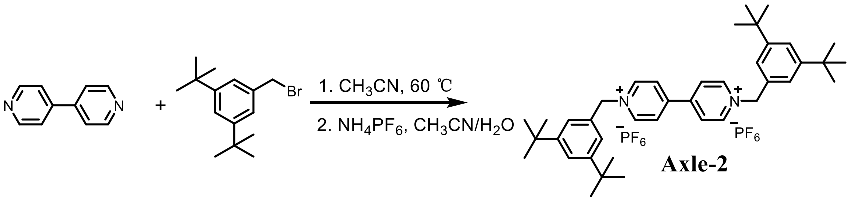

A mixture of 3,5-di-(tert-butyl)benzyl bromide (**Stopper-Br**) (350 mg, 1.24 mmol) and 4,4'-bipyridine (88 mg, 0.56 mmol) was dissolved in CH_3CN. Then the mixture was stirred under N_2 for 6 days at 60 ℃. Then the diethyl ether was added to the mixture and the precipitate was filtered off. This solid was washed with CH_2Cl_2 and then removed of the solvent of the filtrate to give a light yellow solid. The solid was dissolved in $CH_3CN–H_2O$ and saturated aqueous NH_4PF_6 was added. The organic solvent was then evaporated under reduced pressure. The precipitate was collected and washed with H_2O to yield **Axle-2** as a white solid (239 mg, 89%, over two steps).

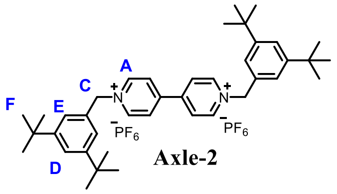

^{1}H NMR (400 MHz, CD_3COCD_3, 298 K): δ 9.45 (d, J = 6.4 Hz, 4H, H_A), 8.68 (d, J = 6.4 Hz, 4H, H_B), 7.49 (m, 6H, H_D and H_E), 6.01 (s, 4H, H_C), 1.18 (s, 36H, H_F); ^{13}C NMR (100 MHz, $CDCl_3$, 298 K): δ 153.31, 151.24, 146.61, 133.37, 128.45, 125.00, 124.71, 66.50, 35.64, 31.58; HRMS (ESI), m/z calcd for $[C_{40}H_{54}N_2F_{12}P_2\text{-}2PF_6]^+$ 562.4282; found: 562.4260; $[C_{40}H_{54}N_2F_{12}P_2\text{-}PF_6]^+$ 707.3923; found: 707.3890.

"Click-capping" approach for the synthesis of [3]rotaxanes or [2]rotaxanes[8]

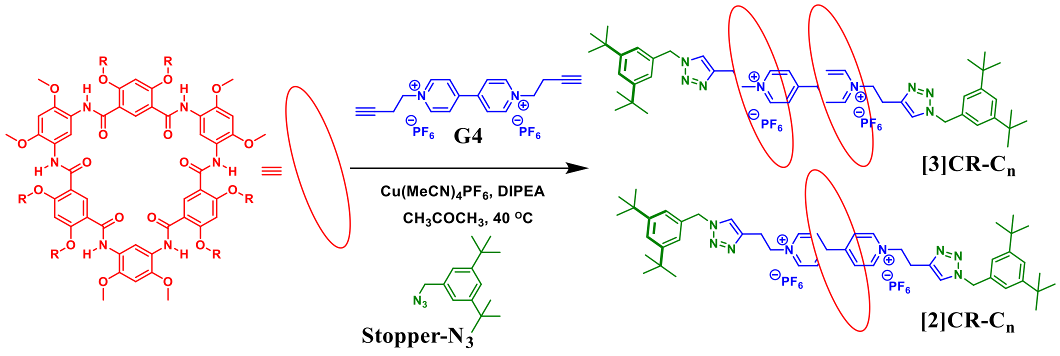

General procedure for [3]CR-C_n (n= 16, 12, 6)

Condition A (Entries 1-3, Table 1 in the main text)

A mixture of macrocycle **1-3** (2.0 equiv.), guest **G4** (1.0 equiv.) and $Cu(CH_3CN)_4PF_6$ (0.3 equiv.) was stirred in dry acetone at room temperature for 20 minutes under N_2. Then a solution of **Stopper-N$_3$** (2.5 equiv.) and N,N-diisopropylethylamine (**DIPEA**) (1.2 equiv.) was injected. The mixture was further stirred at 40 °C for 24 h. The resulting solution was washed with 16% aqueous EDTA tetra-sodium saturated ammonia solution (2 × 50 mL). The organic layer was retained and the aqueous layer

extracted twice with CH_2Cl_2 (2 × 50 mL). The organic extracts were combined and washed by water, dried over Na_2SO_4 and dried in *vacuo*. Removal of the solvent afforded a red solid and the crude material was purified by flash column chromatography using silica gel ($CHCl_3/CH_3OH$, 20:1, v/v) to give the red solid **[3]CR-C$_n$** or **[2]CR-C$_n$**.

[3]CR-C$_{16}$ was synthesized according to the above general procedure using macrocycle **1** (100.0 mg, 0.043 mmol), guest **G4** (12 mg, 0.021 mmol), $Cu(CH_3CN)_4PF_6$ (3 mg, 0.006 mmol) **Stopper-N$_3$** (13 mg, 0.053 mmol) and N,N-diisopropylethylamine (**DIPEA**) (3 mg, 0.024 mmol) in dry acetone. Flash column chromatography using silica gel ($CHCl_3/CH_3OH$, 20:1) afforded 103 mg (86% yield) of [3]rotaxane **[3]R-C$_{16}$** as a red solid.

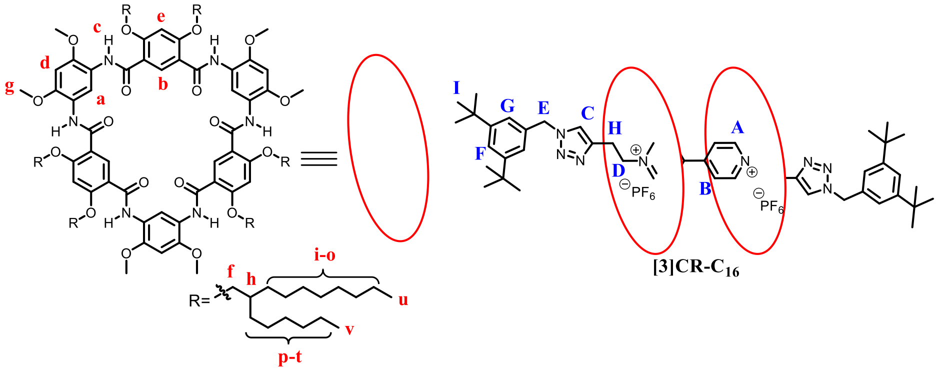

^{1}H NMR (400 MHz, CD_3COCD_3, 298 K): δ 10.08 (s, 6H, H_a), 9.80 (s, 12H, H_c), 9.59 (d, J = 6.4 Hz, 4H, H_A), 8.95 (d, J = 6.4 Hz, 4H, H_B), 8.64 (s, 6H, H_b), 7.46 (s, 2H, H_C), 7.08 (m, 8H, H_d and H_F), 6.77 (d, J = 1.6 Hz, 4H, H_G), 6.67 (m, 6H, H_e), 5.12 (m, 4H, H_D), 5.10 (s, 4H, H_E), 4.73 (m, 24H, H_f), 4.00 (s, 36H, H_g), 3.37 (m, 4H, H_H), 2.20 (m, 12H, H_h), 1.62-1.21 (m, 324H, H_I, H_{i-o} and H_{p-t}), 0.92-0.81 (m, 72H, H_u and H_v); ^{13}C NMR (100 MHz, CD_3COCD_3, 298 K): δ 162.54, 161.46, 151.41, 150.74, 146.88, 146.18, 145.03, 141.59, 139.08, 136.07, 128.95, 123.38, 123.20, 122.30, 120.94, 116.31, 115.62, 98.25, 95.00, 73.71, 71.57, 71.43, 63.27, 56.16, 54.17, 38.36, 35.01, 32.75, 32.71, 32.62, 31.55, 31.43, 31.24, 31.17, 30.93, 30.81, 30.63, 30.48, 30.35, 30.40, 30.07, 27.48, 26.51, 23.52, 23.41, 23.33, 14.51, 14.45, 14.40; MALDI-TOF-MS, m/z calcd for $[C_{336}H_{532}N_{20}O_{36}F_{12}P_2\text{-}2PF_6]^{\cdot+}$ 5427.051; found: 5427.495.

[3]CR-C$_{12}$ was synthesized according to the above general procedure using macrocycle **2** (201 mg, 0.100 mmol), guest **G4** (26 mg, 0.048 mmol),

$Cu(CH_3CN)_4PF_6$ (5 mg, 0.014 mmol) **Stopper-N_3** (30 mg, 0.119 mmol) and **DIPEA** (7 mg, 0.057 mmol) in dry acetone. Flash column chromatography using silica gel ($CHCl_3$ / CH_3OH, 20 : 1) afforded 220 mg (91% yield) of [3]rotaxane **[3]R-C_{12}** as a red solid.

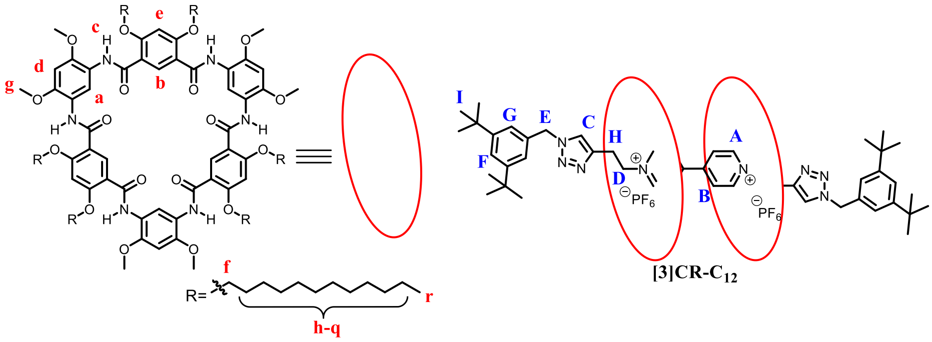

^{1}H NMR (400 MHz, CD_3COCD_3, 298 K): δ 10.17 (s, 6H, H_a), 9.90 (s, br, 4H, H_A), 9.57 (br, 4H, H_B), 9.43 (s, 12H, H_c), 8.60 (s, 6H, H_b), 7.14 (s, 2H, H_C), 6.70 (s, 4H, H_G), 6.60 (m, br, 2H, H_F), 6.54 (m, br, 12H, H_d and H_e), 5.22 (m, br, 4H, H_D), 5.03 (m, br, 4H, H_E), 4.18-4.07 (m, 24H, H_f), 3.85 (s, 36H, H_g), 3.53 (partial overlayer, m, br, 4H, H_H), 1.33-1.20 (m, 276H, H_I and H_{h-q}), 1.00-0.83 (m, 36H, H_r); ^{13}C NMR (100 MHz, CD_3COCD_3, 298 K): δ 162.42, 160.90, 151.52, 146.67, 145.60, 142.24, 139.18, 138.65, 136.02, 130.44, 123.91, 122.79, 122.46, 121.31, 116.03, 115.49, 96.87, 94.44, 83.11, 70.66, 55.87, 35.06, 32.72, 31.55, 30.90, 30.69, 30.56, 30.47, 26.60, 23.38, 14.44; MALDI-TOF-MS, m/z calcd for $[C_{288}H_{436}N_{20}O_{36}F_{12}P_2\text{-}2PF_6]^{\cdot+}$ 4753.296; found: 4753.335.

[3]CR-C_6 was synthesized according to the above general procedure using macrocycle **3** (200 mg, 0.133 mmol), guest **G4** (35 mg, 0.064 mmol), $Cu(CH_3CN)_4PF_6$ (7 mg, 0.019 mmol) **Stopper-N_3** (39 mg, 0.159 mmol) and **DIPEA** (10 mg, 0.076 mmol) in dry acetone. The undissolved macrocycle **3** (65 mg) was collected by filter. The percent conversion of macrocycle **3** achieved 68%. Flash column chromatography using silica gel ($CHCl_3$ / CH_3OH, 20 : 1) afforded 166 mg (64% yield) of [3]rotaxane **[3]CR-C_6** as a red solid.

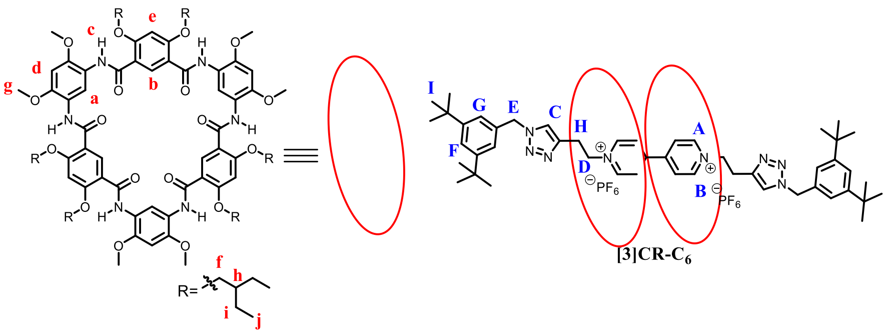

^{1}H NMR (400 MHz, CD_3COCD_3, 298 K): δ 10.10 (s, 6H, H_a), 9.73 (s, 12H, H_c), 9.62 (d, J = 6.4 Hz, 4H, H_A), 9.06 (d, J = 6.4 Hz, 4H, H_B), 8.60 (s, 6H, H_b), 7.59 (s, 2H, H_C), 7.09 (m, 8H, H_d and H_F), 6.75 (d, J = 1.6 Hz, 4H, H_G), 6.67 (s, 6H, H_e), 5.12-5.10 (m, 4H, H_D), 5.10 (s, 4H, H_E), 4.40-4.36 (m, 12H, H_f), 4.31-4.27 (m, 12H, H_f), 3.99 (s, 36H, H_g), 4.36 (m, 4H, H_H), 2.10 (m, 12H, H_h), 1.73-1.38 (m, 48H, H_i and H_I), 0.99 (m, 36H, H_i), 0.91 (m, 72H, H_j); ^{13}C NMR (100 MHz, CD_3COCD_3, 298 K): δ 161.73, 160.62, 150.63, 150.46, 145.97, 145.31, 138.05, 135.23, 128.48, 122.65, 122.58, 122.30, 121.43, 120.07, 115.45, 114.74, 97.36, 94.21, 72.00, 62.11, 55.25, 53.13, 39.75, 34.12, 30.50, 27.47, 22.31, 22.08, 10.44, 9.20; MALDI-TOF-MS, m/z calcd for $[C_{216}H_{292}N_{20}O_{36}F_{12}P_2\text{-}2PF_6]^{\cdot+}$ 3744.178; found: 3744.265.

Condition B (Entry 4, Table 1 in the main text)

A mixture of macrocycle **1** (100 mg, 0.043 mmol), guest **G4** (24 mg, 0.043 mmol) and $Cu(CH_3CN)_4PF_6$ (5 mg, 0.013 mmol) was stirred in dry acetone at room temperature for 20 minutes under N_2. Then a solution of **Stopper-N$_3$** (26 mg, 0.107 mmol) and N,N-diisopropylethylamine (**DIPEA**) (7 mg, 0.051 mmol) was injected. The mixture was further stirred at 40 ^{o}C for 24 h. The resulting solution was washed with 16% aqueous EDTA tetra-sodium saturated ammonia solution (2 × 50 mL). The organic layer was retained and the aqueous layer extracted twice with CH_2Cl_2 (2 × 50 mL). The organic extracts were combined and washed by water, dried over Na_2SO_4 and dried in *vacuo*. Removal of the solvent afforded a red solid and the crude material was purified by flash column chromatography using silica gel ($CHCl_3/CH_3OH$, 20:1, v/v) to give the red solid **[3]CR-C$_{16}$** 44 mg (36% yield) and **[2]CR-C$_{16}$** 49 mg (34% yield). The yield of [2]/[3]rotaxane was calculated based on the macrocycle **1**. **[3]CR-C$_{16}$/[2]CR-C$_{16}$** = 53/100.

Condition C (Entry 5, Table 1 in the main text)

A mixture of macrocycle **1** (100 mg, 0.043 mmol), guest **G4** (12 mg, 0.021 mmol) and $Cu(CH_3CN)_4PF_6$ (3 mg, 0.006 mmol) was stirred in dry CH_3COCH_3 / CH_3CN = 1 : 1 (v/v) at room temperature for 20 minutes under N_2. Then a solution of **Stopper-N$_3$** (13 mg, 0.053 mmol) and N,N-diisopropylethylamine (**DIPEA**) (3 mg, 0.024 mmol) was injected. The mixture was further stirred at 40 ^oC for 24 h. The resulting solution was washed with 16% aqueous EDTA tetra-sodium saturated ammonia solution (2 × 50 mL). The organic layer was retained and the aqueous layer extracted twice with CH_2Cl_2 (2 × 50 mL). The organic extracts were combined and washed by water, dried over Na_2SO_4 and dried in *vacuo*. Removal of solvents afforded a red solid and the crude material was purified by flash column chromatography using silica gel ($CHCl_3/CH_3OH$, 20:1, v/v) to give the red solid **[3]CR-C$_{16}$** 72 mg (60% yield) and **[2]CR-C$_{16}$** 22 mg (18% yield). The yield of [2]/[3]rotaxane based on the guest **G4**. **[3]CR-C$_{16}$/[2]CR-C$_{16}$** = 193/100.

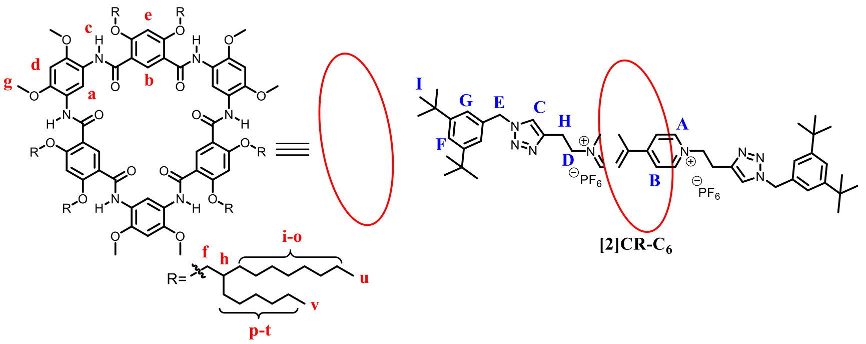

[2]CR-C$_{16}$, red solid. ^1H NMR (400 MHz, CD_3COCD_3, 298 K): δ 9.97 (s, 3H, H_a), 9.77 (s, 6H, H_c), 9.55 (d, J = 6.4 Hz, 4H, H_A), 9.23 (d, J = 6.4 Hz, 4H, H_B), 8.95 (s, 3H, H_b), 7.73 (s, 2H, H_C), 7.33 (s, 2H, H_F), 7.13 (s, 4H, H_G), 6.99 (m, 6H, H_d and H_e), 5.32 (m, 4H, H_D), 5.25 (s, 4H, H_E), 4.43-4.42 (m, 12H, H_f), 4.08 (s, 18H, H_g), 3.52 (m, 4H, H_H), 2.21 (m, 6H, H_h), 1.60-1.16 (m, 180H, H_I, $H_{i\text{-}o}$ and $H_{p\text{-}t}$), 0.88-0.82 (m, 36H, H_u and H_v); ^{13}C NMR (100 MHz, CD_3COCD_3, 298 K): δ 163.04, 161.74, 152.00, 147.01, 146.87, 140.19, 135.88, 135.36, 123.56, 123.07, 121.02, 115.77, 98.55, 96.14, 73.99, 73.15, 69.27, 65.56, 62.01, 56.99, 54.61, 54.30, 51.68, 48.36, 38.58, 35.28, 32.73, 32.64, 32.58, 31.85, 31.83, 31.60, 30.83, 30.53, 30.48, 27.23, 23.34, 14.41; MALDI-TOF-MS, m/z calcd for $[C_{192}H_{298}N_{14}O_{18}F_{12}P_2\text{-}2PF_6]^{\cdot+}$ 3090.299; found: 3090.275.

“Facile one-pot” approach for the synthesis of [3]rotaxanes and [2]rotaxanes[9]

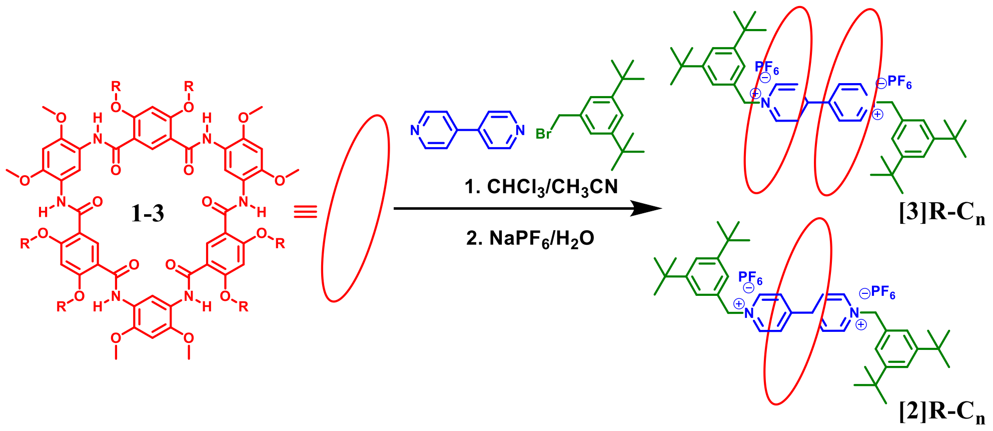

General procedure for [3]R-C_n or **[2]R-C_n**

A mixture of cyclo[6]aroamide **1-3** (2 equiv.), 3,5-di-*tert*-butylbenzyl bromide **Stopper-Br** (2.5 equiv.) and 4,4’-bipyridine (1 equiv.) was stirred in 6 mL $CHCl_3/CH_3CN$ (1/1, v/v) () under N_2 at 40 ^{o}C for 48 h. Removal of solvents afforded a pale red solid and the crude compound was dissolved in acetone/H_2O and saturated aqueous $NaPF_6$ was added; the organic solvent was then evaporated under reduced pressure. The precipitate was collected and washed with H_2O. Then the crude material was purified by flash column chromatography using silica gel ($CHCl_3/CH_3OH$, 30:1, and then $CHCl_3/CH_3OH$, 10:1, v/v) to give the red solid **[3]R-C_n** or **[2]R-C_n**.

[3]R-C_{16} was synthesized according to the above general procedure using macrocycle **1** (210 mg, 89.84 μmol), **Stopper-Br** (30 mg, 106.95 μmol) and 4,4’-bipyridine (6.7 mg, 42.78 μmol) in $CHCl_3$ / CH_3CN = 1 / 1 (v/v) (6 mL). Flash column chromatography using silica gel ($CHCl_3$ / CH_3OH, 30 : 1, v/v and then $CHCl_3/CH_3OH$, 10:1, v/v) afforded 191 mg (85% yield, over two steps) of [3]rotaxane **[3]R-C_{16}** as a red solid.

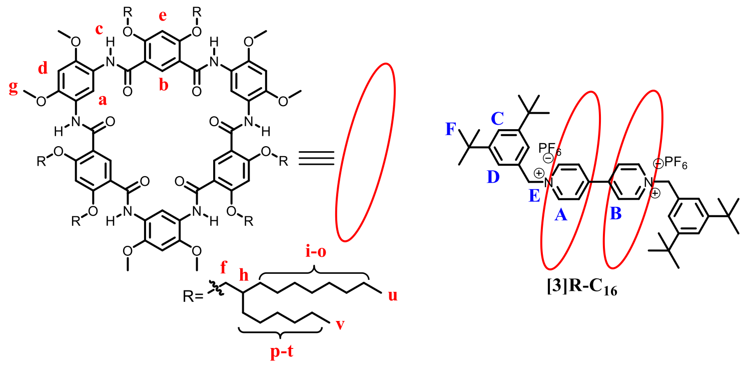

^{1}H NMR (400 MHz, CD_3COCD_3, 298 K): δ 10.07 (s, br, 6H, H_a), 9.98 (d, J = 6.4 Hz,

4H, H_A), 9.73 (s, 12H, H_c), 8.95 (d, J = 6.4 Hz, 4H, H_B), 8.77 (m, 6H, H_b), 7.31 (d, J = 1.6 Hz, 4H, H_D), 7.16 (t, J = 1.6 Hz, 2H, H_C), 7.09 (s, 6H, H_d), 6.64 (m, 6H, H_e), 6.02 (s, 4H, H_E), 4.39-4.33 (m, 24H, H_f), 3.98 (m, 36H, H_g), 2.16 (m, 12H, H_h), 1.63-1.21 (m, 324H, H_I, $H_{i\text{-}o}$ and $H_{p\text{-}t}$), 0.90-0.79 (m, 72H, H_u and H_v); ^{13}C NMR (100 MHz, CD_3COCD_3, 298 K): δ 160.85, 159.98, 150.83, 149.75, 145.15, 144.60, 137.98, 132.36, 132.00, 130.56, 128.20, 128.13, 127.45, 122.79, 122.51, 119.64, 114.99, 114.39, 96.85, 93.45, 72.33, 64.47, 54.70, 36.95, 33.80, 31.32, 31.28, 31.20, 30.21, 29.97, 29.69, 29.51, 29.34, 29.21, 29.02, 28.93, 28.67, 26.15, 25.13, 22.09, 21.98, 21.92, 18.45, 13.08, 13.04, 12.99, 12.59; MALDI-TOF-MS, m/z calcd for $[C_{328}H_{522}N_{14}O_{36}F_{12}P_2\text{-}2PF_6]^{\cdot+}$ 5236.954; found: 5236.883.

[3]R-C$_{12}$ was synthesized according to the above general procedure using macrocycle **2** (200 mg, 99.96 μmol), **Stopper-Br** (34 mg, 119.00 μmol) and 4,4'-bipyridine (7.4 mg, 47.60 μmol) in $CHCl_3/CH_3CN$ = 1/1 (v/v) (6 mL). Flash column chromatography using silica gel ($CHCl_3/CH_3OH$, 30:1, v/v and then $CHCl_3/CH_3OH$, 10:1, v/v) afforded 196 mg (85% yield, over two steps) of [3]rotaxane **[3]R-C$_{12}$** as a red solid.

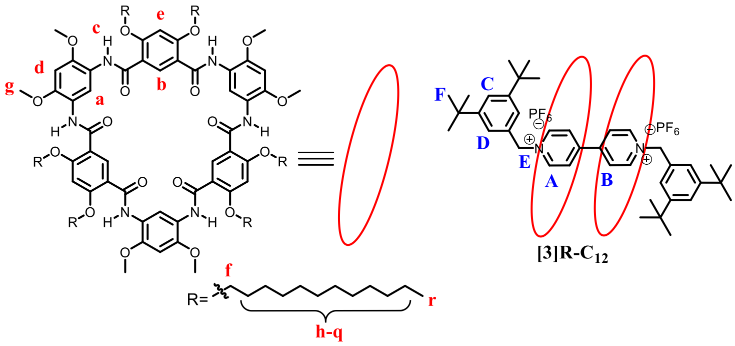

^{1}H NMR (400 MHz, CD_3COCD_3, 298 K): δ 10.02 (s, br, 6H, H_a), 9.96 (d, br, J = 6.4 Hz, 4H, H_A), 9.73 (d, br, J = 6.4 Hz, 4H, H_B), 9.30 (s, 12H, H_c), 8.56 (s, 6H, H_b), 7.37 (m, 4H, H_D), 7.10 (s, 2H, H_C), 6.49 (s, 6H, H_d), 6.39 (s, br, 6H, H_e), 6.04 (s, br, 4H, H_E), 4.03-3.96 (m, 24H, H_f), 3.83 (s, 36H, H_g), 1.92 (m, br, 24H, H_h), 1.32-1.21 (m, 252H, H_F and $H_{i\text{-}q}$), 0.85 (m, 36H, H_r); ^{13}C NMR (100 MHz, CD_3COCD_3, 298 K): δ 161.23, 159.72, 151.43, 151.15, 145.27, 144.44, 138.50, 133.04, 130.32, 123.69, 120.61, 114.97, 114.75, 95.66, 93.41, 69.54, 54.87, 34.44, 34.25, 31.88, 31.16, 30.94, 30.65, 30.07, 29.86, 29.71, 29.39, 25.77, 22.55, 13.60; MALDI-TOF-MS, m/z calcd for $[C_{280}H_{426}N_{14}O_{36}F_{12}P_2\text{-}2PF_6]^{\cdot+}$ 4563.199; found: 4563.225.

[2]R-C_6 was synthesized according to the above general procedure using macrocycle **3** (200 mg, 133.70 μmol), **Stopper-Br** (45 mg, 159.17 μmol) and 4,4'-bipyridine (9.9 mg, 63.67 μmol) in $CHCl_3$ / CH_3CN = 1 / 1 (v / v) (6 mL). The undissolved macrocycle **3** (33 mg) was collected by filter. The percent conversion of macrocycle **3** achieved 84%. Flash column chromatography using silica gel ($CHCl_3$ / CH_3OH, 30 : 1, v / v and then $CHCl_3$ / CH_3OH, 10 : 1, v / v) afforded 106 mg (71% yield) of [2]rotaxane **[2]R-C_6** as a pale orange solid.

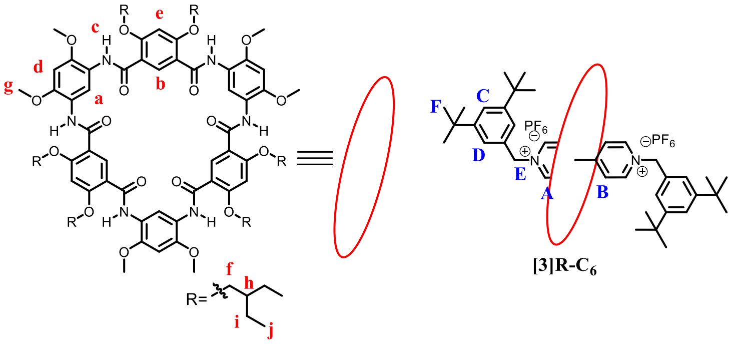

[3]R-C_6

^{1}H NMR (400 MHz, CD_3COCD_3, 298 K): δ 9.91 (s, 3H, H_a), 9.72 (d, overlap, 4H, H_A), 9.70 (s, 6H, H_c), 9.31 (d, J = 6.4 Hz, 4H, H_B), 8.98 (s, 3H, H_b), 7.41 (s, br, 4H, H_D), 7.38 (s, 2H, H_C), 7.12 (s, 3H, H_d), 6.97 (s, 3H, H_e), 6.23 (s, 4H, H_E), 4.40 (d, J = 6.0 Hz, 12H, H_f), 4.07 (s, 18H, H_g), 3.13 (m, br, 6H, H_h), 1.64 (m, 24H, H_i), 1.06 (s, 36H, H_F), 1.04-0.99 (m, 36H, H_j); ^{13}C NMR (100 MHz, CD_3COCD_3, 298 K): δ 162.13, 160.09, 151.89, 150.84, 145.91, 145.71, 138.07, 132.65, 128.58, 123.53, 120.04, 116.31, 114.80, 97.95, 95.22, 72.26, 65.40, 55.71, 40.30, 34.44, 30.51, 29.75, 22.89, 10.23; MALDI-TOF-MS, m/z calcd for $[C_{124}H_{168}N_8O_{18}F_{12}P_2\text{-}2PF_6]^{+}$ 2058.255; found: 2058.365.

References

[1] Yuan, L.; Feng, W.; Yamato, K.; Sanford, A. R.; Xu, D.; Guo, H.; Gong, B., *J. Am. Chem. Soc.* **2004**, *126*, 11120.

[2] Yang, Y.; Feng, W.; Hu, J.; Zou, S.; Gao, R.; Yamato, K.; Kline, M.; Cai, Z.; Gao, Y.; Wang, Y.; Li, Y.; Yang, Y.; Yuan, L.; Zeng, X. C.; Gong, B., *J. Am. Chem. Soc.* **2011**, *133*, 18590.

[3] Hu, J.; Chen, L.; Ren, Y.; Deng, P.; Li, X.; Wang, Y.; Jia, Y.; Luo, J.; Yang, X.; Feng, W.; Yuan, L., *Org. Lett.* **2013**, *15*, 4670.

[4] Hu, J.; Chen, L.; Shen, J.; Luo, J.; Deng, P.; Ren, Y.; Zeng, H.; Feng, W.; Yuan, L. *Chem. Commun.* **2014**, *50*, 8024.

[5] Xiao, Y.; Chu, L.; Sanakis, Y.; Liu, P., *J. Am. Chem. Soc.* **2009**, *131*, 9931.

[6] Coskun, A.; Saha, S.; Aprahamian, I.; Stoddart, J. F., *Org. Lett.* **2008**, *10*, 3187.

[7] Gassensmith, J. J.; Barr, L.; Baumes, J. M.; Paek, A.; Nguyen, A.; Smith, B. D., *Org. Lett.* **2008**, *10*, 3343.
[8] (a) Neal, E. A.; Goldup, S., *Chem. Sci*. **2015**, *6*, 2398; (b) Winn, J.; Pinczewska, A.; Goldup, S., *J. Am. Chem. Soc*. **2013**, *135*, 13318.
[9] (a) Xu, Z.; Jiang, L.; Feng, Y.; Zhang, S.; Liang, J.; Pan, S.; Yang, Y.; Yang, D.; Cai, Y., *Org. Biomol. Chem.* **2011**, *9*, 1237; (b) Cheng, P.-N.; Lin, C.-F.; Liu, Y.-H.; Lai, C.-C.; Peng, S.-M.; Chiu, S.-H., *Org. Lett*. **2006**, *8*, 435.

3. Spectroscopic Characterization

3.1 ^{1}H and ^{13}C NMR Spectra of Novel Compounds

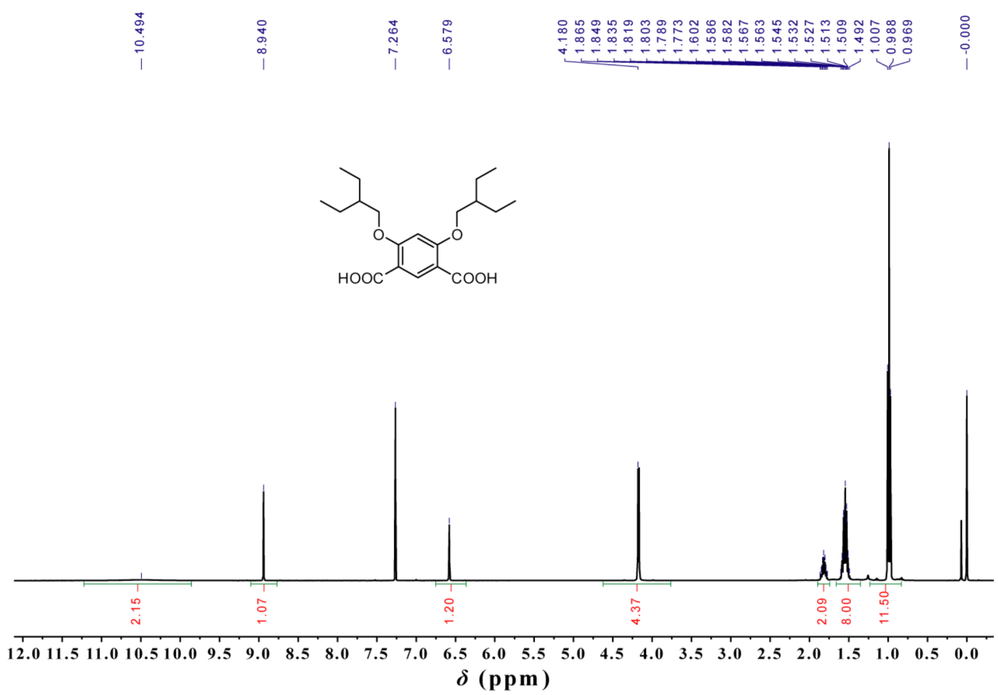

Figure S1 ^{1}H NMR spectrum of **4,6-bis(2-ethylbutoxy)isophthalic acid** (400 MHz, $CDCl_3$, 298 K).

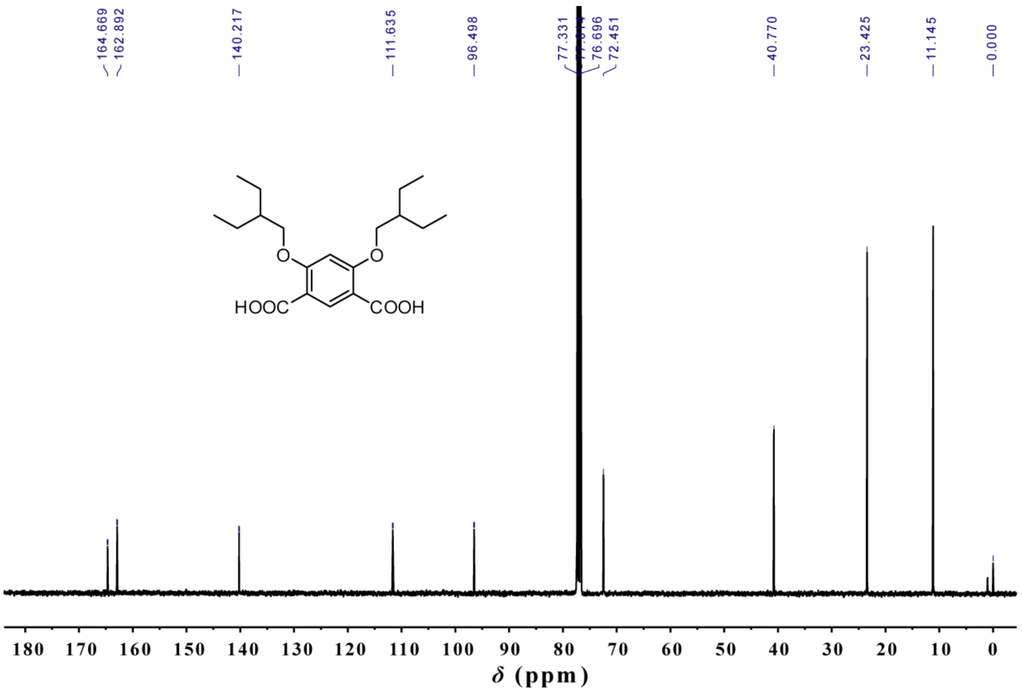

Figure S2 ^{13}C NMR spectrum of **4,6-bis(2-ethylbutoxy)isophthalic acid** (100 MHz, $CDCl_3$, 298 K).

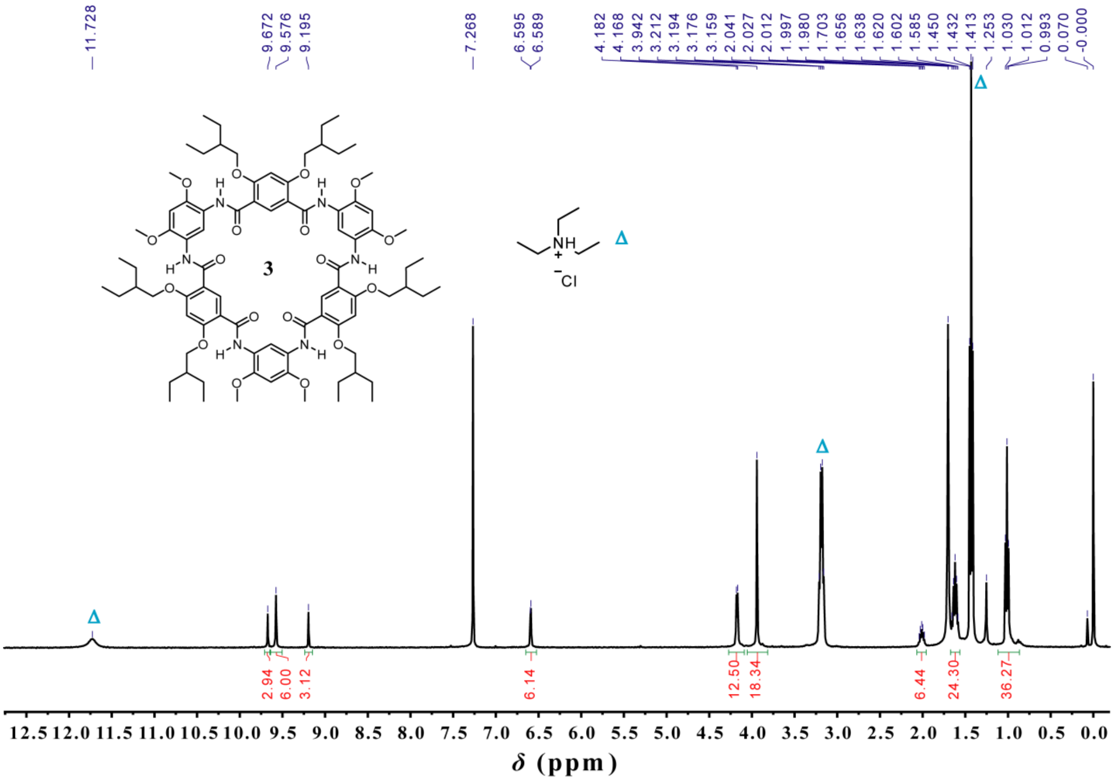

Figure S3 ^{1}H NMR spectrum of cyclo[6]aramide **3** and additional **Et$_3$N·HCl** (Δ) (400 MHz, $CDCl_3$, 298 K).

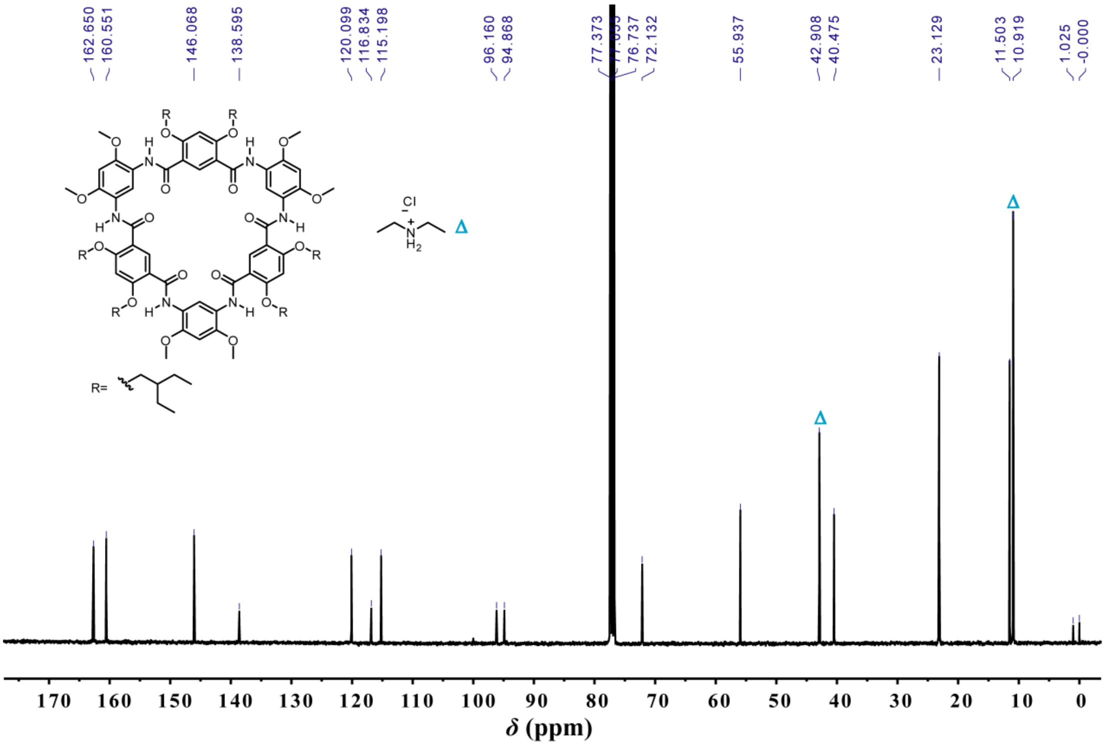

Figure S4 ^{13}C NMR spectrum of cyclo[6]aramide **3** and additional **Et$_2$NH·HCl** (Δ) (100 MHz, $CDCl_3$, 298 K).

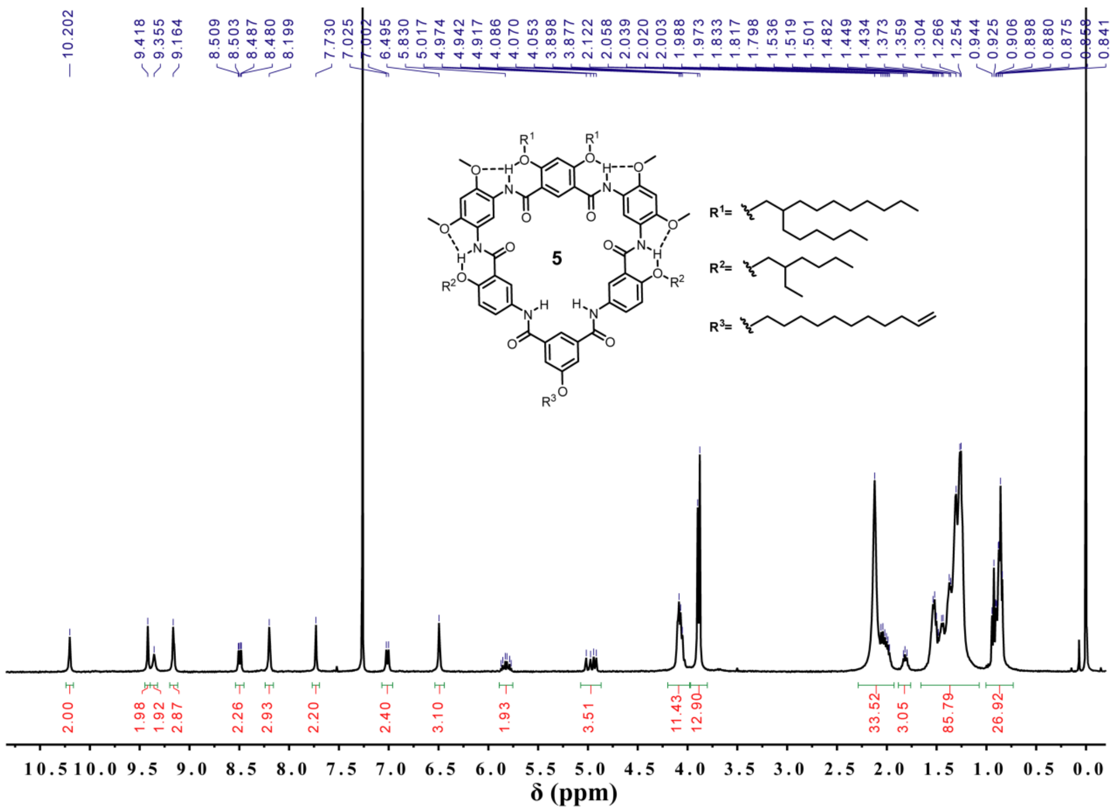

Figure S5 ^{1}H NMR spectrum of heteroditopic cyclo[6]aramides **5** (400 MHz, $CDCl_3$, 298 K).

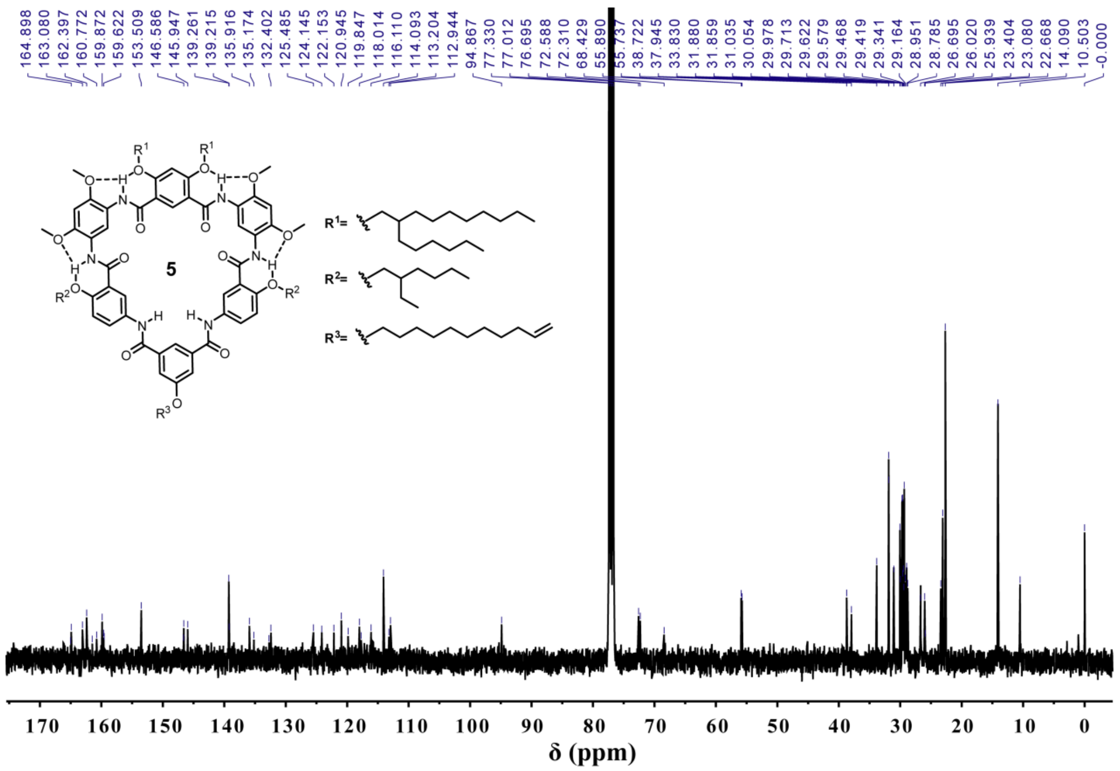

Figure S6 ^{13}C NMR spectrum of heteroditopic cyclo[6]aramides **5** (100 MHz, $CDCl_3$, 298 K).

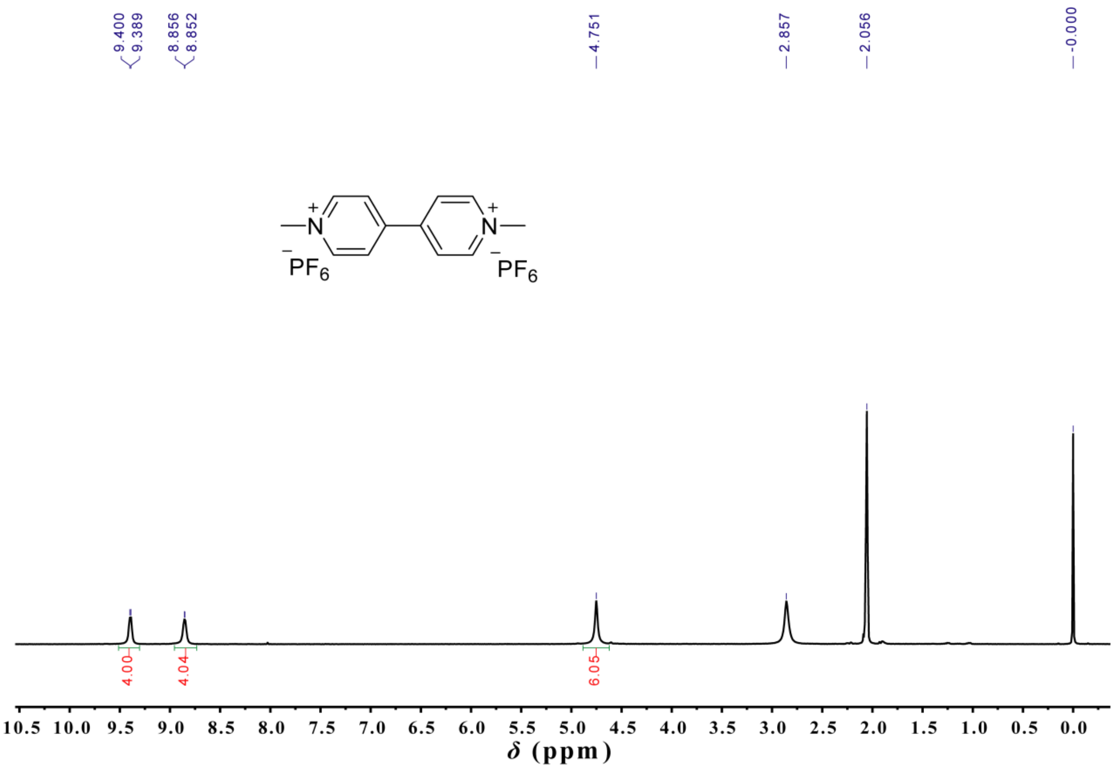

Figure S7 ^{1}H NMR spectrum of **G1** (400 MHz, CD_3COCD_3, 298 K).

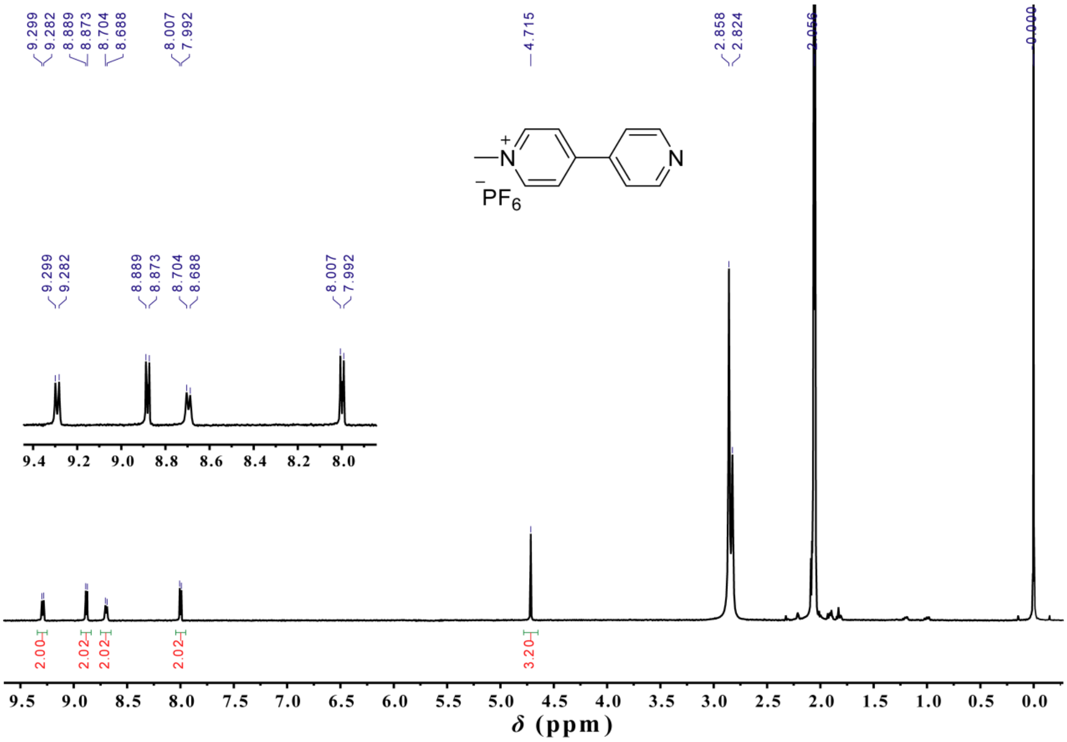

Figure S8 ^{1}H NMR spectrum of **G2** (400 MHz, CD_3COCD_3, 298 K).

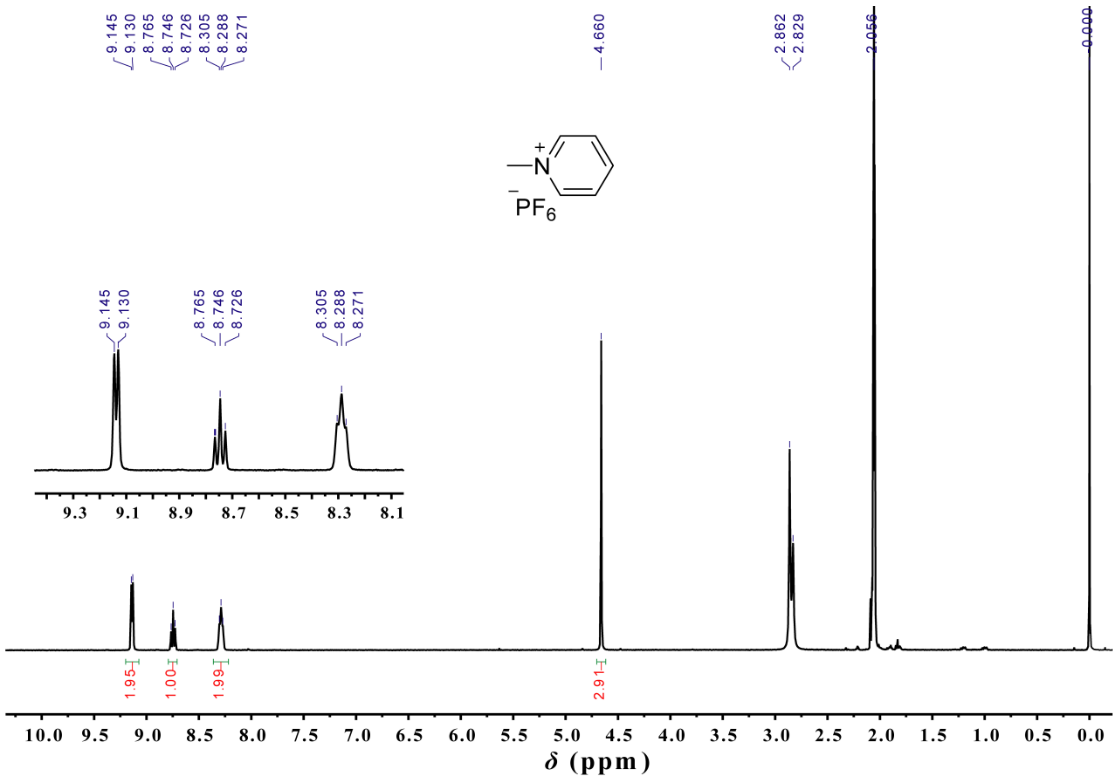

Figure S9 ^{1}H NMR spectrum of **G3** (400 MHz, CD_3COCD_3, 298 K).

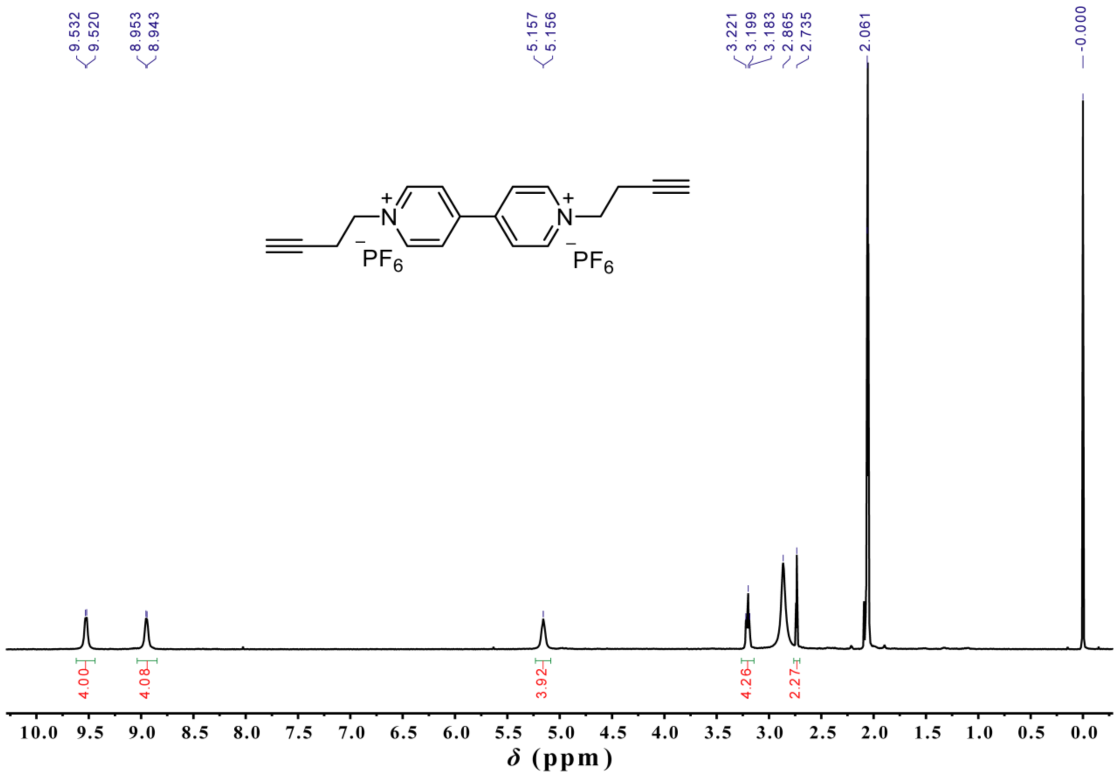

Figure S10 ^{1}H NMR spectrum of **G4** (400 MHz, CD_3COCD_3, 298 K).

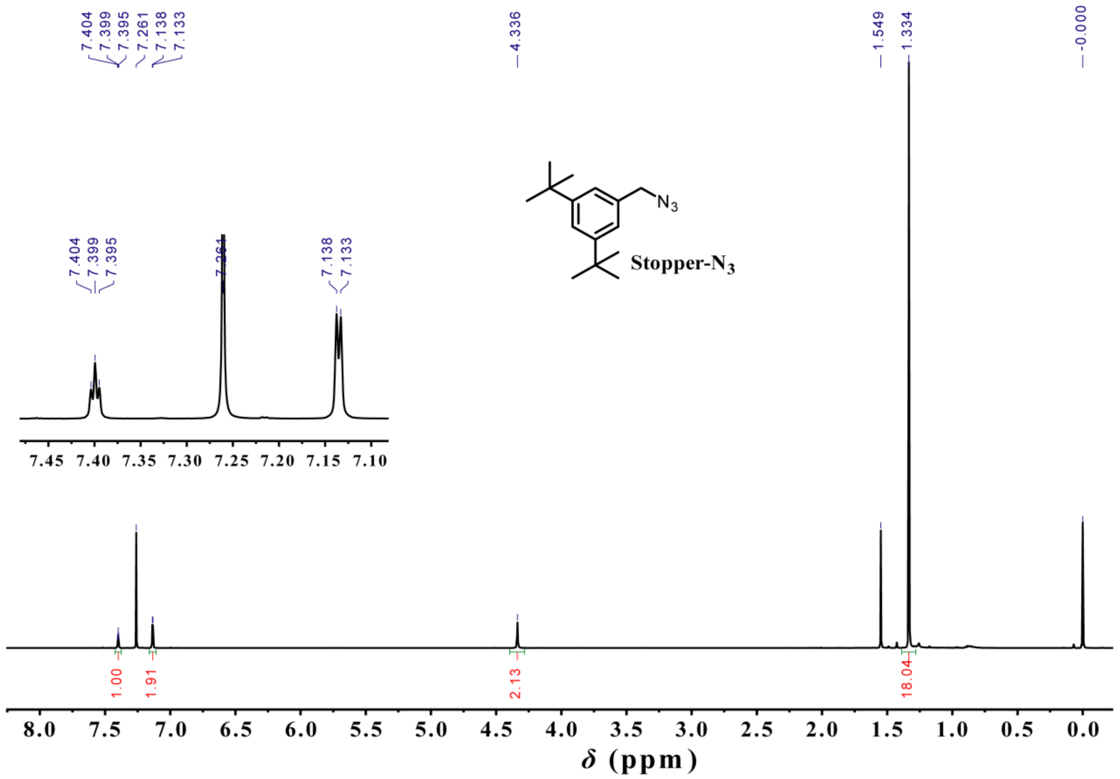

Figure S11 ^{1}H NMR spectrum of **Stopper-N$_3$** (400 MHz, $CDCl_3$, 298 K).

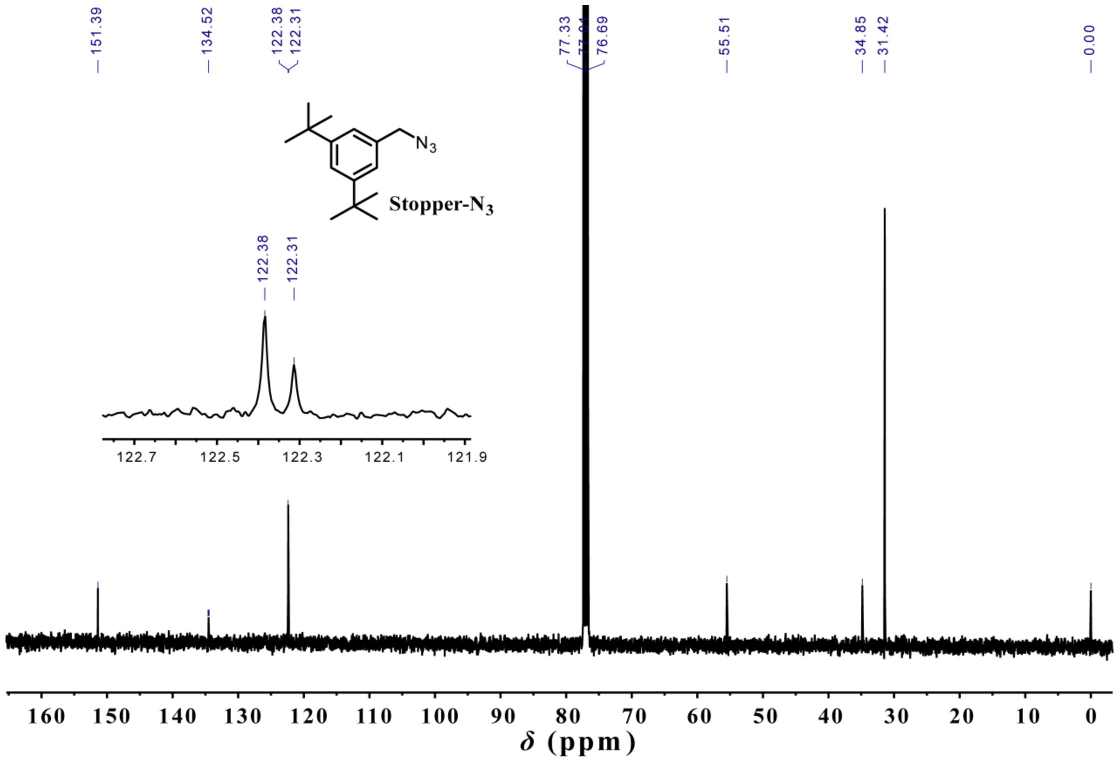

Figure S12 ^{13}C NMR spectrum of **Stopper-N$_3$** (100 MHz, $CDCl_3$, 298 K).

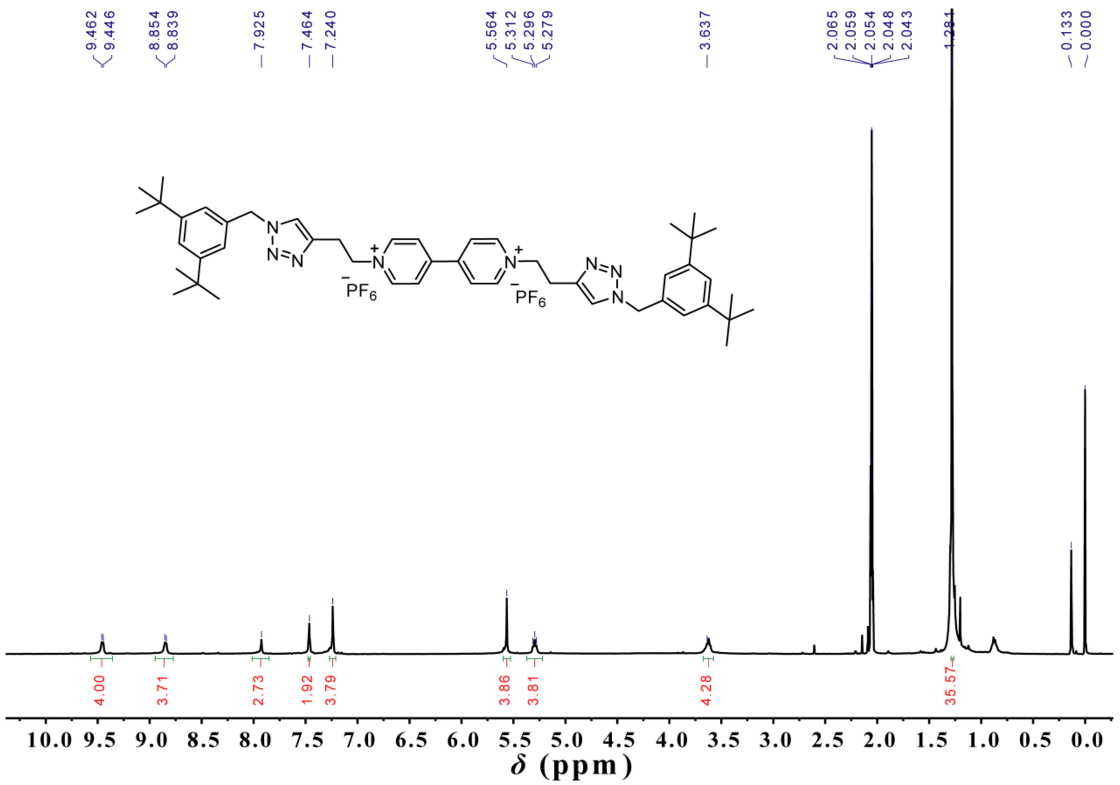

Figure S13 ^{1}H NMR spectrum of **Axle-1** (400 MHz, CD_3COCD_3, 298 K).

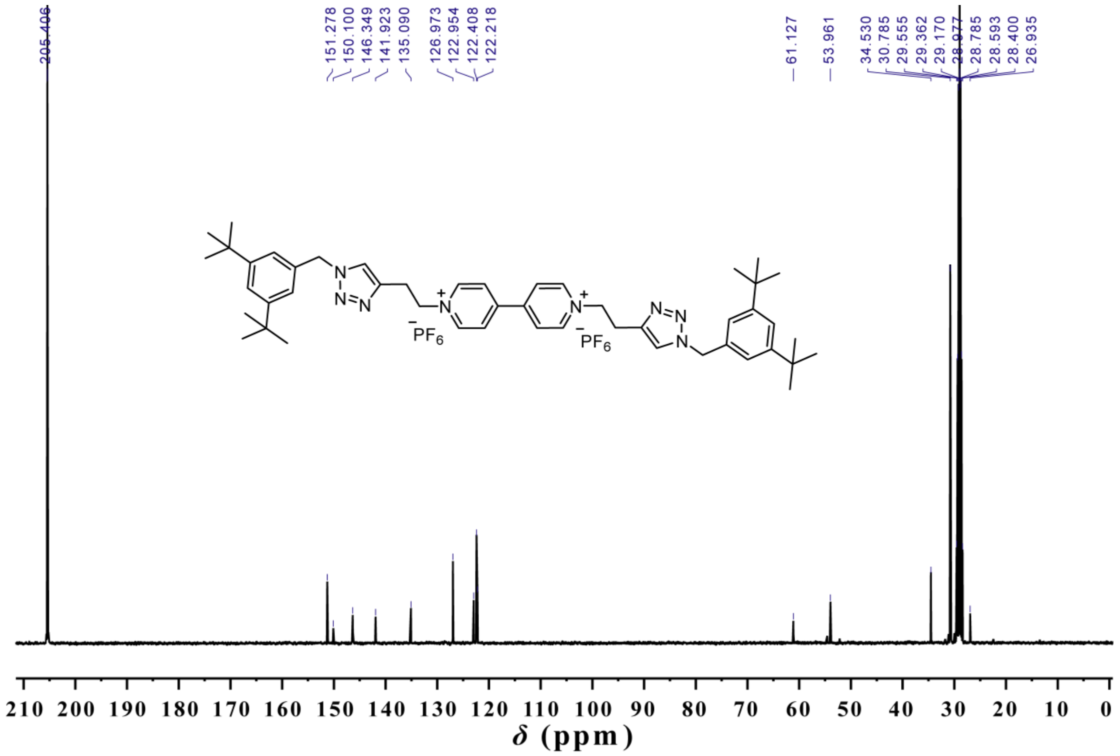

Figure S14 ^{13}C NMR spectrum of **Axle-1** (100 MHz, CD_3COCD_3, 298 K).

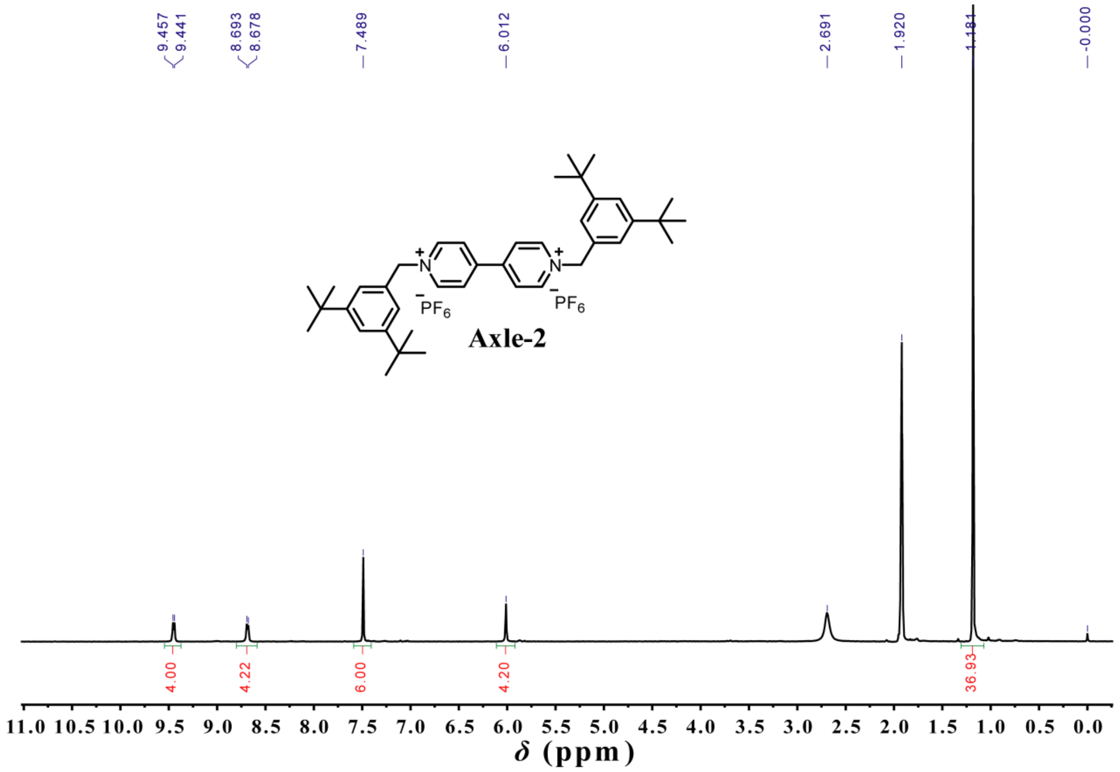

Figure S15 ^{1}H NMR spectrum of **Axle-2** (400 MHz, CD_3COCD_3, 298 K).

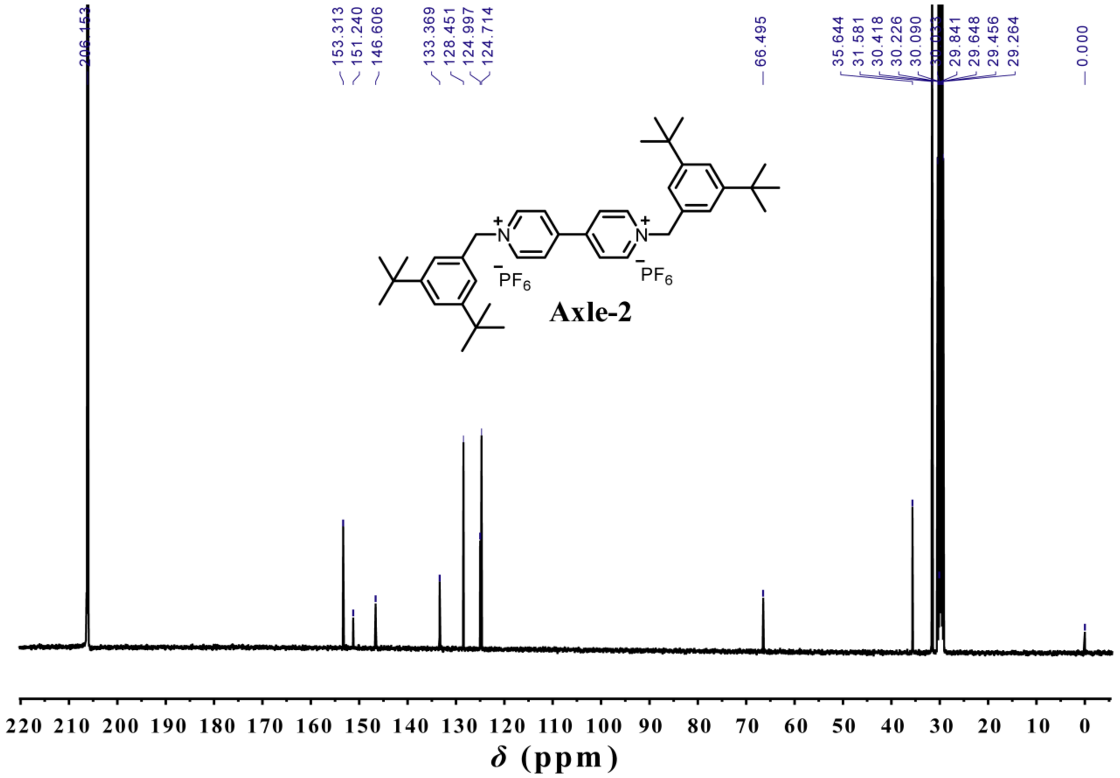

Figure S16 ^{13}C NMR spectrum of **Axle-2** (100 MHz, CD_3COCD_3, 298 K).

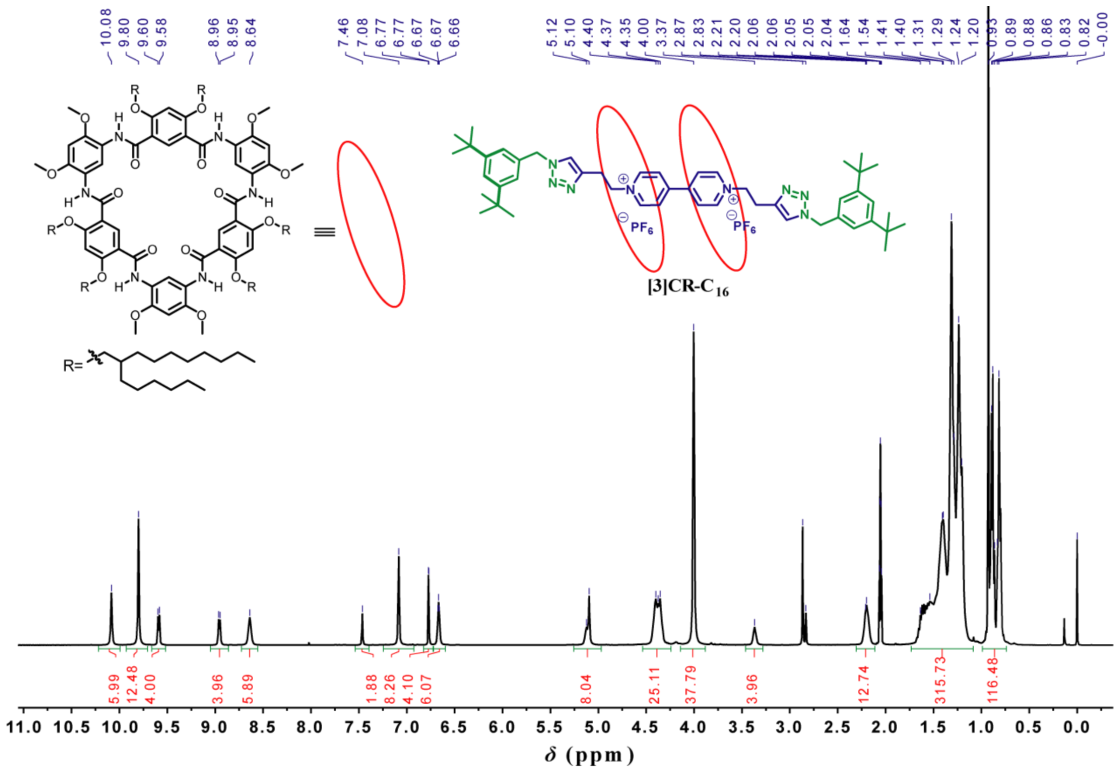

Figure S17 ^{1}H NMR spectrum of **[3]CR-C$_{16}$** (400 MHz, CD_3COCD_3, 298 K).

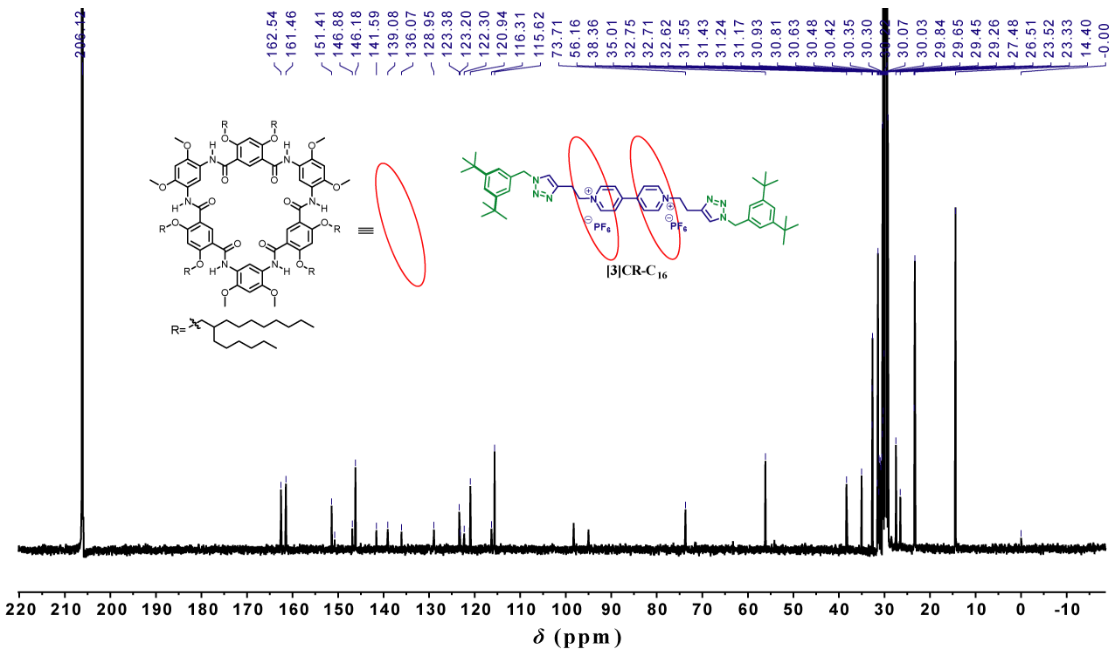

Figure S18 ^{13}C NMR spectrum of compound **[3]CR-C$_{16}$** (100 MHz, CD_3COCD_3, 298 K).

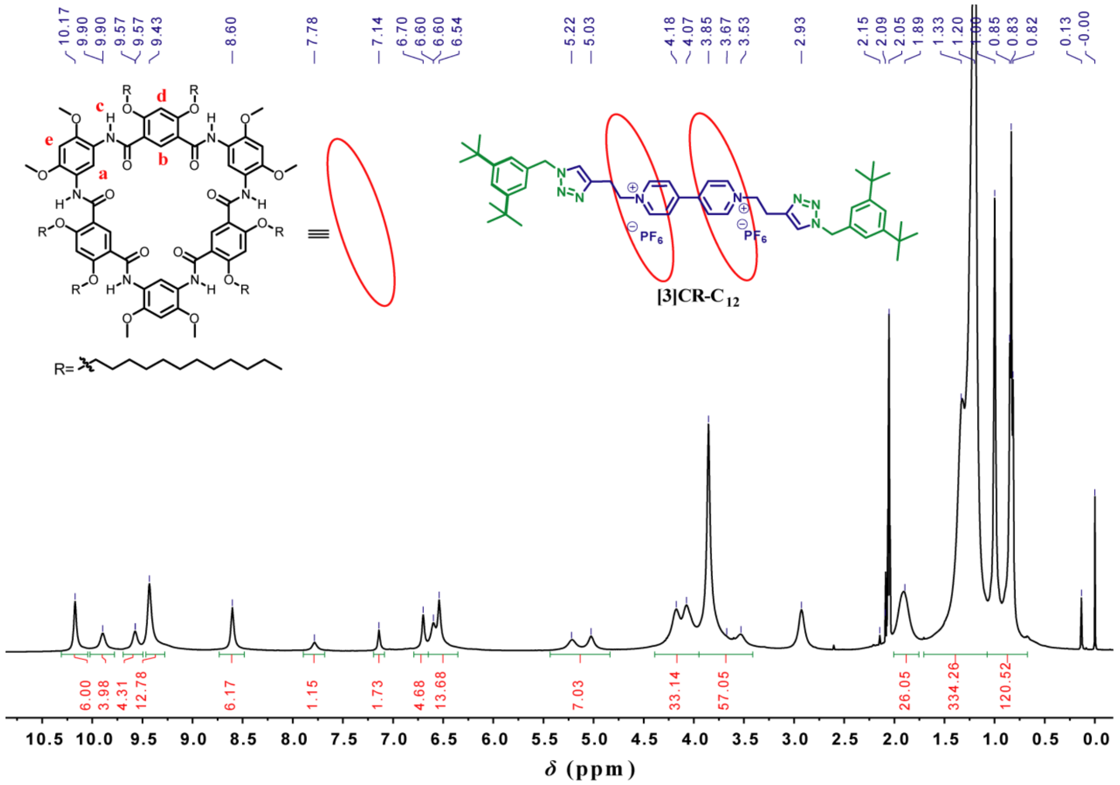

Figure S19 ^{1}H NMR spectrum of **[3]CR-C$_{12}$** (400 MHz, CD_3COCD_3, 298 K).

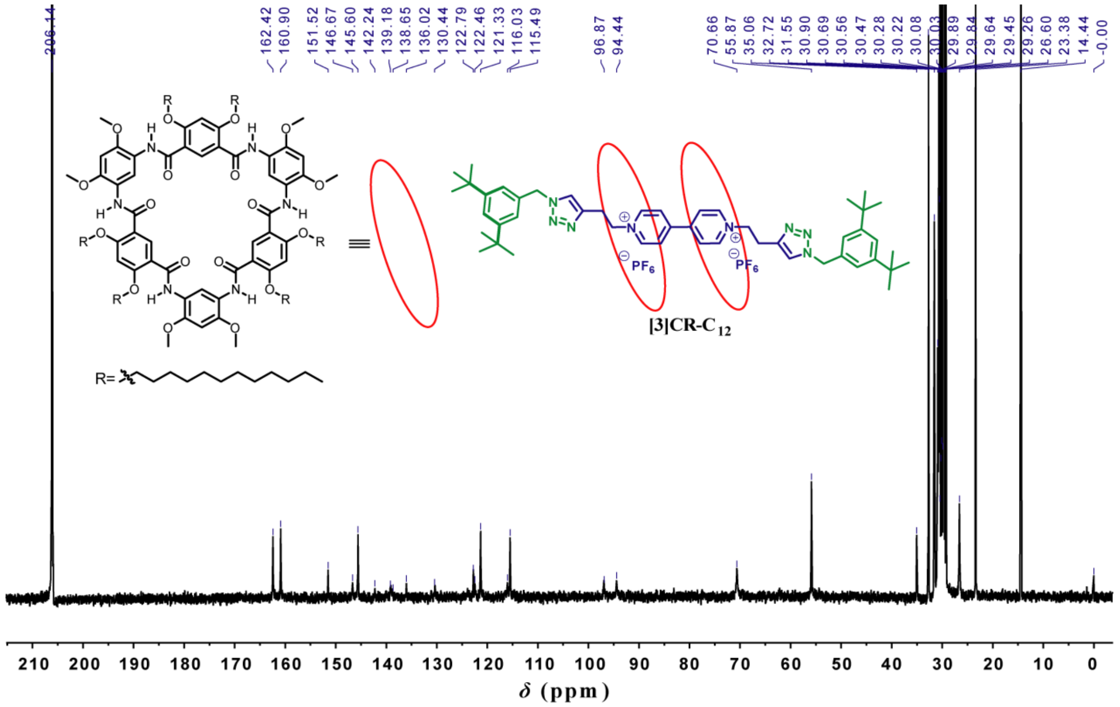

Figure S20 ^{13}C NMR spectrum of compound **[3]CR-C$_{12}$** (100 MHz, CD_3COCD_3, 298 K).

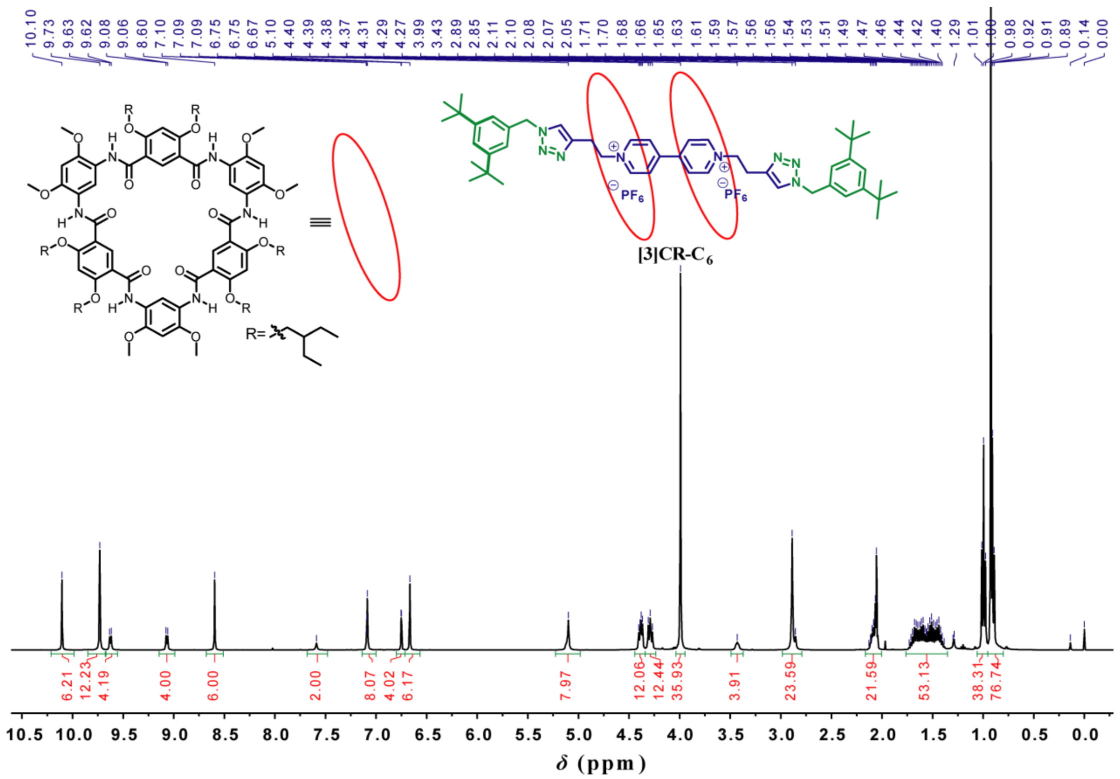

Figure S21 ^{1}H NMR spectrum of **[3]CR-C$_6$** (400 MHz, CD_3COCD_3, 298 K).

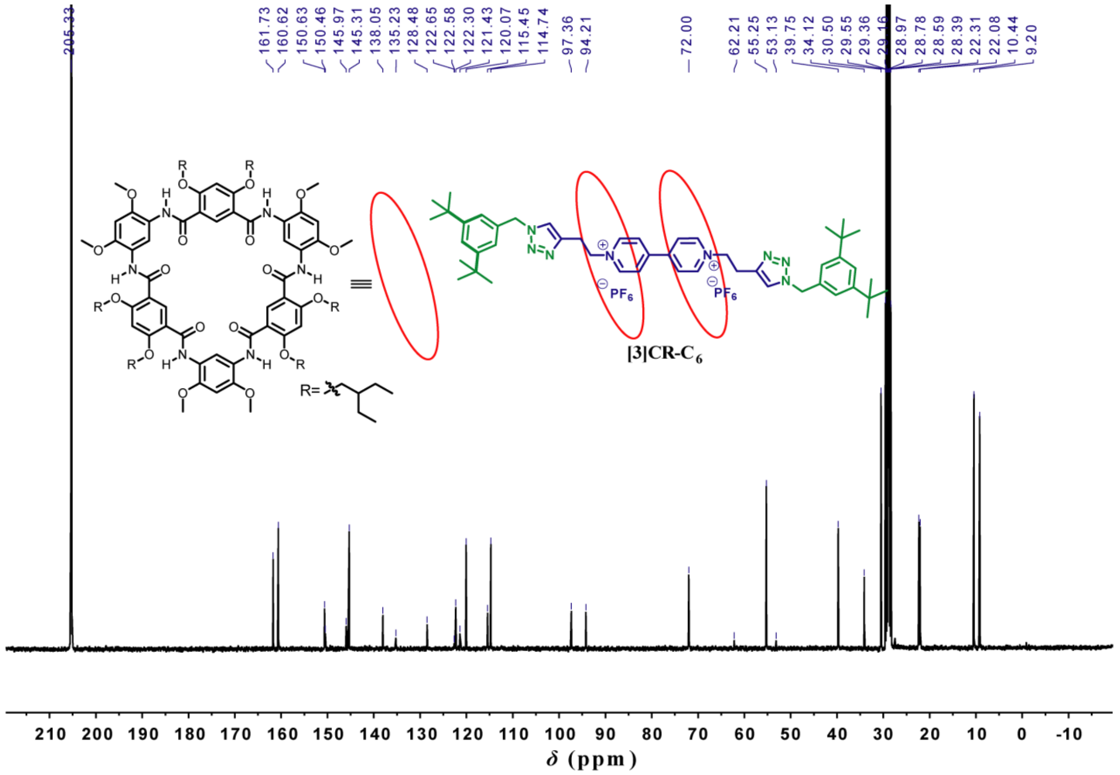

Figure S22 ^{13}C NMR spectrum of compound **[3]CR-C$_6$** (100 MHz, CD_3COCD_3, 298 K).

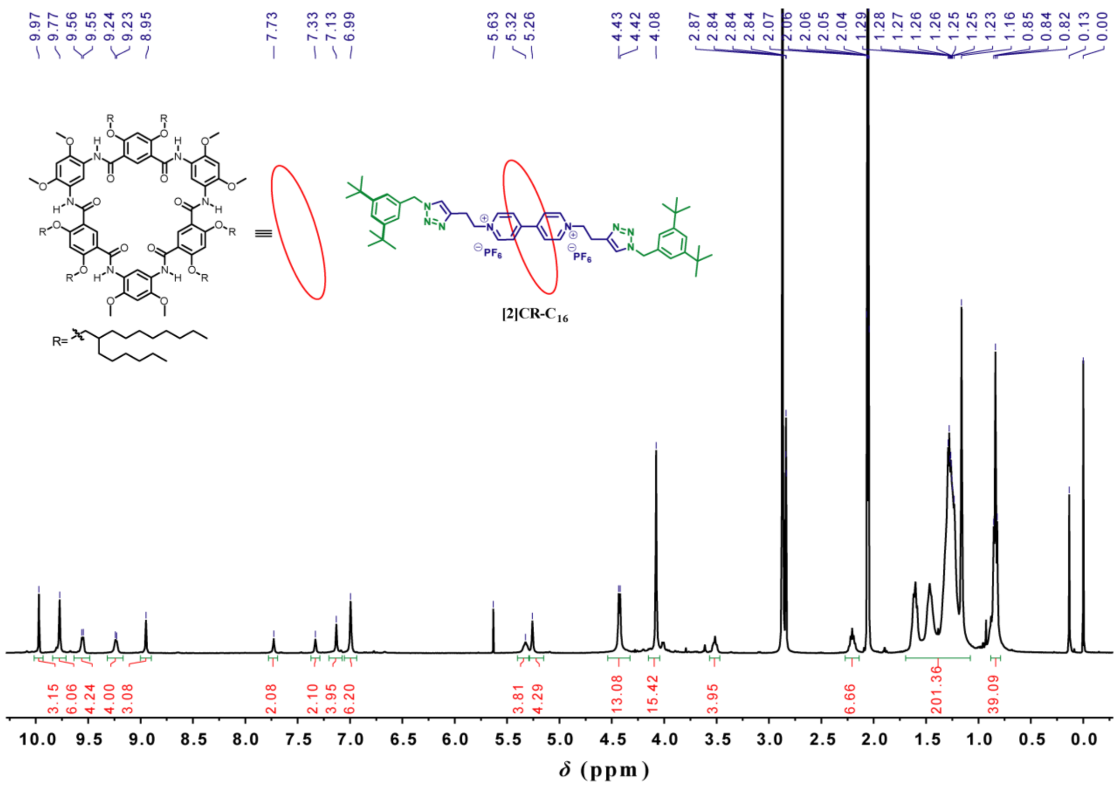

Figure S23 ^{1}H NMR spectrum of **[2]CR-C$_{16}$** (400 MHz, CD_3COCD_3, 298 K).

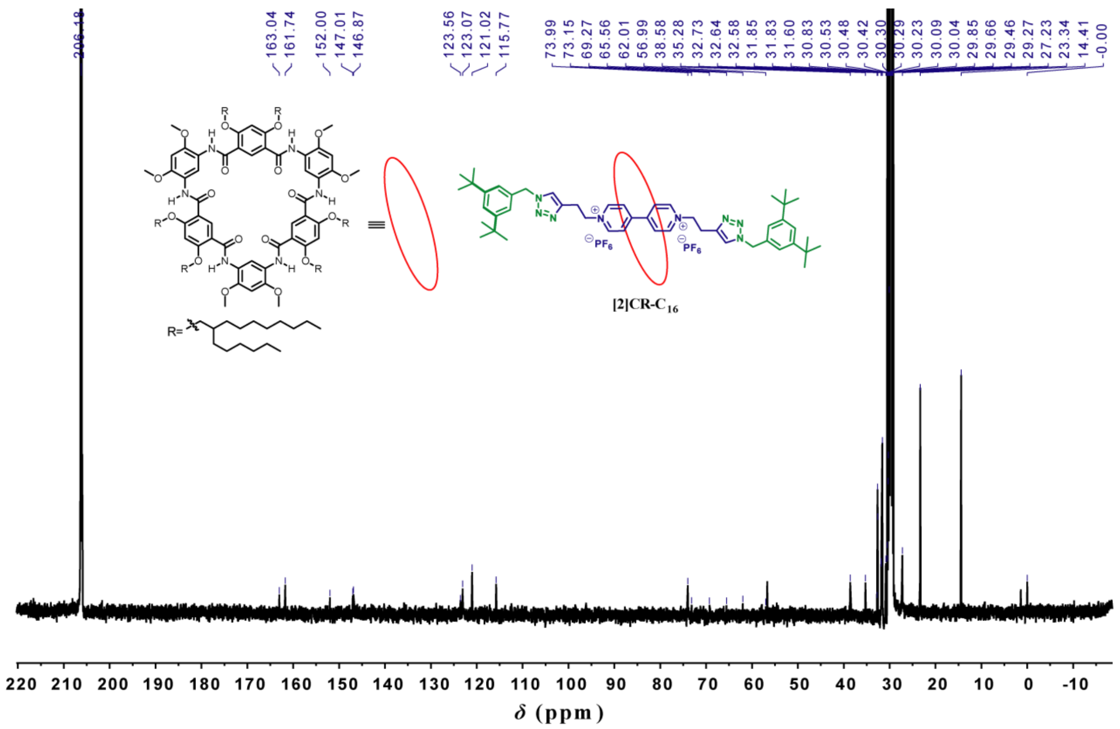

Figure S24 ^{13}C NMR spectrum of compound **[2]CR-C$_{16}$** (100 MHz, CD_3COCD_3, 298 K).

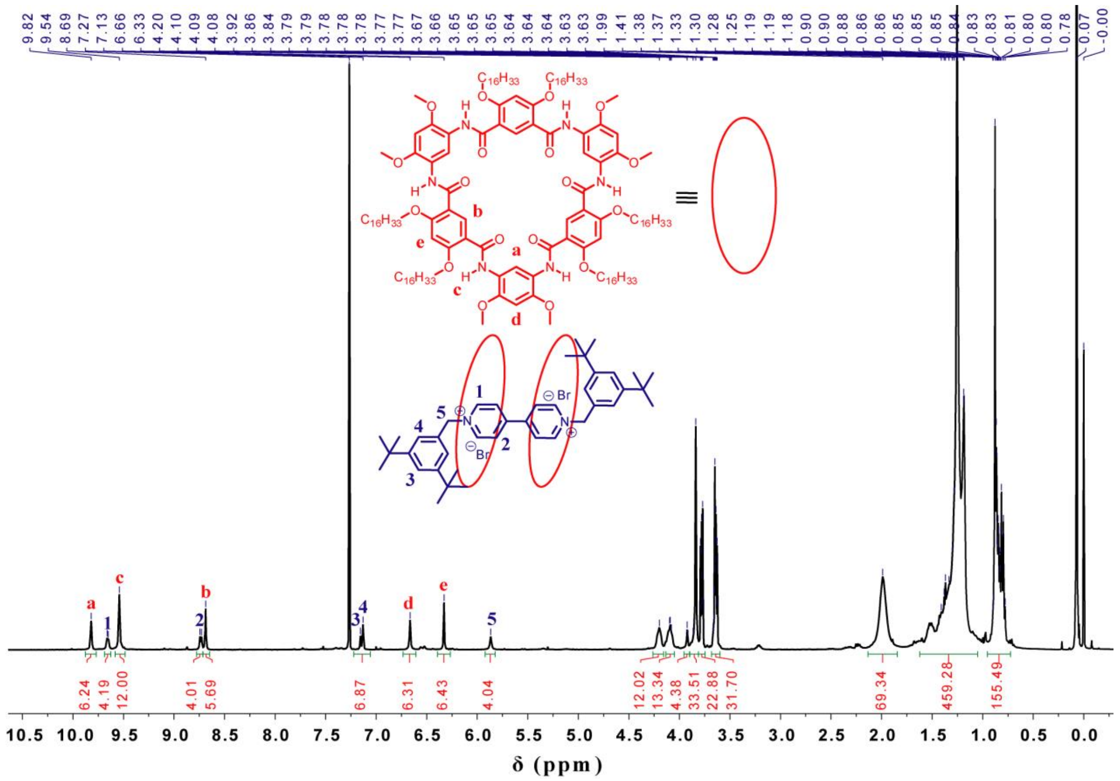

Figure S25 ^{1}H NMR spectrum of compound **[3]R-C$_{16}$-Br** (400 MHz, $CDCl_3$, 298 K).

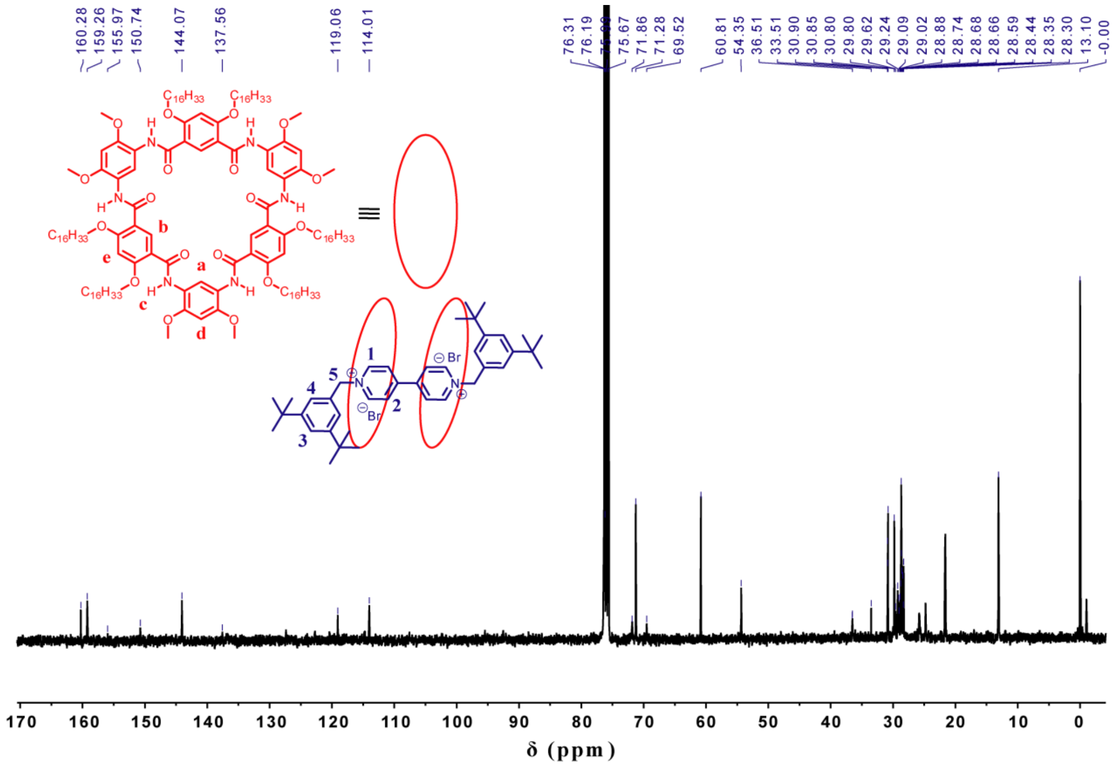

Figure S26 ^{13}C NMR spectrum of compound **[3]R-C$_{16}$-Br** (100 MHz, $CDCl_3$, 298 K).

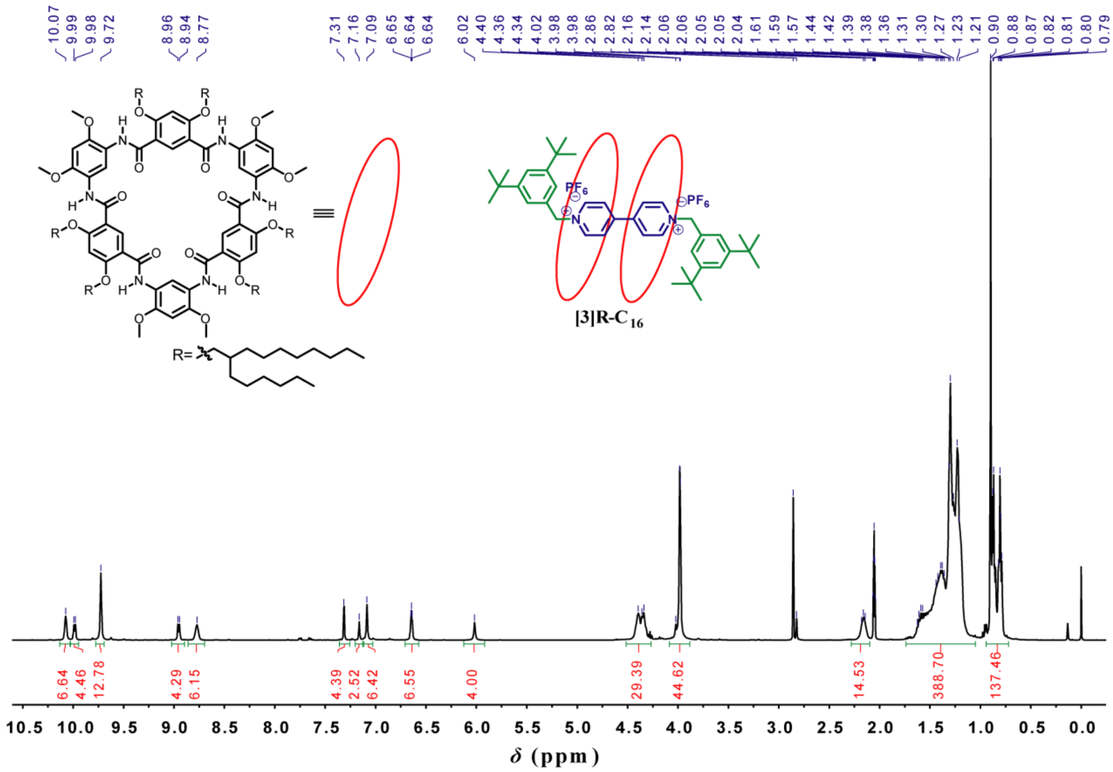

Figure S27 ^{1}H NMR spectrum of compound **[3]R-C$_{16}$** (400 MHz, CD_3COCD_3, 298 K).

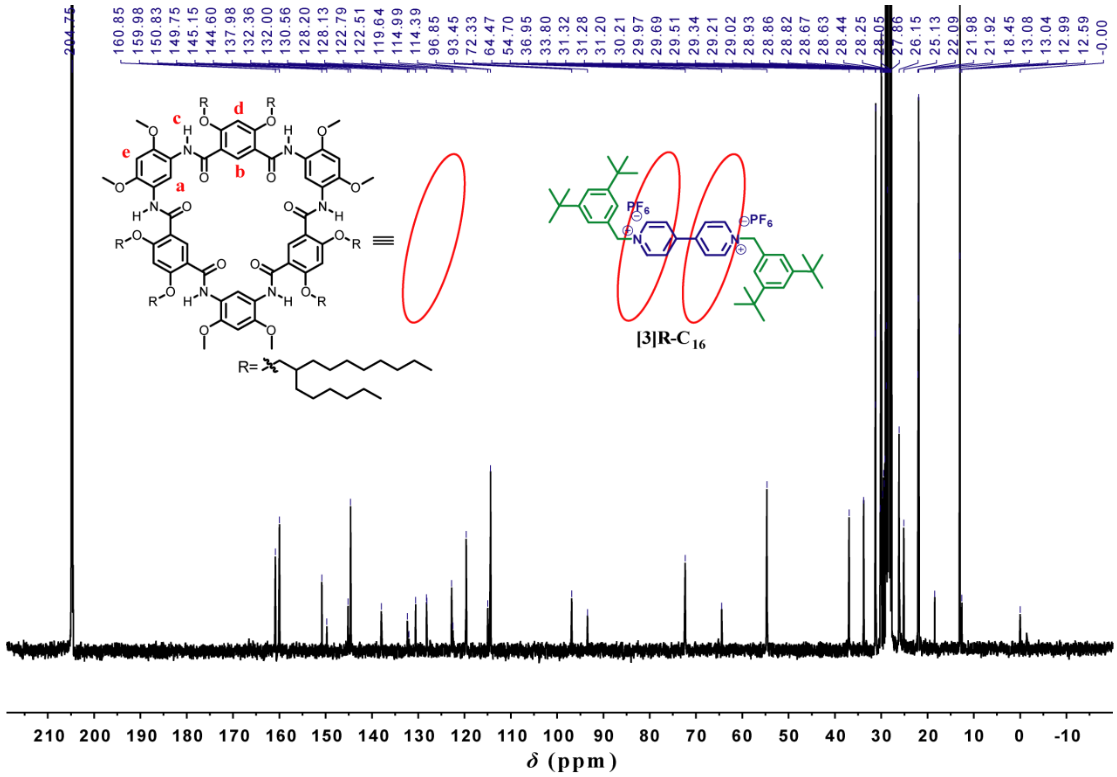

Figure S28 ^{13}C NMR spectrum of compound **[3]R-C$_{16}$** (100 MHz, CD_3COCD_3, 298 K).

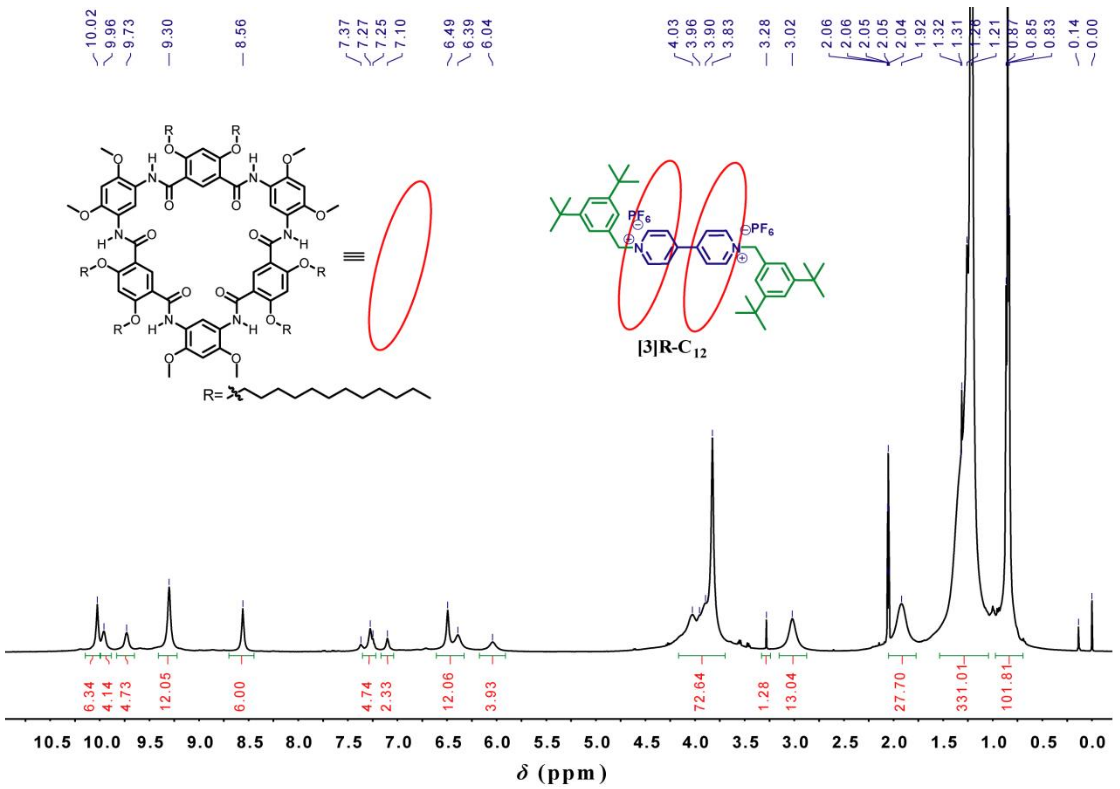

Figure S29 ^{1}H NMR spectrum of compound **[3]R-C$_{12}$** (400 MHz, CD_3COCD_3, 298 K).

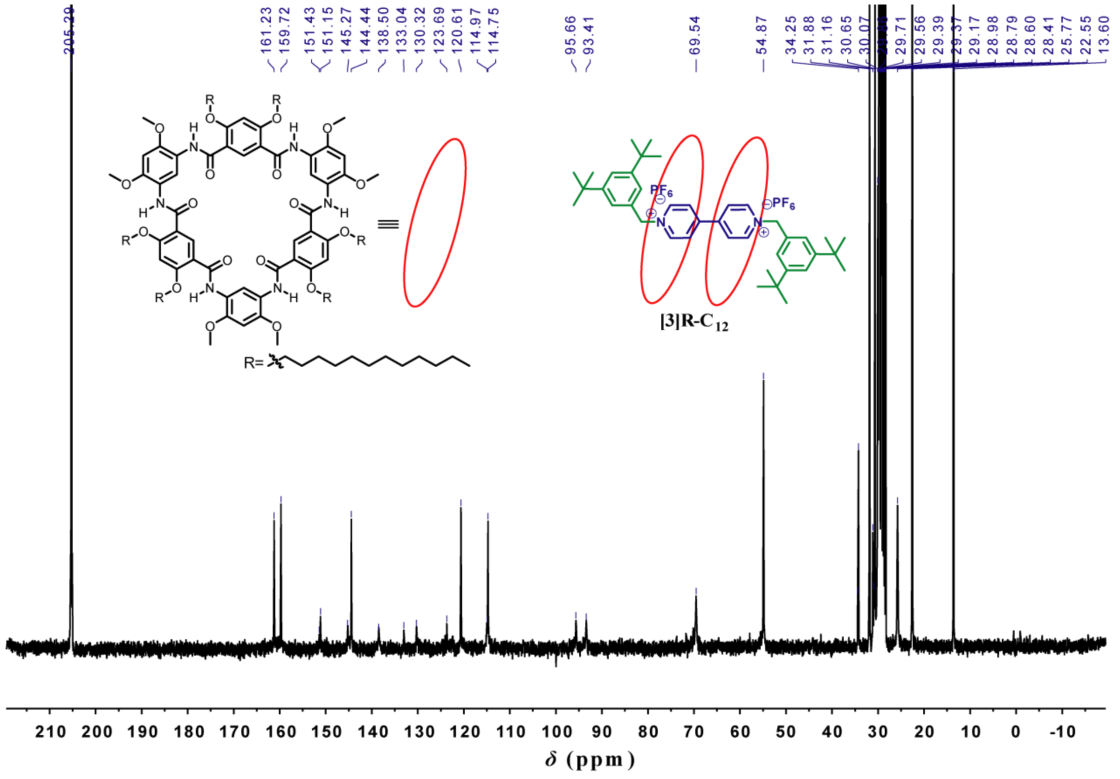

Figure S30 ^{13}C NMR spectrum of compound **[3]R-C$_{12}$** (100 MHz, CD_3COCD_3, 298 K).

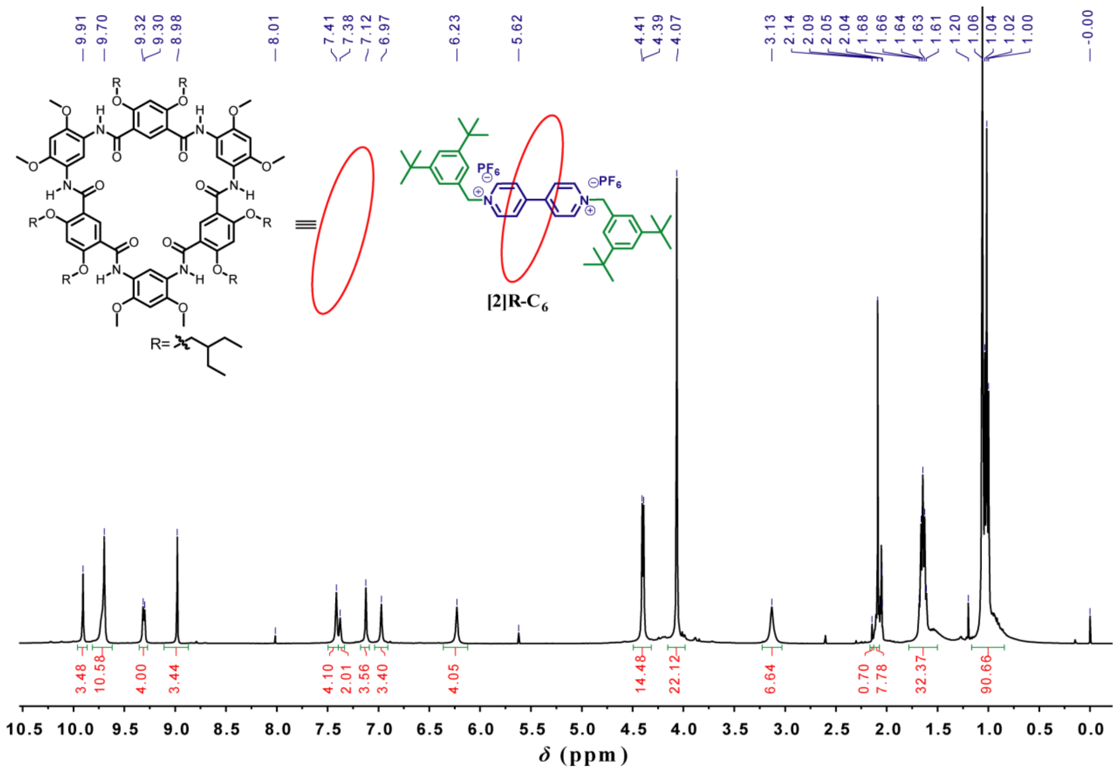

Figure S31 ^{1}H NMR spectrum of compound **[2]R-C$_6$** (400 MHz, CD_3COCD_3, 298 K).

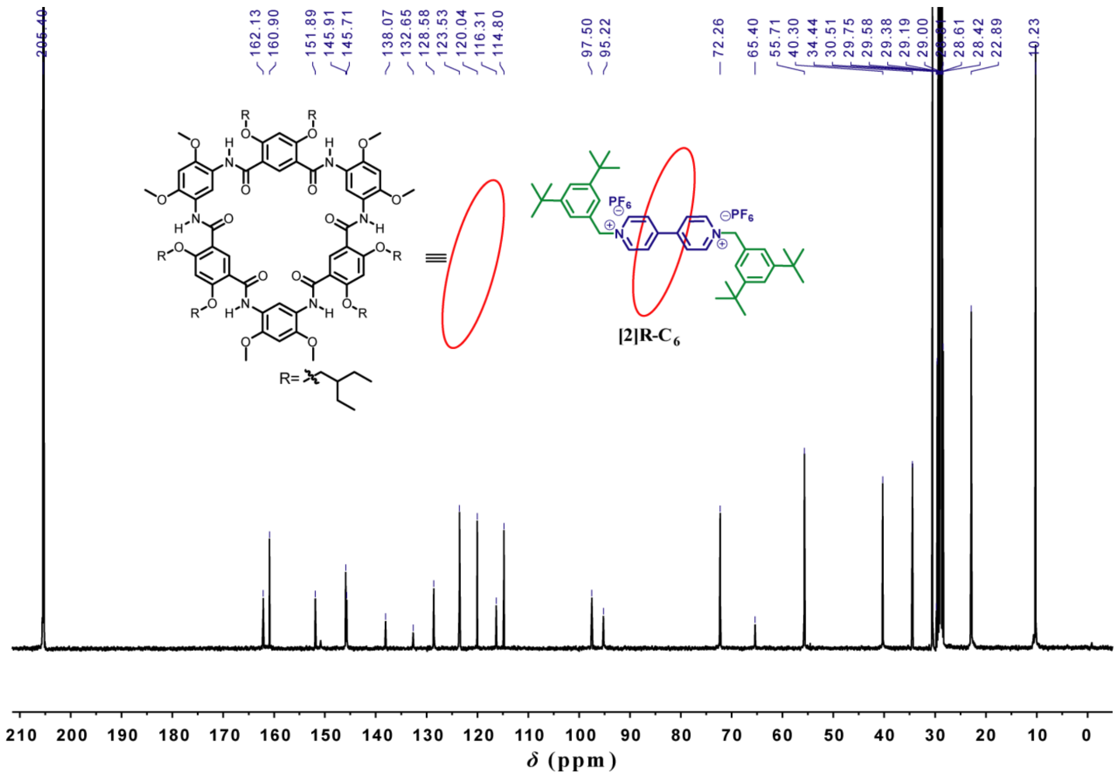

Figure S32 ^{13}C NMR spectrum of compound **[2]R-C$_6$** (100 MHz, CD_3COCD_3, 298 K).

3.2 MALDI-TOF-MS or HRESI-MS Spectra of Novel Compounds

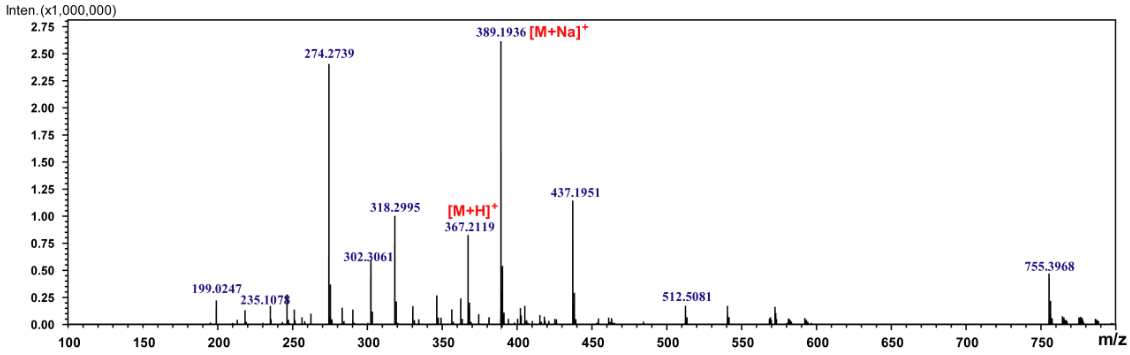

Figure S33 HRESI-MS spectrum of compound **5.**

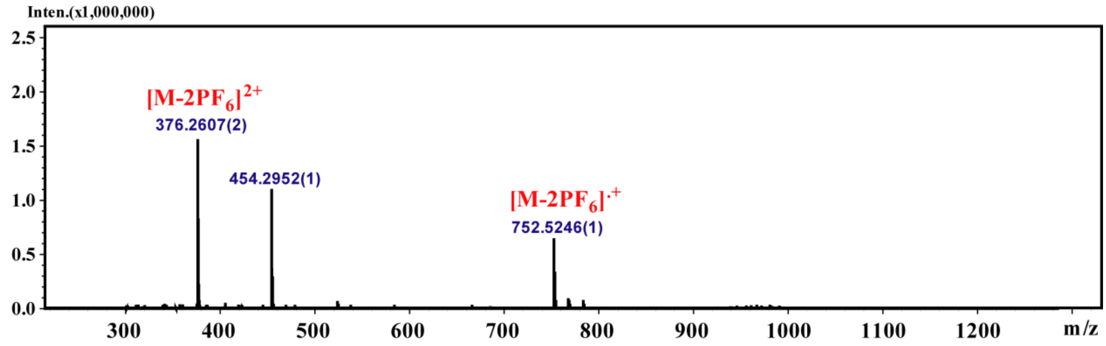

Figure S34 HRESI-MS spectrum of **Axle-1.**

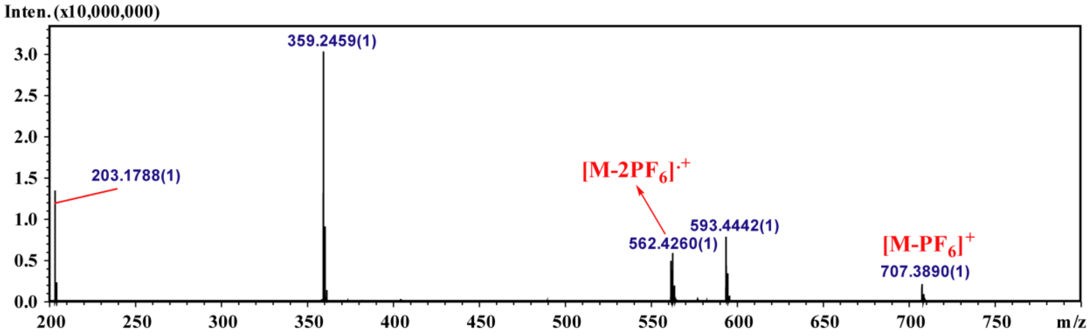

Figure S35 HRESI-MS spectrum of **Axle-2.**

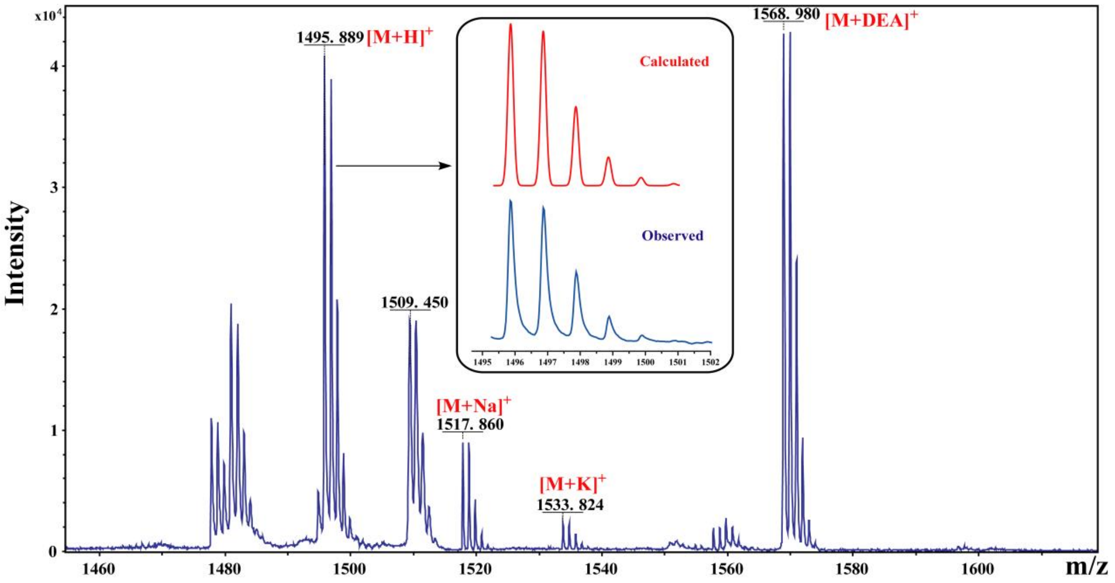

Figure S36 MALDI-TOF mass spectrum of cyclo[6]aramide **3** (inset: experimental isotope distribution (blue) and computer simulation (red)).

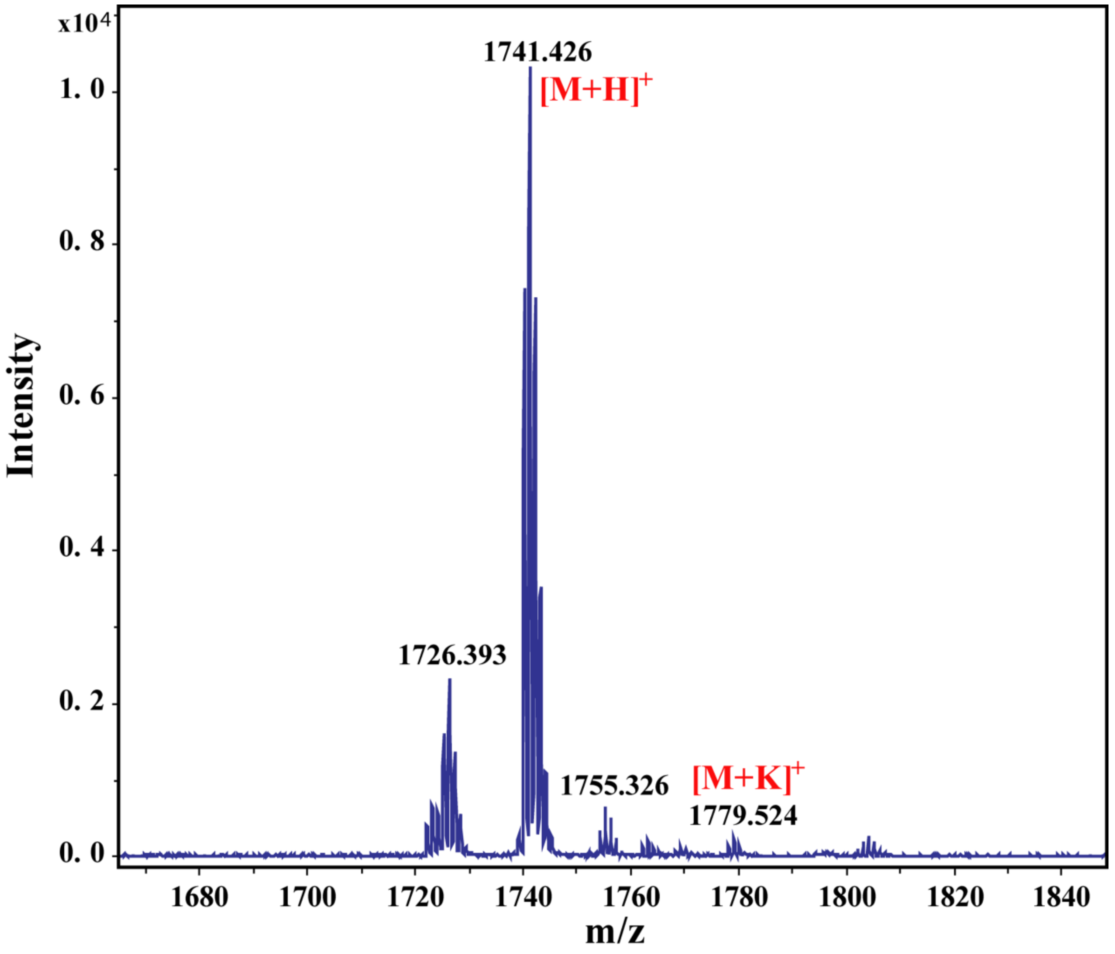

Figure S37 MALDI-TOF mass spectrum of cyclo[6]aramides **5.**

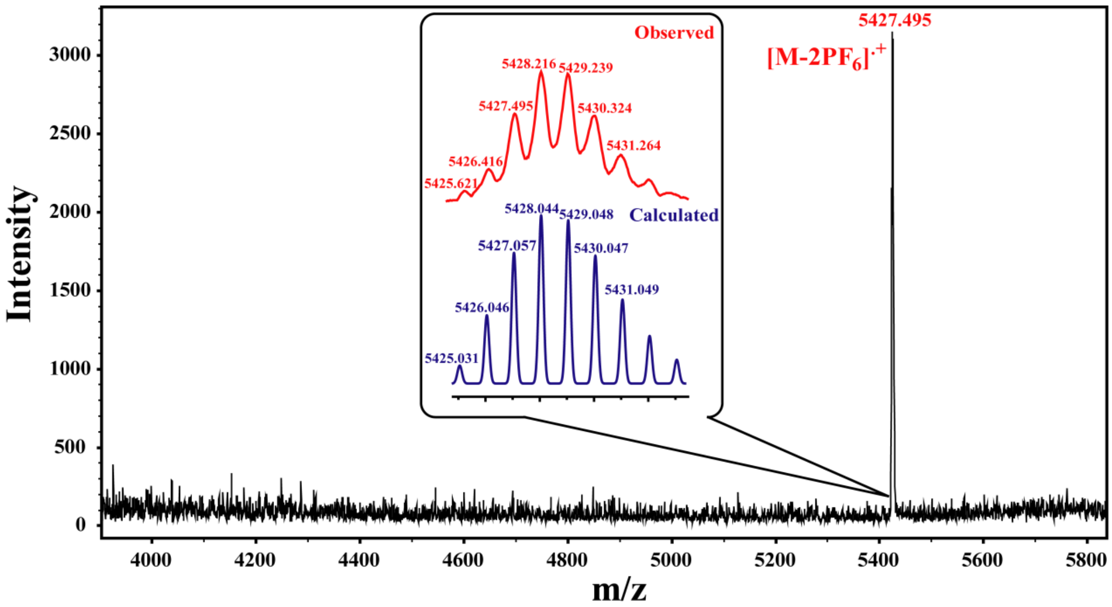

Figure S38 MALDI-TOF mass spectrum of **[3]CR-C$_{16}$** (inset: experimental isotope distribution (red) and computer simulation (blue)).

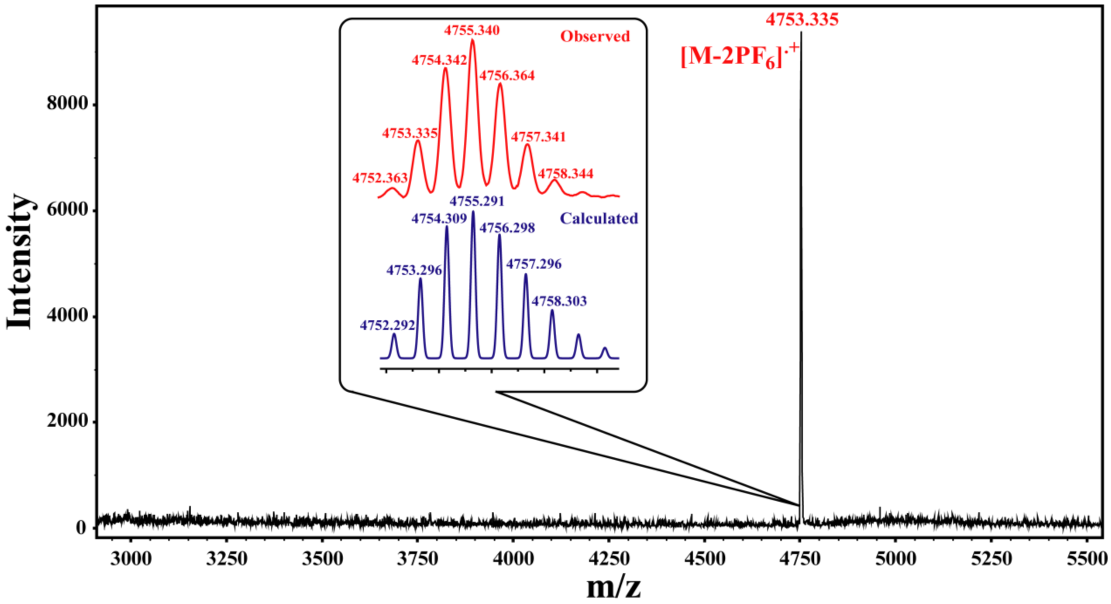

Figure S39 MALDI-TOF mass spectrum of **[3]CR-C$_{12}$** (inset: experimental isotope distribution (red) and computer simulation (blue)).

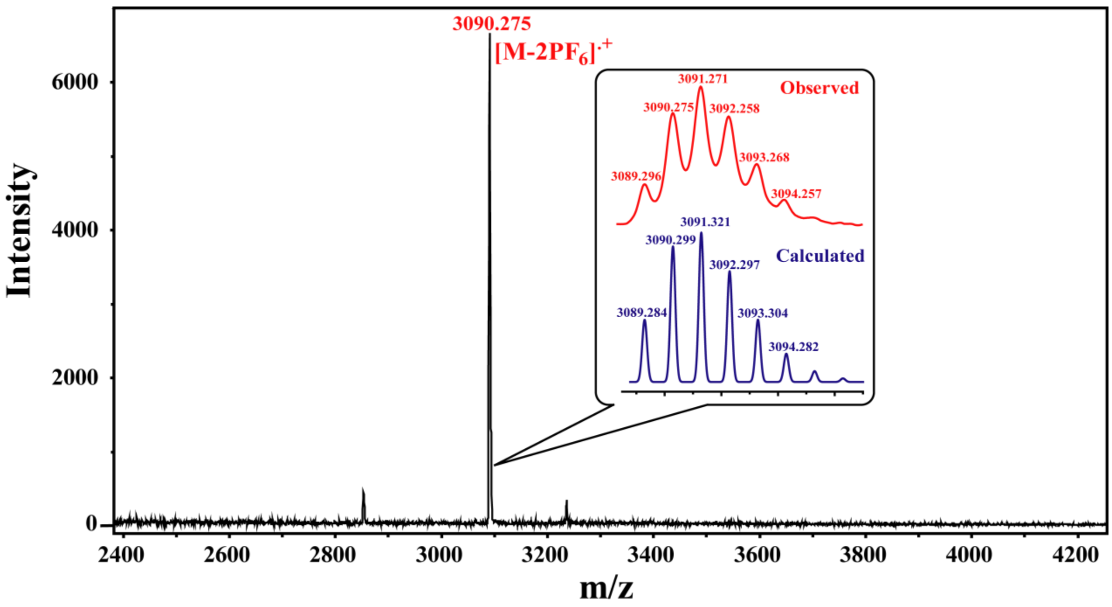

Figure S40 MALDI-TOF mass spectrum of **[2]CR-C$_{16}$** (inset: experimental isotope distribution (red) and computer simulation (blue)).

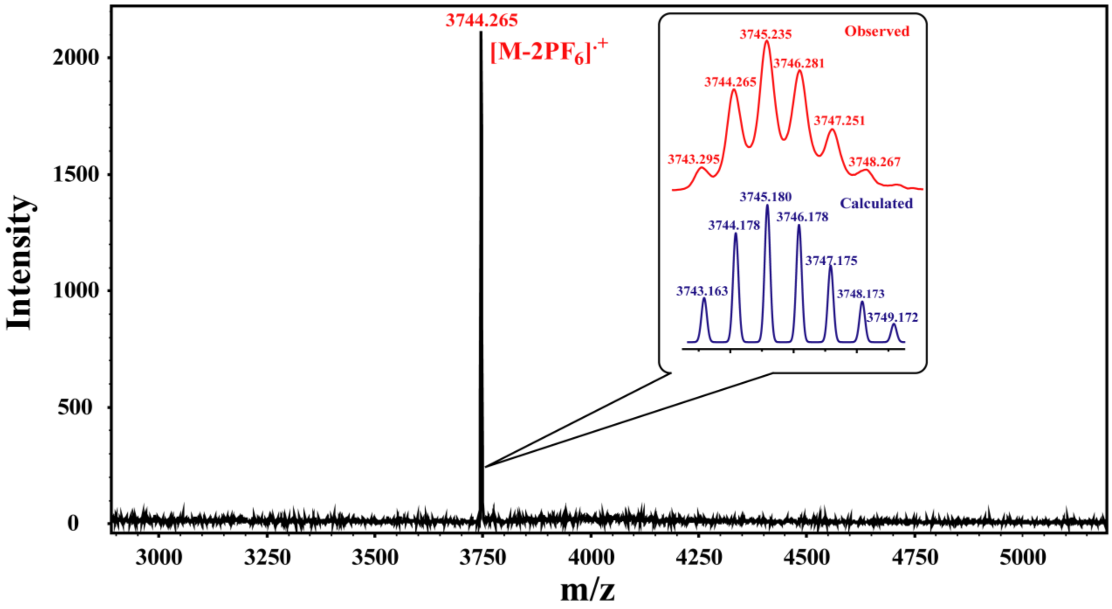

Figure S41 MALDI-TOF mass spectrum of **[3]CR-C$_{6}$** (inset: experimental isotope distribution (red) and computer simulation (blue)).

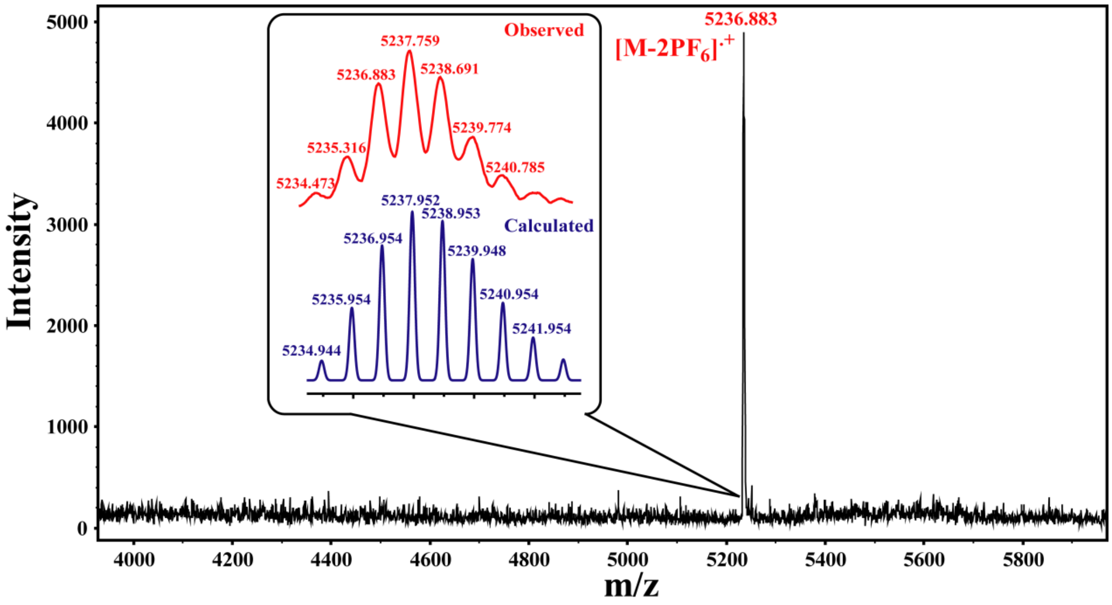

Figure S42 MALDI-TOF mass spectrum of **[3]R-C$_{16}$** (inset: experimental isotope distribution (red) and computer simulation (blue)).

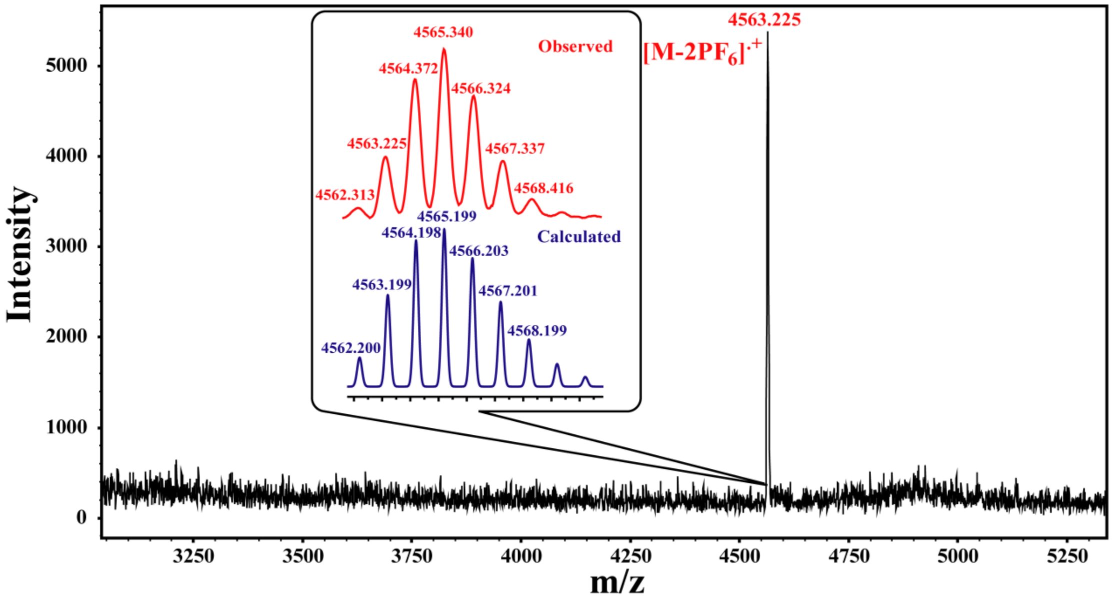

Figure S43 MALDI-TOF mass spectrum of **[3]R-C$_{12}$** (inset: experimental isotope distribution (red) and computer simulation (blue)).

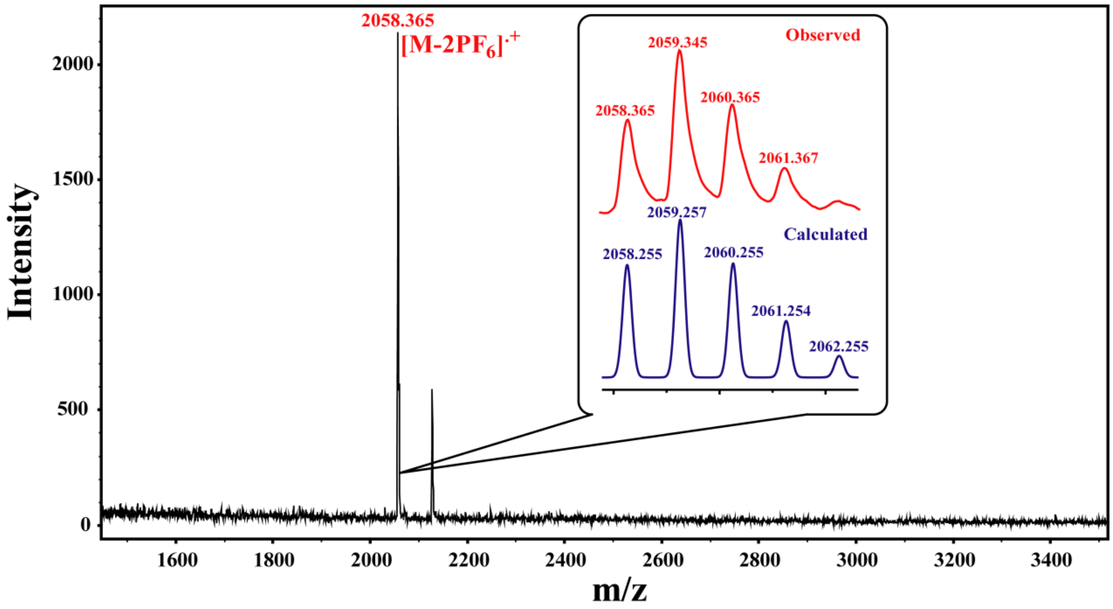

Figure S44 MALDI-TOF mass spectrum of **[2]R-C_6** (inset: experimental isotope distribution (red) and computer simulation (blue)).

4. Host-Guest Complexation of 1 and G1-G4

4.1 NMR Spectra of Complexation

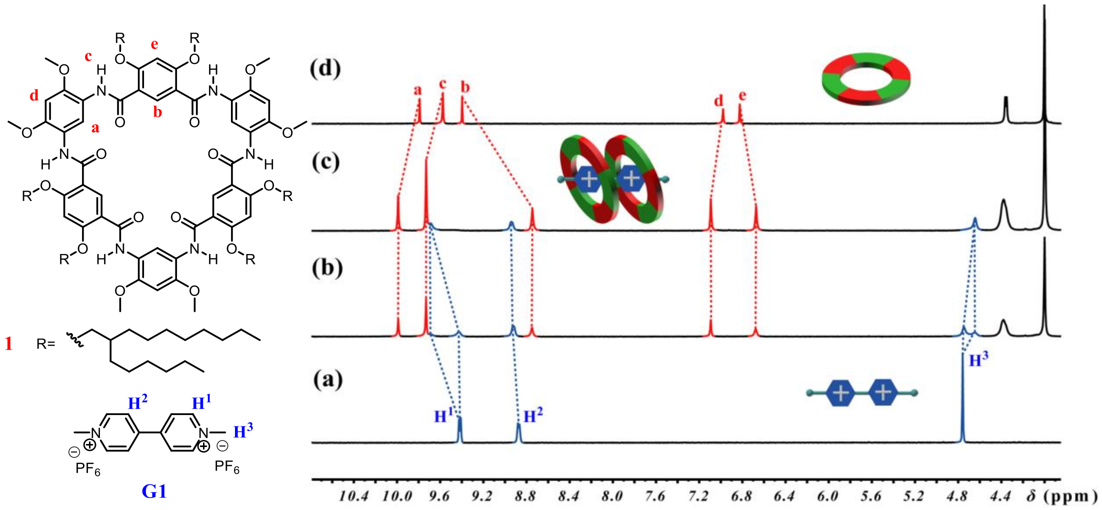

Figure S45 Partial ^{1}H NMR spectra (400 MHz, acetone-d_6, 298 K) of (a) 2.0 mM **G1**, (b) 2.0 mM **1** and **G1**, (c) 4.0 mM **1** and 2.0 mM **G1**, (d) 2.0 Mm **1**.

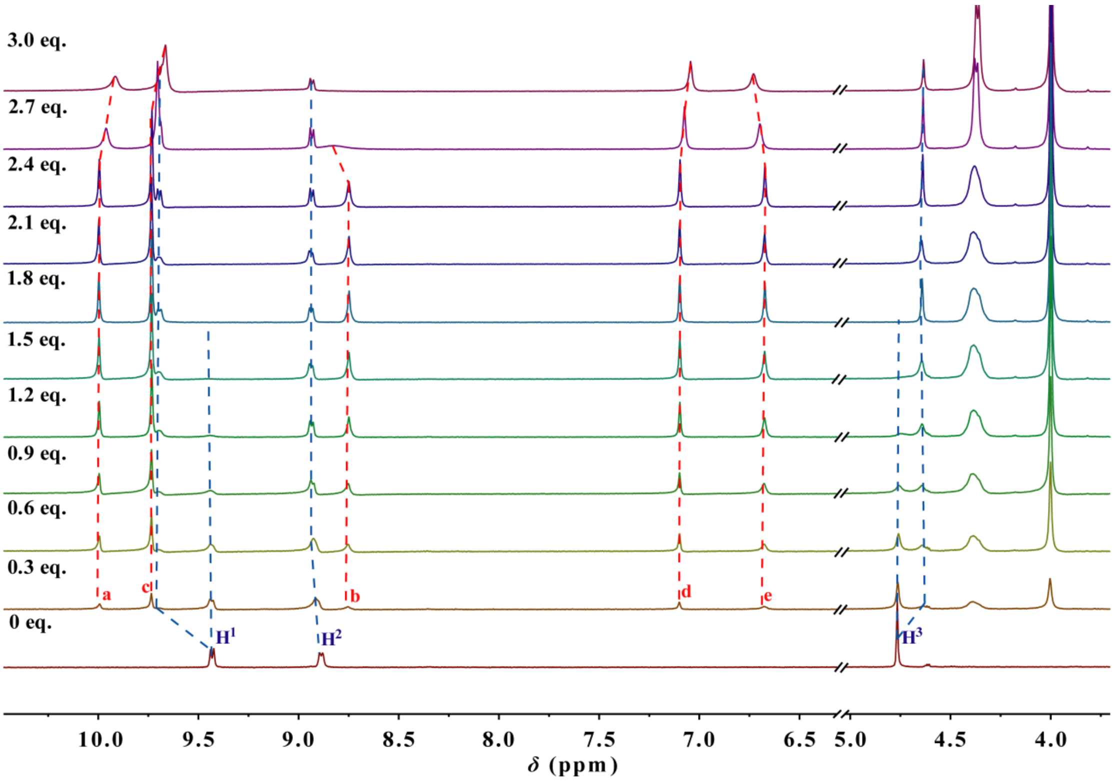

Figure S46 Stacked plots of ^{1}H NMR spectra of **G1** (1 mM) titrated with **1** (0-3.0 mM) in acetone-d_6 (400 MHz, 298 K).

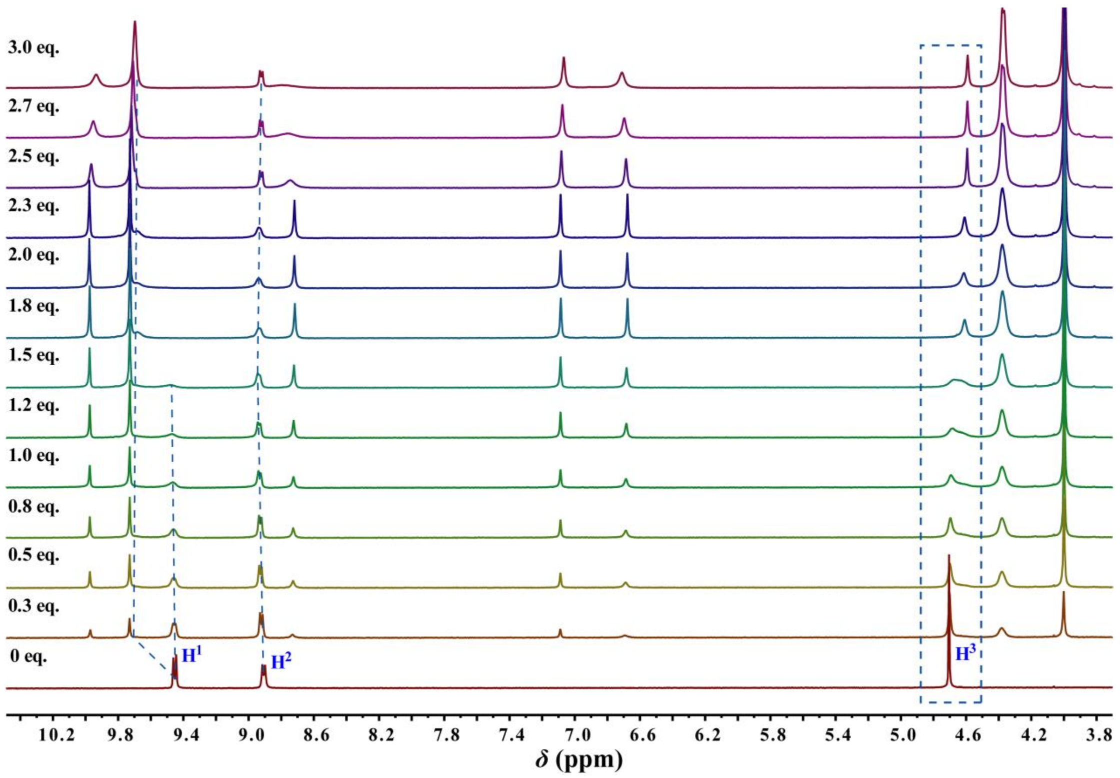

Figure S47 Stacked plots of ^{1}H NMR spectra of **G1** (1 mM) titrated with **1** (0-3.0 mM) in acetone-d_6/DMSO-d_6 (9/1, v/v) (400 MHz, 298 K).

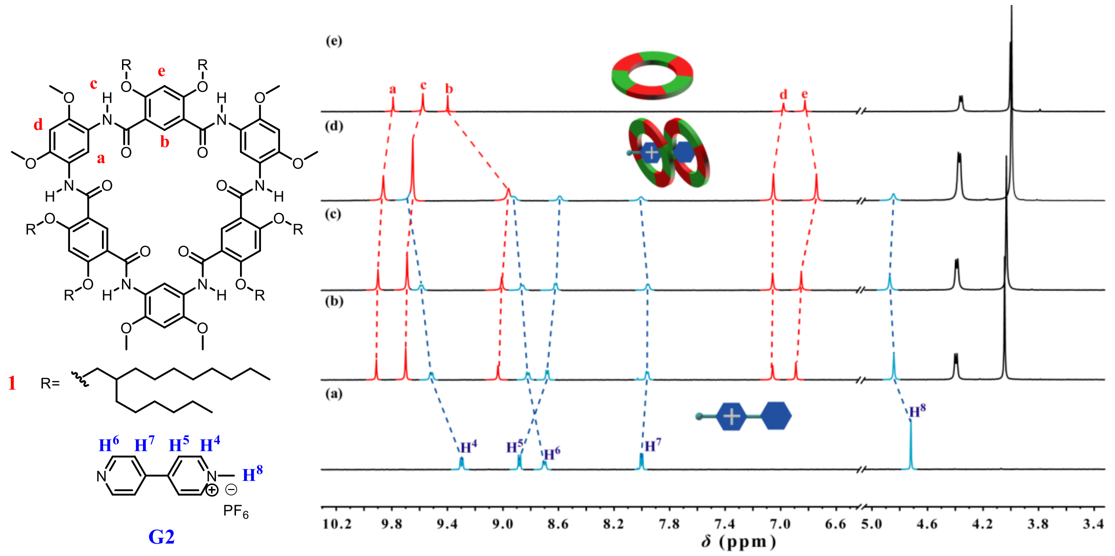

Figure S48 Partial ^{1}H NMR spectra (400 MHz, acetone-d_6, 298 K) of (a) 1.0 mM **G2**, (b) 0.5 mM **1** and 1.0 mM **G2**, (c) 1.0 mM **1** and 1.0 mM **G2**, (d) 2.0 mM **1** and 1.0 mM **G2**, (e) 1.0 Mm **1**.

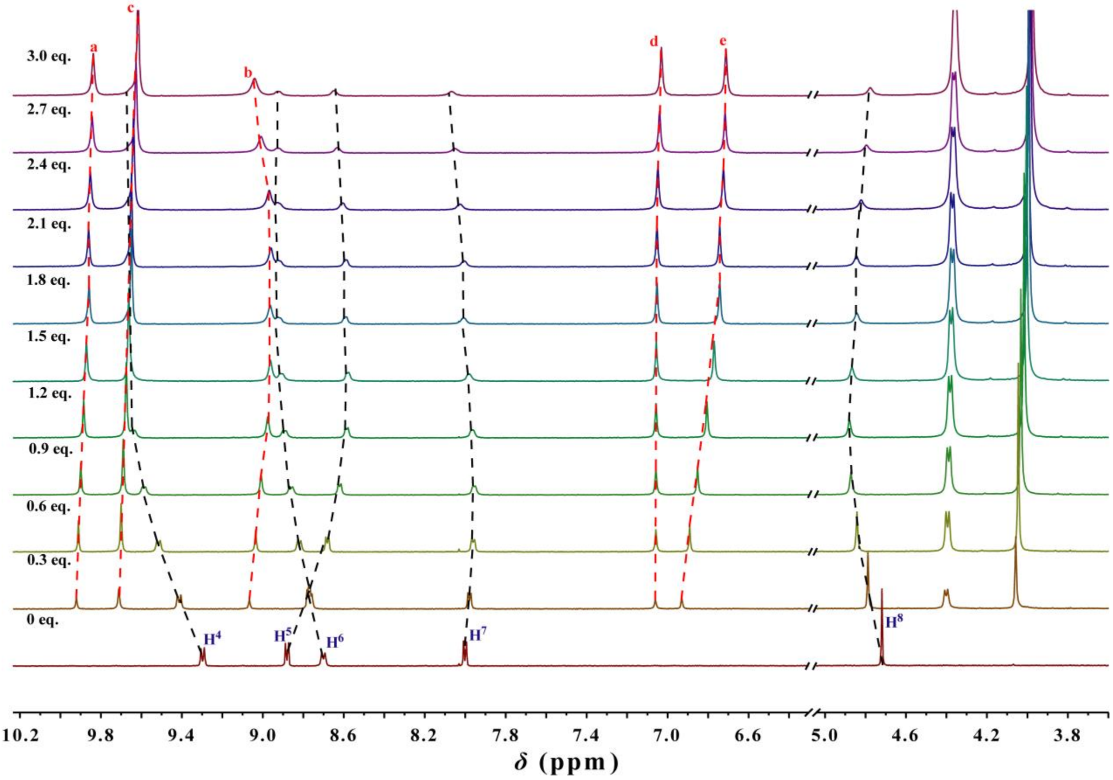

Figure S49 Stacked plots of ^{1}H NMR spectra of **G2** (1 mM) titrated with **1** (0-3.0 mM) in acetone-d$_6$ (400 MHz, 298 K).

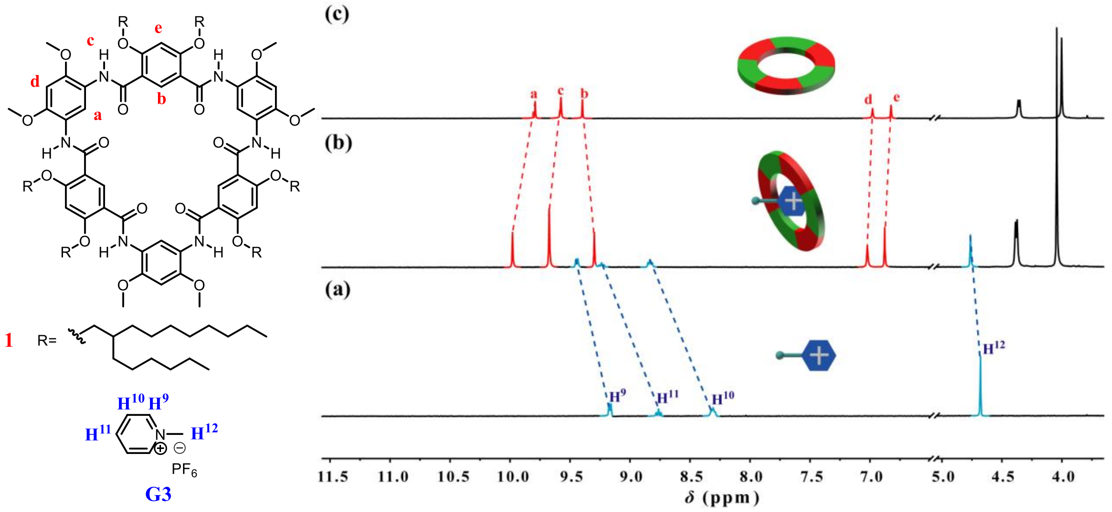

Figure S50 Partial ^{1}H NMR spectra (400 MHz, acetone-d$_6$, 298 K) of (a) 1.0 mM **G3**, (b) 1.0 mM **1** and 1.0 mM **G3**, (c) 1.0 Mm **1**.

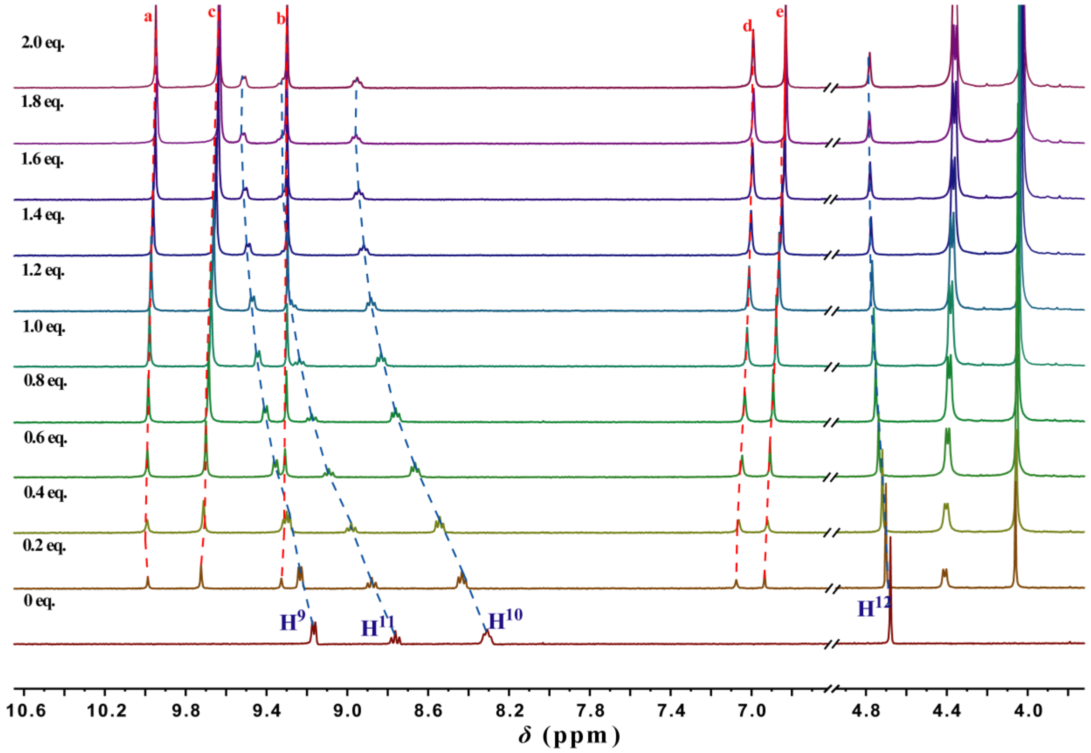

Figure S51 Stacked plots of ^{1}H NMR spectra of **G3** (1 mM) titrated with **1** (0-2.0 mM) in acetone-d$_6$ (400 MHz, 298 K).

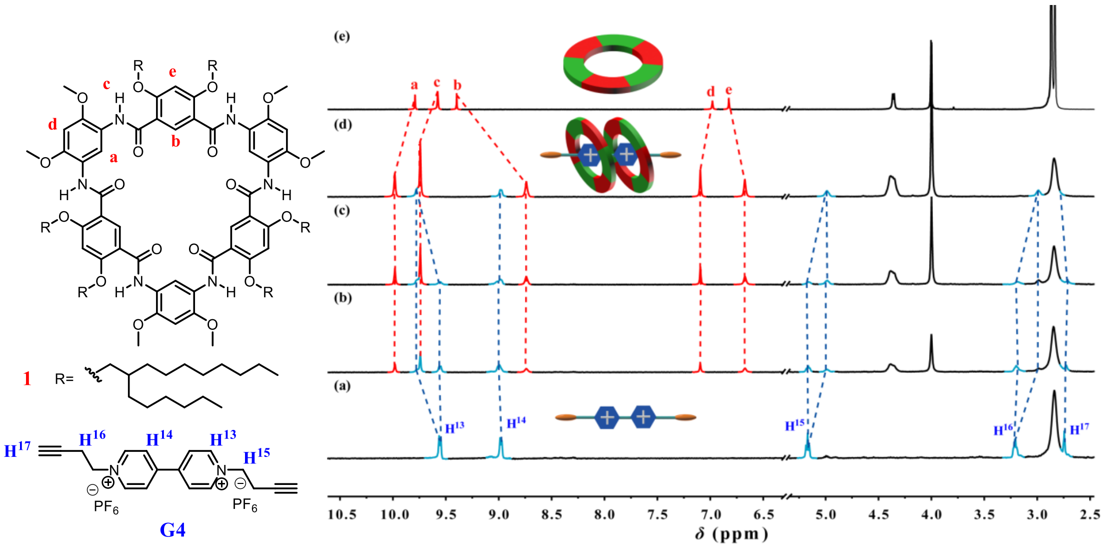

Figure S52 Partial ^{1}H NMR spectra (400 MHz, acetone-d$_6$, 298 K) of (a) 1.0 mM **G4**, (b) 0.5 mM **1** and 1.0 mM **G4**, (c) 1.0 mM **1** and 1.0 mM **G4**, (d) 2.0 mM **1** and 1.0 mM **G4**, (e) 1.0 Mm **1**.

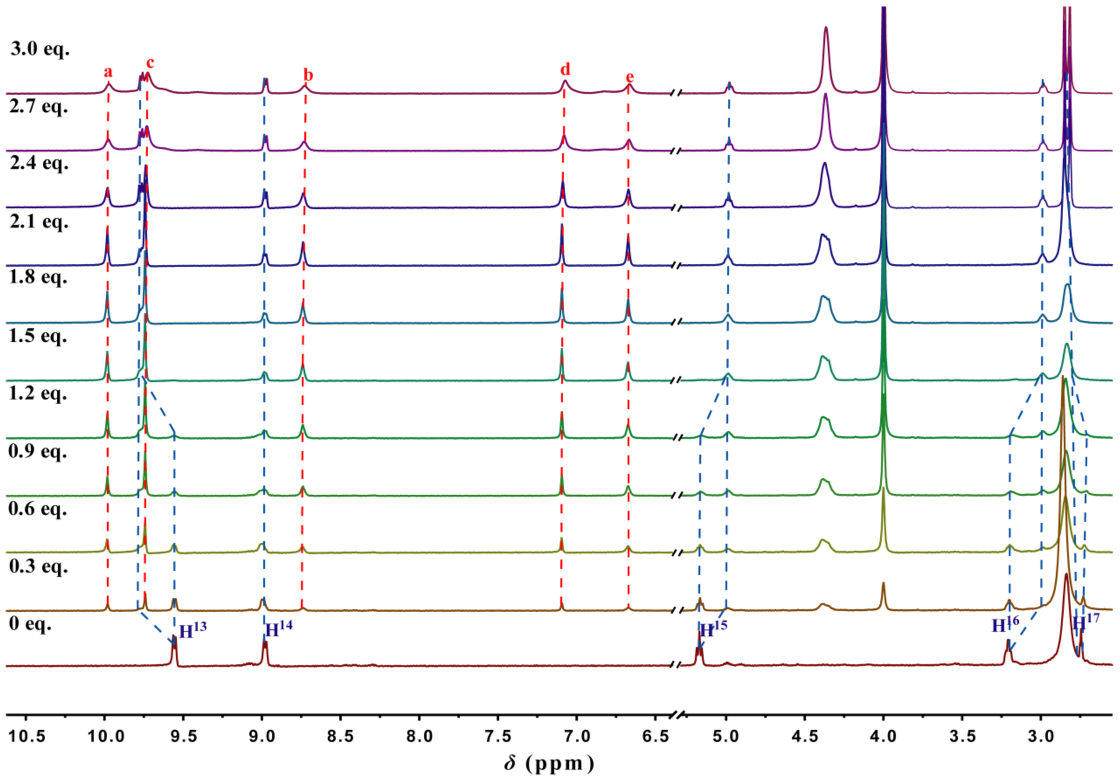

Figure S53 Stacked plots of ^{1}H NMR spectra of **G4** (1 mM) titrated with **1** (0-3.0 mM) in acetone-d_6 (400 MHz, 298 K).

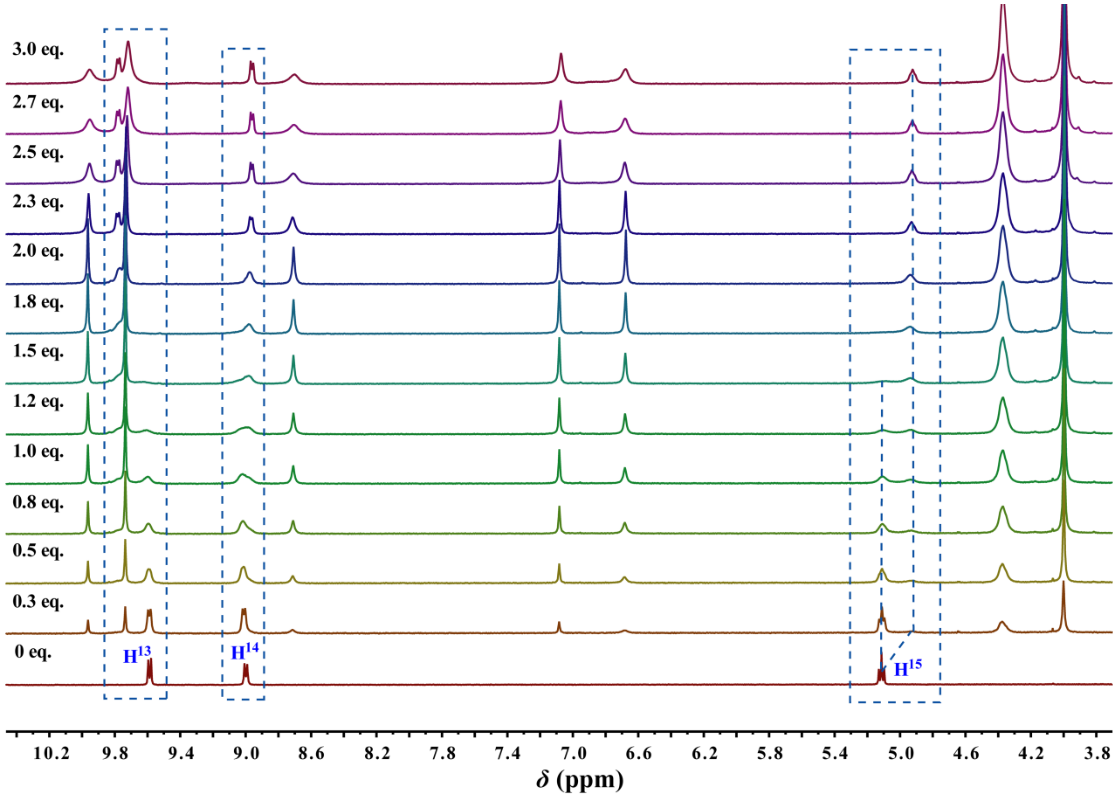

Figure S54 Stacked plots of ^{1}H NMR spectra of **G4** (1 mM) titrated with **1** (0-3.0 mM) in acetone-d_6/DMSO-d_6 (9/1, v/v) (400 MHz, 298 K).

4.2 2D NMR Spectra of Host-Guest Complexes

4.2.1 2D-NOESY Spectra of Host-Guest Complexes

NOESY NMR spectroscopic studies were carried out in an effort to elucidate the nature of the host-guest interactions between cyclo[6]aramide **1** and pyridinium guests **G1**-**G4** in CD_3COCD_3 solution. In the resulting spectra of $\mathbf{1}_2 \supset$ **G1**, correlations between (H^a, H^b) and H^2, (H^a, H^b) and H^3 were observed. In the resulting spectra of **1** $\supset$ **G4**, correlations between H^a and (H^{13}, H^{14}, H^{15}), H^b and (H^{13}, H^{14}, H^{15}), were observed. These spectra are consistent with the guests complexed in the cavities of cyclo[6]aramides and the threaded binding mode shown in **Scheme 1** of the main text. Also, in the resulting spectra of $\mathbf{1}_2 \supset$ **G2**, correlations between H^a and (H^5, H^6, H^7, H^8), H^b and (H^6, H^8) were observed. In the resulting spectra of **1** $\supset$ **G3**, correlations between H^a and (H^9, H^{10}, H^{12}), H^b and H^{12} were observed.

2D-NOESY Spectra of $\mathbf{1}_2 \supset$ G1

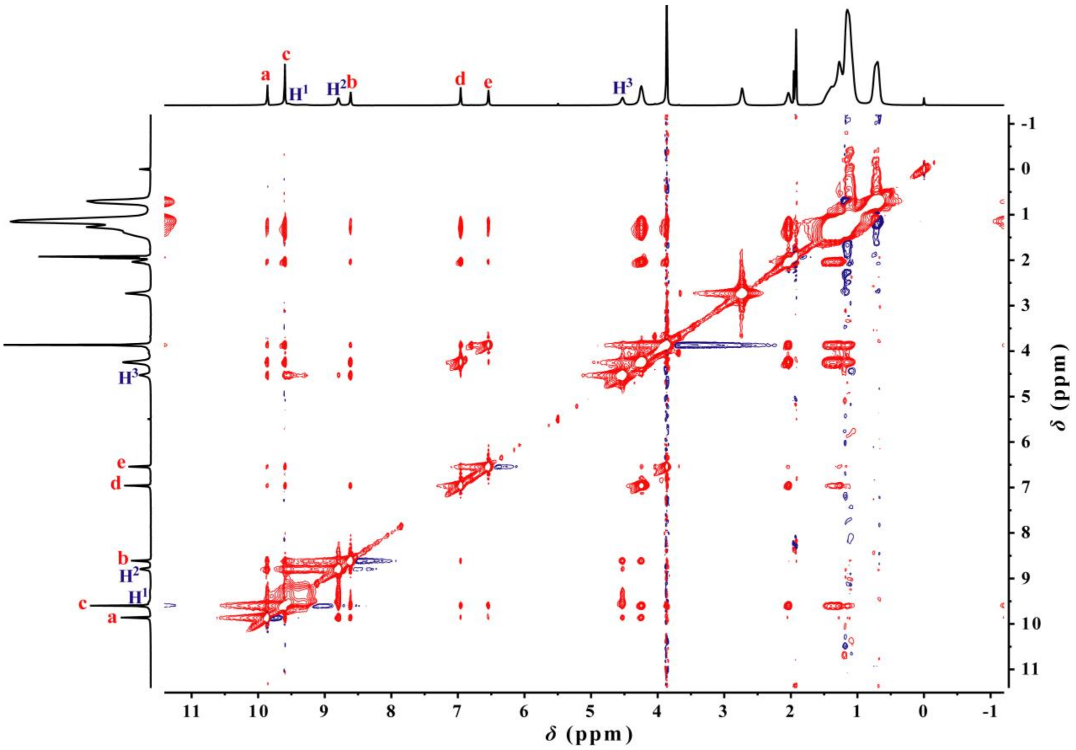

Figure S55 2D-NOESY spectra of $\mathbf{1}_2 \supset$ **G1** ([**1**] = 10 mM, [**G1**] = 5 mM, **1** : **G1** = 2 : 1) (CD_3COCD_3, 400 MHz, 298 K, mixing time = 0.4 s).

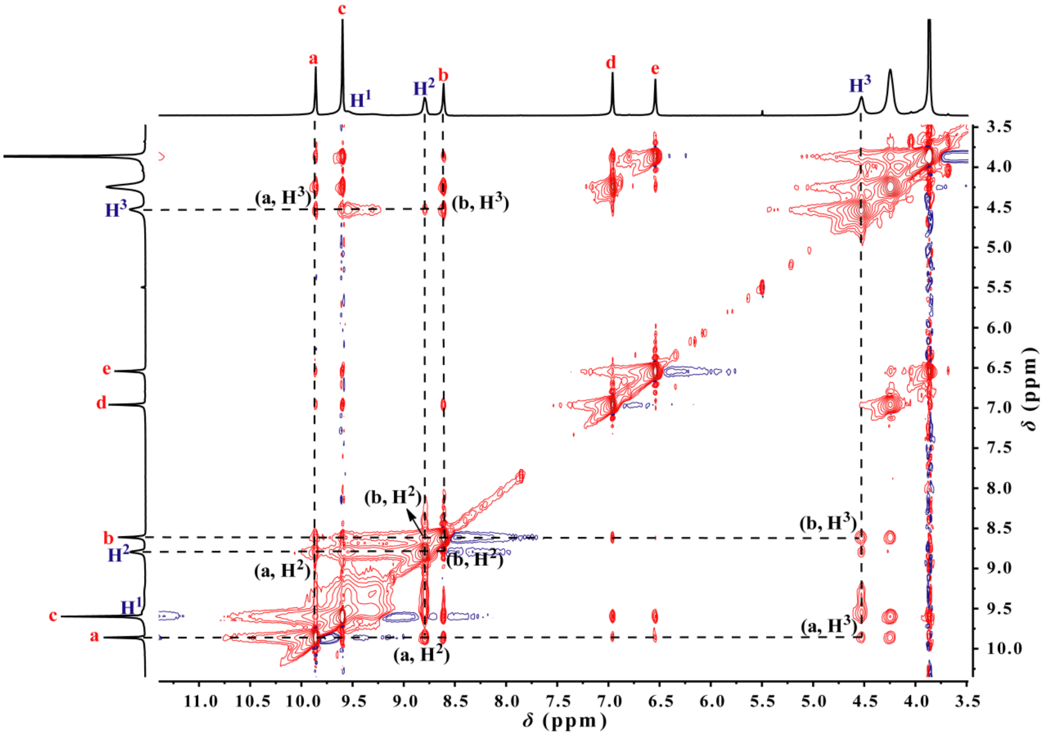

Figure S56 Expanded 2D-NOESY spectra of $\mathbf{1}_2 \supset$ **G1** ([**1**] = 10 mM, [**G1**] = 5 mM, **1** : **G1** = 2 : 1) (CD_3COCD_3, 400 MHz, 298 K, mixing time = 0.4 s).

2D-NOESY Spectra of $\mathbf{1}_2 \supset$ G2

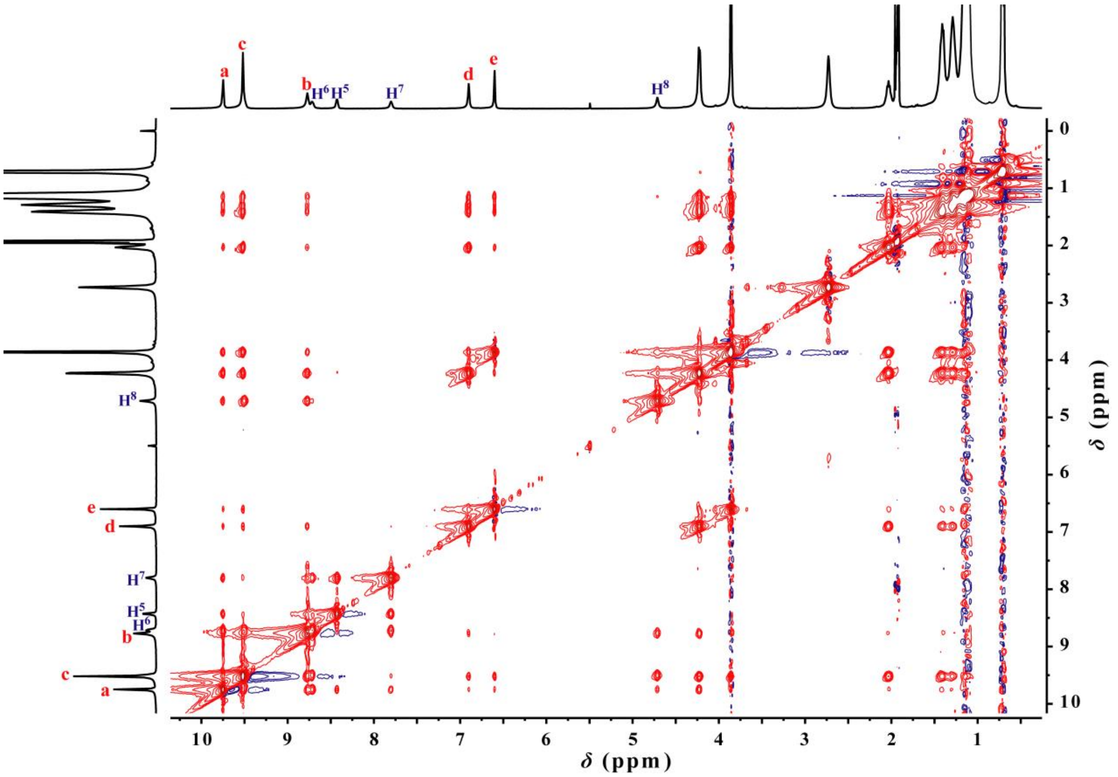

Figure S57 2D-NOESY spectra of $\mathbf{1}_2 \supset$ **G2** ([**1**] = 10 mM, [**G2**] = 5 mM, **1** : **G2** = 2 : 1) (CD_3COCD_3, 400 MHz, 298 K, mixing time = 0.4 s).

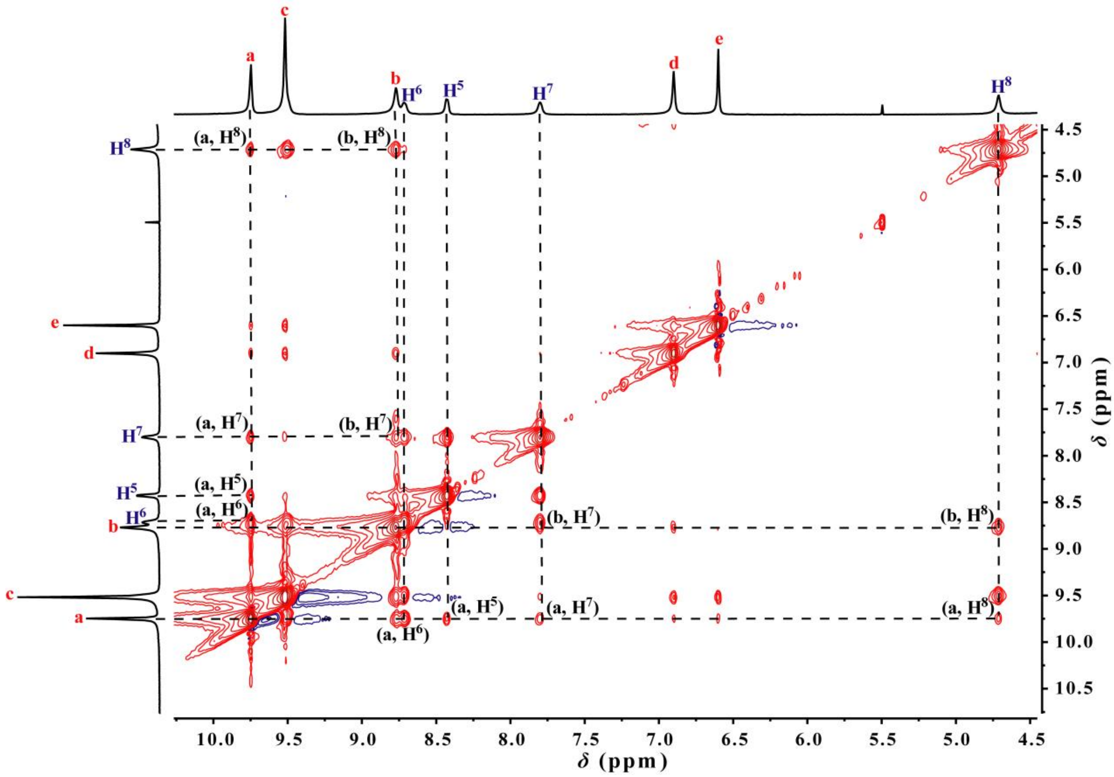

Figure S58 Expanded 2D-NOESY spectra of $\mathbf{1}_2 \supset$ **G2** ([**1**] = 10 mM, [**G2**] = 5 mM, **1** : **G2** = 2 : 1) (CD_3COCD_3, 400 MHz, 298 K, mixing time = 0.4 s).

2D-NOESY Spectra of 1 ⊃ G3

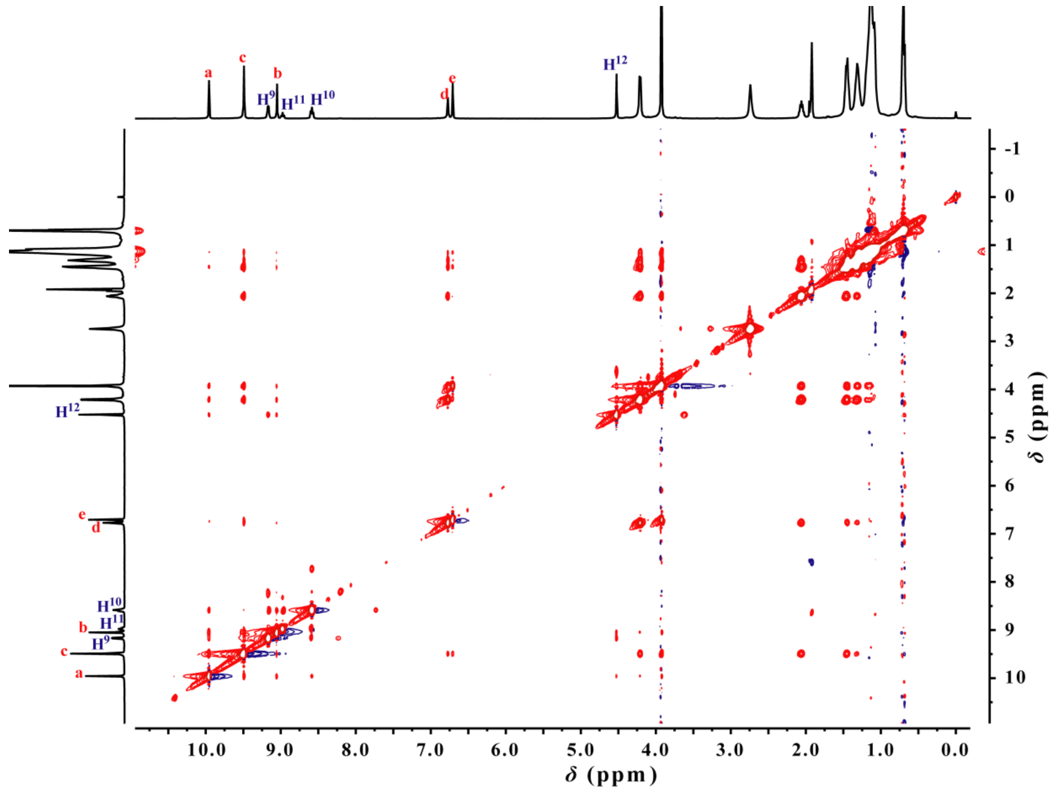

Figure S59 2D-NOESY spectra of **1** ⊃ **G3** ([**1**] = 10 mM, [**G3**] = 5 mM, **1** : **G3** = 2 :1) (CD_3COCD_3, 400 MHz, 298 K, mixing time = 0.4 s).

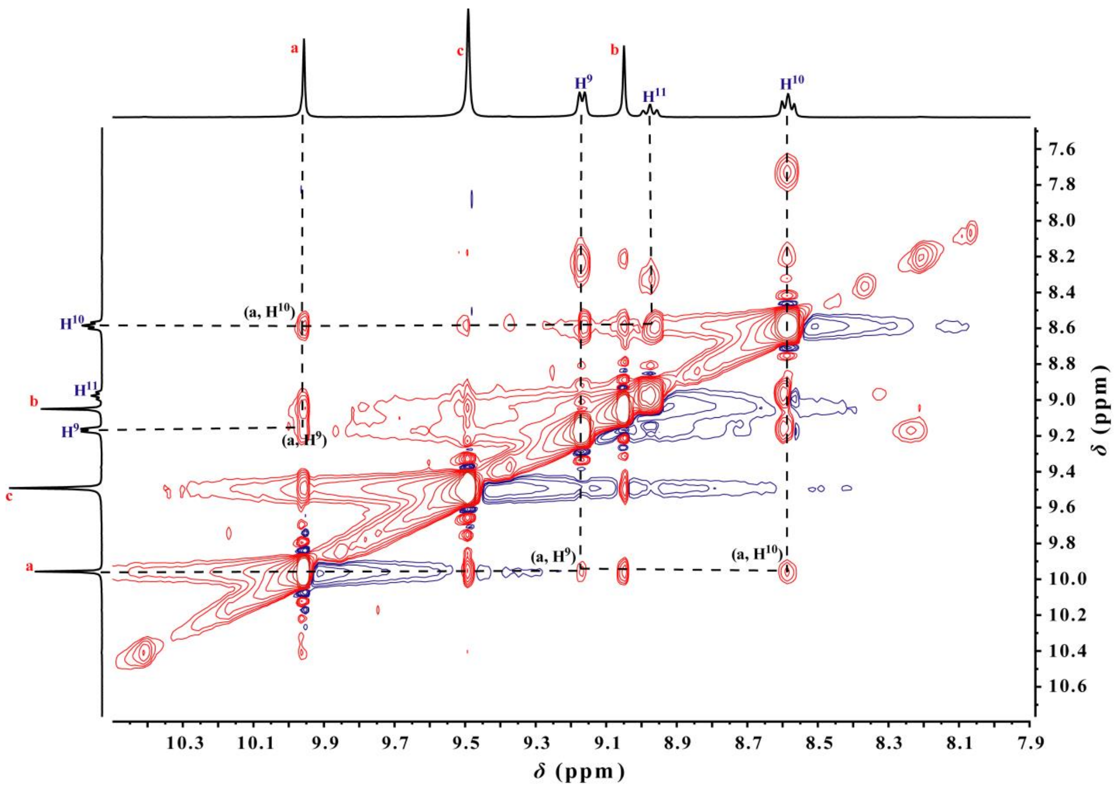

Figure S60 Expanded 2D-NOESY spectra of **1** ⊃ **G3** ([**1**] = 10 mM, [**G3**] = 5 mM, **1** : **G3** = 2 : 1) (CD_3COCD_3, 400 MHz, 298 K, mixing time = 0.4 s).

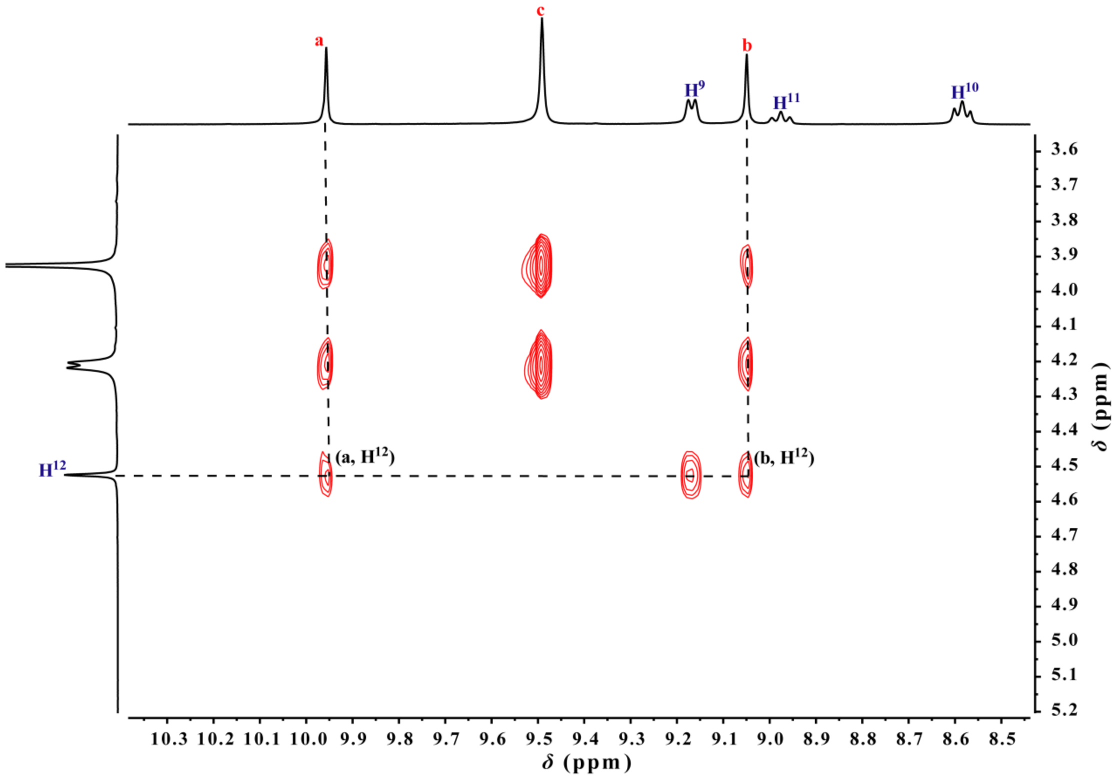

Figure S61 Expanded 2D-NOESY spectra of **1** ⊃ **G3** ([**1**] = 10 mM, [**G3**] = 5 mM, **1** : **G3** = 2 : 1) (CD_3COCD_3, 400 MHz, 298 K, mixing time = 0.4 s).

2D-NOESY Spectra of $1_2 \supset$ G4

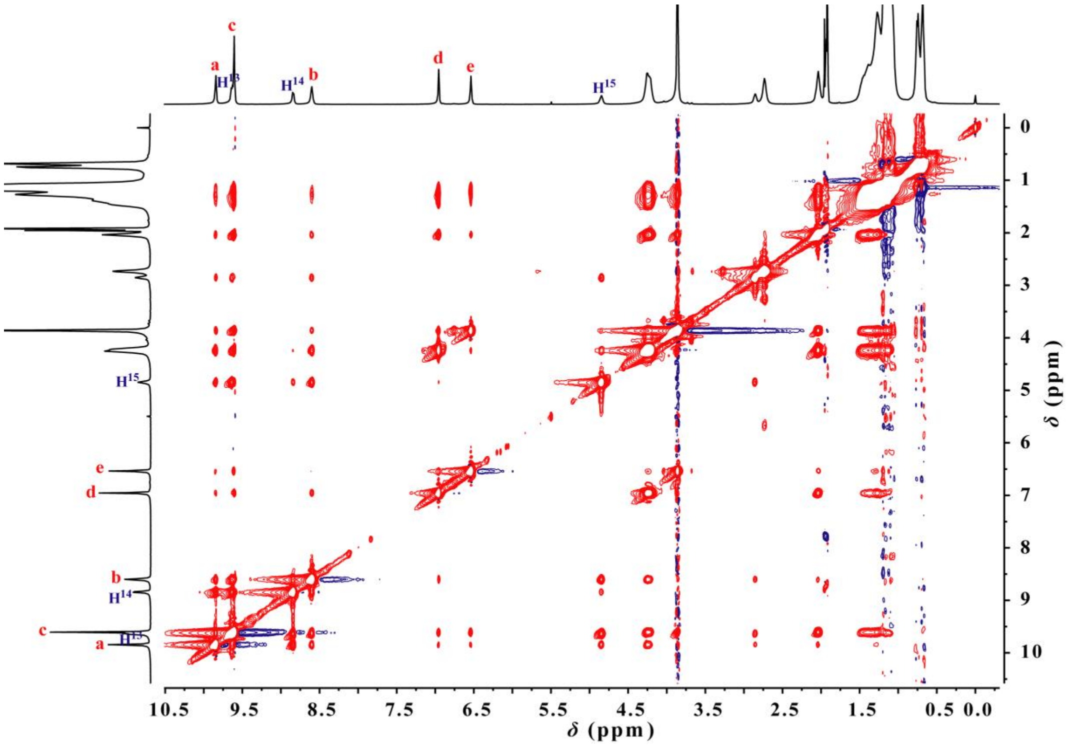

Figure S62 2D-NOESY spectra of $\mathbf{1}_2 \supset$ **G4** ([**1**] = 10 mM, [**G4**] = 5 mM, **1** : **G4** = 2 : 1) (CD_3COCD_3, 400 MHz, 298 K, mixing time = 0.4 s).

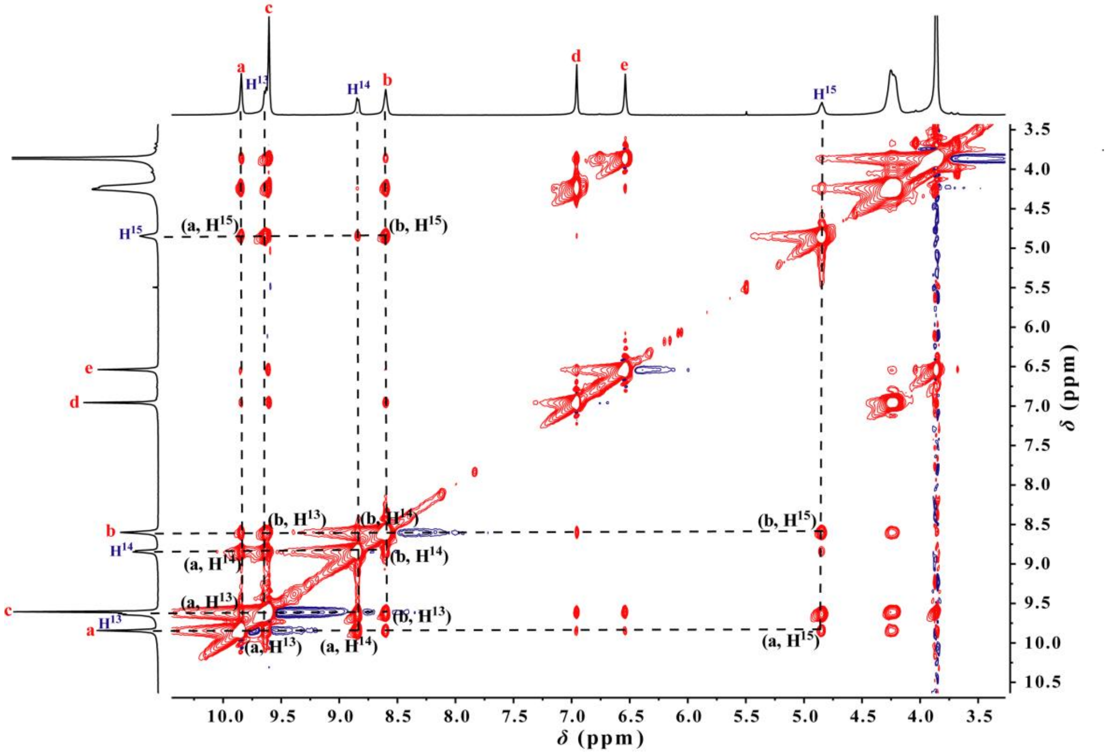

Figure S63 Expanded 2D-NOESY spectra of $\mathbf{1}_2 \supset$ **G4** ([**1**] = 10 mM, [**G4**] = 5 mM, **1** : **G4** = 2 : 1) (CD_3COCD_3, 400 MHz, 298 K, mixing time = 0.4 s).

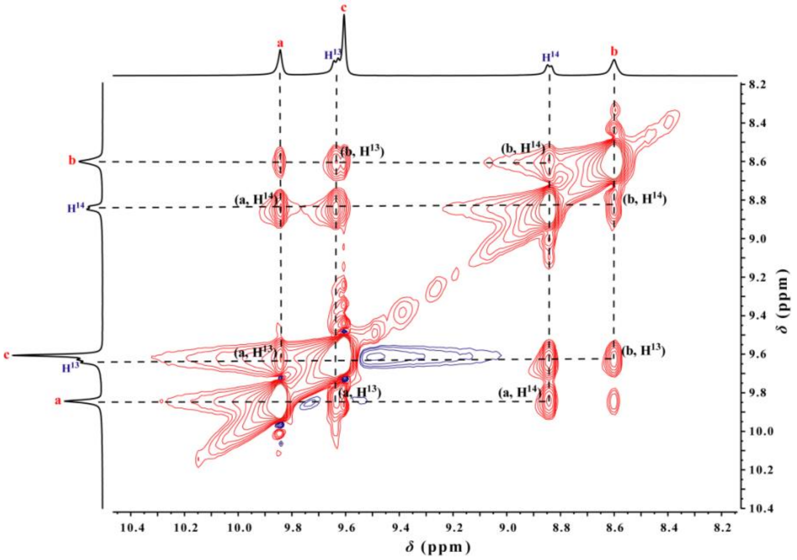

Figure S64 Expanded 2D-NOESY spectra of $\mathbf{1}_2 \supset$ **G4** ([**1**] =10 mM, [**G4**] = 5 mM, **1** : **G4** = 2 : 1) (CD_3COCD_3, 400 MHz, 298 K, mixing time = 0.4 s).

4.2.2 2D-DOSY Spectra of Host-Guest Complexes

2D-DOSY spectroscopic analyses provided evidence that a stable complex was formed between host cyclo[6]aramide **1** and pyridinium guests **G1**-**G4**. For the complexes $\mathbf{1}_2 \supset$ **G1,** $\mathbf{1}_2 \supset$ **G2** and $\mathbf{1}_2 \supset$ **G4,** all of the protons except the solvent, including those located on **1** and guests (**G1**, **G2** and **G4**), showed the same diffusion constants in the representative solution state mixtures. For the complex of **1** $\supset$ **G3**, all of the protons, including those located on **1** and **G3** showed the very similar diffusion constants in the representative solution state mixtures.

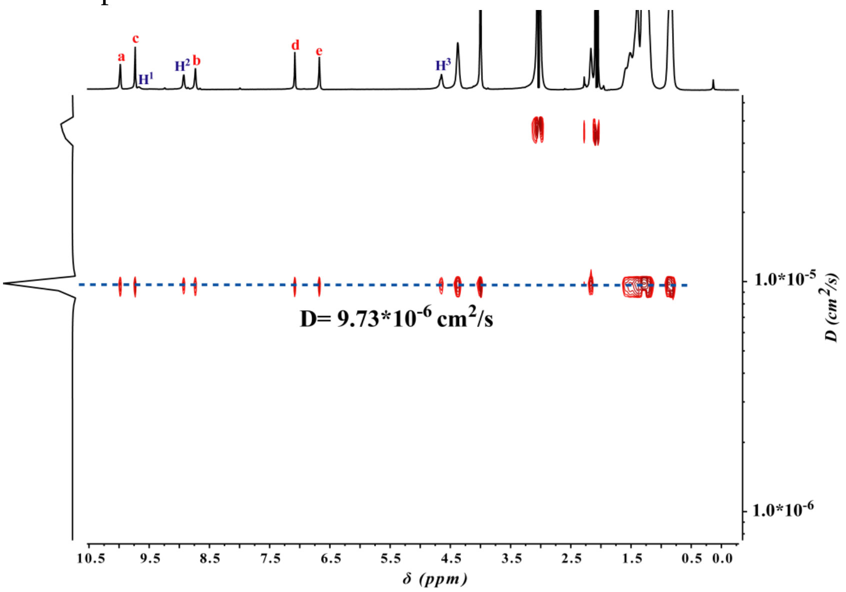

Figure S65 Expanded view of the 600 MHz 2D-DOSY NMR spectrum of **1** (10.0 mM) recorded in the presence of 1 molar equiv. of **G1** (5.0 mM) in CD_3COCD_3 at 298 K.

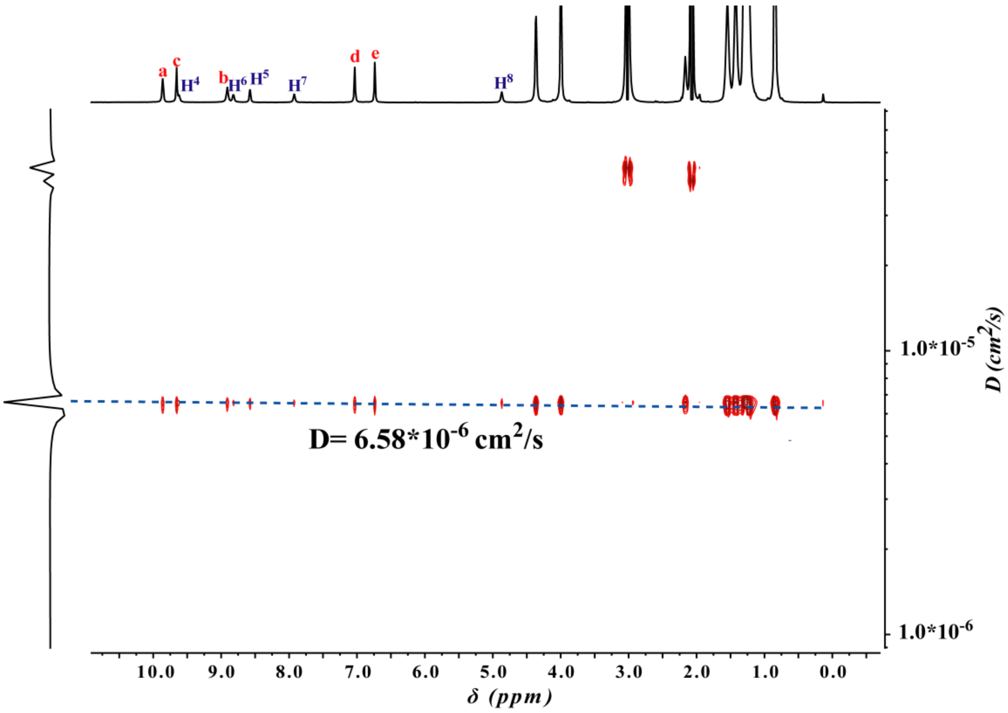

Figure S66 Expanded view of the 600 MHz 2D-DOSY NMR spectrum of **1** (10.0 mM) recorded in the presence of 1 molar equiv. of **G2** (5.0 mM) in CD_3COCD_3 at 298 K.

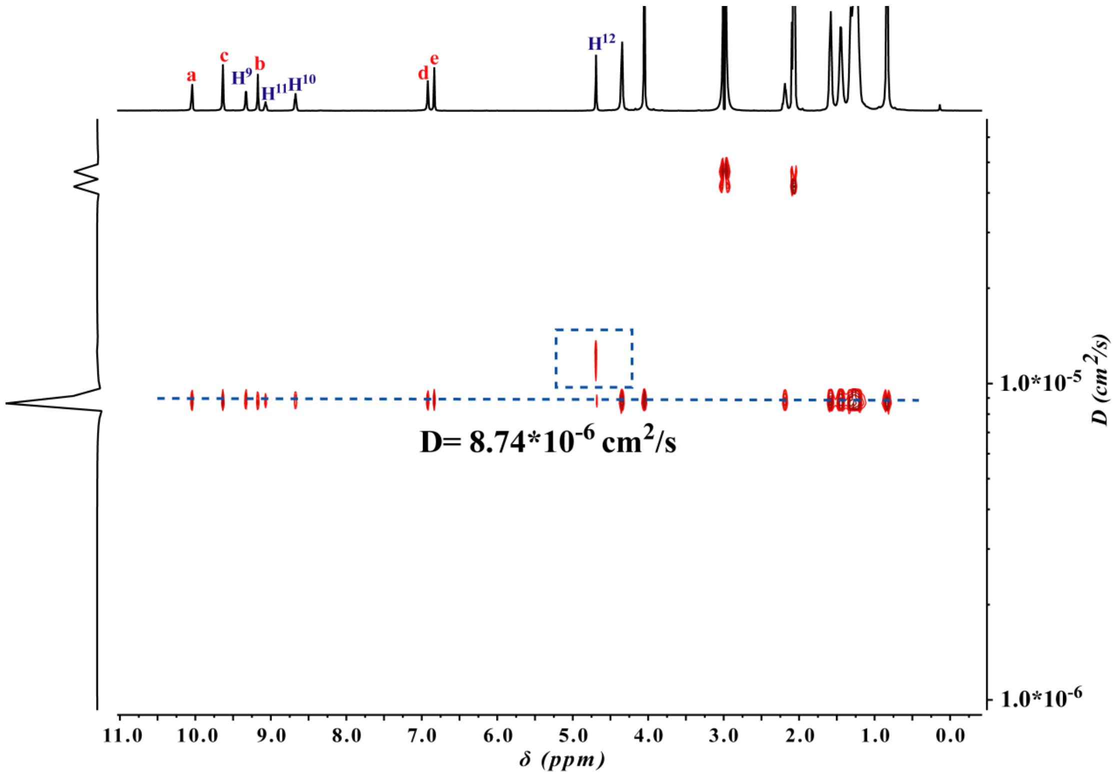

Figure S67 Expanded view of the 600 MHz 2D-DOSY NMR spectrum of **1** (10.0 mM) recorded in the presence of 1 molar equiv. of **G3** (10.0 mM) in CD_3COCD_3 at 298 K.

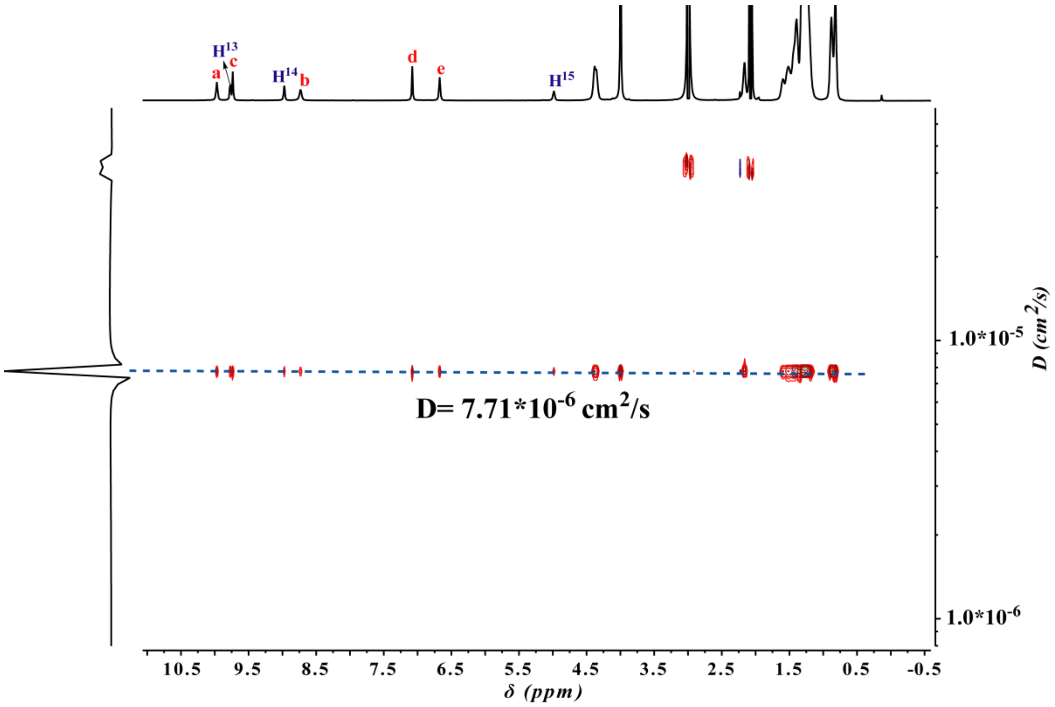

Figure S68 Expanded view of the 600 MHz 2D-DOSY NMR spectrum of **1** (10.0 mM) recorded in the presence of 1 molar equiv. of **G4** (5.0 mM) in CD_3COCD_3 at 298 K.

4.3 UV-vis Spectra of $1_2 \supset$ G1 and $1_2 \supset$ G4

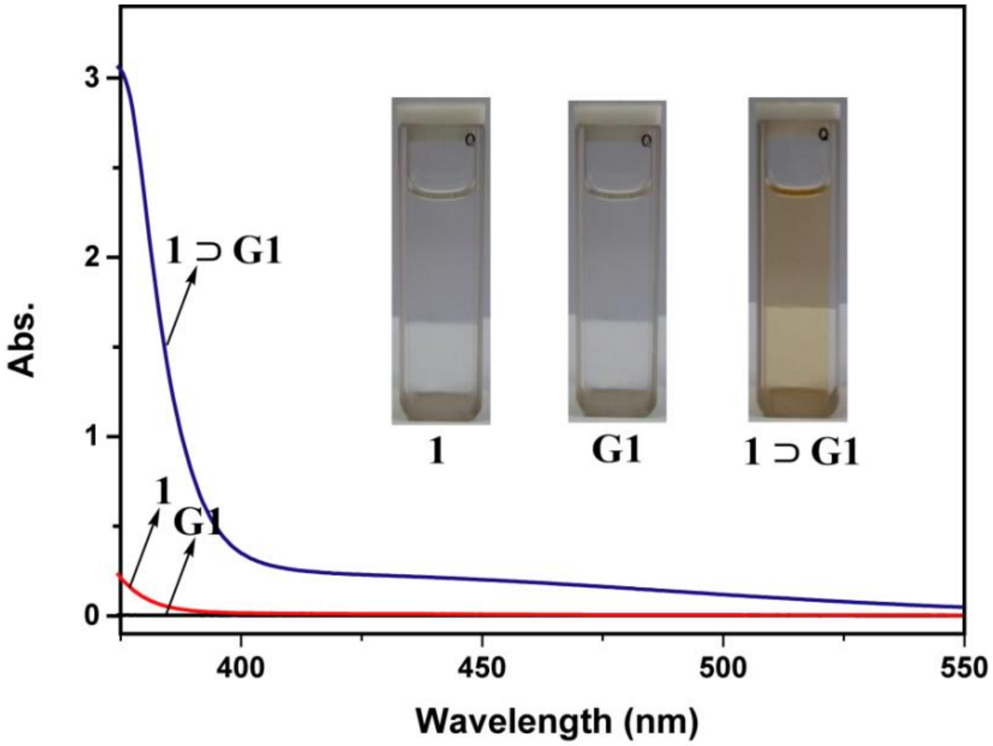

Figure S69 UV-vis spectra of **1**, **G1** and $\mathbf{1}_2 \supset$ **G1** (1 mM for each) in acetone. Inserted images show the color change.

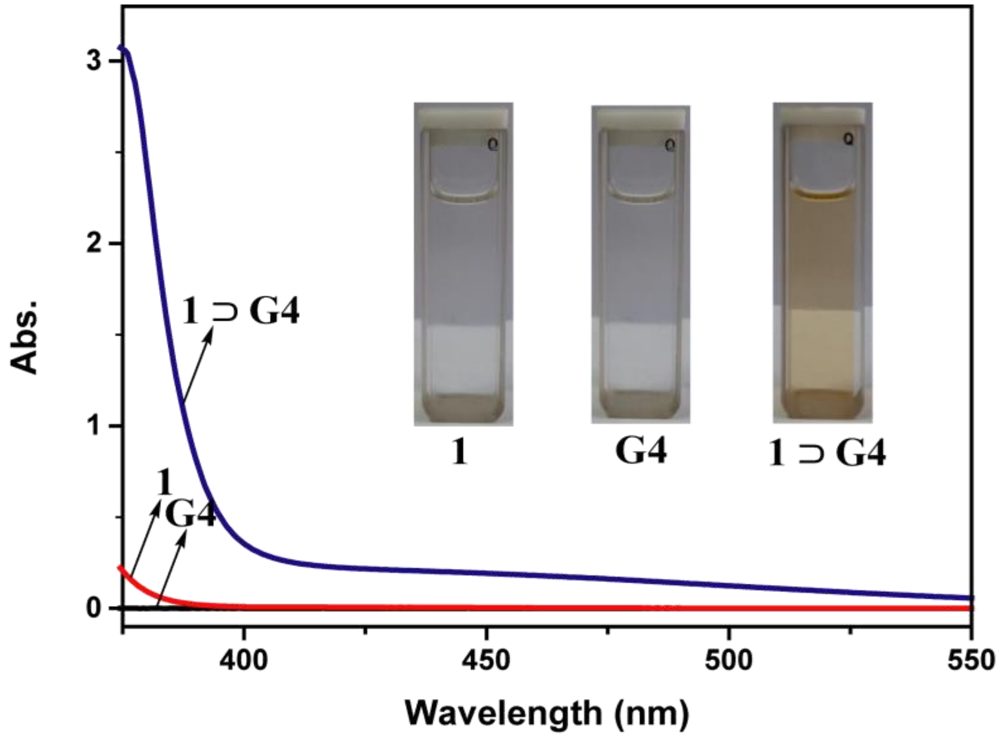

Figure S70 UV-vis spectra of **1**, **G4** and $\mathbf{1}_2 \supset$ **G4** (1 mM for each) in acetone. Inserted images show the color change.

4.4 Job Plots of Host-Guest Complexes

Job plots of $1_2 \supset$ G1

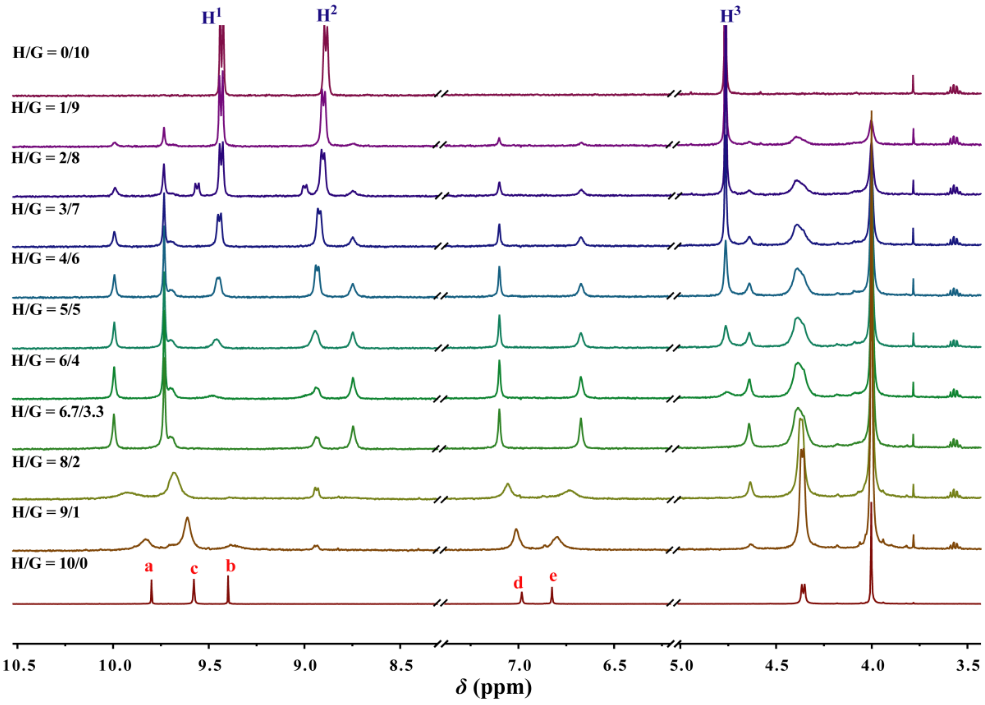

Figure S71 Partial stacked ^{1}H NMR spectra (400 MHz, 298 K, CD_3COCD_3) of $\mathbf{1}_2 \supset$ **G1** in the presence of the different ratio of **1** and **G1** at a fixed total concentration 1.0 mM.

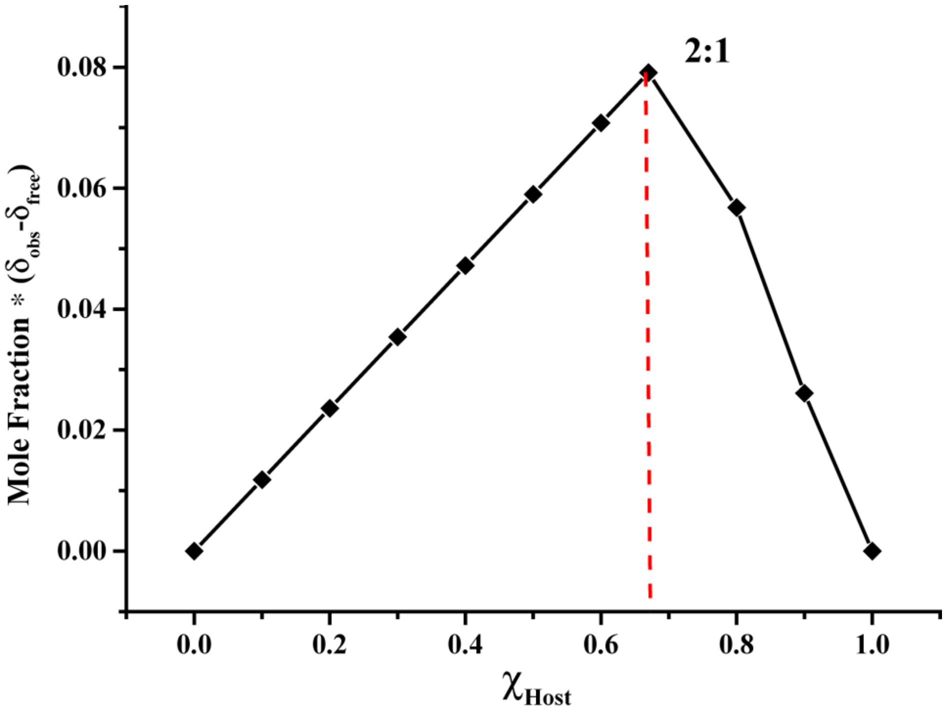

Figure S72 Job plots between $\mathbf{1}_2 \supset$ **G1** were obtained by plotting the chemical shift changes of the proton **a** (low-field signal) on cyclo[6]aramide **1** indicating a 2:1 stoichiometry.

Job plots of $\mathbf{1}_2 \supset$ G2

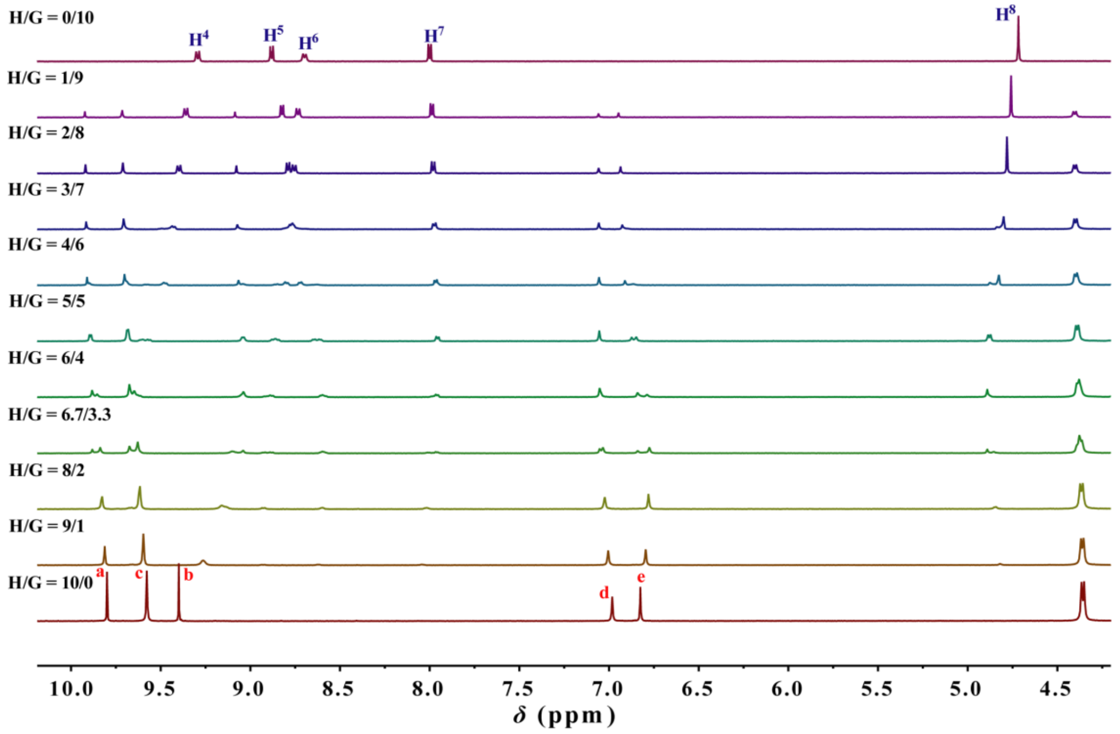

Figure S73 Partial stacked ^{1}H NMR spectra (400 MHz, 298 K, CD_3COCD_3) of $\mathbf{1}_2 \supset$ **G2** in the presence of the different ratio of **1** and **G2** at a fixed total concentration 1.0 mM.

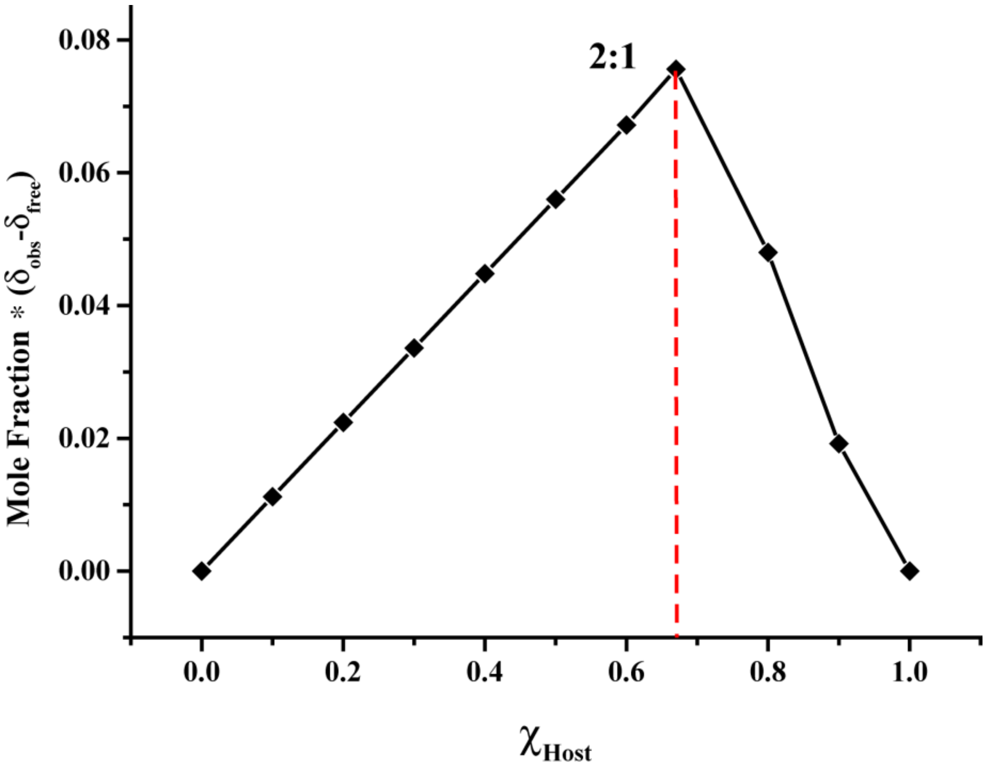

Figure S74 Job plots between $\mathbf{1}_2 \supset$ **G2** were obtained by plotting the chemical shift changes of the proton **d** (low-field signal) on cyclo[6]aramide **1** indicating a 2:1 stoichiometry.

Job plots of 1 ⊃ G3

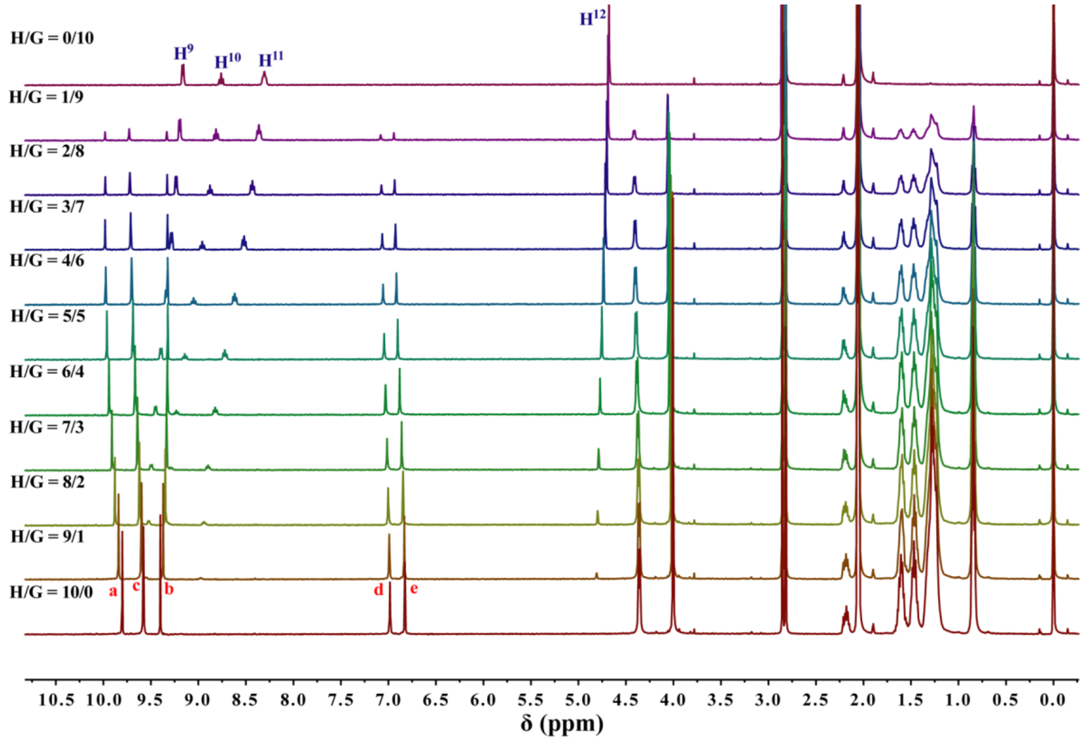

Figure S75 Partial stacked ^{1}H NMR spectra (400 MHz, 298 K, CD_3COCD_3) of **1** ⊃ **G3** in the presence of the different ratio of **1** and **G3** at a fixed total concentration 1.0 mM.

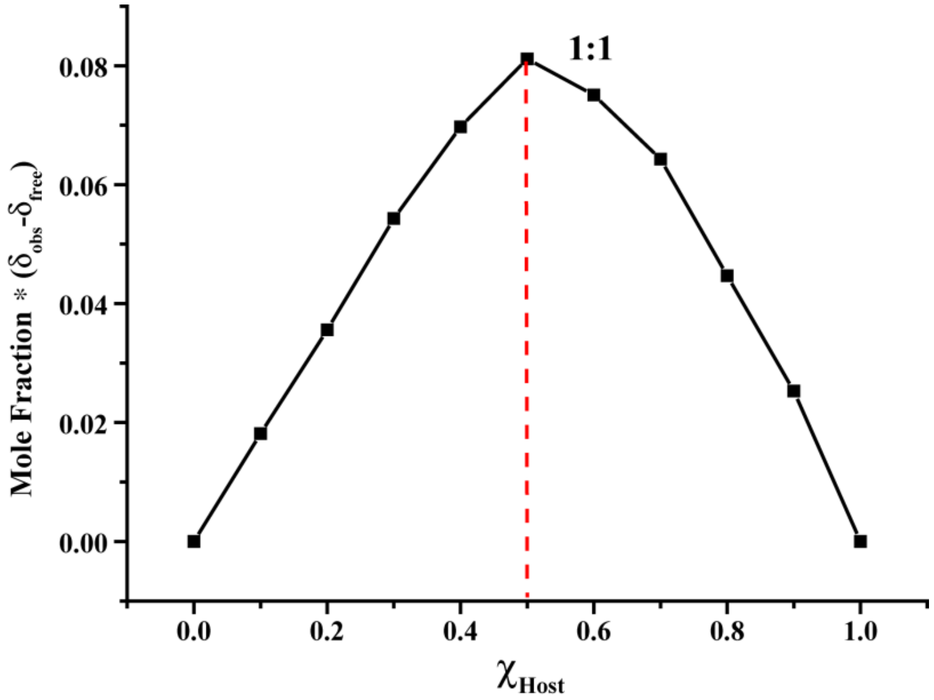

Figure S76 Job plots between **1** ⊃ **G3** were obtained by plotting the chemical shift changes of the proton **a** on cyclo[6]aramide **1** indicating a 1:1 stoichiometry.

Job plots of 1_2 ⊃ G4

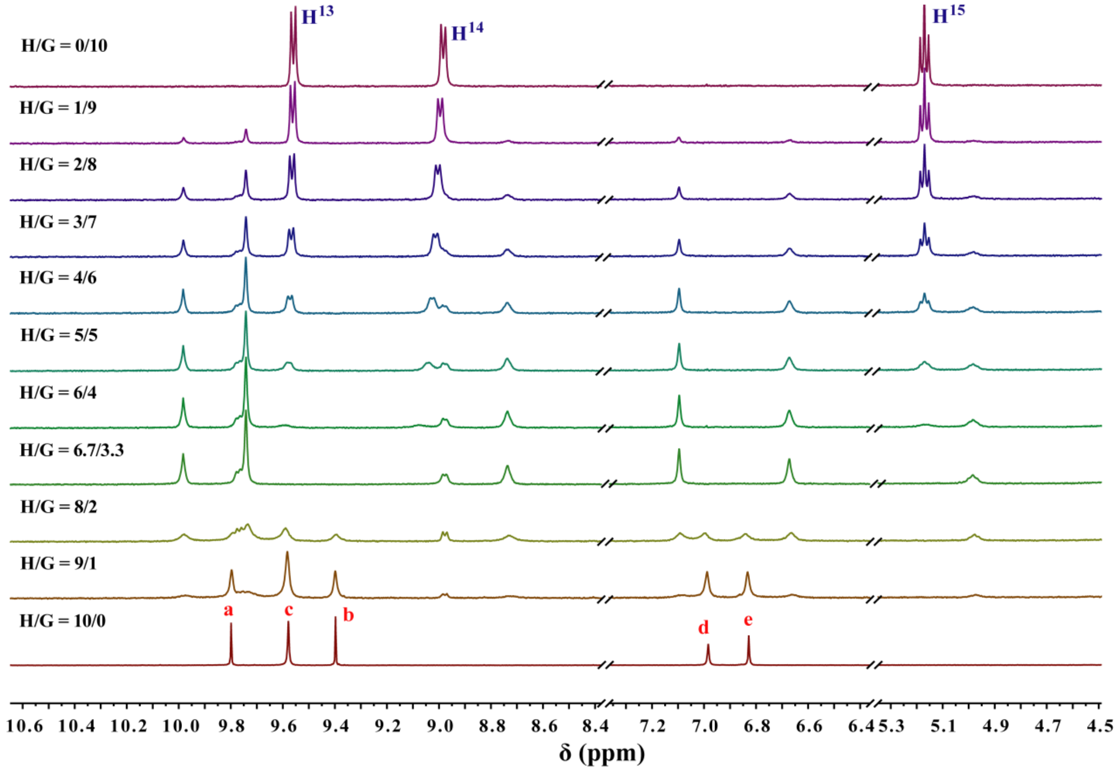

Figure S77 Partial stacked ^{1}H NMR spectra (400 MHz, 298 K, CD_3COCD_3) of $\mathbf{1}_2$ ⊃ **G4** in the presence of the different ratio of **1** and **G4** at a fixed total concentration 1.0 mM.

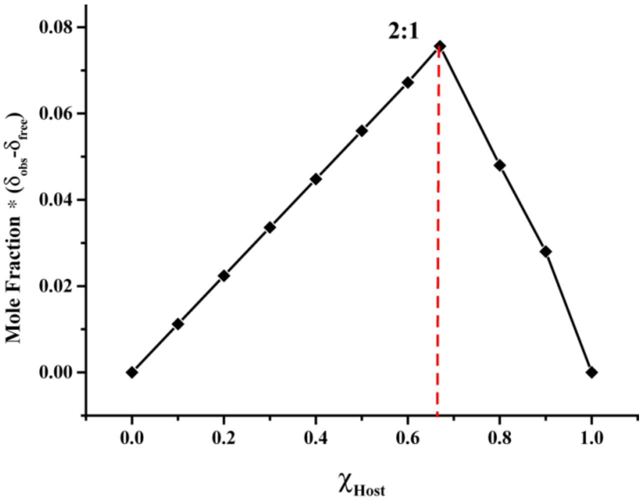

Figure S78 Job plots between $\mathbf{1}_2 \supset$ **G4** were obtained by plotting the chemical shift changes of the proton **d** on cyclo[6]aramide **1** indicating a 2:1 stoichiometry.

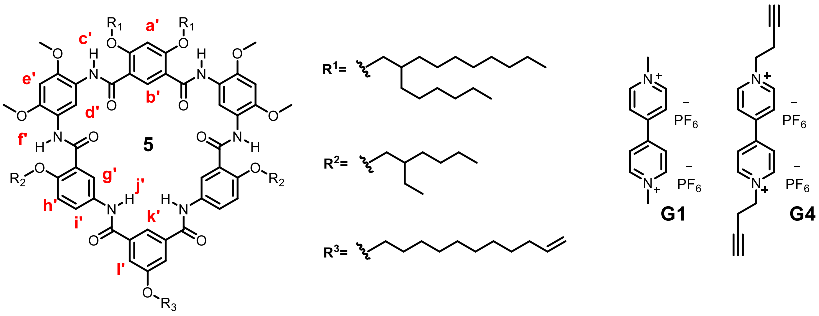

Job plots of $\mathbf{5}_2 \supset$ G1

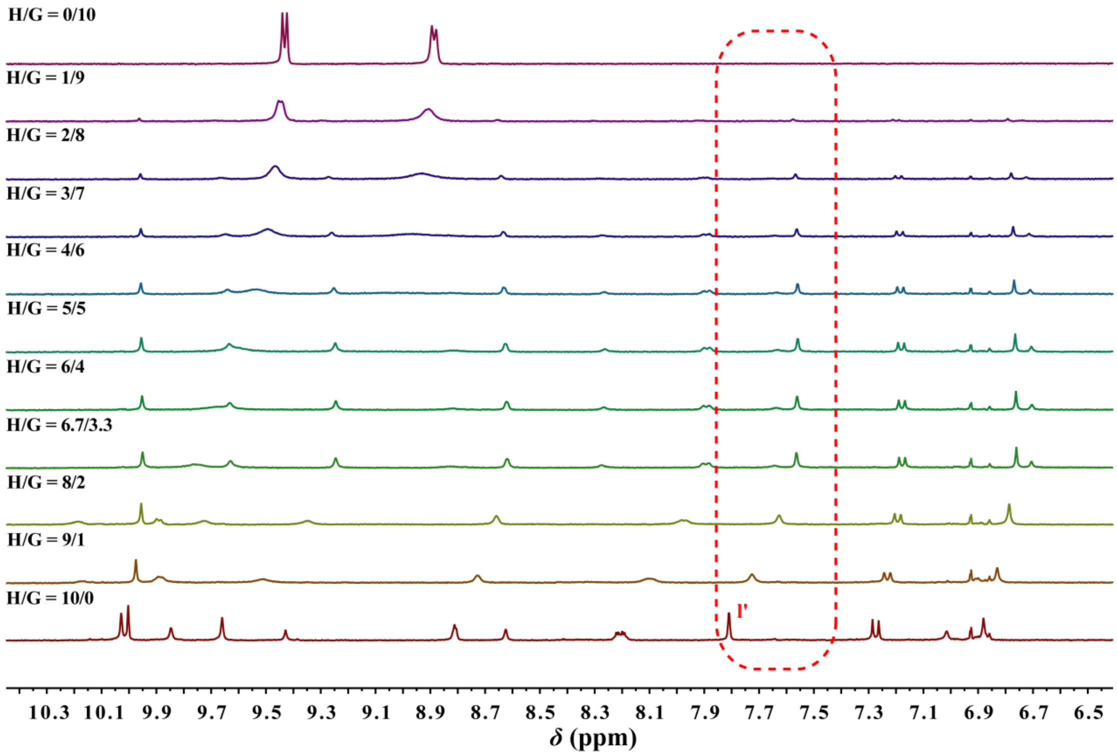

Figure S79 Partial stacked ^{1}H NMR spectra (400 MHz, 298 K, CD_3COCD_3) of $\mathbf{5}_2 \supset$ **G1** in the

presence of the different ratio of **5** and **G1** at a fixed total concentration 1.0 mM.

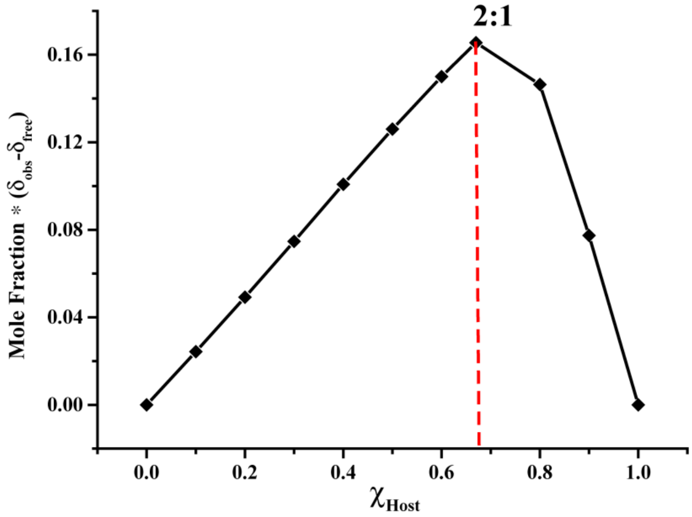

Figure S80 Job plots between $5_2 \supset$ **G1** were obtained by plotting the chemical shift changes of the proton **l'** on heteroditopic cyclo[6]aramide **5** indicating a 2:1 stoichiometry.

Job plots of $5_2 \supset$ G4

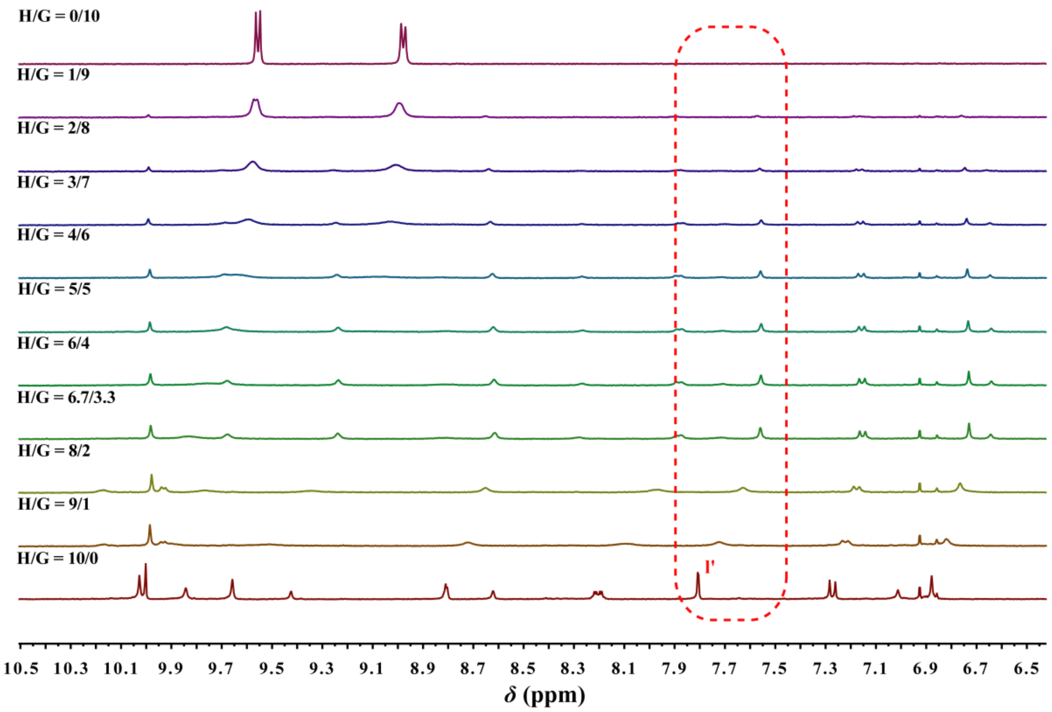

Figure S81 Partial stacked ^{1}H NMR spectra (400 MHz, 298 K, CD_3COCD_3) of $5_2 \supset$ **G4** in the presence of the different ratio of **5** and **G4** at a fixed total concentration 1.0 mM.

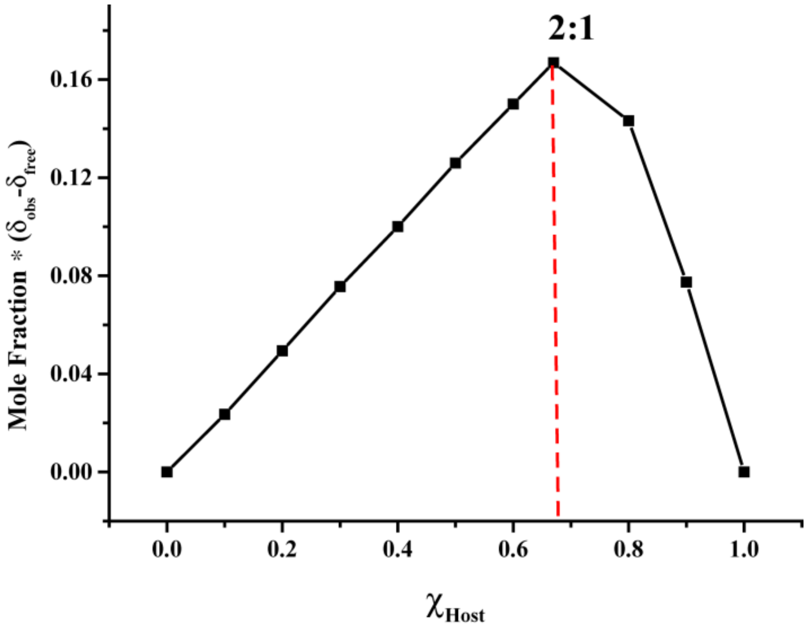

Figure S82 Job plots between **5**$_2$ ⊃ **G4** were obtained by plotting the chemical shift changes of the proton **l'** on heteroditopic cyclo[6]aramide **5** indicating a 2:1 stoichiometry.

4.5 Determination of the Stoichiometries and Binding Constants

To determine the binding constants (K_a) for cyclo[6]aramide **1** binding with guests **G1-G4**, UV-vis titration experiments were done with CH_3COCH_3 solutions which had a constant concentration of cyclo[6]aramide **1** and varying concentration of guests **G1-G4**. For each titration, at least 30 data points were collected. Typically wavelength was monitored around the absorption maxima for the complex formed. This gave data sets from which the binding constants were obtained using a custom written global nonlinear regression analysis program within the Matlab 8.1 by Thordarson[1].

From the job plot and molar ratio plot, 2:1 stoichiometries were obtained for **1**$_2$ ⊃ **G1, 1**$_2$ ⊃ **G2 and 1**$_2$ ⊃ **G4**. The binding constants (K_1 and K_2) were estimated by a non-linear curve-fitting method with the equation **S-1**:[1]

$$\Delta A = \frac{[G]_0 (\varepsilon_{\Delta HG} K_1 [H] + 2\varepsilon_{\Delta H_2G} K_1 K_2 [H]^2)}{1 + K_1[H] + K_1 K_2 [H]^2} \qquad \textbf{S-1}$$

$$\mathbf{H + G \overset{K_1}{\rightleftharpoons} HG \qquad HG + H \overset{K_2}{\rightleftharpoons} H_2G}$$

$$K_1 = \frac{[HG]}{[H][G]} \qquad K_2 = \frac{[H_2G]}{[HG][H]} = \frac{[H_2G]}{K_1[H]^2[G]}$$

$$[H]_0 = [H] + [HG] + 2[H_2G] \qquad [G]_0 = [G] + [HG] + [H_2G]$$

$$K_1K_2[H]^3 + (K_1 + 2K_1K_2[G]_0 - K_1K_2[H]_0)[H]^2 + (K_1[G]_0 - K_1[H]_0 + 1)[H] - [H]_0 = 0$$

(S-2)

This cubic equation **S-2** is solved directly in Matlab3 and the results put into equation **S-1**.

Where $[G]_0$ is the concentration of guests (**G1**, **G2** and **G4**), $[H]_0$ is the concentration of cyclo[6]aramide **1**, ΔA is the absorption change of the complex formed, $\varepsilon_{\Delta HG}$ is the molar extinction coefficient of 1 to 1 host-guest complex, $\varepsilon_{\Delta H_2G}$ is the molar extinction coefficient of 2 to 1 host-guest complex.

UV-vis titration experiments of $1_2 \supset$ G1

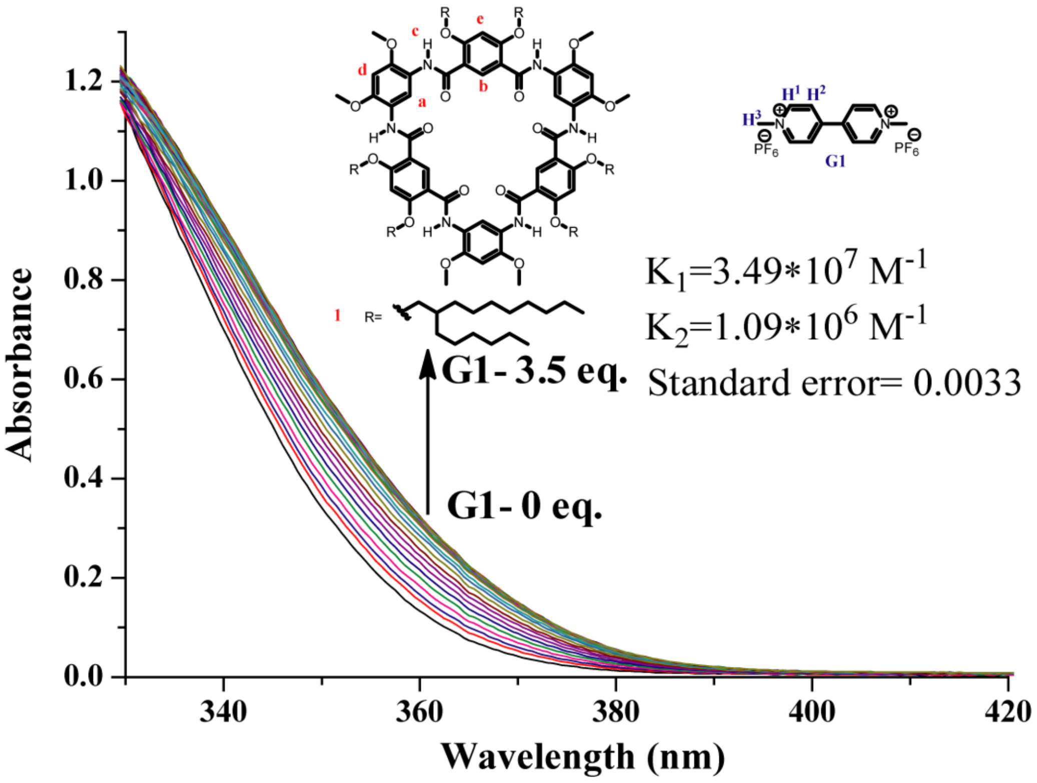

Figure S83 Stacked UV-vis spectra of **1**(20 μM) titrated with **G1** in acetone from 0 equiv. to 3.5 equiv. at 298 K.

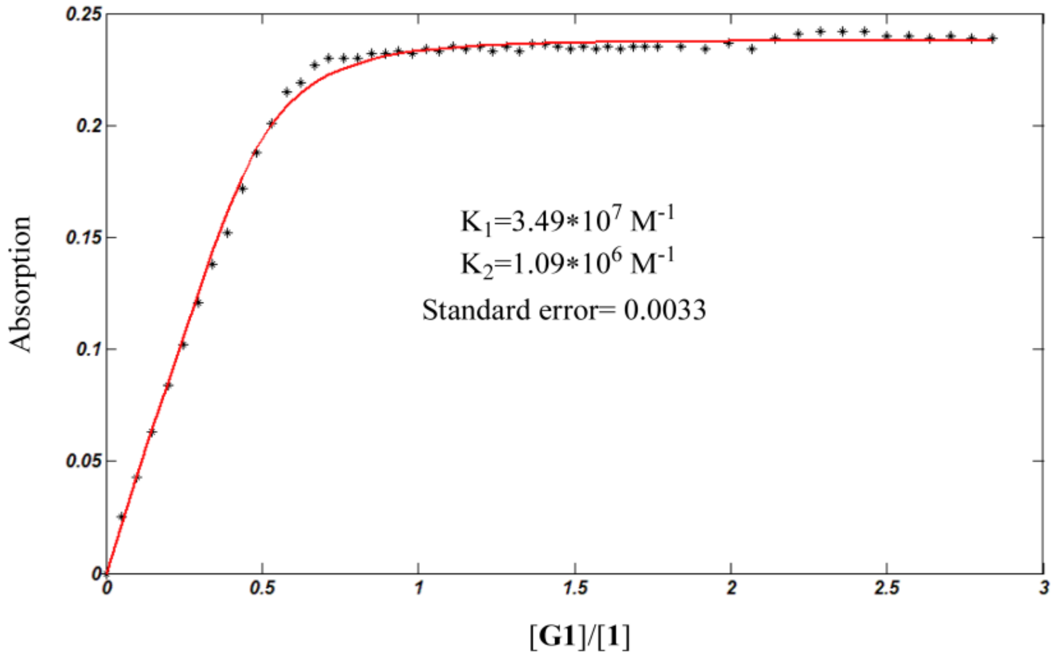

Figure S84 The change of absorption of **1** titrated with **G1** at 352 nm in acetone. The red solid line was obtained from the non-linear curve-fitting with **Eq. S-1.**

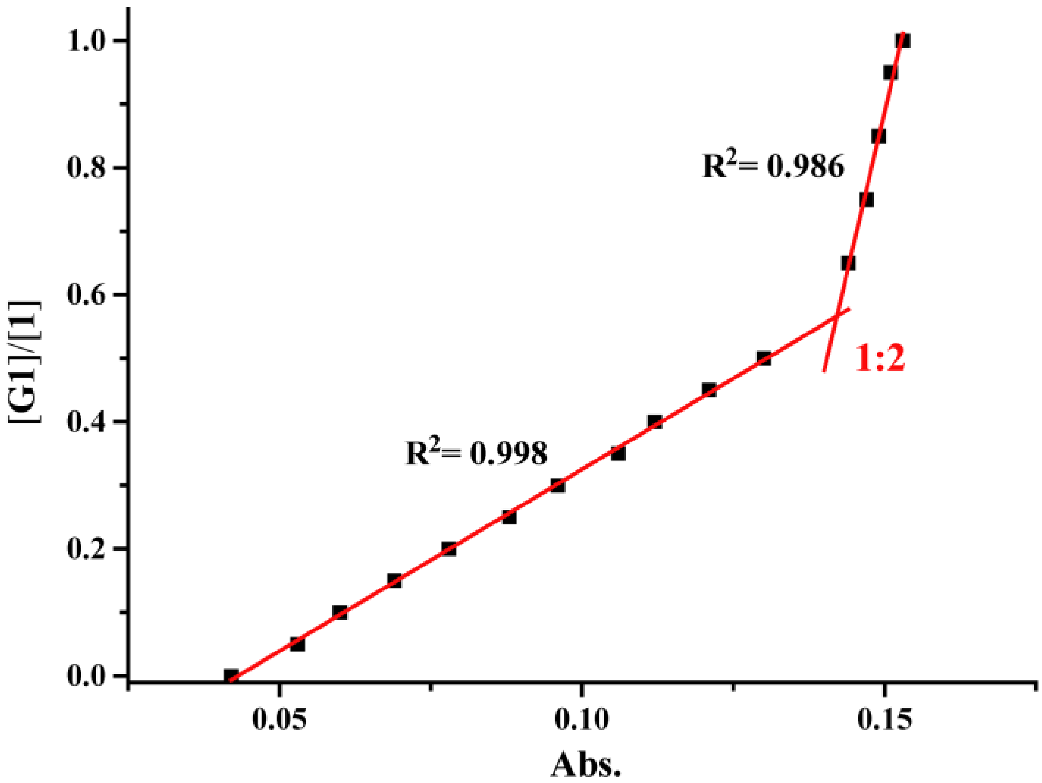

Figure S85 Mole ratio plot for the complexation of **1** and **G1** in acetone indicating a 2:1 stoichiometry at 352 nm in acetone.

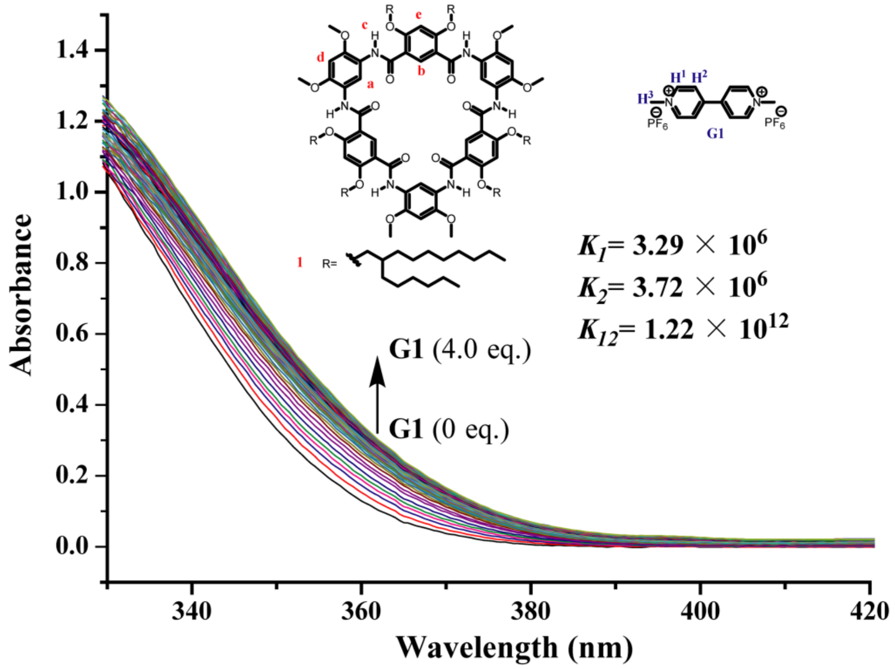

Figure S86 Stacked UV-vis spectra of **1**(20 µM**)** titrated with **G1** in acetone/DMSO (9/1, v/v) from 0 equiv. to 4.0 equiv. at 298 K.

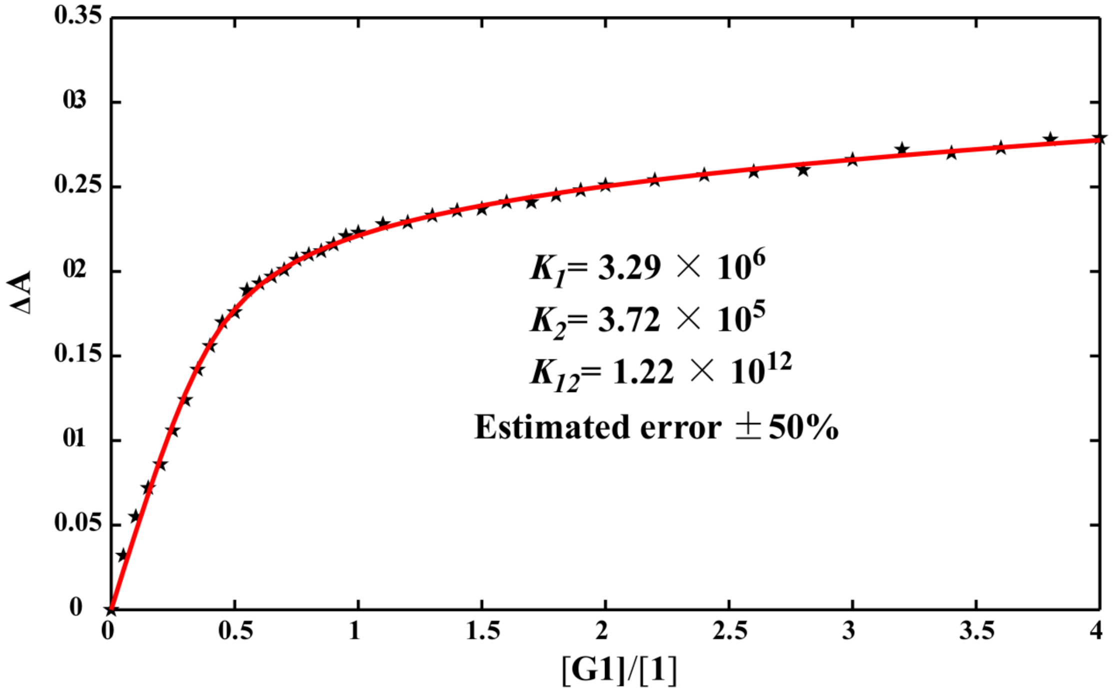

Figure S87 The change of absorption of **1** titrated with **G1** at 353 nm in acetone/DMSO (9/1, v/v). The red solid line was obtained from the non-linear curve-fitting with **Eq. S-1.**

UV-vis titration experiments of $1_2 \supset$ G2

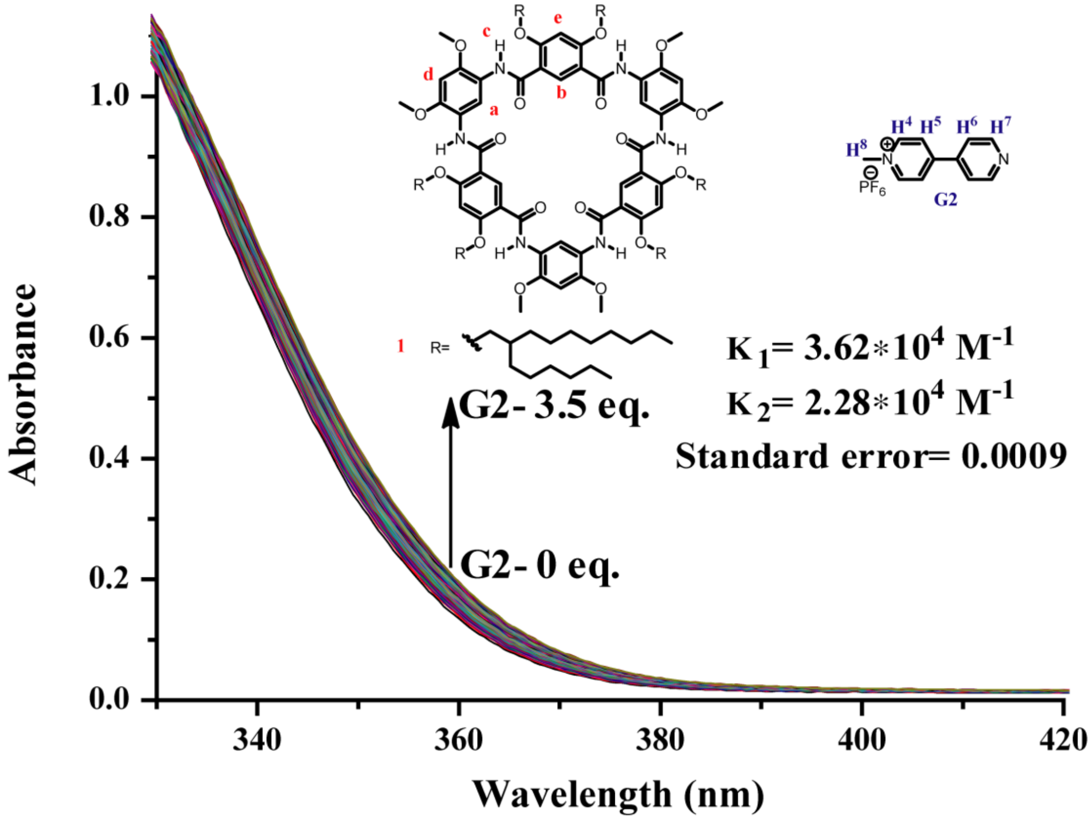

Figure S88 Stacked UV-vis spectra of **1**(20 μM**)** titrated with **G2** in acetone from 0 equiv. to 3.5 equiv. at 298 K.

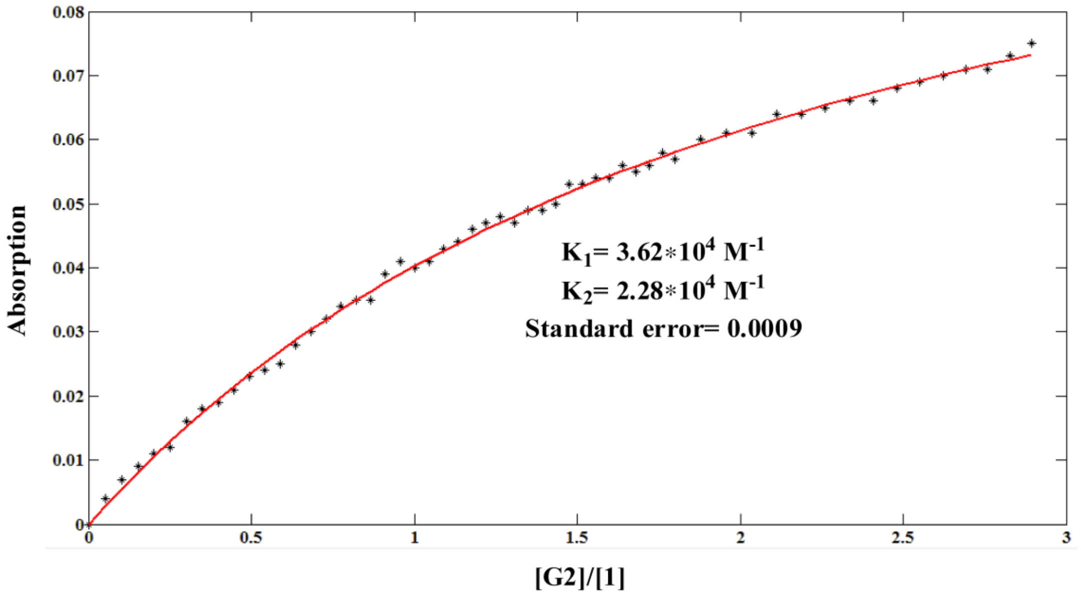

Figure S89 The change of absorption of **1** titrated with **G2** at 355 nm in acetone. The red solid line was obtained from the non-linear curve-fitting with **Eq. S-1.**

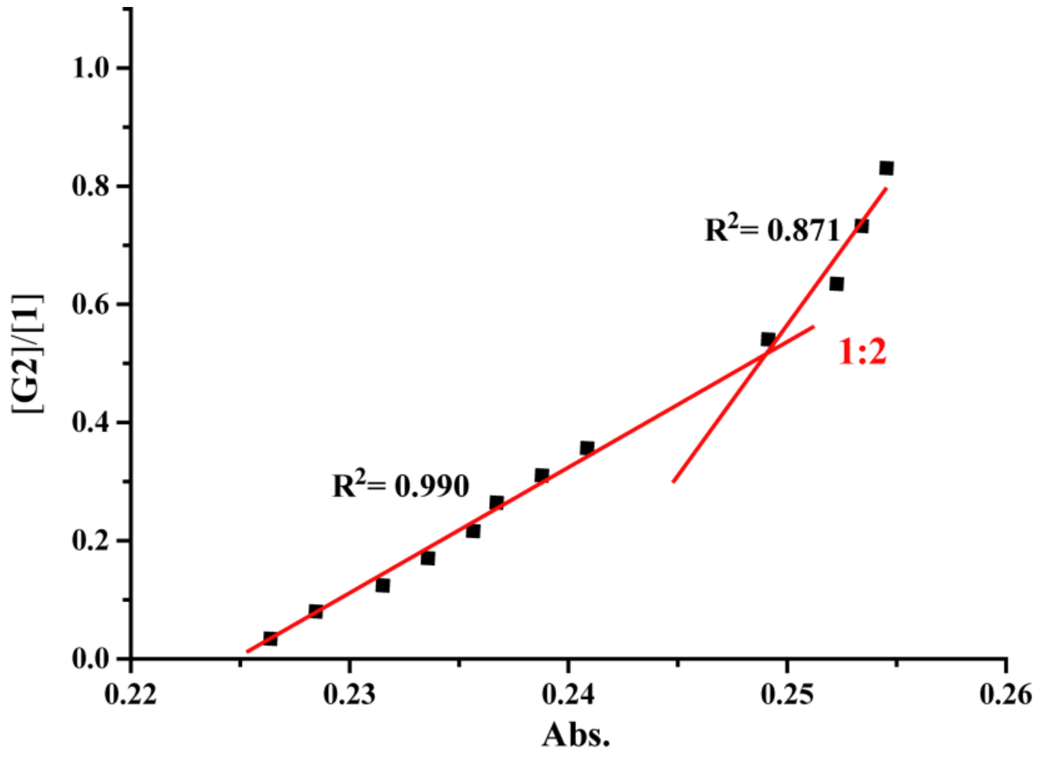

Figure S90 Mole ratio plot for the complexation of **1** and **G2** in acetone indicating a 2:1 stoichiometry at 355 nm in acetone.

UV-vis titration experiments of $1_2 \supset$ G4

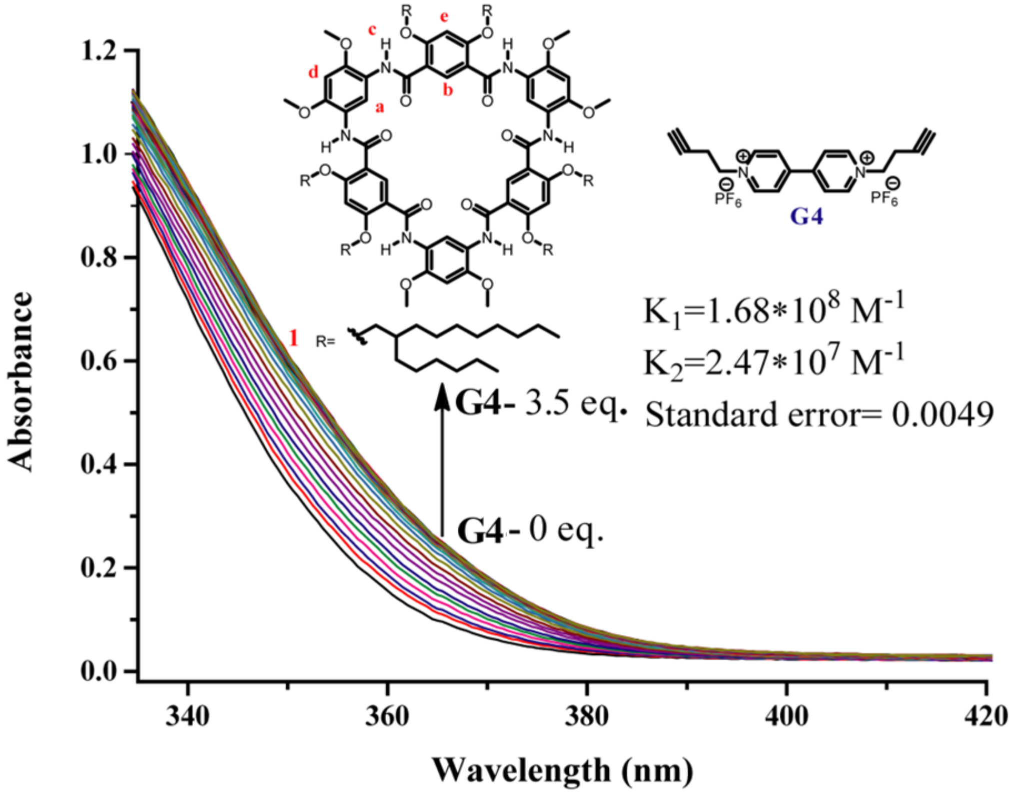

Figure S91 Stacked UV-vis spectra of **1**(20 μM**)** titrated with **G4** in acetone from 0 equiv. to 3.0 equiv. at 298 K.

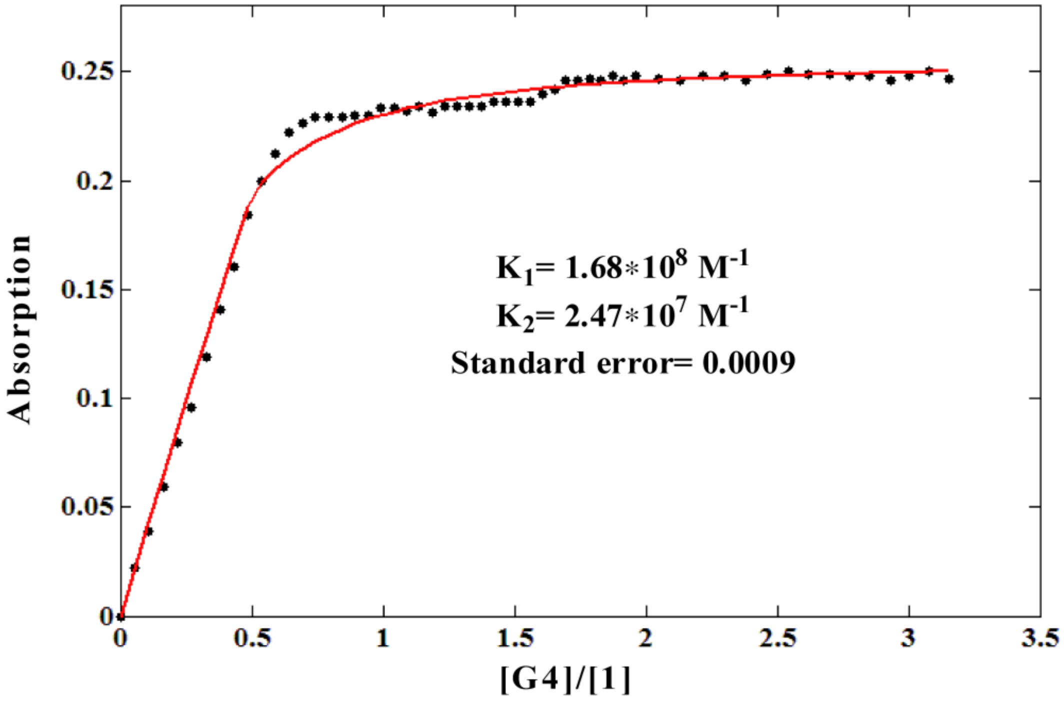

Figure S92 The change of absorption of **1** titrated with **G4** at 350 nm in acetone. The red solid line was obtained from the non-linear curve-fitting with **Eq. S-1.**

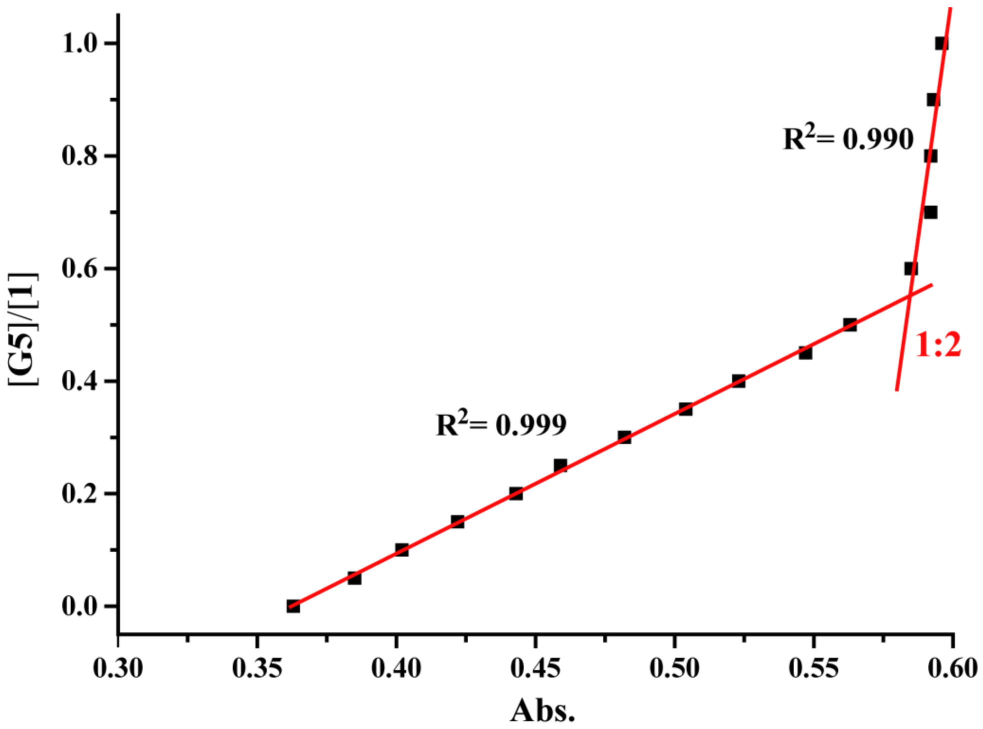

Figure S93 Mole ratio plot for the complexation of **1** and **G4** in acetone indicating a 2:1 stoichiometry at 350 nm in acetone.

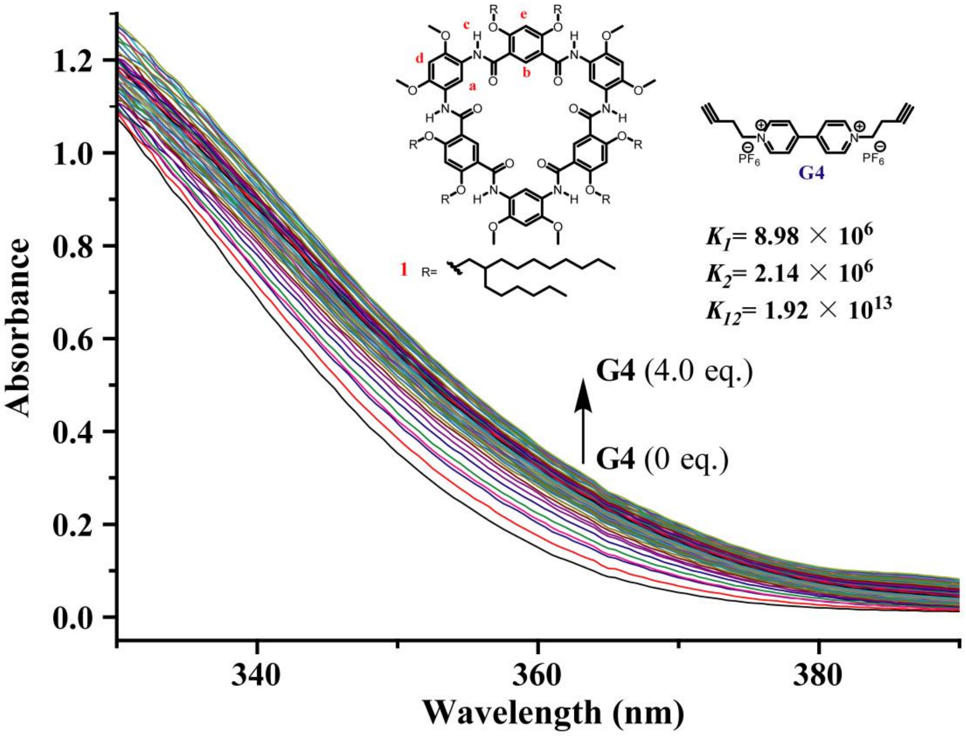

Figure S94 Stacked UV-vis spectra of **1**(20 μM**)** titrated with **G4** in acetone/DMSO (9/1, v/v) from 0 equiv. to 4.0 equiv. at 298 K.

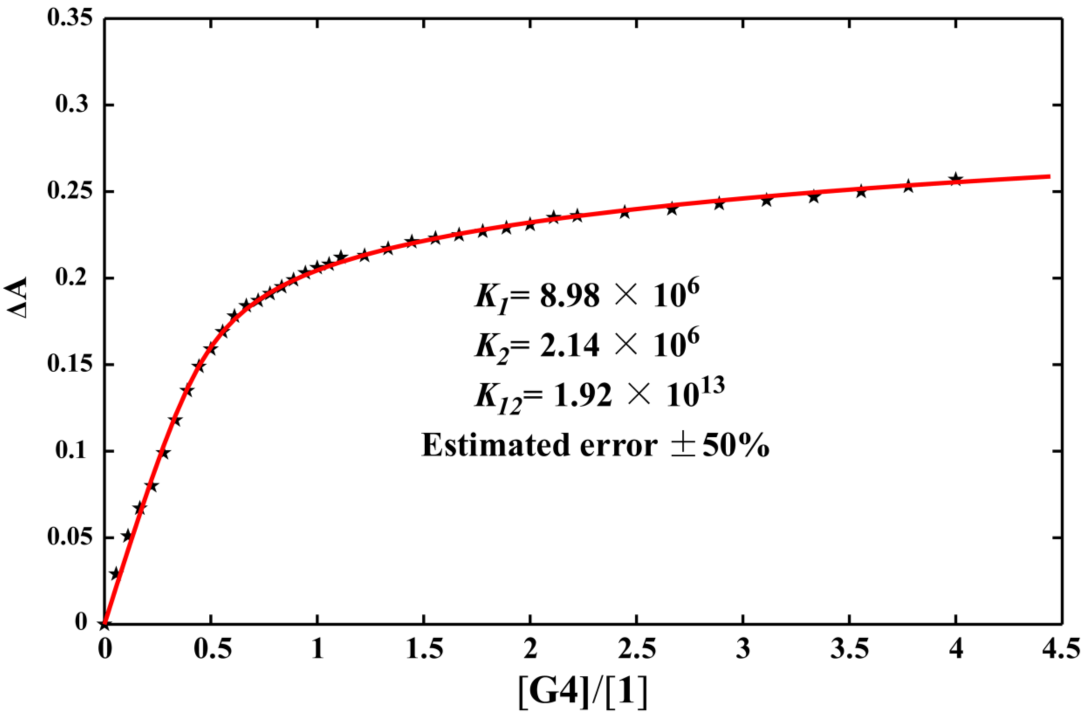

Figure S95 The change of absorption of **1** titrated with **G4** at 354 nm in acetone. The red solid line was obtained from the non-linear curve-fitting with **Eq. S-1.**

From the job plot and molar ratio plot, a 1:1 stoichiometry was obtained for **1** ⊃ **G3**.

The binding constant (K_a) was estimated by a non-linear curve-fitting method with the equation **S-3** by UV-vis titration experiments:[1]

$$\Delta A = \varepsilon_{\Delta HG} (0.5\{([G]_0+[H]_0 + 1/K_a)-\{([G]_0+[H]_0+1/K_a)^2+4[H]_0[G]_0\}^{0.5}\}) \qquad \textbf{Eq. S-3}$$

Where $[G]_0$ is the concentration of **G3**, $[H]_0$ is the concentration of cyclo[6]aramide **1**, ΔA is the absorption change of the complex formed, $\varepsilon_{\Delta HG}$ is the molar extinction coefficient of 1 to 1 host-guest complex.

UV-vis titration experiments of 1 ⊃ G3

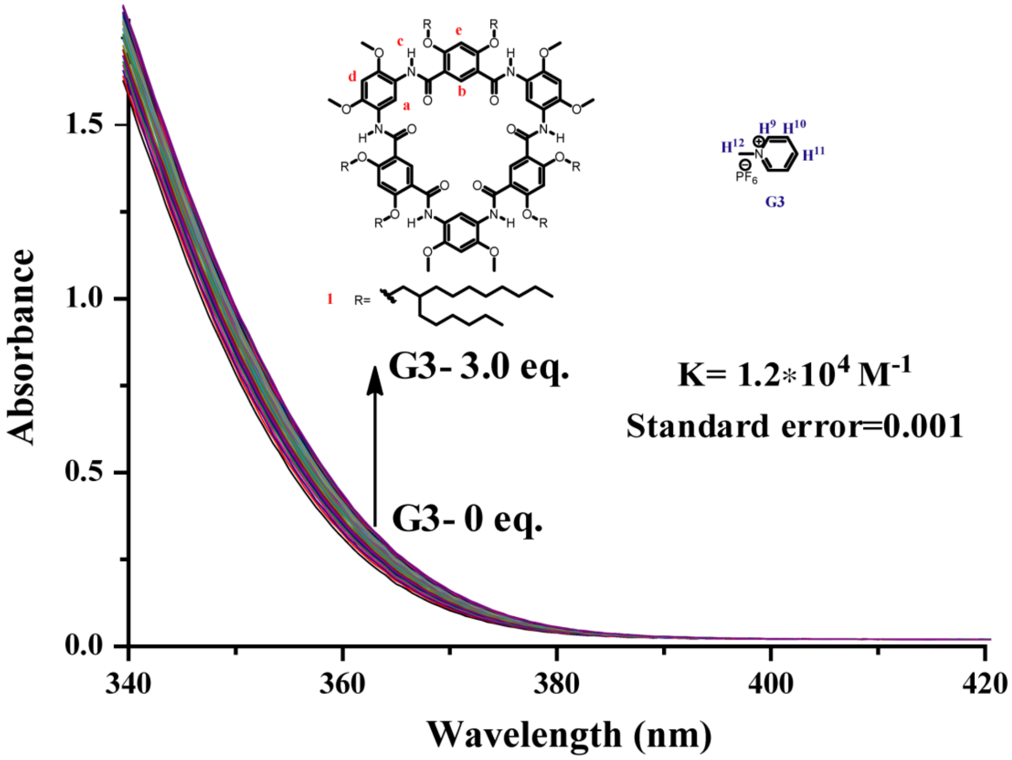

Figure S96 Stacked UV-vis spectra of **1**(50 μM**)** titrated with **G3** in acetone from 0 to 3.0 equiv. at 298 K.

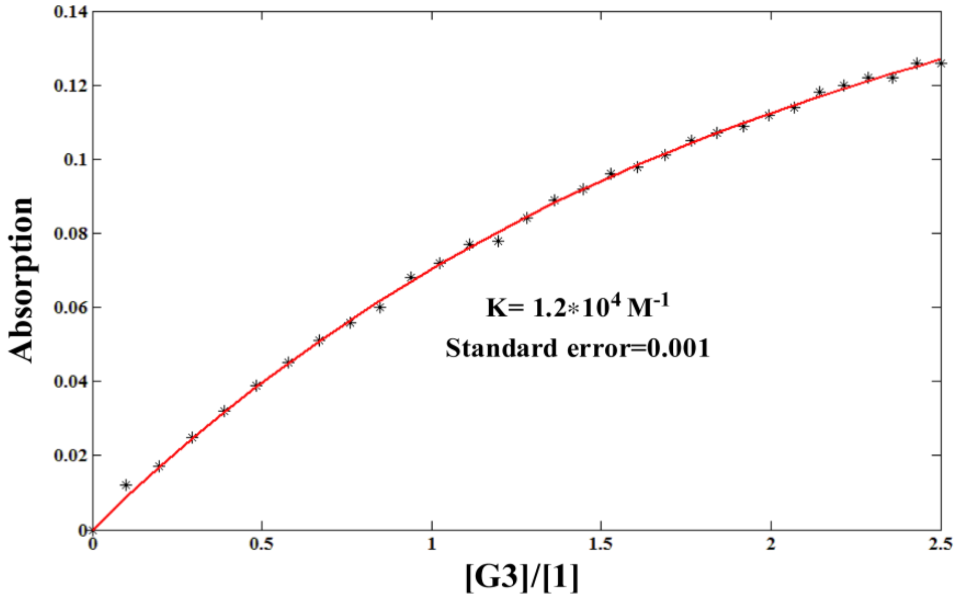

Figure S97 The change of absorption of **1** titrated with **G3** at 360 nm in acetone. The red solid line was obtained from the non-linear curve-fitting with **Eq. S-3.**

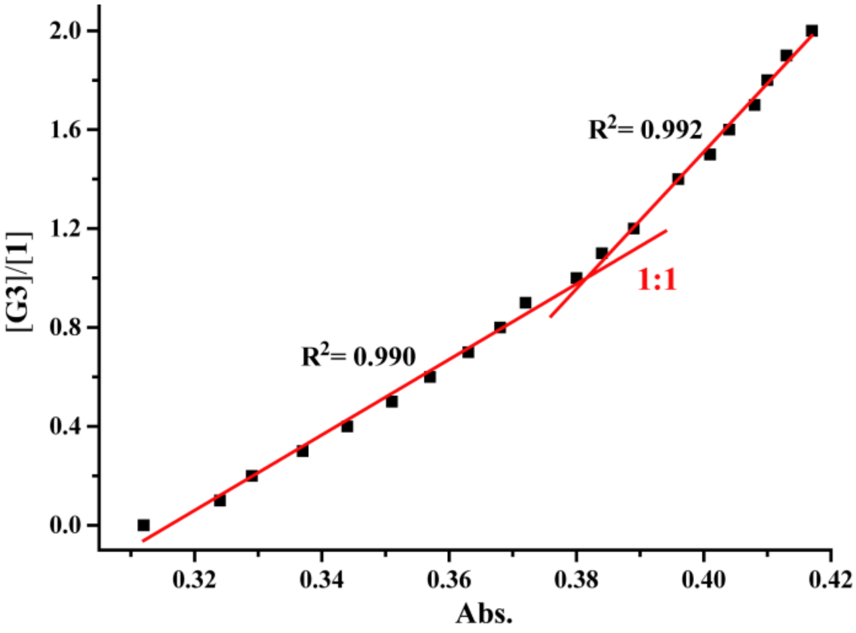

Figure S98 Mole ratio plot for the complexation of **1** and **G3** in acetone indicating a 1:1 stoichiometry at 355 nm in acetone.

The binding constant of complex **1** ⊃ **G3** in CD_3COCD_3 was determined by NMR titration experiments with keeping the concentration of **G3** as a constant (1.0 mM) and varying the concentration of **1** (0-2.0 mM). The binding constant was determined by plotting the chemical shift of proton H^{10} on **G3** versus the concentration of **1** based on

the following equation [2]:

$$\delta_{obs} = \delta_f + \frac{\delta_b - \delta_f}{2[H]_0}\left\{\frac{1}{K_a} + [G]_0 + [H]_0 - \sqrt{\left(\frac{1}{K_a} + [G]_0 + [H]_0\right)^2 - 4[H]_0[G]_0}\right\}$$ **Eq. S-4**

where δ_{obs} is the observed chemical shift of proton H^{10}; δ_b is the chemical shift of proton H^{10} in complex; δ_f is the chemical shift of proton H^{10} in free **G3**; $[H]_0$ is the total concentration of **1**; $[G]_0$ is the total concentration of **G3**; K_a is the binding constant.

The binding constants for $\mathbf{5}_2 \supset$ **G4** (K_1 and K_2) were estimated by a non-linear curve-fitting method with the equation **S-5** and **S-6**:[1]

$$\Delta\delta = (\delta_{\Delta HG}K_1[G]_0[H] + 2\delta_{\Delta H2G}K_1 K_2[G]_0[H]^2) / ([H]_0 (1 + K_1 [H] + K_1 K_2 [H]^2))$$ **Eq. S-5**

$$[H]^3(K_1 K_2) + [H]^2\{K_1 (2K_2[G]_0 - K_2[H]_0 + 1)\} + [H]\{K_1 ([G]_0 - [H]_0 + 1)\} - [H]_0 = 0$$ **Eq. S-6**

Where $[G]_0$ is the concentration of guest **G4**, $[H]_0$ is the concentration of cyclo[6]aramide **5**, $\Delta\delta$ is the chemical shift changes of the complex formed, $\delta_{\Delta HG}$ is the molar extinction coefficient of 1 to 1 host-guest complex, $\delta_{\Delta H_2G}$ is the molar extinction coefficient of 2 to 1 host-guest complex.

^{1}H NMR titration experiments for 1 ⊃ G3

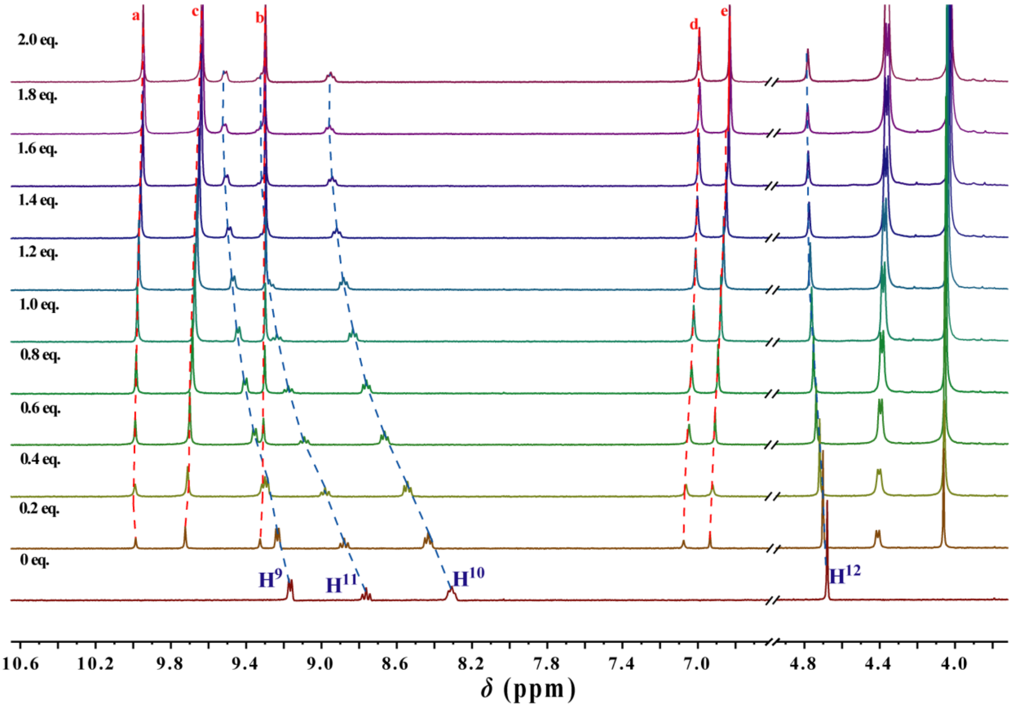

Figure S99 Stacked plots of ^{1}H NMR spectra of **G3** (1 mM) titrated with **1** (0-2.0 mM) in acetone-d_6 (400 MHz, 298 K).

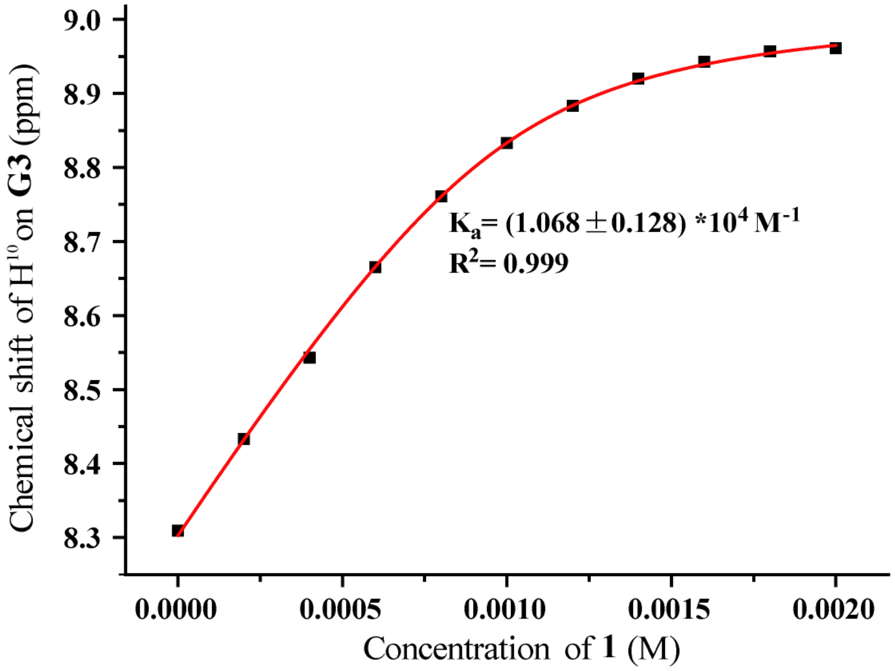

Figure S100 Determination of the binding constant of **1** ⊃ **G3** in acetone at 298 K. Fitting result based on H^{10}. **Figure 8** Changes of the chemical shift changes of proton **l'** on **4** with addition of **G4** (The red solid line was obtained from the non-linear curve-fitting using above equations)

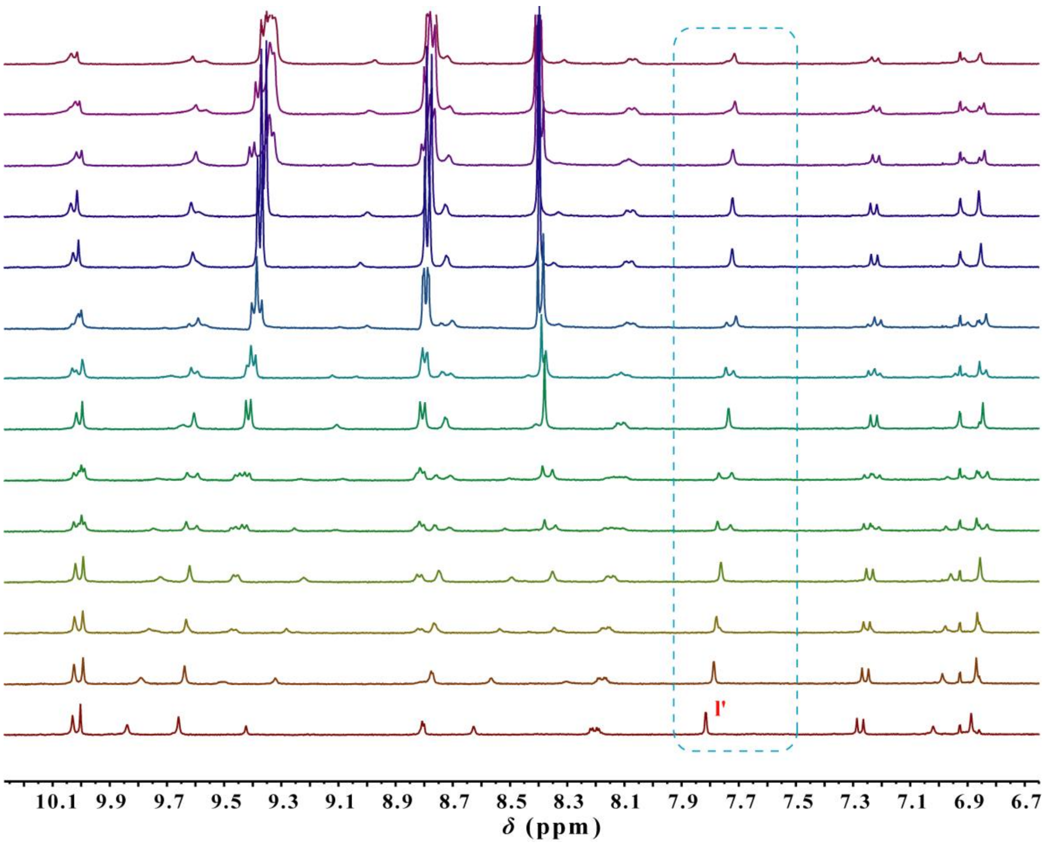

Figure S101 Partial stacked ^1H NMR spectra (400 MHz, 298 K, CD_3COCD_3, 1 mM) of $\mathbf{5_2}$ ⊃ **G4** in the presence of the different concentration of **G4** (0-6.0 eq.).

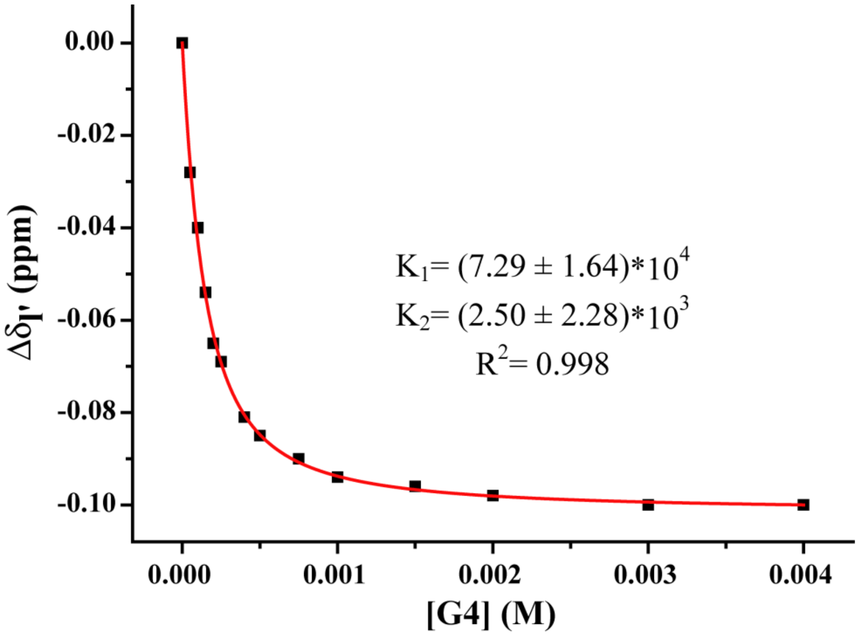

Figure S102 Changes of the chemical shift changes of proton **l'** on **5** with addition of **G4** (The red solid line was obtained from the non-linear curve-fitting using above equations **S-5** and **S-6**).

We can quantify the extent of this cooperativity with the interaction parameter a according to **Eq. S-5**.[1] If $\alpha > 1$ the system displays positive cooperativity, if $\alpha <$ 1 it displays negative cooperativity and if $\alpha = 1$, the system displays non-cooperative binding.

$$\alpha = \frac{4K_2}{K_1} \qquad \textbf{Eq. S-5}$$

References

[1] Thordarson, P., *Chem. Soc. Rev.* **2011**, *40*, 1305-1323.

[2] Bisson, A. P.; Carver, F. J.; Eggleston, D. S.; Haltiwanger, R. C.; Hunter, C. A.;. Livingstone, D. L; McCabe, J. F.; Rotger, C.; Rowan, A. E., *J. Am. Chem. Soc.*, **2000**, *122*, 8856.

4.6 MALDI-TOF-MS Spectra of Complexes

Matrix-assisted laser ionization time of flight mass spectrometry (MALDI-TOF-MS) analyses of cyclo[6]aramide **1** carried in the presence of each of the guests **G1-G4** produced several ions consistent with the formation of host-guest complexes.

MALDI-TOF-MS Spectra of $1_2 \supset$ G1

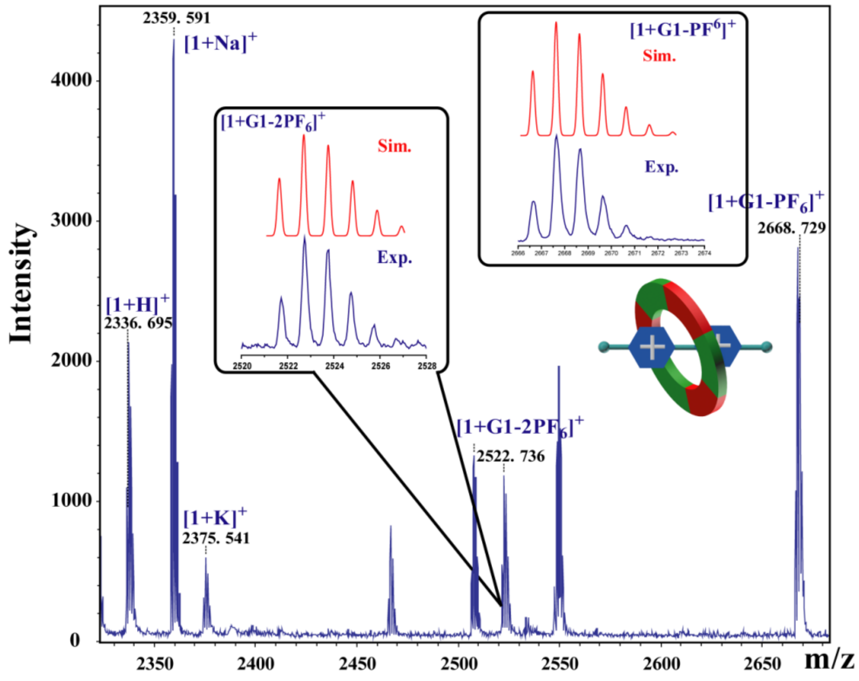

Figure S103 Partial MALDI-TOF mass spectrum of a mixture of $1_2 \supset$ **G1** (1:1) (inset: experimental isotope distribution (blue) and computer simulation (red)).

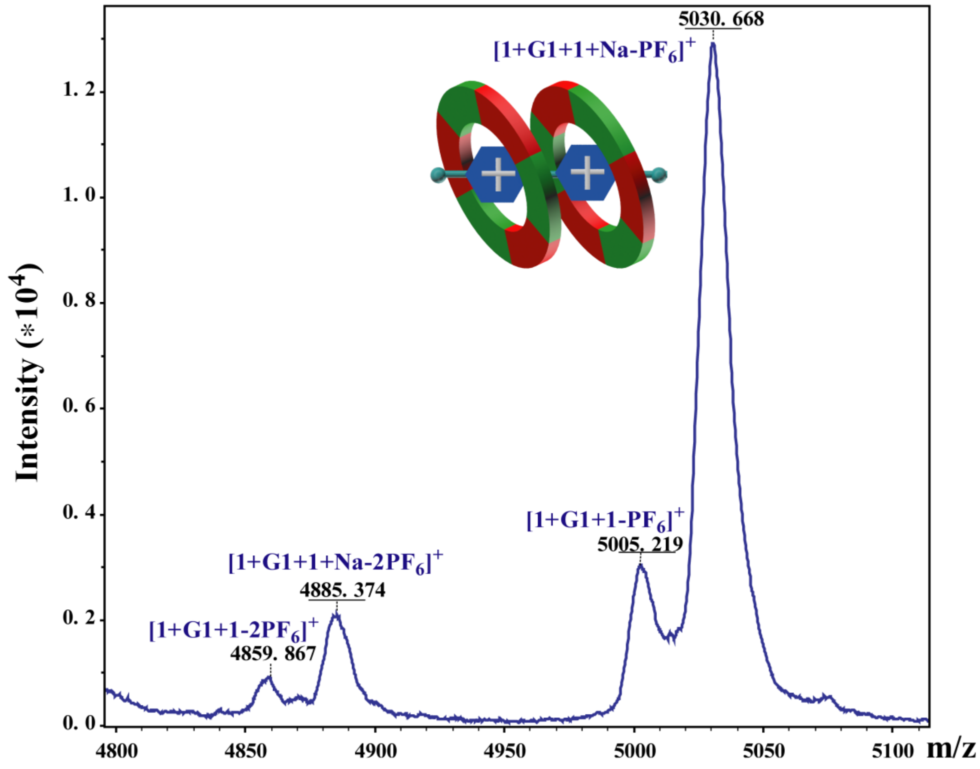

Figure S104 Partial MALDI-TOF mass spectrum of a mixture of $\mathbf{1}_2 \supset$ **G1** (2:1).

MALDI-TOF-MS Spectra of $1_2 \supset$ G2

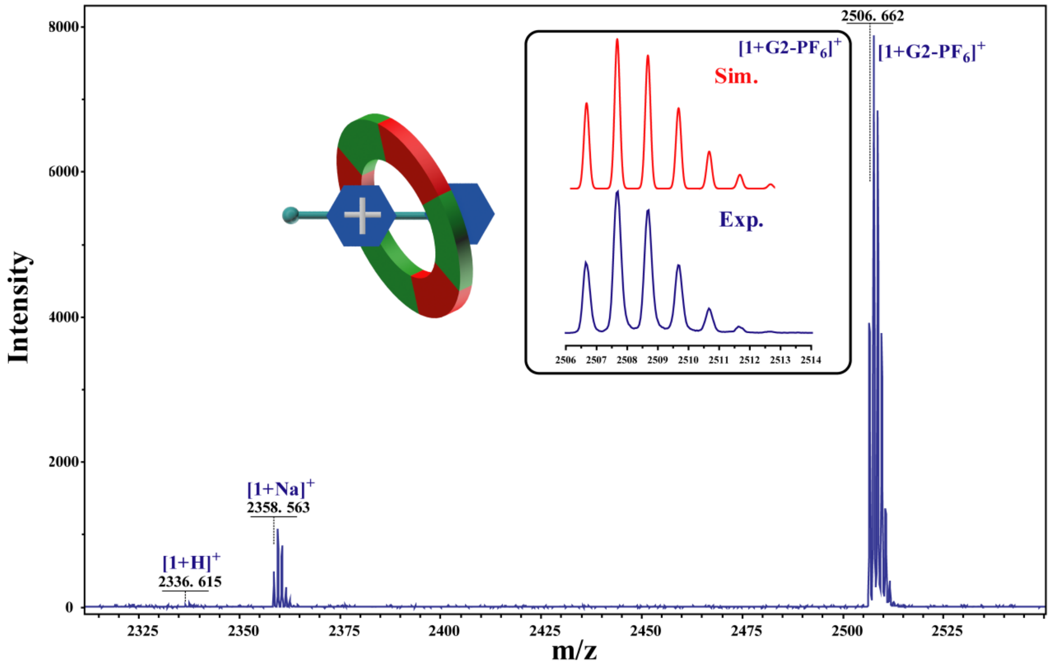

Figure S105 Partial MALDI-TOF mass spectrum of a mixture of $\mathbf{1}_2 \supset$ **G2** (1:1) (inset: experimental isotope distribution (blue) and computer simulation (red)).

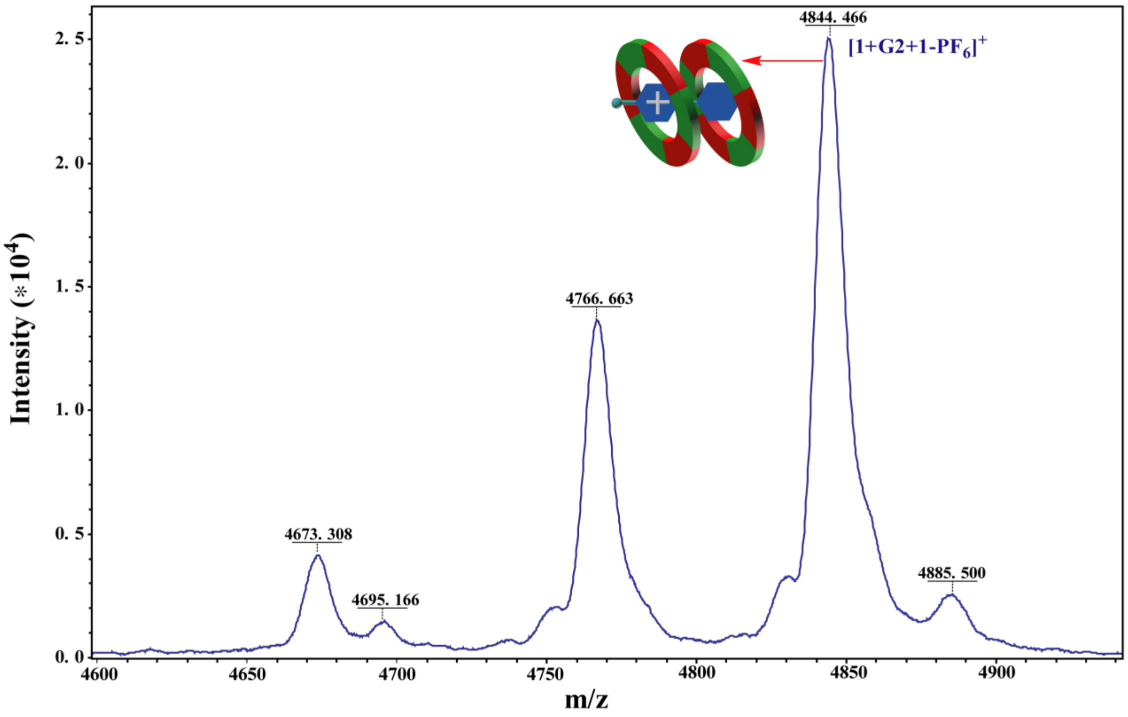

Figure S106 Partial MALDI-TOF mass spectrum of a mixture of $\mathbf{1}_2 \supset$ **G2** (2:1).

MALDI-TOF-MS Spectra of 1 ⊃ G3

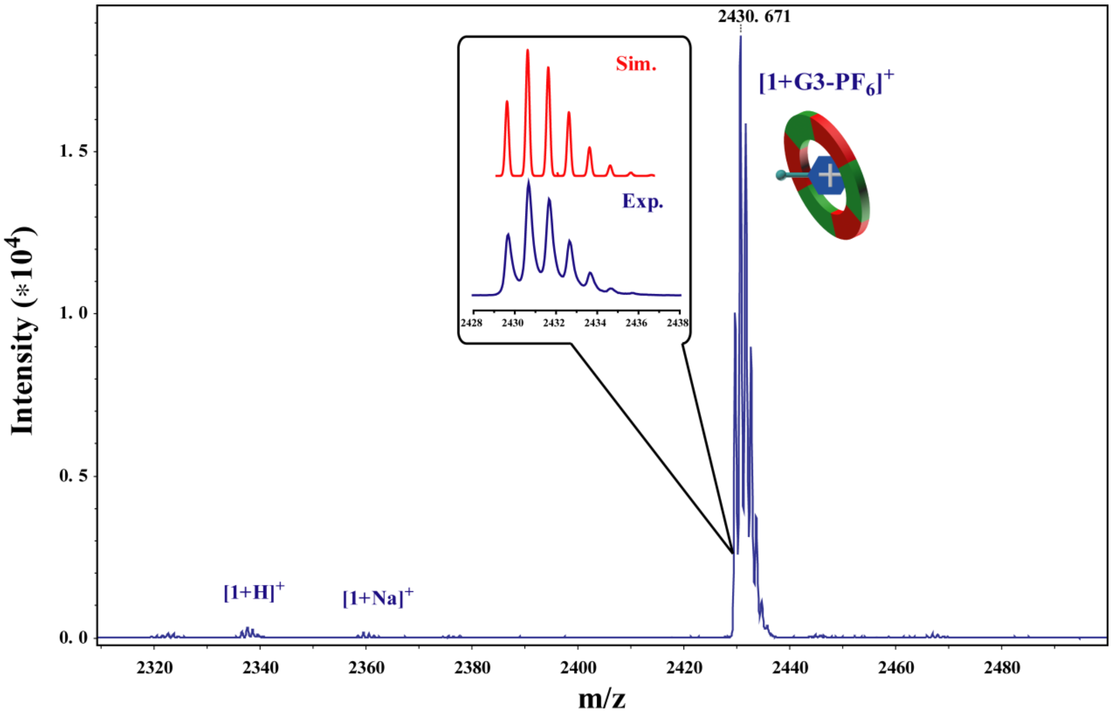

Figure S107 Partial MALDI-TOF mass spectrum of a mixture of **1** ⊃ **G3** (1:1) (inset: experimental isotope distribution (blue) and computer simulation (red)).

MALDI-TOF-MS Spectra of $1_2 \supset$ G4

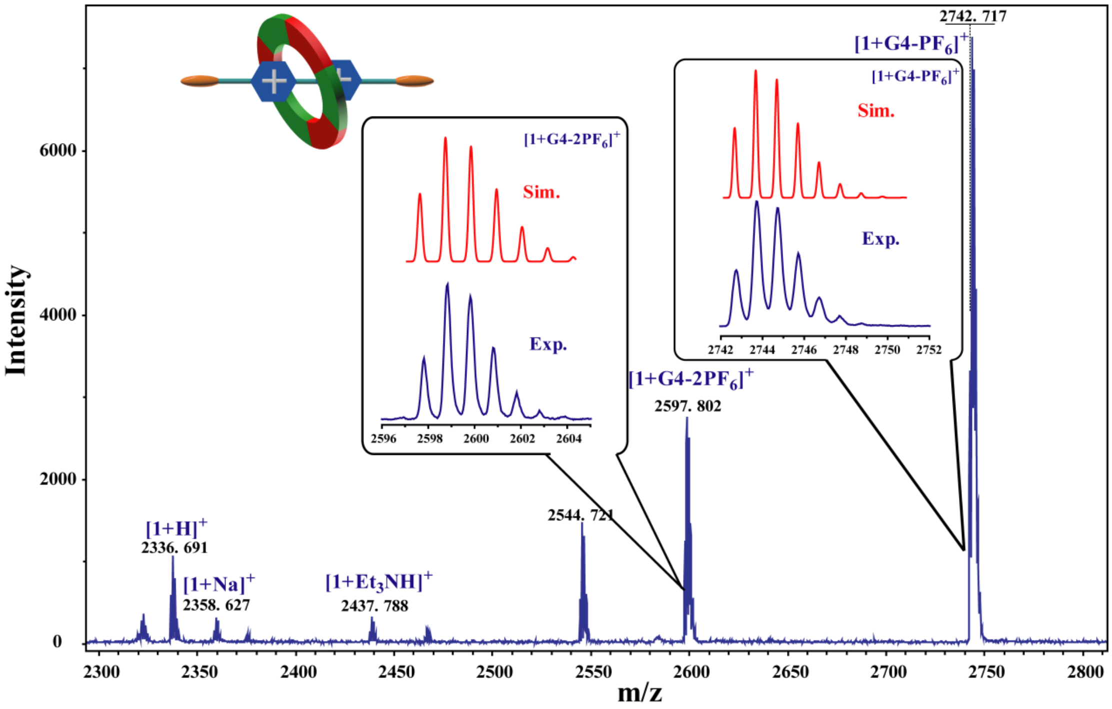

Figure S108 Partial MALDI-TOF mass spectrum of a mixture of $\mathbf{1}_2 \supset$ **G4** (1:1) (inset: experimental isotope distribution (blue) and computer simulation (red)).

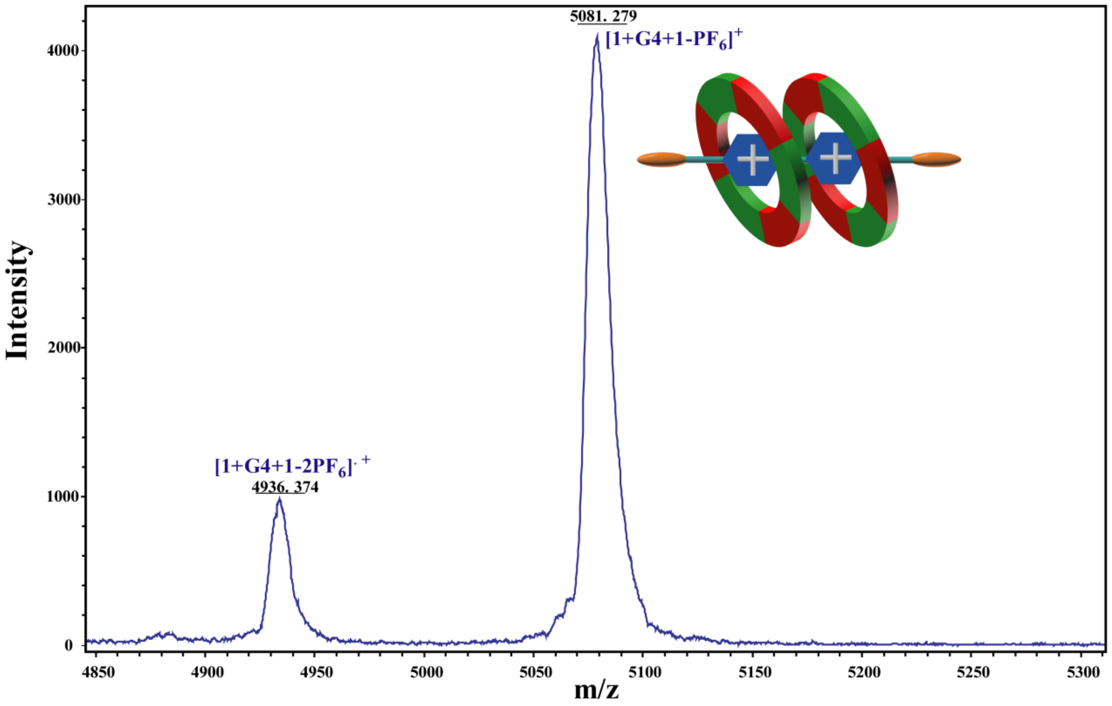

Figure S109 Partial MALDI-TOF mass spectrum of a mixture of $\mathbf{1}_2 \supset$ **G4** (2:1).

4.7 FT-IR Spectra of Complexes

Fourier transform infrared spectrometry (FT-IR) analyses of cyclo[6]aramide **1** carried in the presence of each of the guest **G1-G4** produced $\nu_{C=O}$ shifts consistent with the formation of solid phase host-guest complexes.

FT-IR spectra of $\mathbf{1_2} \supset$ **G1**

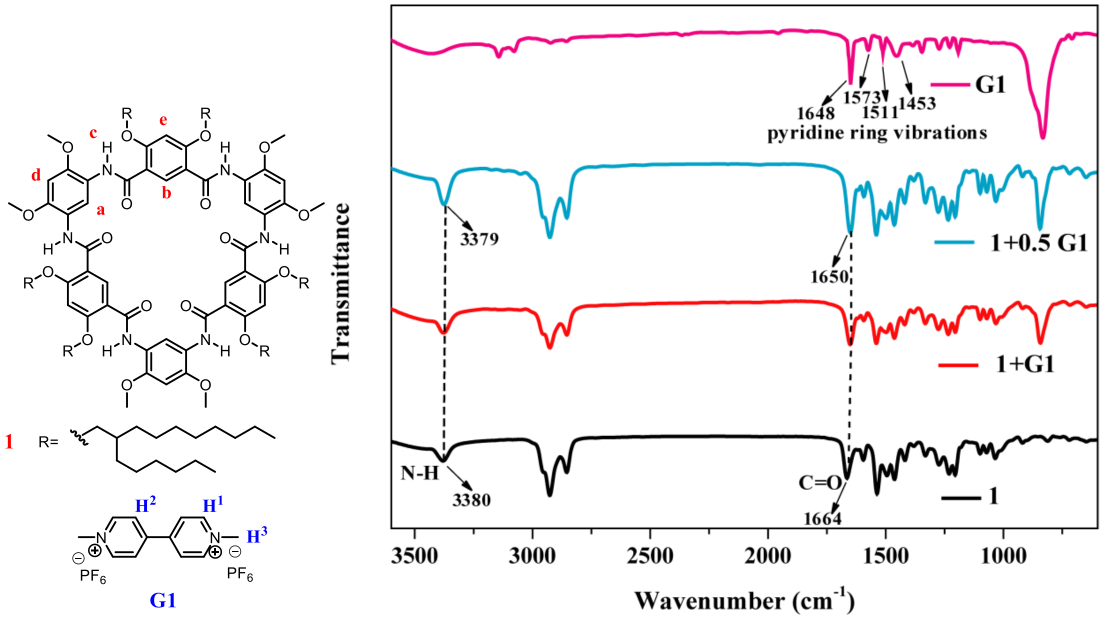

Figure S110 FT-IR spectra of **1** in the different equivalent of **G1**

FT-IR spectra of $\mathbf{1_2} \supset$ **G2**

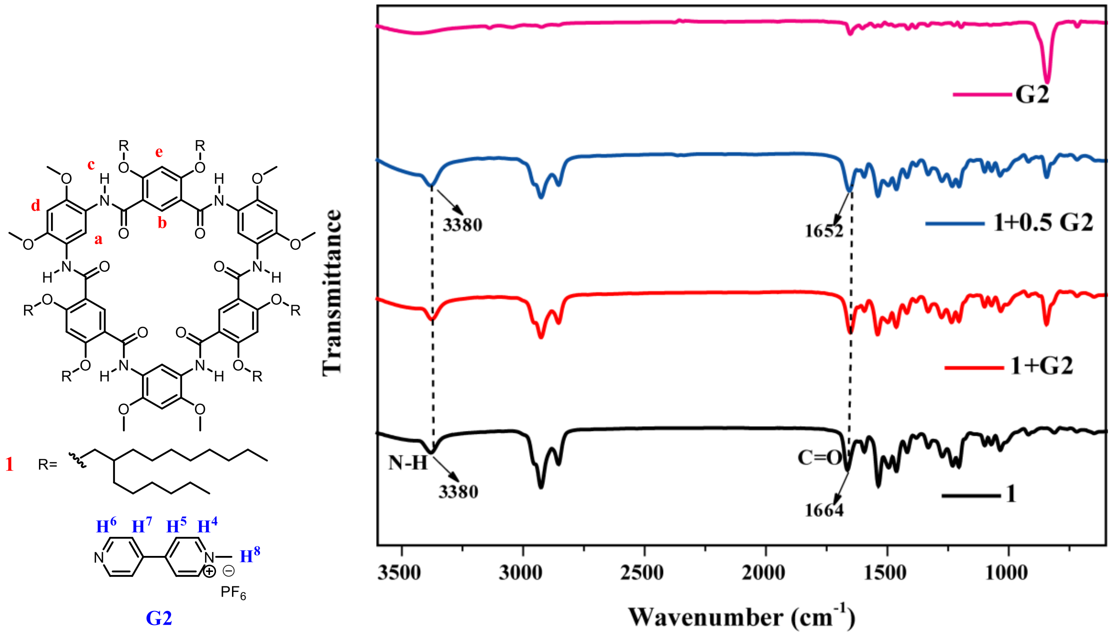

Figure S111 FT-IR spectra of **1** in the different equivalent of **G2**

FT-IR spectra of **1** ⊃ **G3**

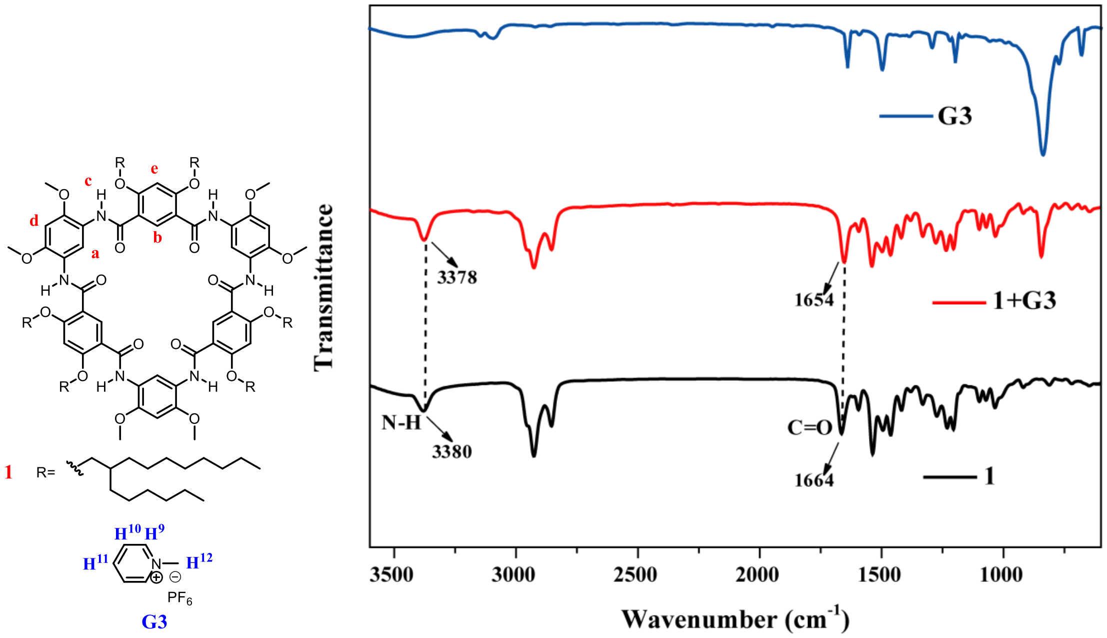

Figure S112 FT-IR spectra of **1** in the different equivalent of **G3**

FT-IR spectra of $\mathbf{1_2}$ ⊃ **G4**

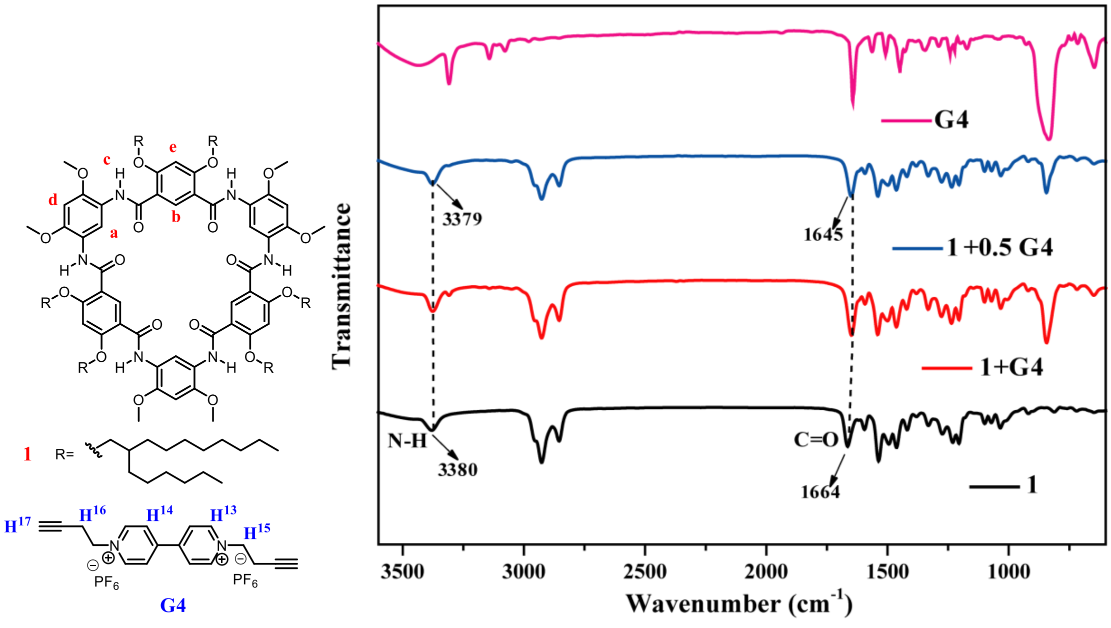

Figure S113 FT-IR spectra of **1** in the different equivalent of **G4**

Table S1 The infrared wave numbers of (C=O) shifts ν (cm^{-1}) on cyclo[6]aramides **1** for the 2:1 or 1:1 solution of the complexes **1** ⊃ **G** in solid state

Complexes	ν_{free} of **1** (cm^{-1})	$\nu_{complex}$ (cm^{-1})	$\Delta\nu = \nu_{complex} - \nu_{free}$
**1** ⊃ **G1**	1664	1650	14
**1** ⊃ **G2**	1664	1652	12
**1** ⊃ **G3**	1664	1654	10
**1** ⊃ **G4**	1664	1645	19

5. Optimization for Synthesis of Rotaxanes

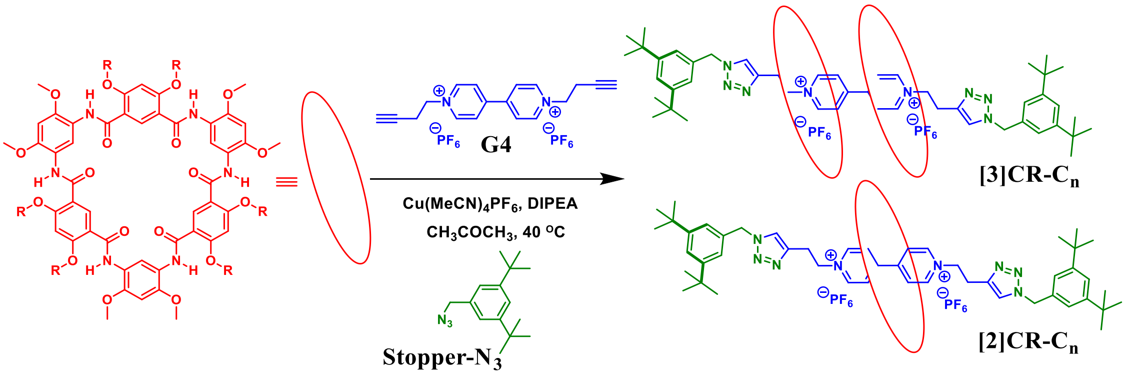

Table S2 "Click-capping" approach for the synthesis of [3]rotaxanes or [2]rotaxanes

Entry	Macrocycle	Conditions	Product Isolated yield (%)[c]	
			[3]Rotaxanes	[2]Rotaxanes
1[a]	**1**	CH_3COCH_3, 40℃, 24 h	86	n.d.
2[a]	**2**	CH_3COCH_3, 40℃, 24 h	91	n.d.
3[a]	**3**	CH_3COCH_3, 40℃, 24 h	64	Trace[d]
4	**1**	CH_3COCH_3 / CH_3CN = 1:1(V/V), 40℃, 24 h [a]	60	18
5[b]	**1**	CH_3COCH_3, 40℃, 24 h	36	34
6	**1**	CH_3COCH_3 / $CHCl_3$ = 1:1(V/V), 40℃, 48 h [a]	79	n.d.

[a] 2.0 equiv. of macrocycle, 2.5 equiv. of **Stopper-N$_3$**, 1.0 equiv. of **G4**, 1.2 equiv. of iPr_2EtN and 0.3 equiv. $CuPF_6(MeCN)_4$. [b] 1.0 equiv. of macrocycle was used. [c] Isolated yield of pure material after chromatography. [d] Observed by MALDI-TOF-MS.

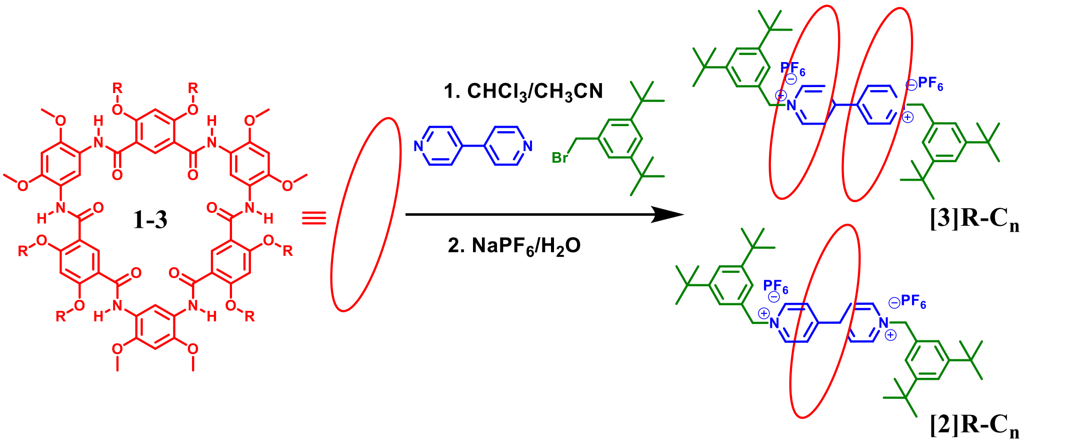

Table S3 "Facile one-pot" approach for the synthesis of [3]rotaxanes or [2]rotaxanes

Entry	Macrocycle	Conditions[a]	Product Isolated yield (%)[b]	
			[3]Rotaxane	[2]Rotaxanes
1	**1**	CH_3CN / $CHCl_3$ = 1:1(V/V), 40℃, 48 h	85	n.d.
2	**2**	CH_3CN / $CHCl_3$ = 1:1(V/V), 40℃, 48 h	85	n.d.
3	**3**	CH_3CN / $CHCl_3$ = 1:1(V/V), 40℃, 48 h	n.d.	71
4	**1**	$CHCl_3$, 40℃, 7 days	77	Not observed

[a] 2.0 equiv. of macrocycle, 2.5 equiv. of **Stopper-Br,** 1.0 equiv. of **4,4'-Bipyridine**. [b] Isolated yield over two steps of pure material after chromatography.

6. Stacked NMR Spectra of Rotaxanes

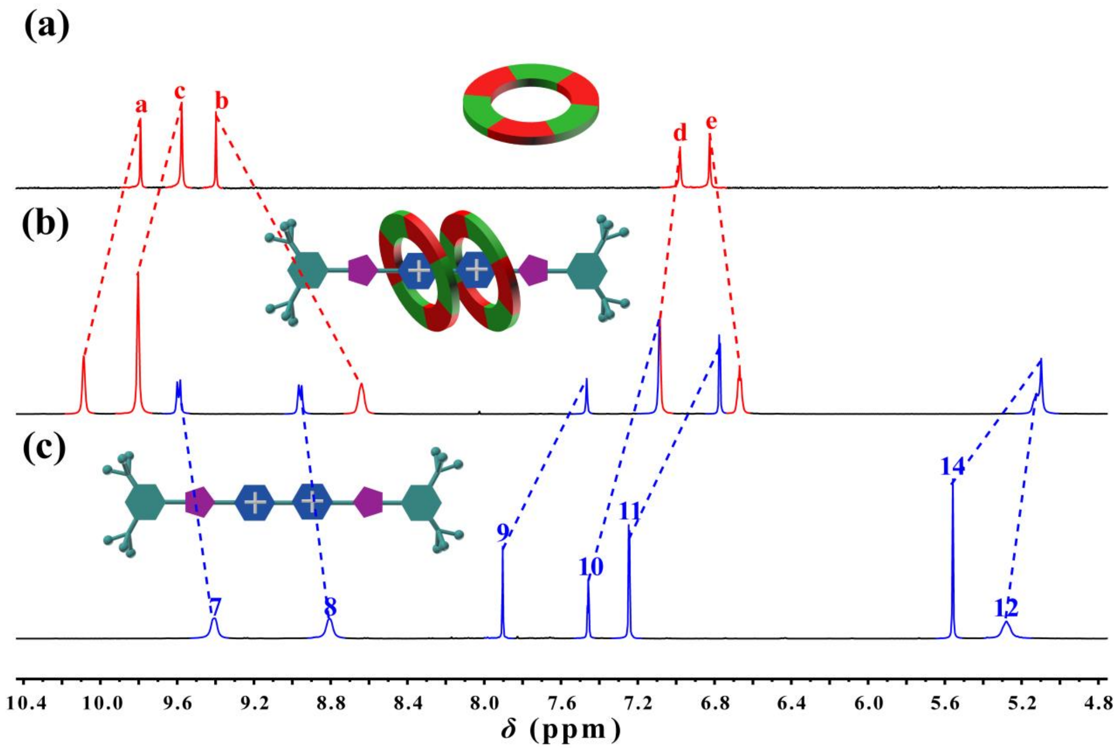

Figure S114 Partial ^{1}H NMR spectrum of (a) **1**, (b) **[3]CR-C_{16}** and (c) **Alxe-1** (400 MHz, CD_3COCD_3, 298 K). The assignments correspond to the lettering shown in main text.

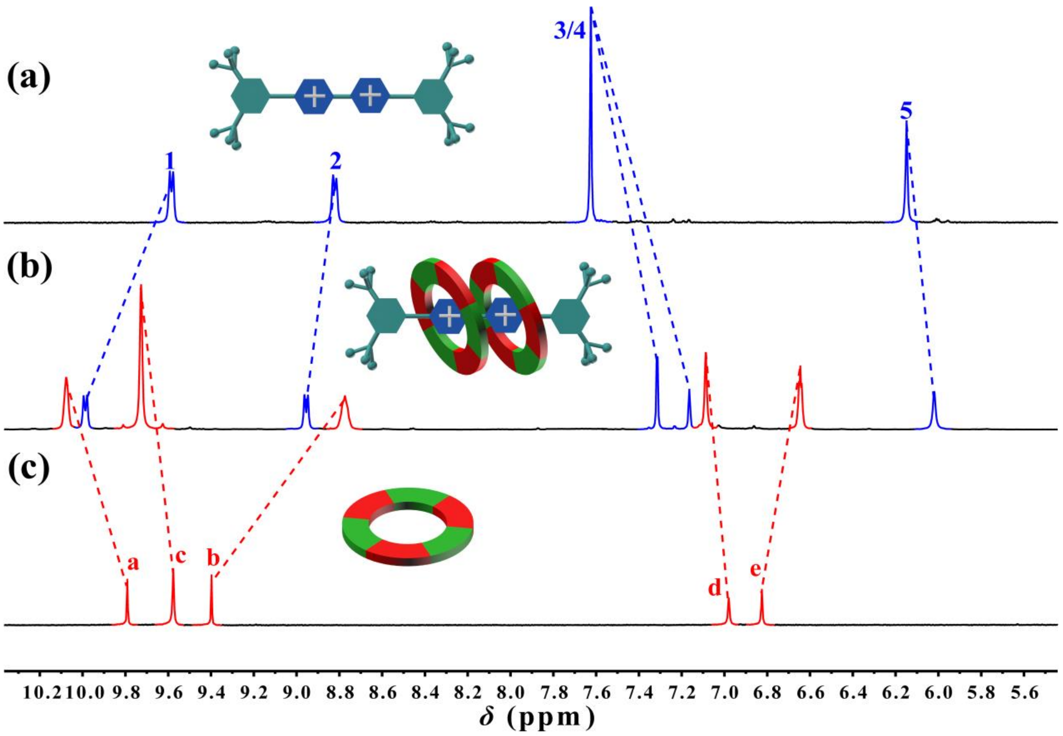

Figure S115 Partial ^{1}H NMR spectrum of (a) **Alxe-2**, (b) **[3]R-C$_{16}$** and (c) **1** (400 MHz, CD_3COCD_3, 298 K). The assignments correspond to the lettering shown in main text.

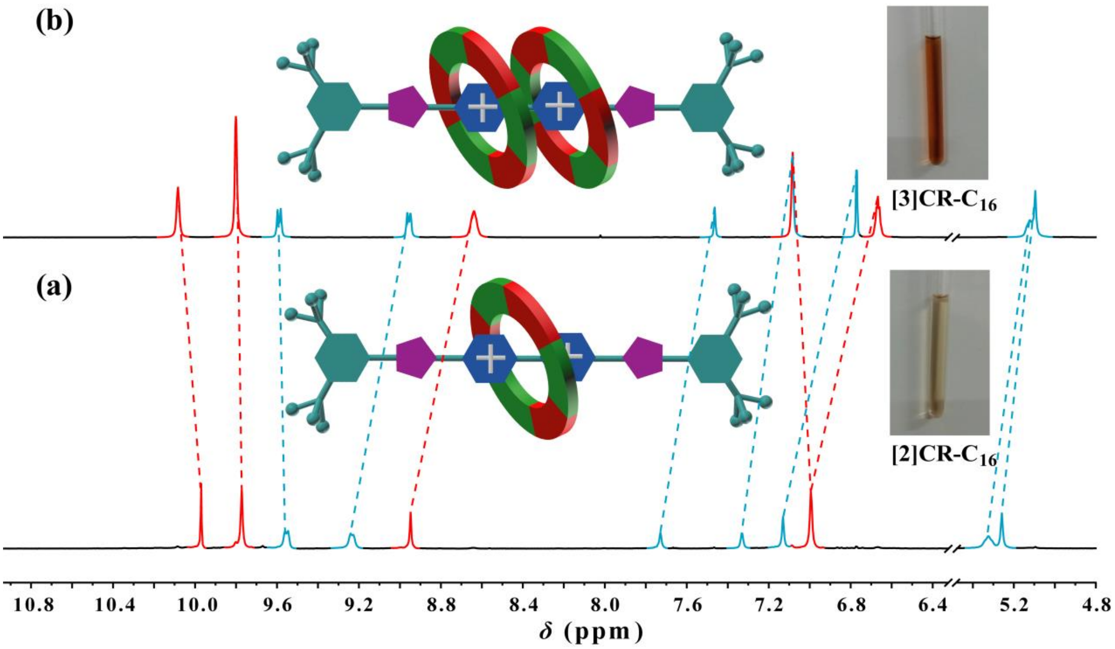

Figure S116 Partial ^{1}H NMR spectra and image of solutions of (a) [2]rotaxane **[2]CR-C$_{16}$** and (b) [3]rotaxane **[3]CR-C$_{16}$** (400 MHz, CD_3COCD_3, 298 K).

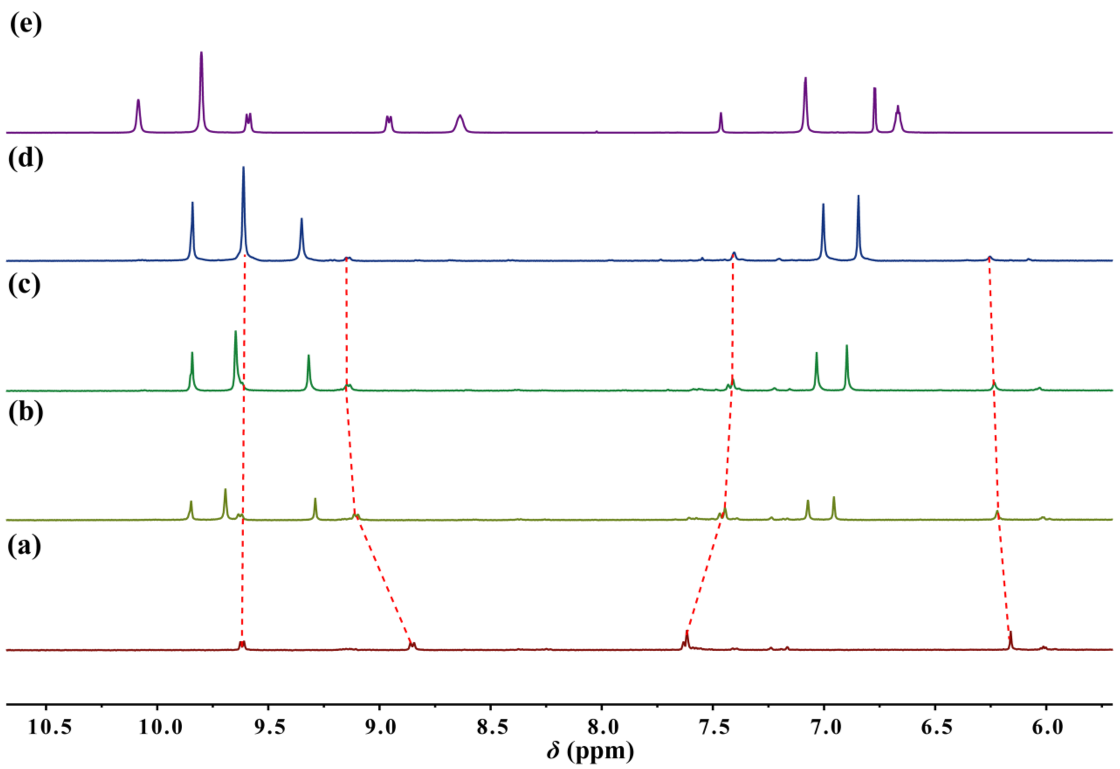

Figure S117 Partial ^{1}H NMR spectra of (a) 1.0 mM **Axle-1**, (b) 1.0 mM **Axle-1** and 2.0 mM macrocycle **1**, (c) 1.0 mM **Axle-1** and 4.0 mM macrocycle **1**, (d) 1.0 mM **Axle-1** and 6.0 mM macrocycle **1** after reflux in CD_3COCD_3/DMSO-d_6, v/v=9:1 for 3 hours, (e) 2.0 mM [3]rotaxane **[3]CR-C$_{16}$** (400 MHz, CD_3COCD_3/DMSO-d_6, v/v=9:1, 298 K).

6.1 2D NOESY, HSQC and HMBC Spectra of Rotaxanes

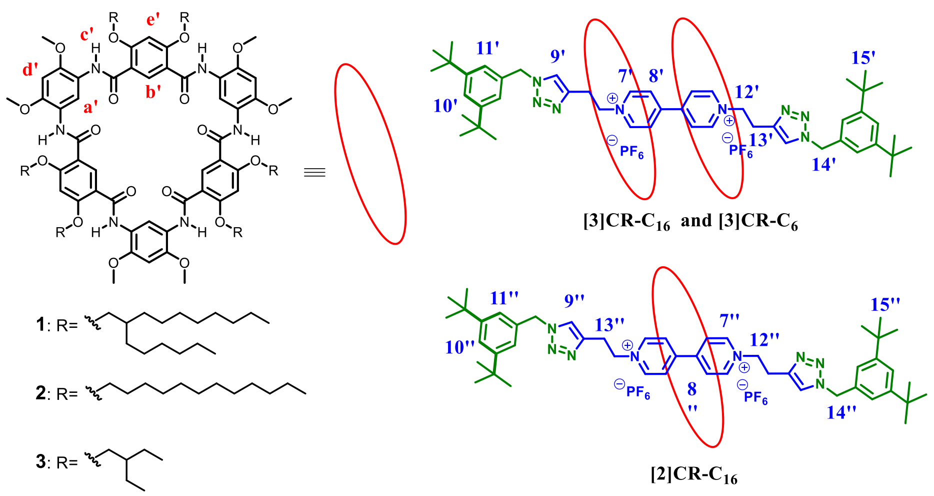

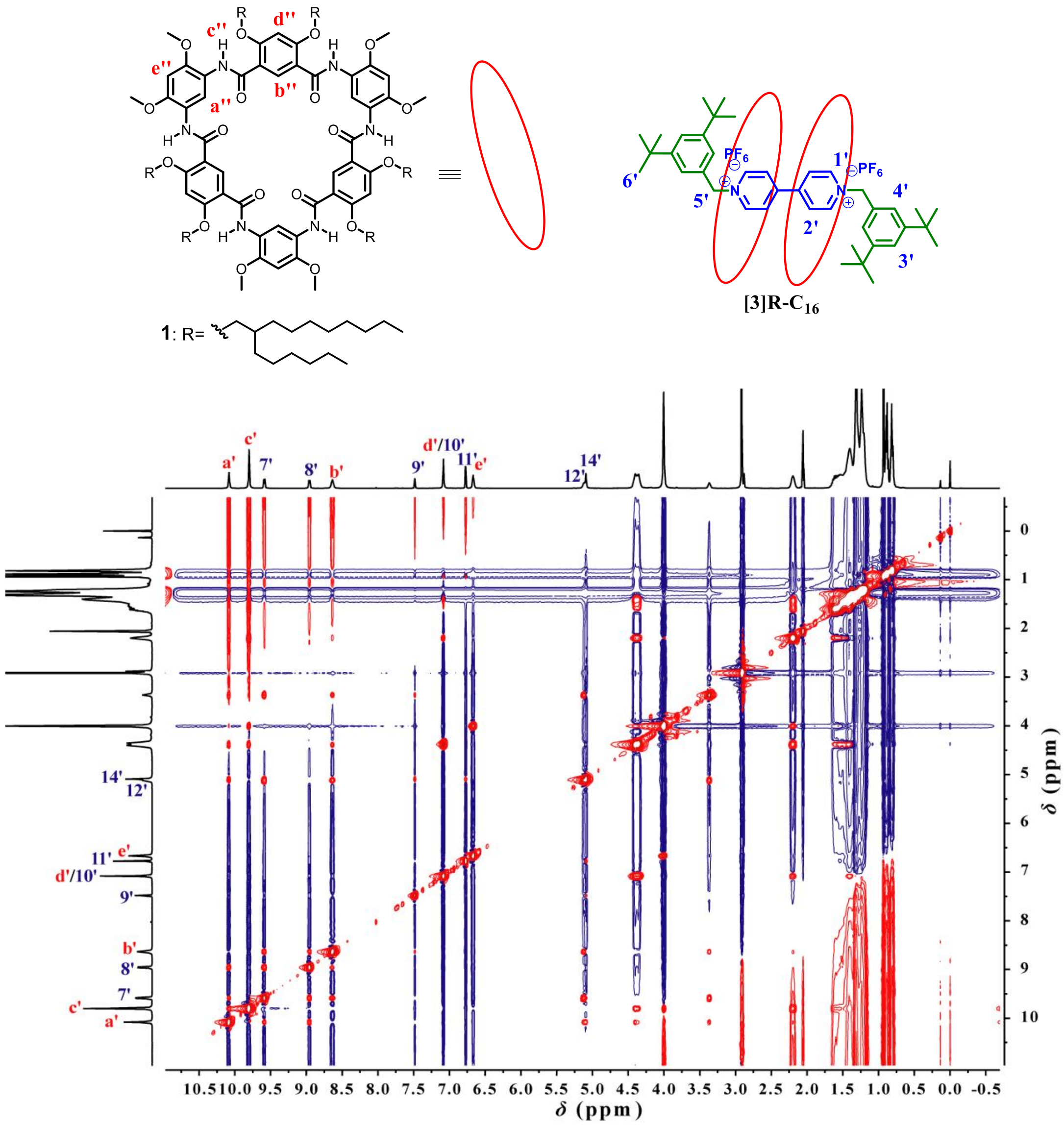

Figure S118 ^{1}H-^{1}H NOESY spectrum of **[3]CR-C$_{16}$** (CD_3COCD_3, 400 MHz, 298K).

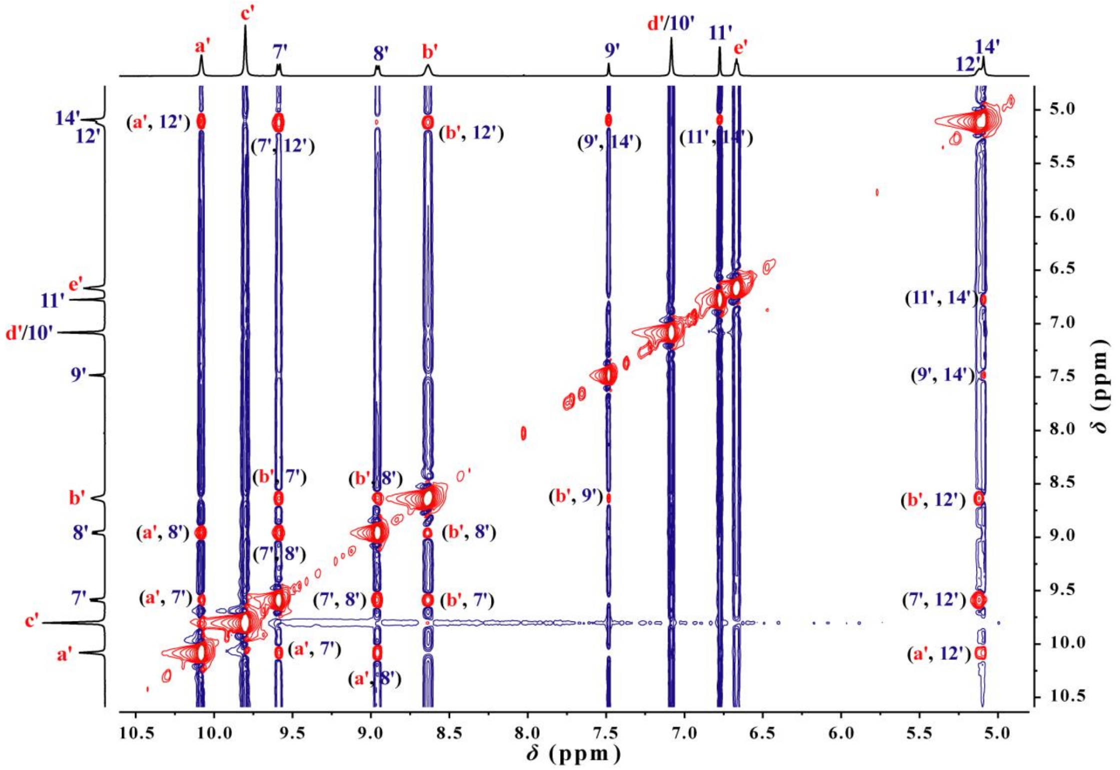

Figure S119 Expanded ^{1}H-^{1}H NOESY spectrum of **[3]CR-C$_{16}$** (CD_3COCD_3, 400 MHz, 298K).

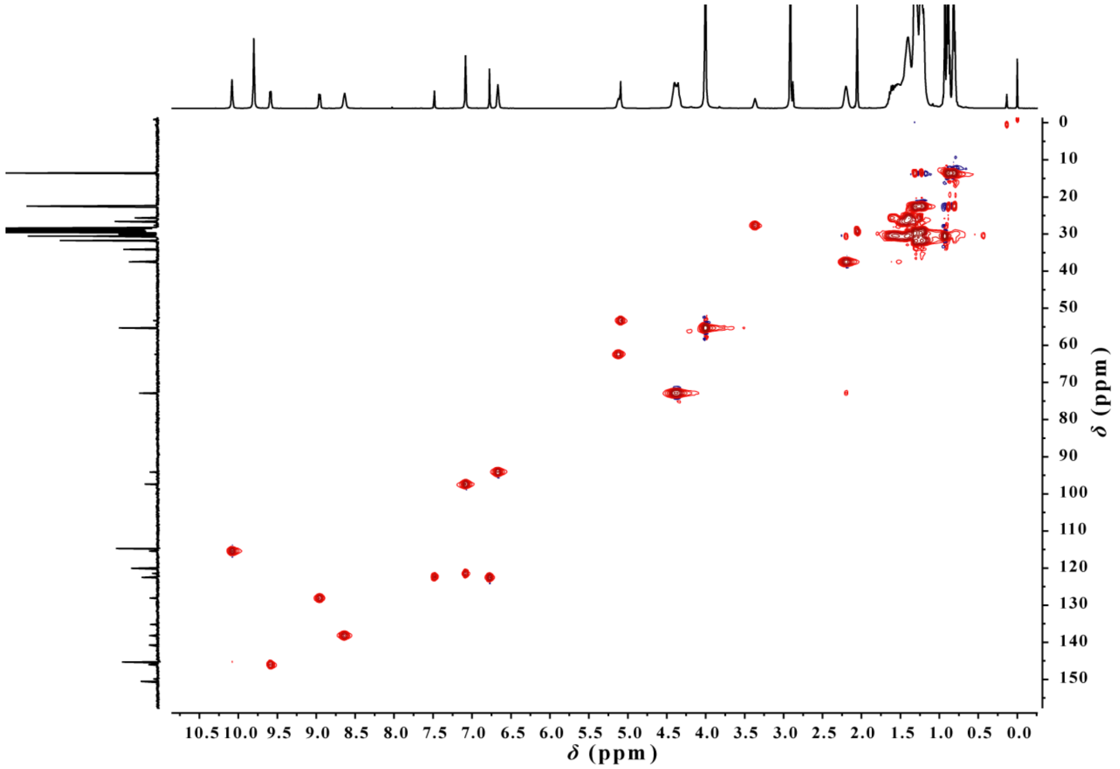

Figure S120 Expanded ^{1}H-^{13}C HSQC spectrum of **[3]CR-C$_{16}$** (CD_3COCD_3, 400 MHz, 298K).

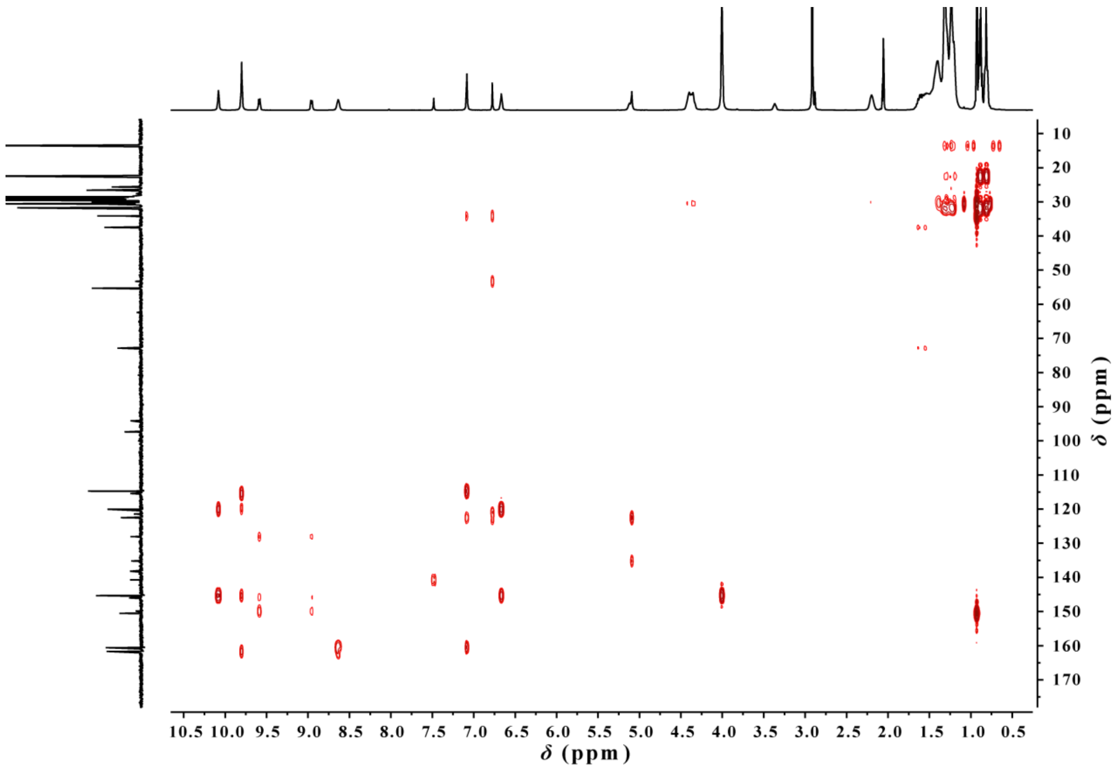

Figure S121 ¹H-¹³C HMBC spectrum of **[3]CR-C_{16}** (CD_3COCD_3, 400 MHz, 298K).

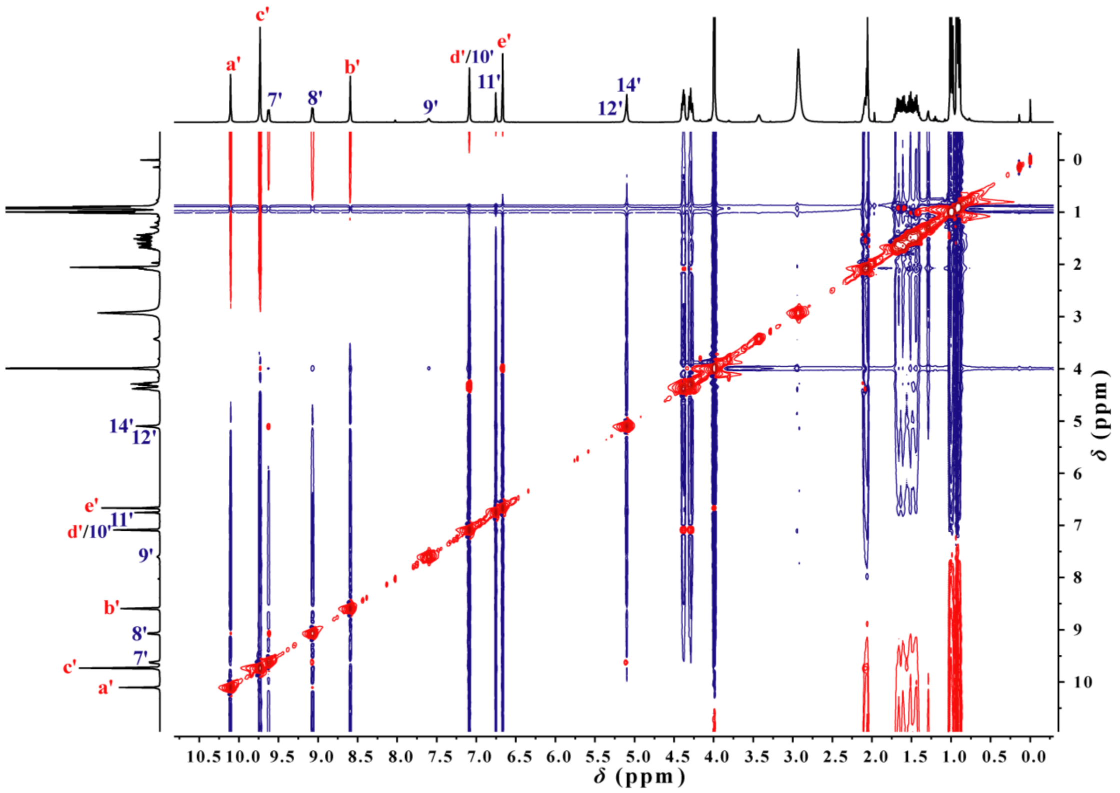

Figure S122 ¹H-¹H NOESY spectrum of **[3]CR-C_6** (CD_3COCD_3, 400 MHz, 298K).

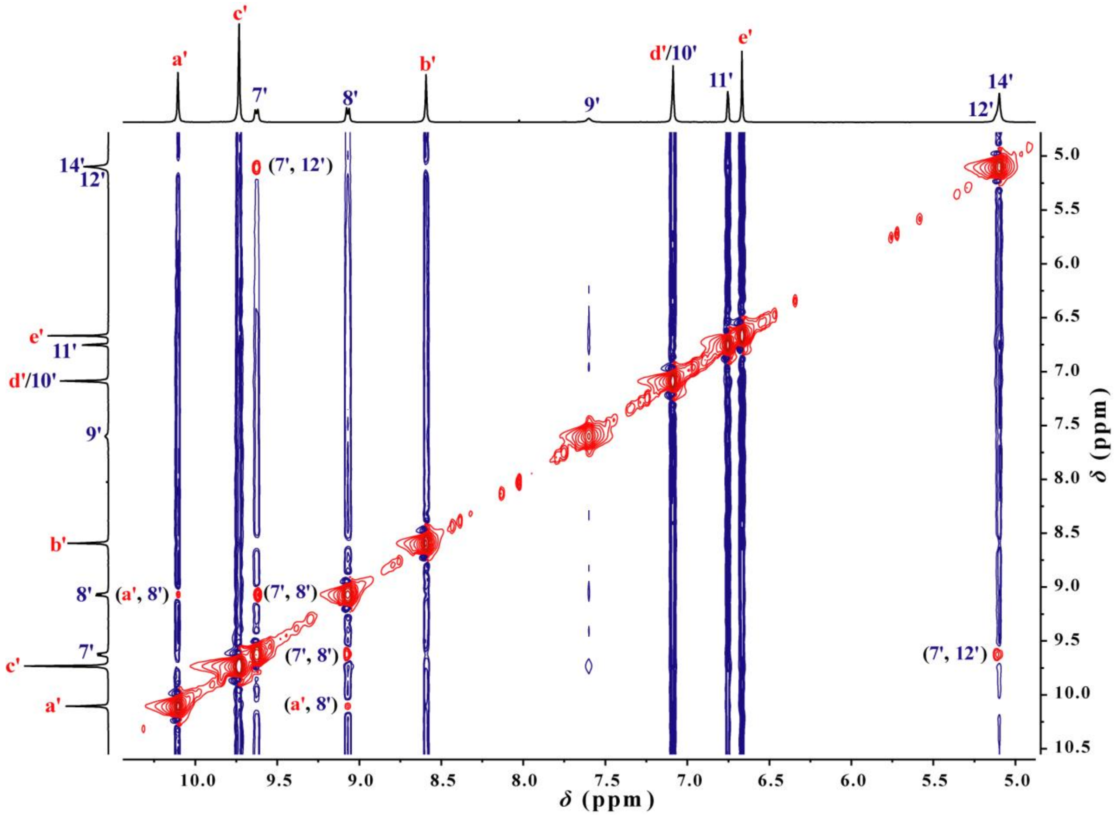

Figure S123 Expanded ^{1}H-^{1}H NOESY spectrum of **[3]CR-C$_6$** (CD_3COCD_3, 400 MHz, 298K).

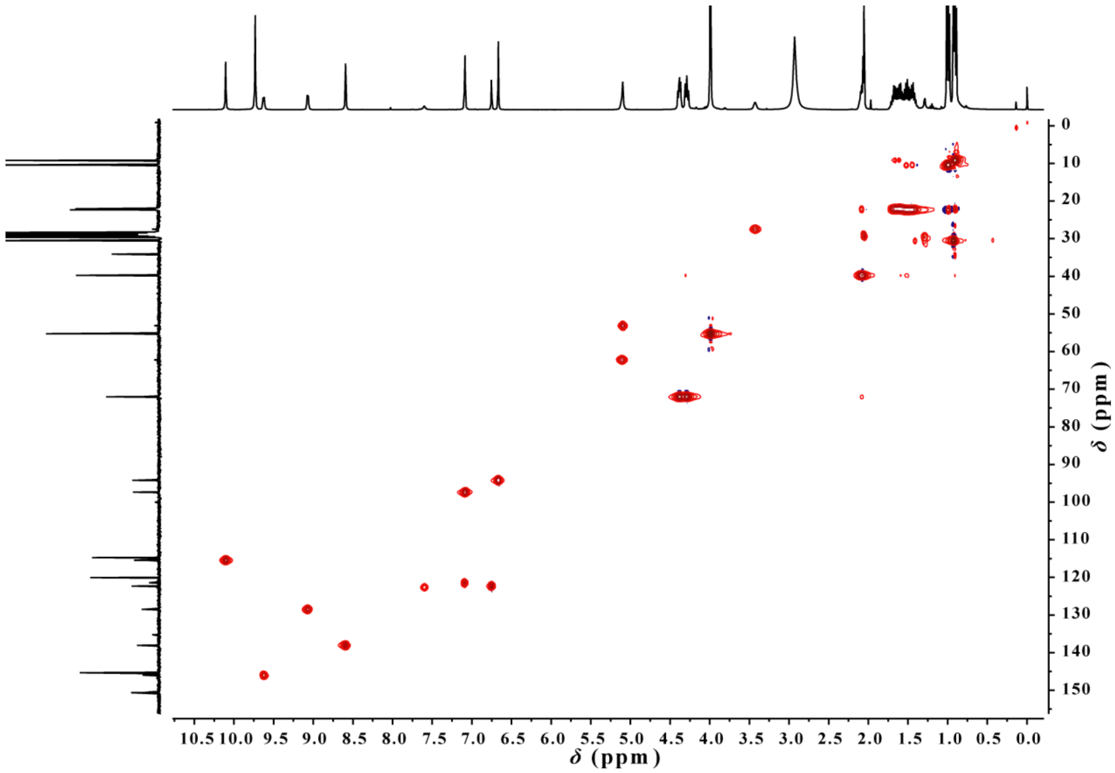

Figure S124 Expanded ^{1}H-^{13}C HSQC spectrum of **[3]CR-C$_6$** (CD_3COCD_3, 400 MHz, 298K).

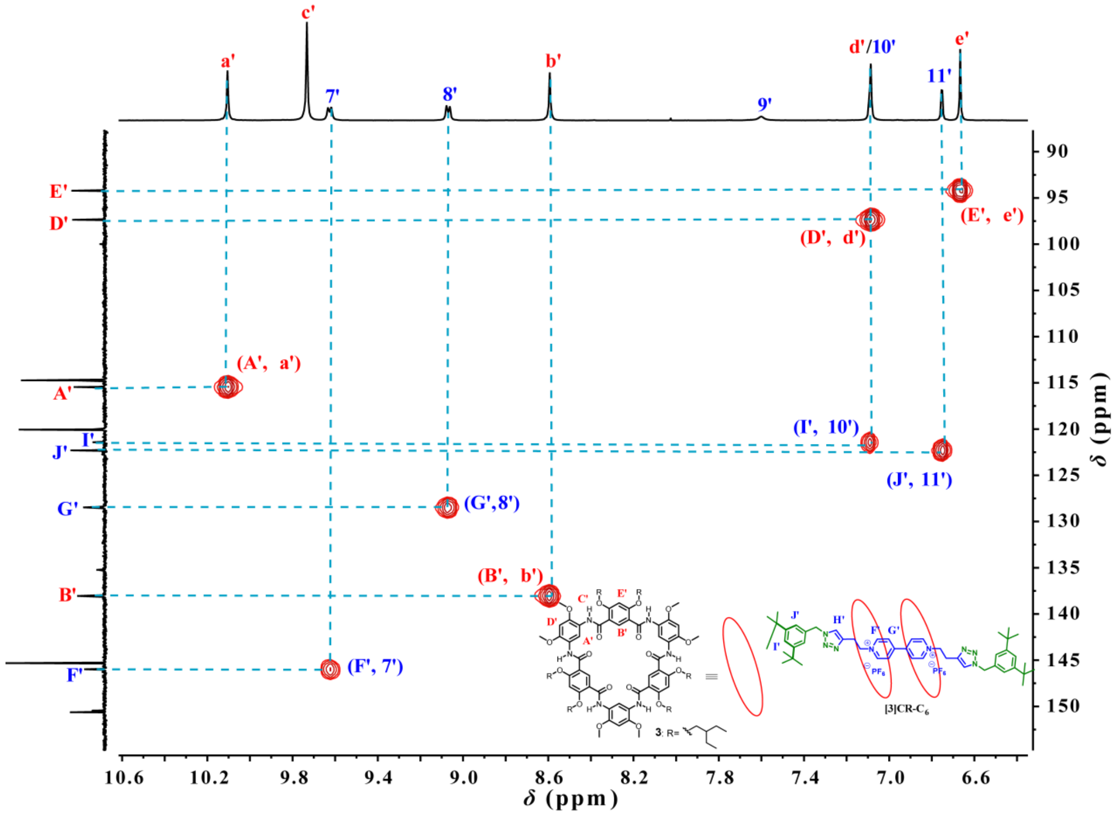

Figure S125 Expanded ^{1}H-^{13}C HSQC spectrum of **[3]CR-C$_6$** (CD_3COCD_3, 400 MHz, 298K).

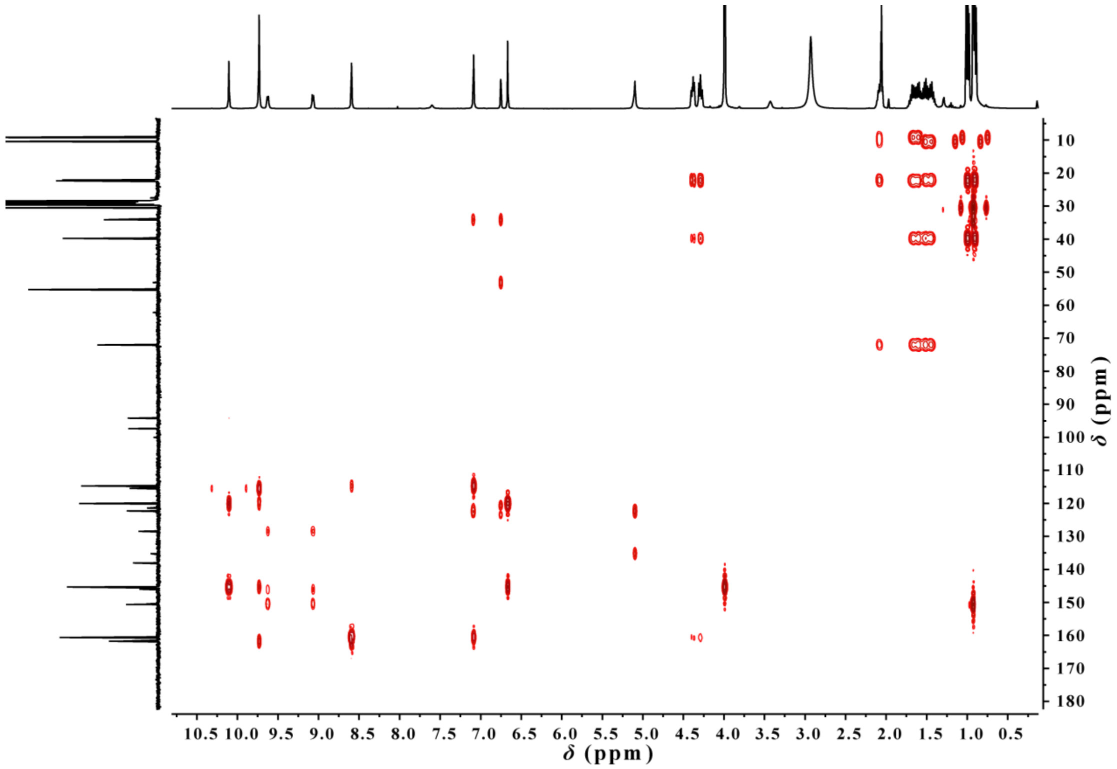

Figure S126 ^{1}H-^{13}C HMBC spectrum of **[3]CR-C$_6$** (CD_3COCD_3, 400 MHz, 298K).

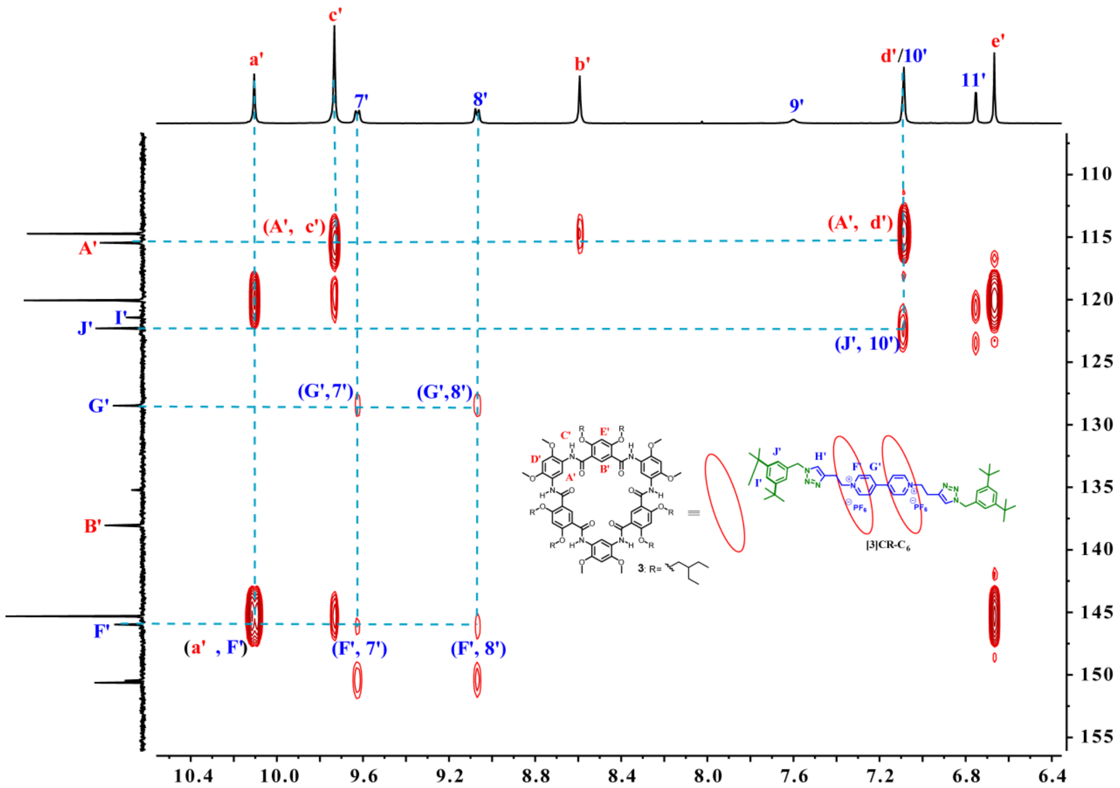

Figure S127 Expanded ^1H-^{13}C HMBC spectrum of **[3]CR-C$_6$** (CD_3COCD_3, 400 MHz, 298K).

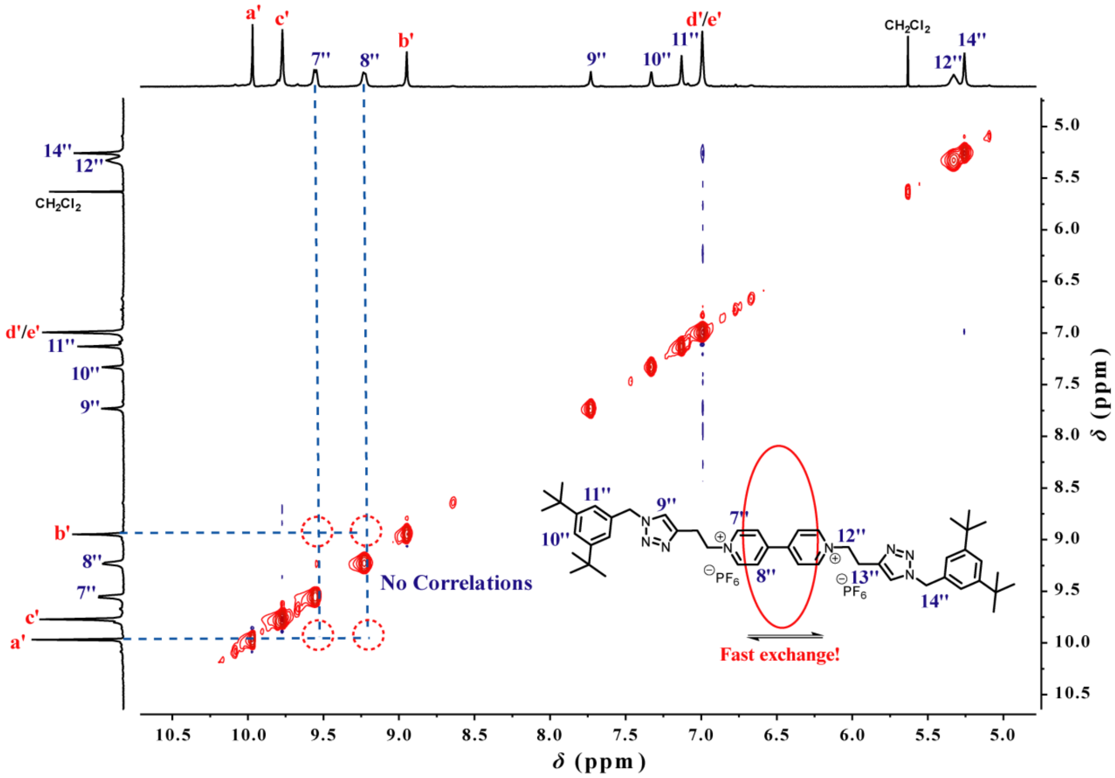

Figure S128 Expanded ^1H-^1H NOESY spectrum of **[2]CR-C$_{16}$** (CD_3COCD_3, 400 MHz, 298K).

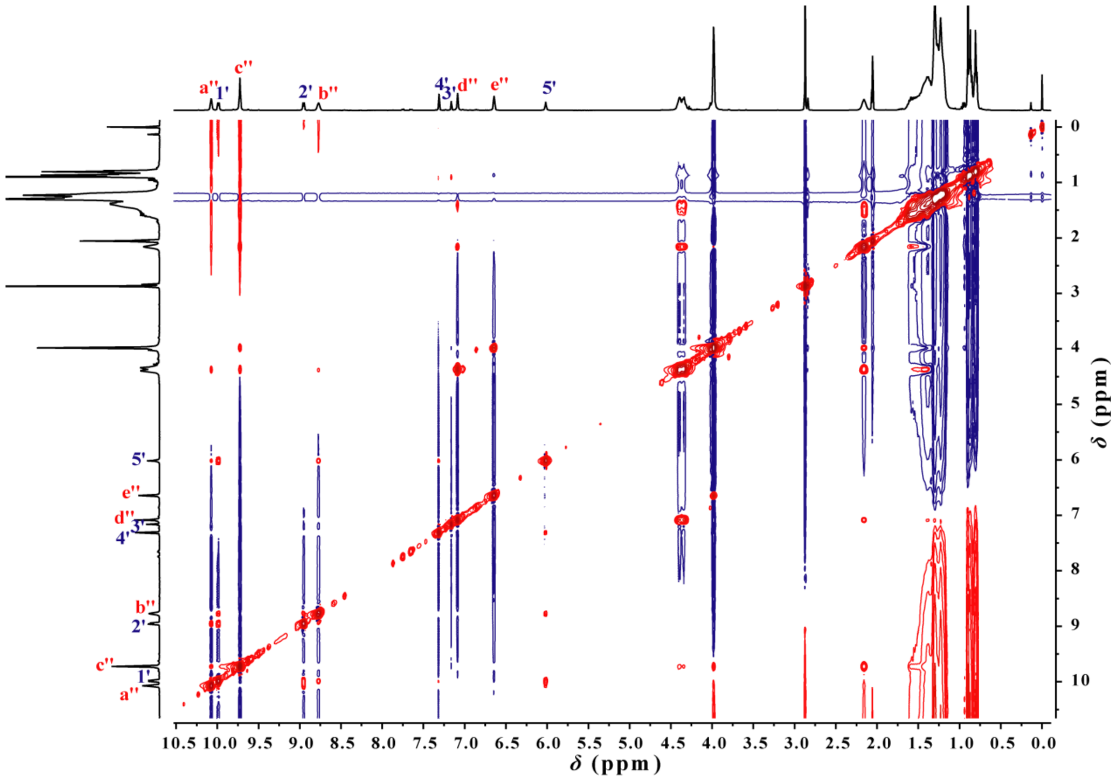

Figure S129 ^{1}H-^{1}H NOESY spectrum of **[3]R-C$_{16}$** (CD_3COCD_3, 400 MHz, 298K).

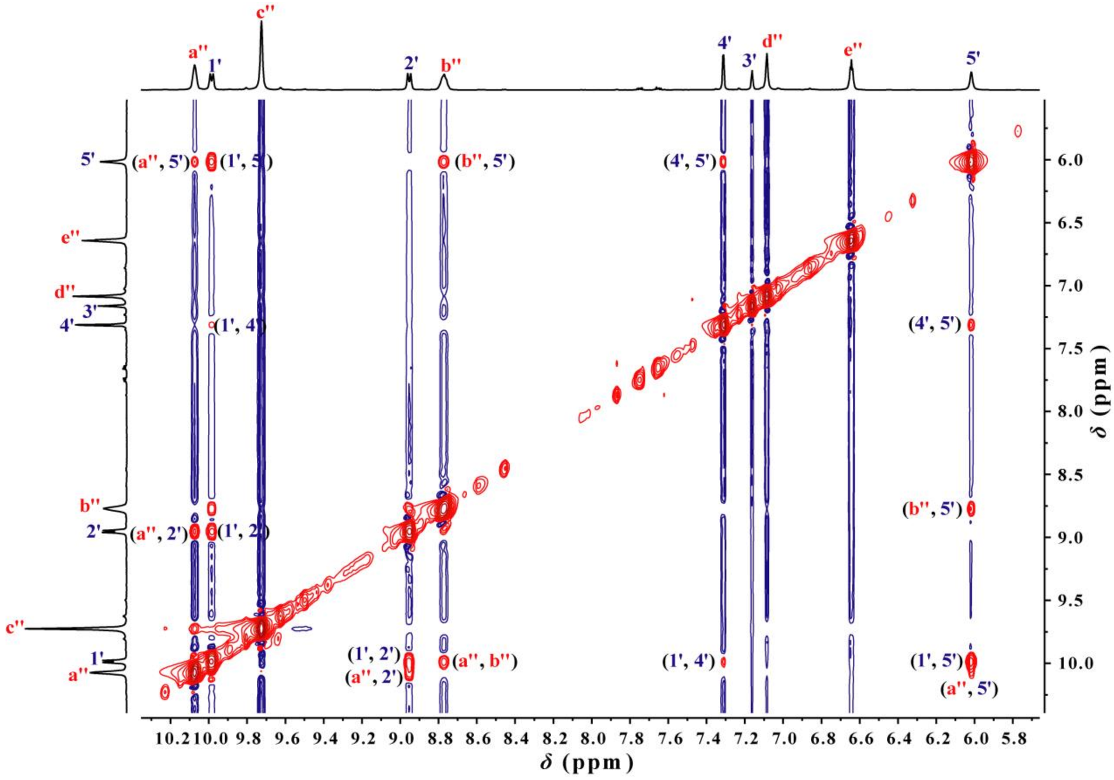

Figure S130 Expanded ^{1}H-^{1}H NOESY spectrum of **[3]R-C$_{16}$** (CD_3COCD_3, 400 MHz, 298K).

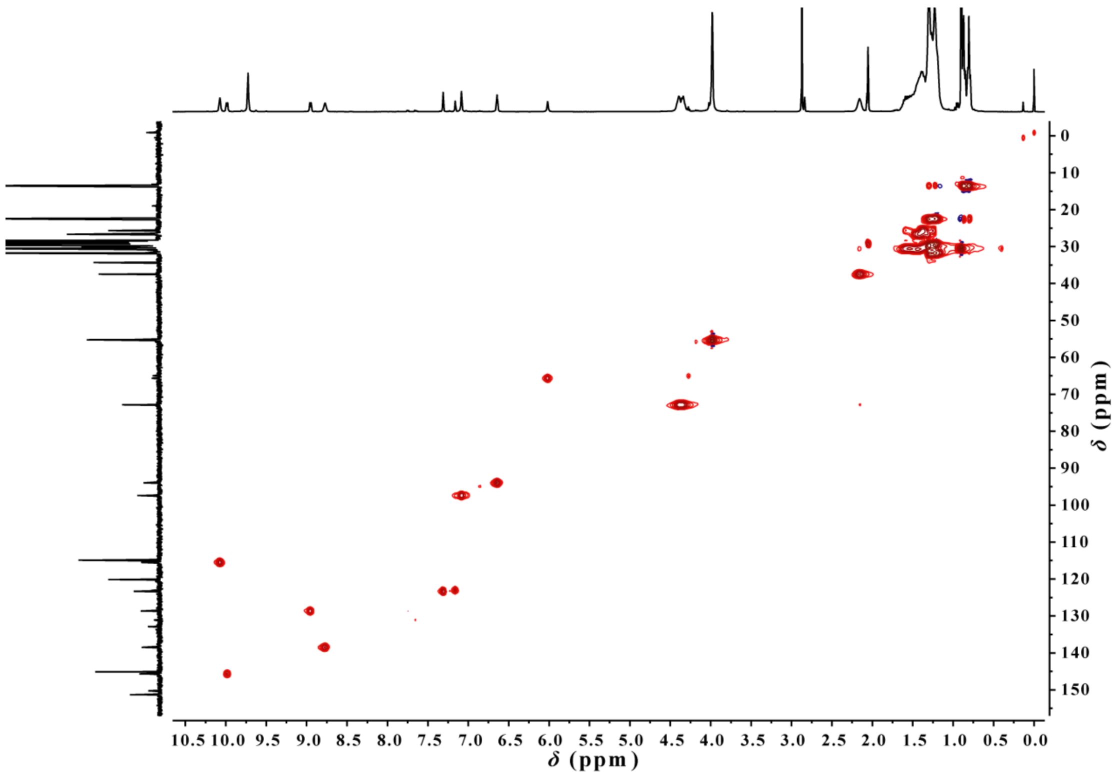

Figure S131 Expanded ^{1}H-^{13}C HSQC spectrum of **[3]R-C$_{16}$** (CD_3COCD_3, 400 MHz, 298K).

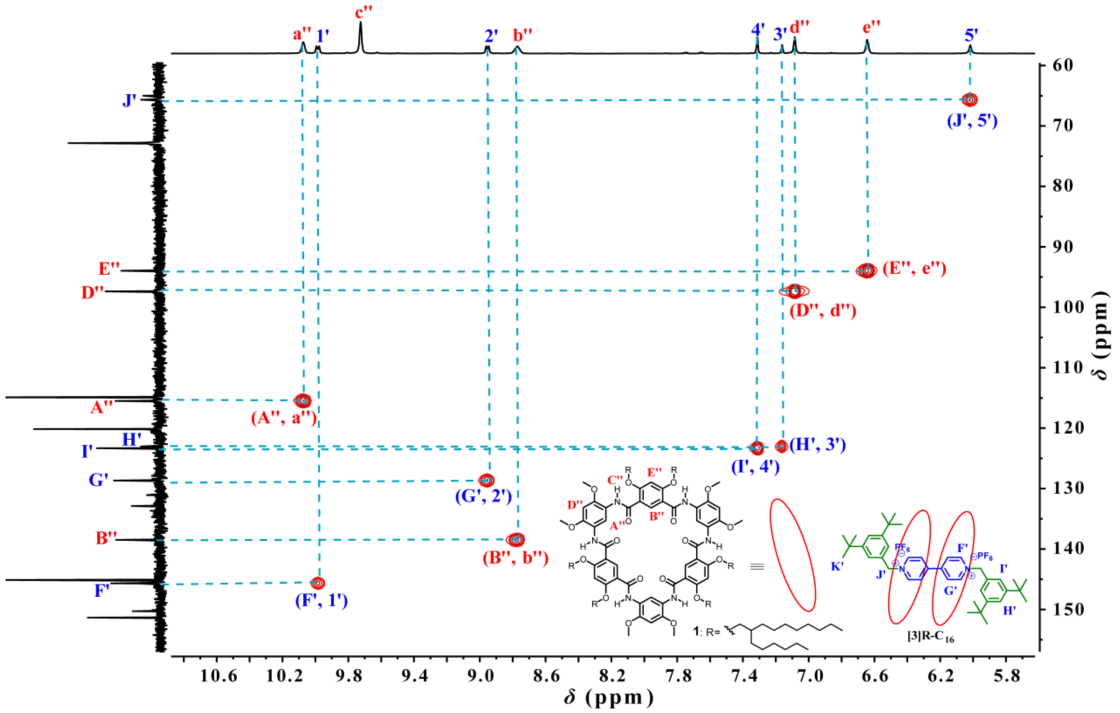

Figure S132 Expanded ^{1}H-^{13}C HSQC spectrum of **[3]R-C$_{16}$** (CD_3COCD_3, 400 MHz, 298K).

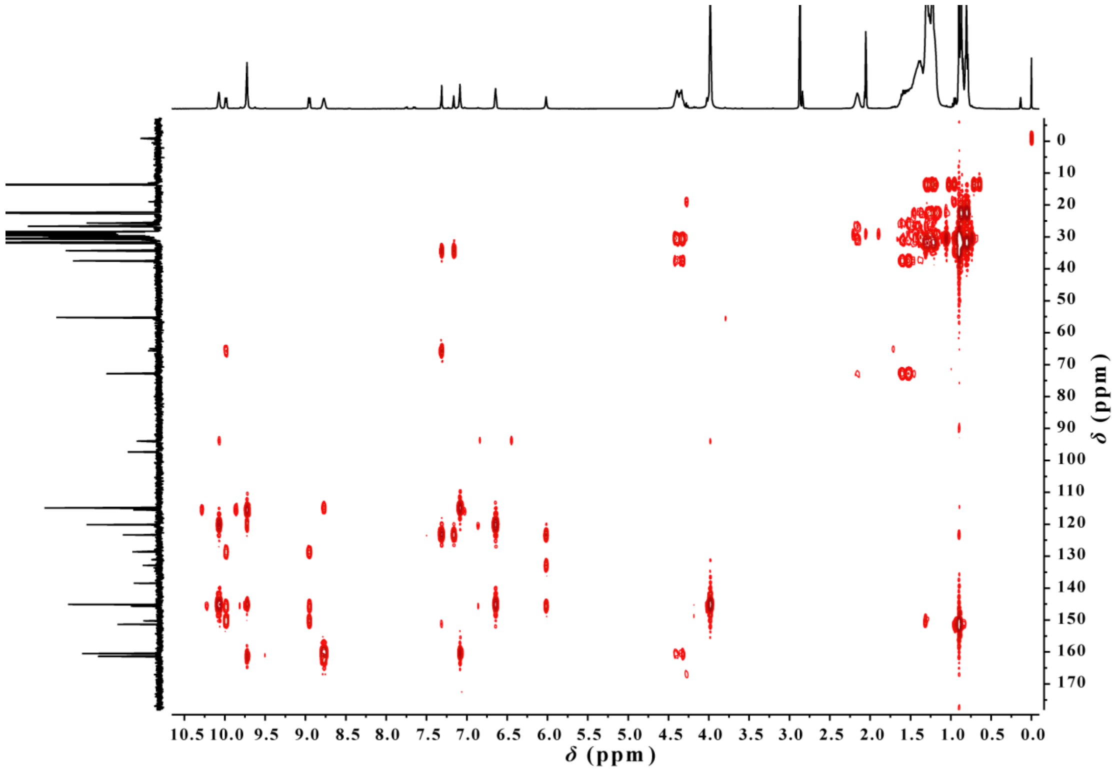

Figure S133 ^{1}H-^{13}C HMBC spectrum of **[3]R-C$_{16}$** (CD_3COCD_3, 400 MHz, 298K).

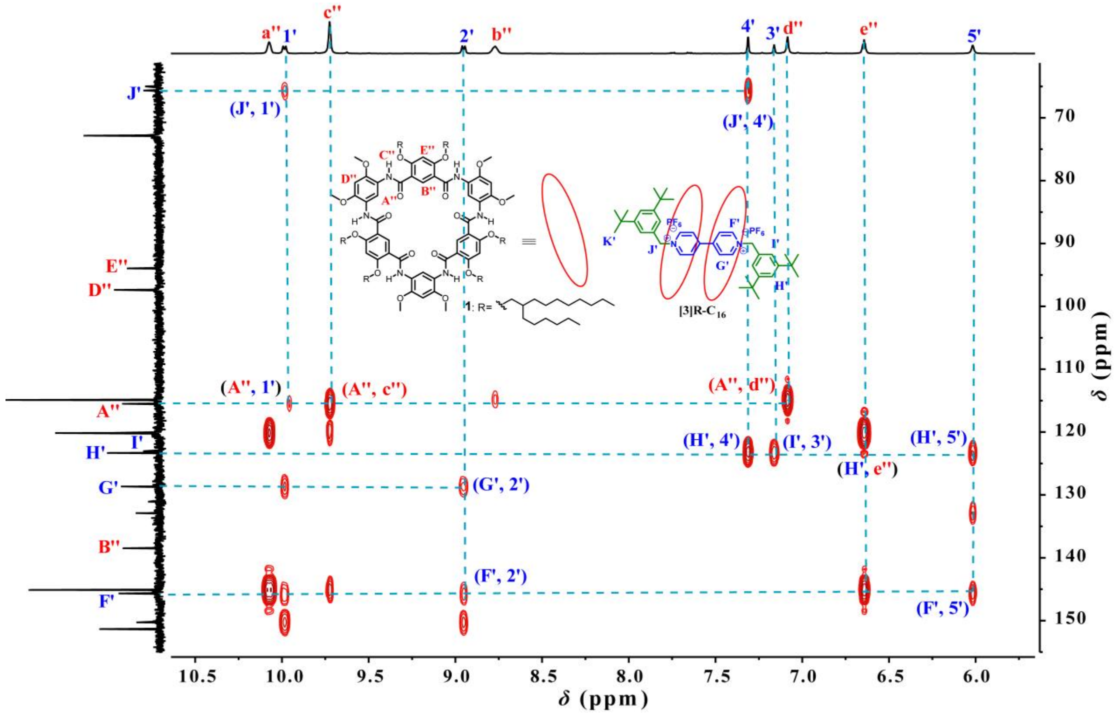

Figure S134 Expanded ^{1}H-^{13}C HMBC spectrum of **[3]R-C$_{16}$** (CD_3COCD_3, 400 MHz, 298K).

6.2 2D DOSY Spectra of Rotaxanes

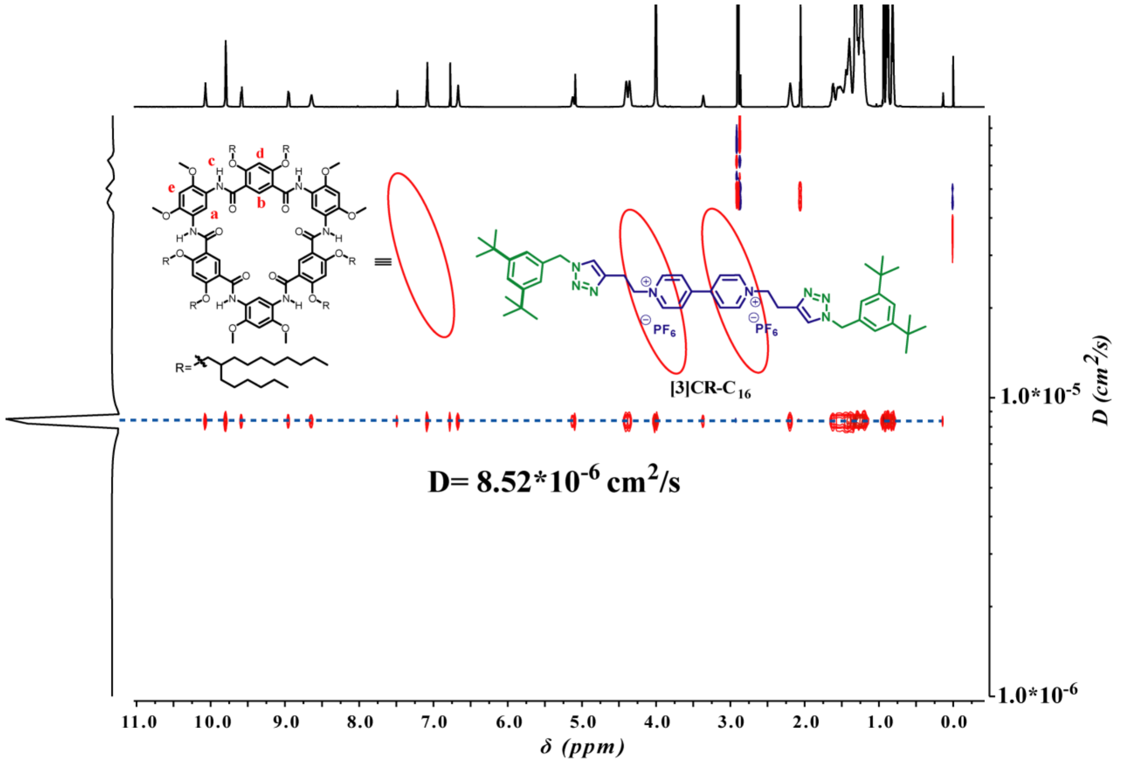

Figure S135 2D-DOSY NMR spectrum of **[3]CR-C$_{16}$** (CD_3COCD_3, 600 MHz, 298K).

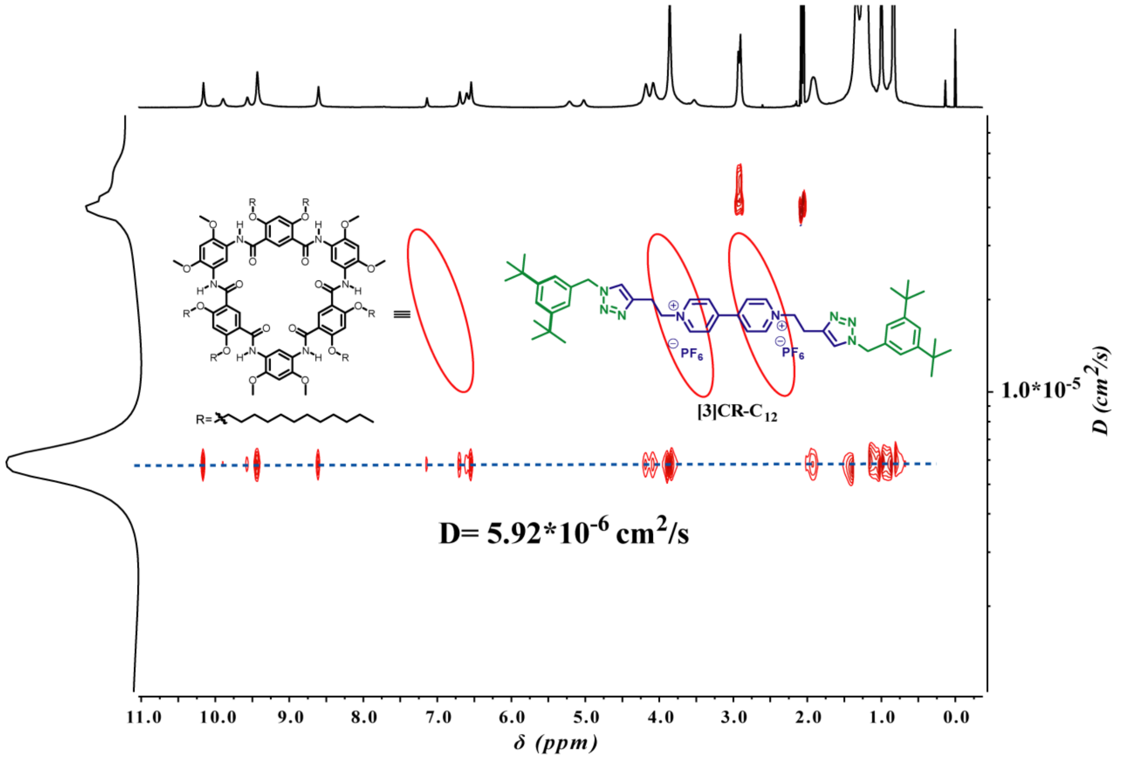

Figure S136 2D-DOSY NMR spectrum of **[3]CR-C$_{12}$** (CD_3COCD_3, 600 MHz, 298K).

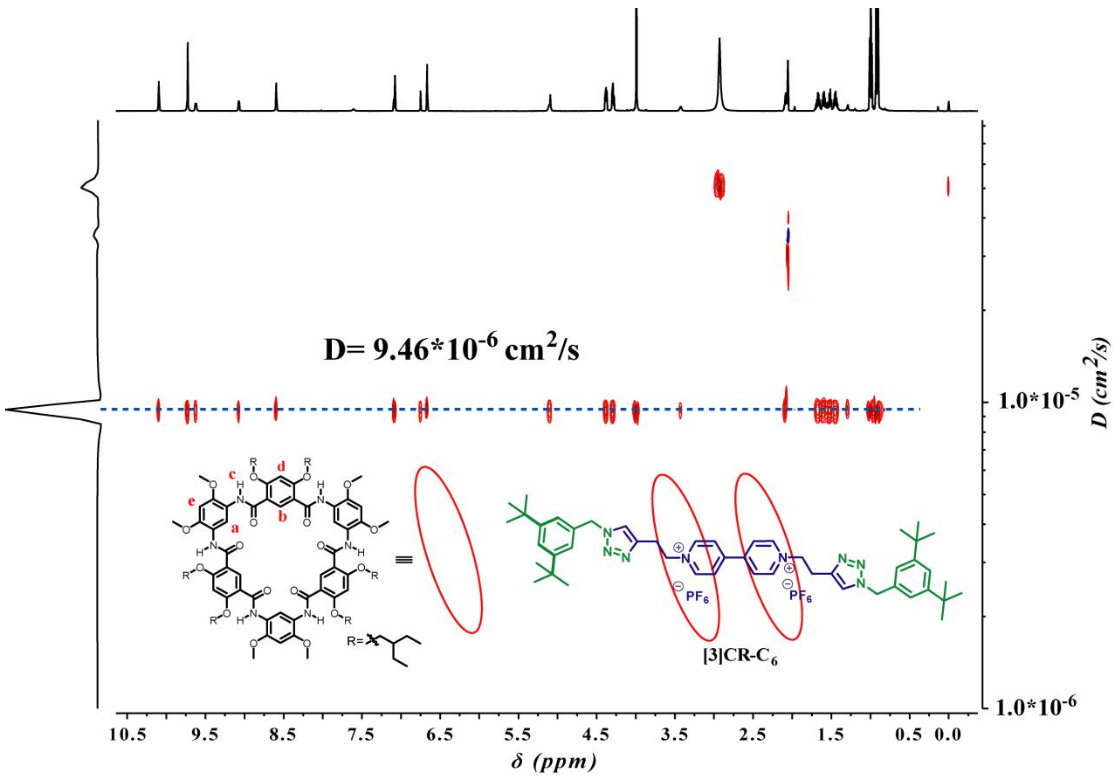

Figure S137 2D-DOSY NMR spectrum of **[3]CR-C$_6$** (CD_3COCD_3, 600 MHz, 298K).

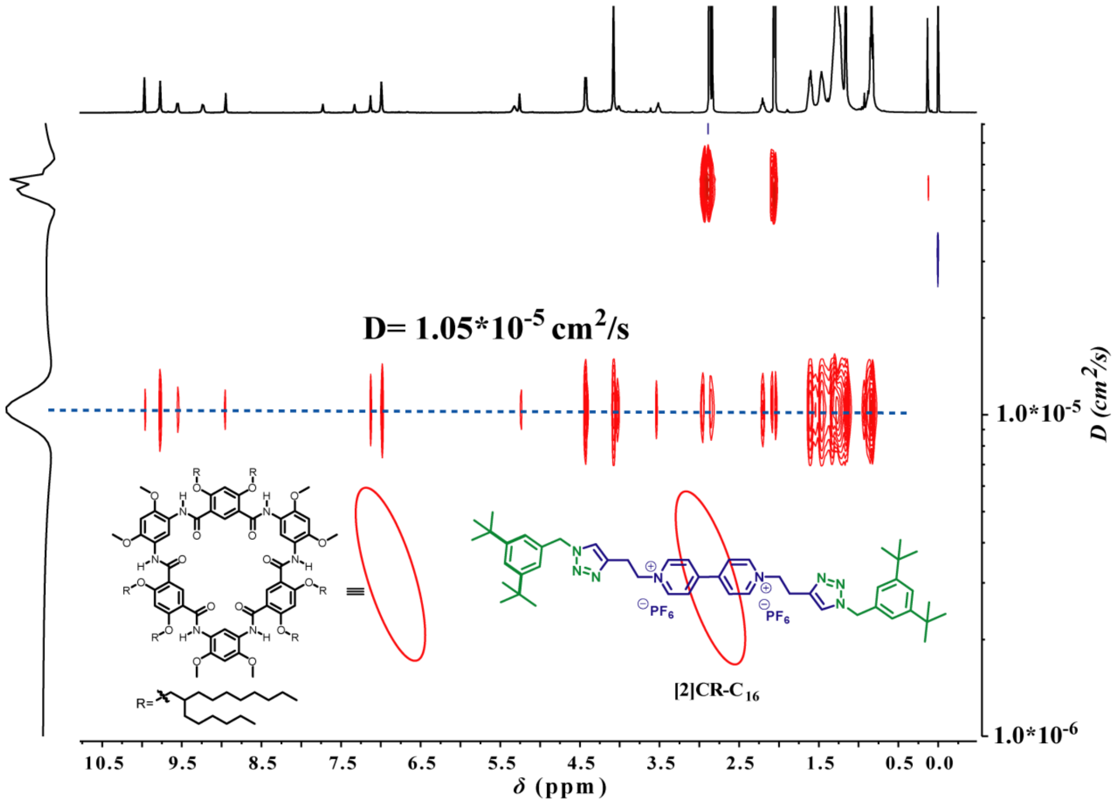

Figure S138 2D-DOSY NMR spectrum of **[2]CR-C$_{16}$** (CD_3COCD_3, 600 MHz, 298K).

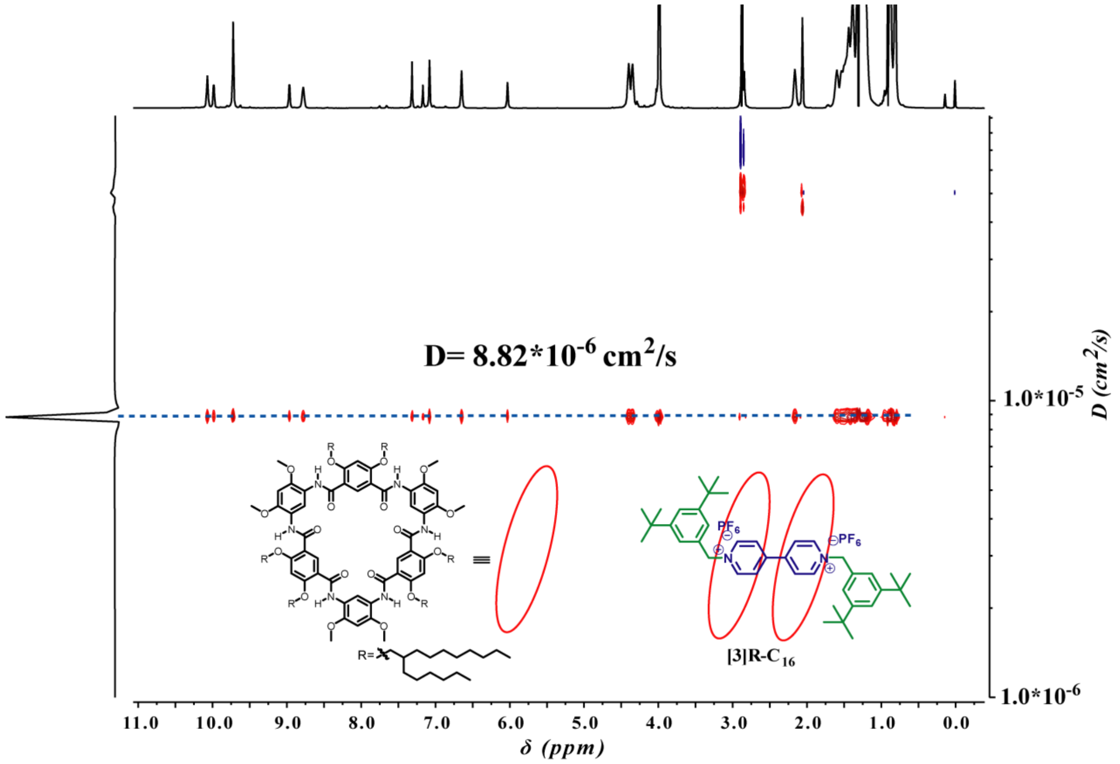

Figure S139 2D-DOSY NMR spectrum of **[3]R-C$_{16}$** (CD_3COCD_3, 600 MHz, 298K).

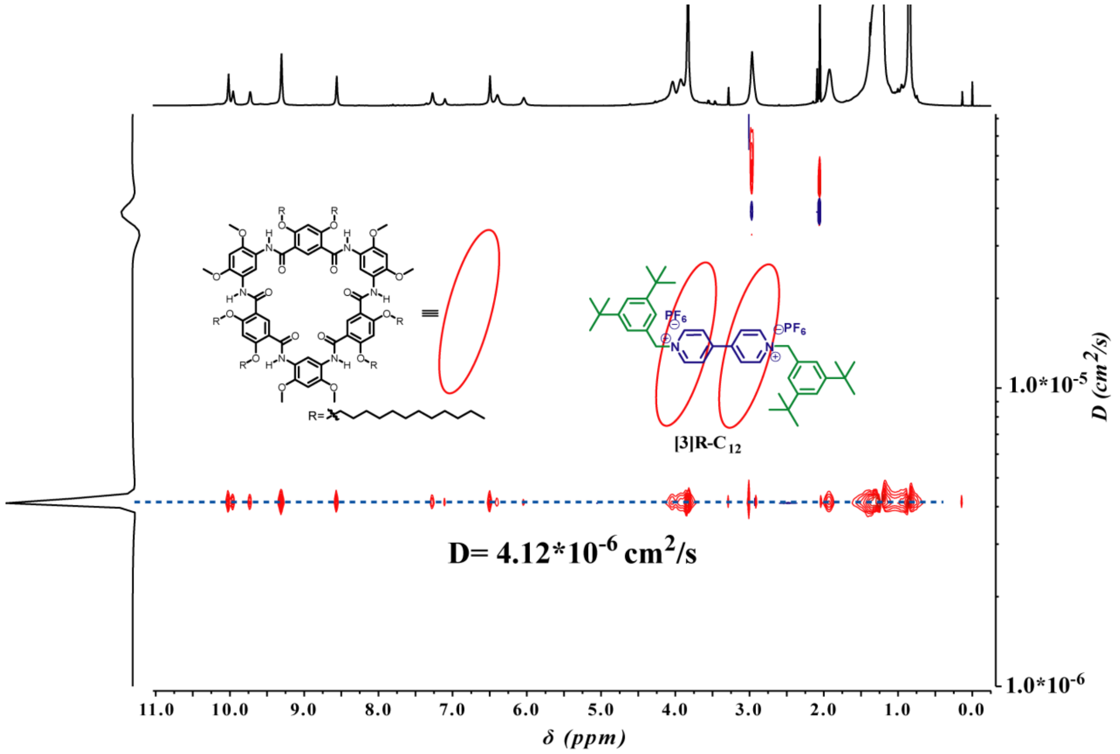

Figure S140 2D-DOSY NMR spectrum of **[3]R-C$_{12}$** (CD_3COCD_3, 600 MHz, 298K).

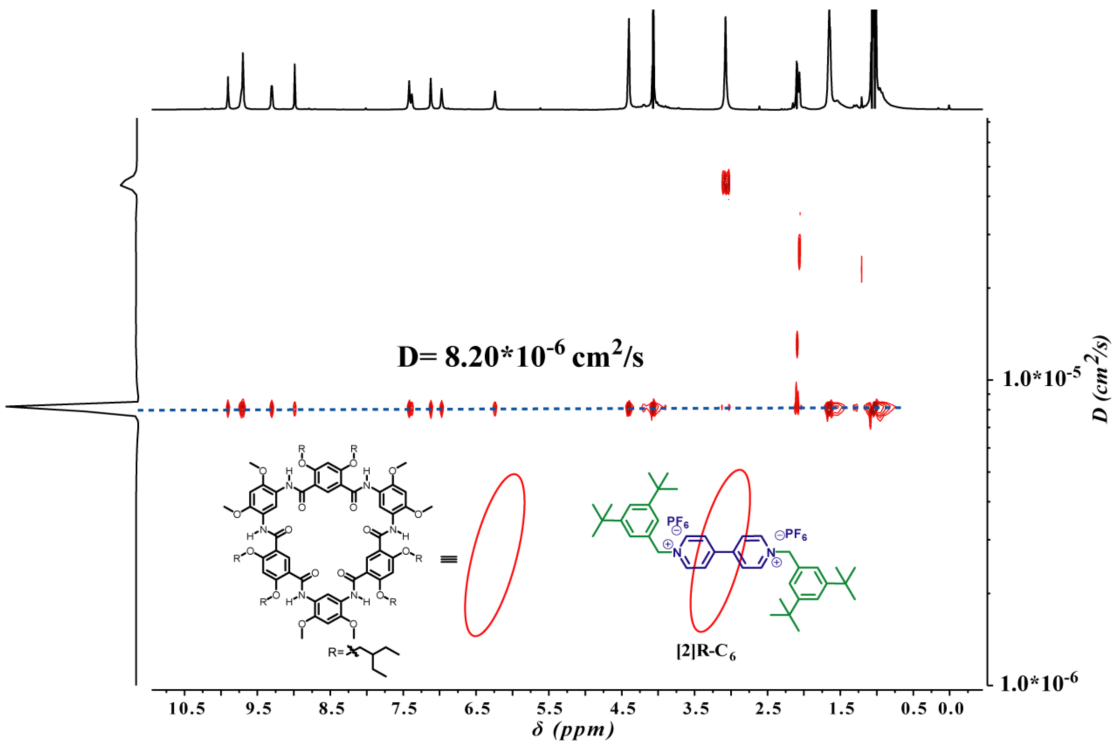

Figure S141 2D-DOSY NMR spectrum of **[2]R-C$_6$** (CD_3COCD_3, 600 MHz, 298K).

7. UV-vis Spectra of Rotaxanes

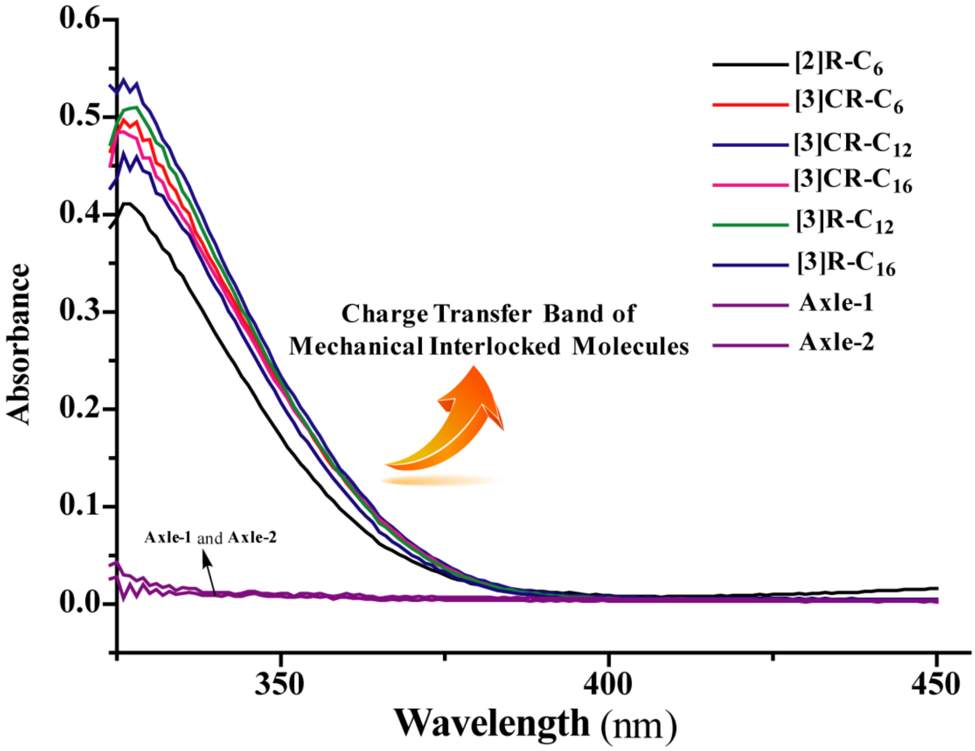

Figure S142 UV-vis spectrum of rotaxanes **[2]R-C$_6$**, **[3]CR-C$_6$**, **[3]R-C$_{12}$**, **[3]CR-C$_{12}$**, **[3]R-C$_{16}$**, **[3]CR-C$_{16}$**, **Axle-1** and **Axle-1** in acetone (concentration of the compound is 5×10^{-5} mol/L).

8. Redox-Responsive of Host-Guest Complexes and Rotaxanes

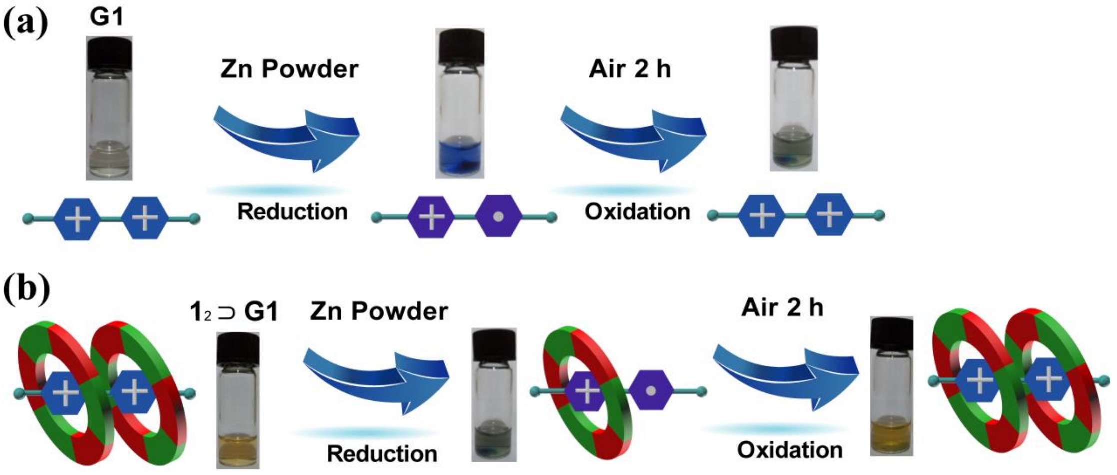

Figure S143 Graphical cartoon representation of redox control of (a) guest **G1** and (b) complex **1**$_2$ ⊃ **G1** and photo showing color changes of redox-responsive complexation, solvent is argon-purged acetone.

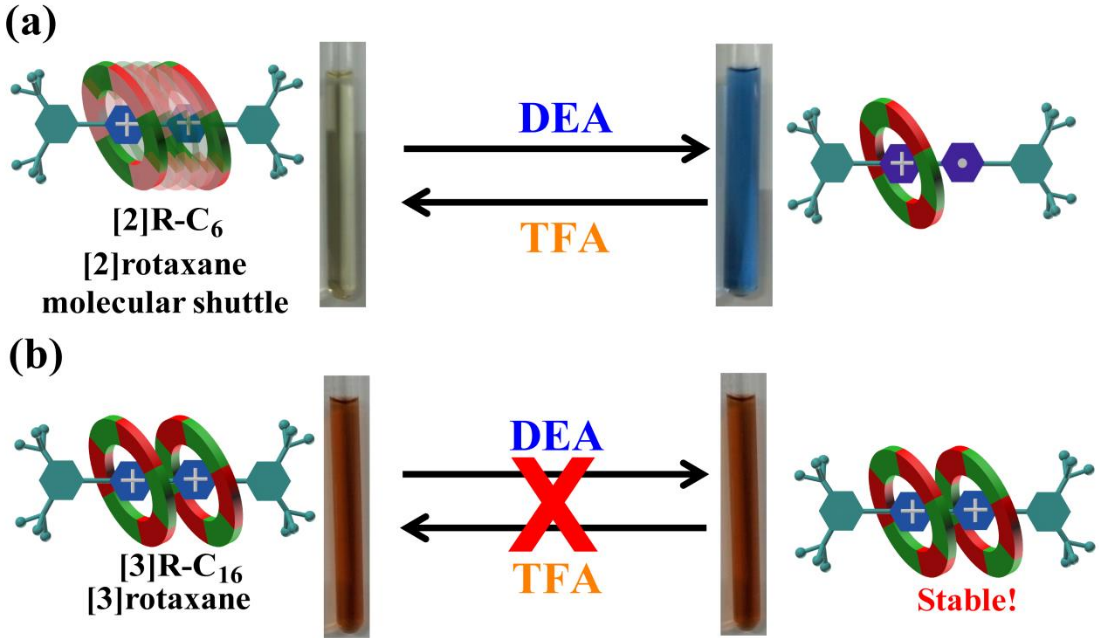

Figure S144 Graphical cartoon representation of redox control of (a) molecular shuttle **[2]R-C$_6$** and (b) [3]rotaxane **[3]R-C$_{16}$** and photo showing color changes of redox-responsive complexation, solvent is argon-purged acetone.

9. X-Ray Single Crystal Structures of $3_2 \supset$ G1 and [3]CR-C$_6$

Crystallographic data (excluding structure factors) for the structures **3_2 ⊃ G1** and **[3]CR-C$_6$** reported in this communication have been deposited with the Cambridge Crystallographic Data Centre as supplementary publication no. CCDC–1475246 and CCDC–1475247. Data collection and structure refinement details can be found in the CIF files or obtained free of charge via www.ccdc.cam.ac.uk/data_request/cif.

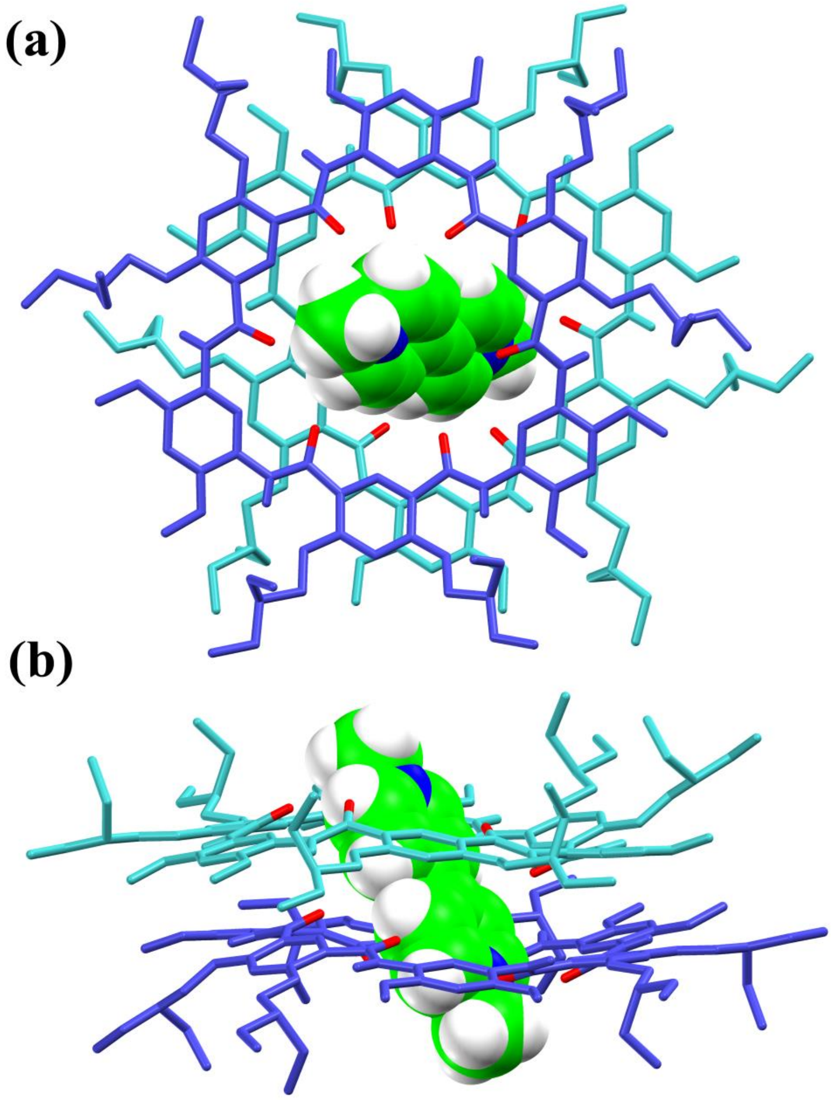

Figure S145 X-ray structure of [3]pseudorotaxane **3_2 ⊃ G1**: (a) top view and (b) side view; cyclo[6]aramide **3** is shown in wire framing representations in cyan and purple, the oxygens in the cavity of macro cycles are shown in red. **G1** is shown in space filling representations. PF_6^- counterions and hydrogens except the ones involved in hydrogen bonding were omitted for clarity.

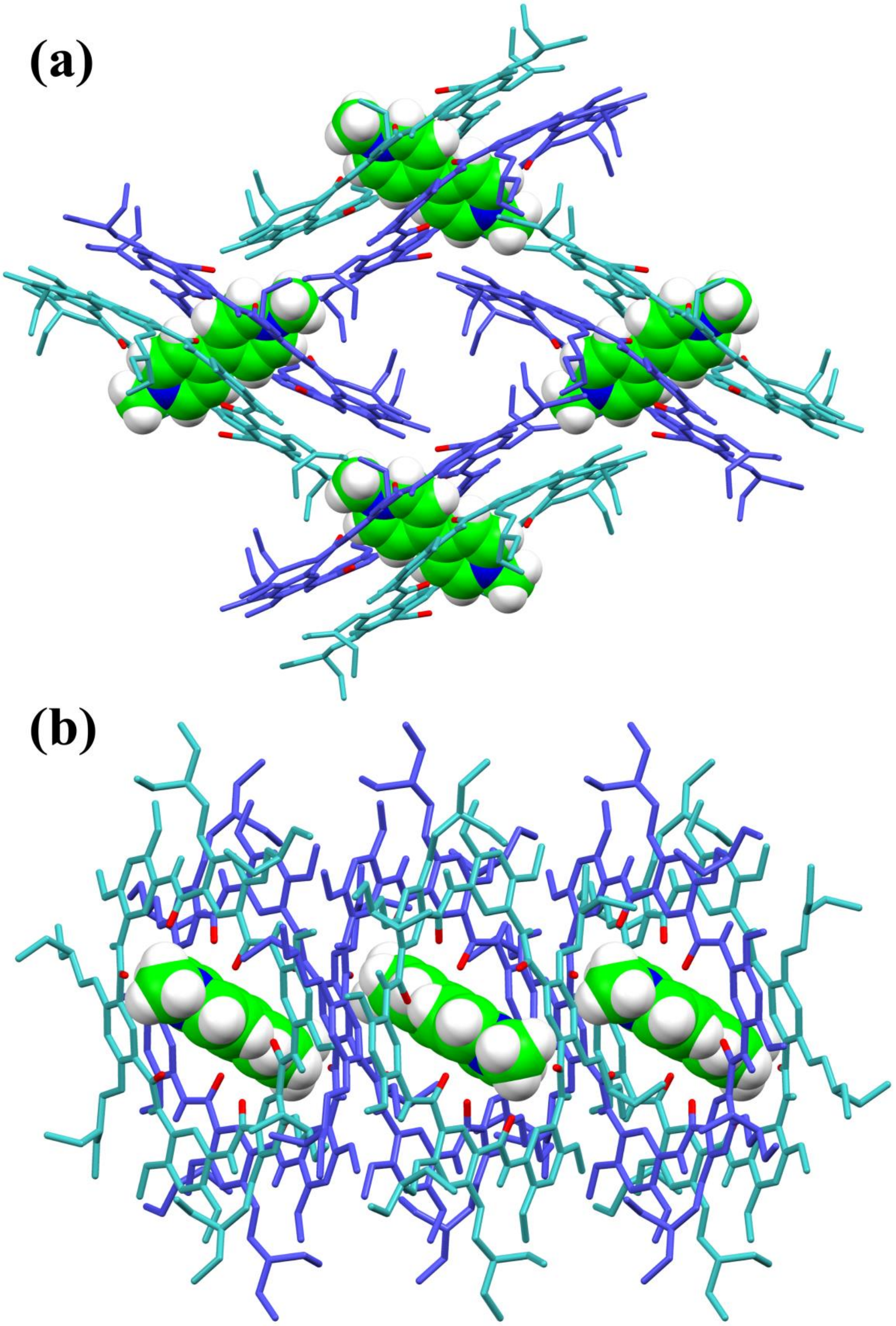

Figure S146 X-ray structure packing of [3]pseudorotaxane $\mathbf{3}_2 \supset$ **G1**, cyclo[6]aramide **3** is shown in wire framing representations in cyan and purple, the oxygens in the cavity of macro cycles are shown in red. **G1** is shown in space filling representations.

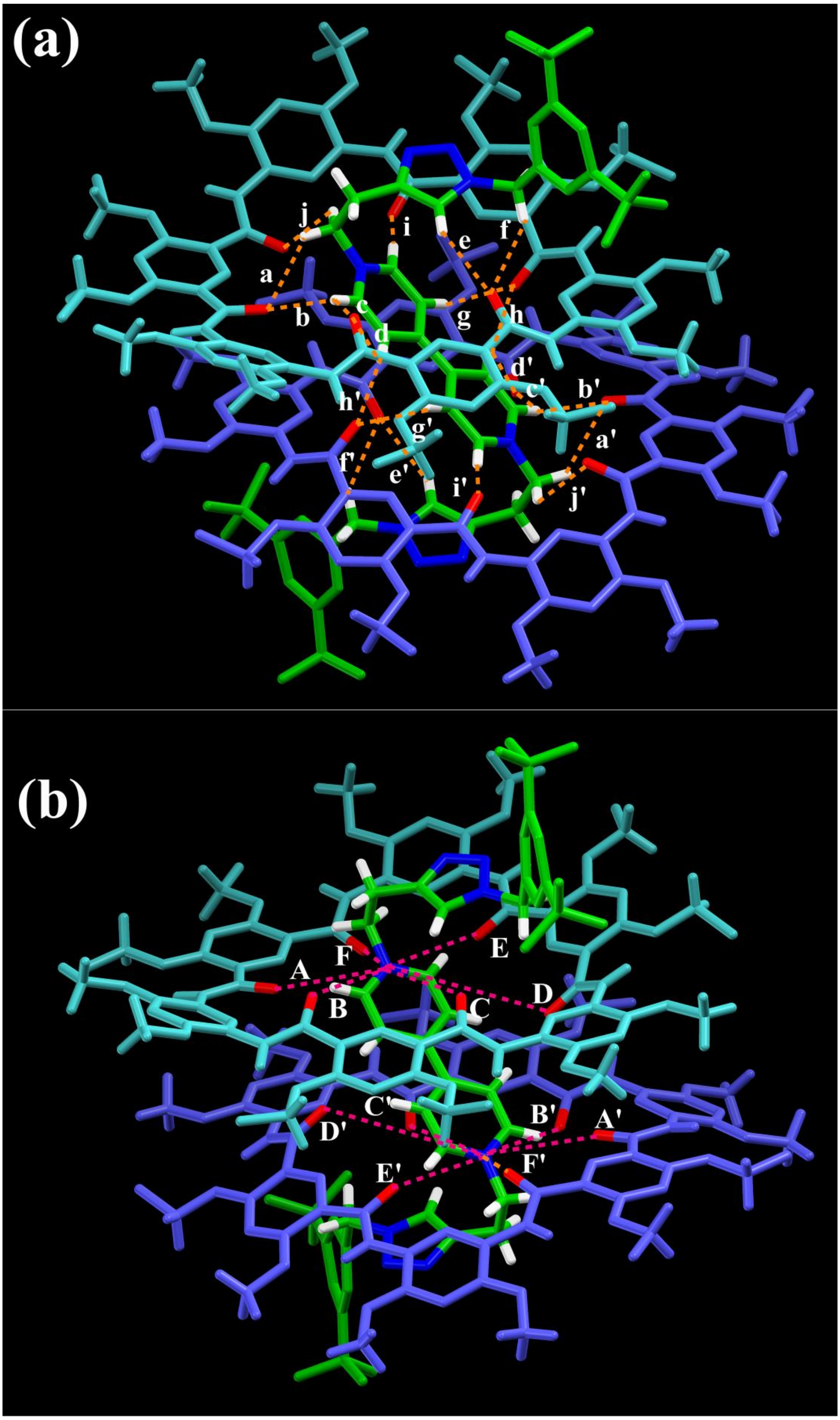

Figure S147 X-ray crystal structure of rotaxane **[3]CR-C$_6$** (a) side and (b) top views. PF_6^- counterions and hydrogens except the ones involved in hydrogen bonding were omitted for clarity.

Table S4 C-H---O hydrogen bonds in the crystal structure of rotaxane **[3]CR-C$_6$**

No. of C-H ··O hydrogen bonds	H ··O / Å C−H···O angles	No. of C-H ··O hydrogen bonds	H ··O / Å (C−H···O angles)
a	2.721 (136.02 °)	a’	2.721 (136.02 °)
b	2.174 (132.31 °)	b’	2.174 (132.31 °)
c	2.429 (117.87 °)	c’	2.429 (117.87 °)
d	2.789 (104.60 °)	d’	2.789 (104.60 °)
e	2.457 (141.76 °)	e’	2.457 (141.76 °)
f	2.854 (134.66 °)	f’	2.854 (134.66 °)
g	2.310 (144.34 °)	g’	2.310 (144.34 °)
h	2.233 (152.01 °)	h’	2.233 (152.01 °)
i	2.173 (160.49 °)	i’	2.173 (160.49 °)
j	2.683 (115.24 °)	j’	2.683 (160.49 °)

Table S5 N^+ ··O interaction in the crystal structure of rotaxane **[3]CR-C$_6$**

No. of N^+ ··O interaction	N^+ ··O / Å	No. of N^+ ··O interaction	N^+ ··O / Å
A	3.515	A’	3.515
B	4.125	B’	4.125
C	4.576	C’	4.576
D	5.181	D’	5.181
E	4.014	E’	4.014
F	3.563	F’	3.563

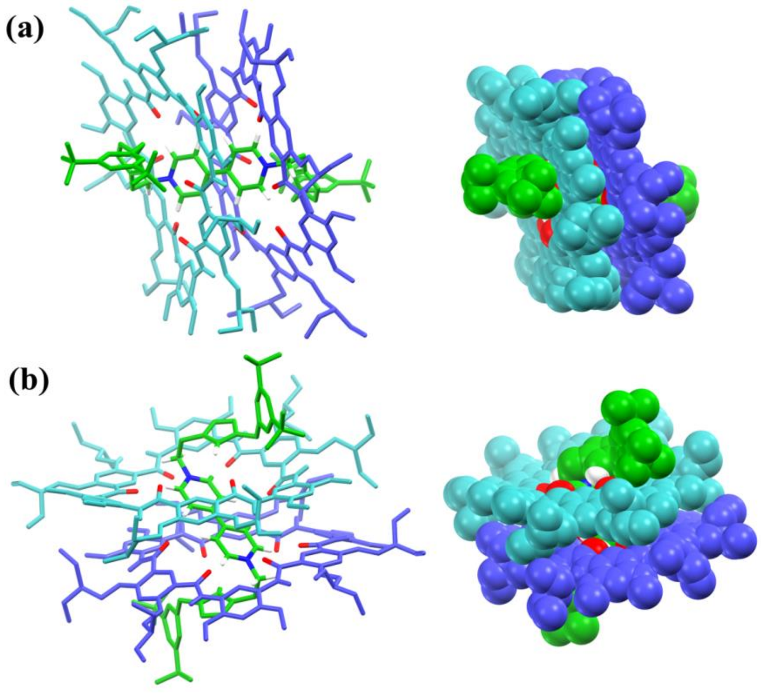

Figure S148 X-ray Crystal structure of rotaxane **[3]CR-C$_6$** (a) side and (b) top views. Insets are the space filling models. PF_6^- counterions and hydrogens except the ones involved in hydrogen bonding were omitted for clarity.

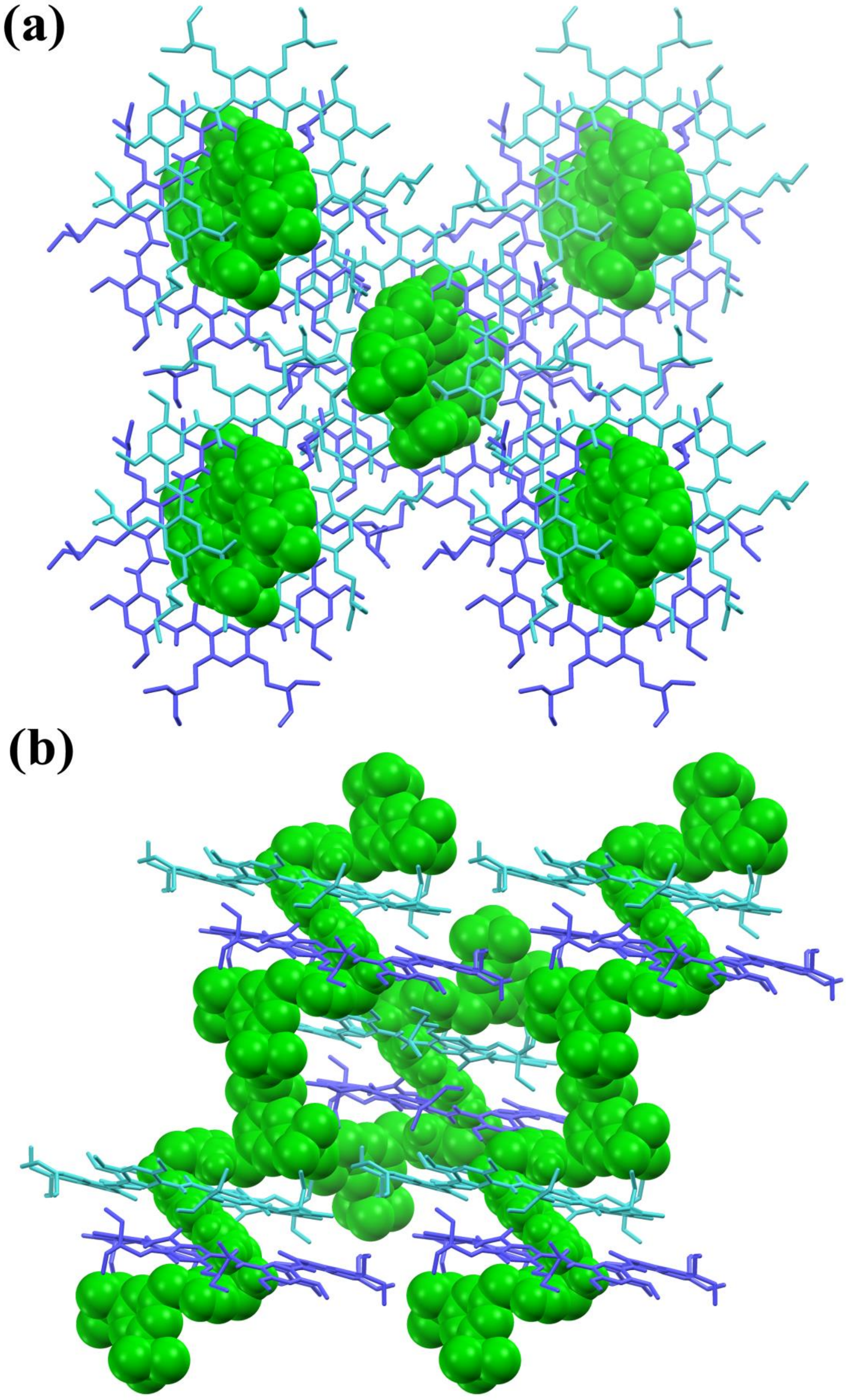

Figure S149 X-ray structure packing of [3] rotaxane **[3]CR-C$_6$**, (a) from the a axle and (b) from the b axle. Cyclo[6]aramide **3** is shown in wire framing representations in cyan and purple and **Axle-1** is shown in space filling representations in green. PF_6^- counterions and hydrogens except the ones involved in hydrogen bonding were omitted for clarity.

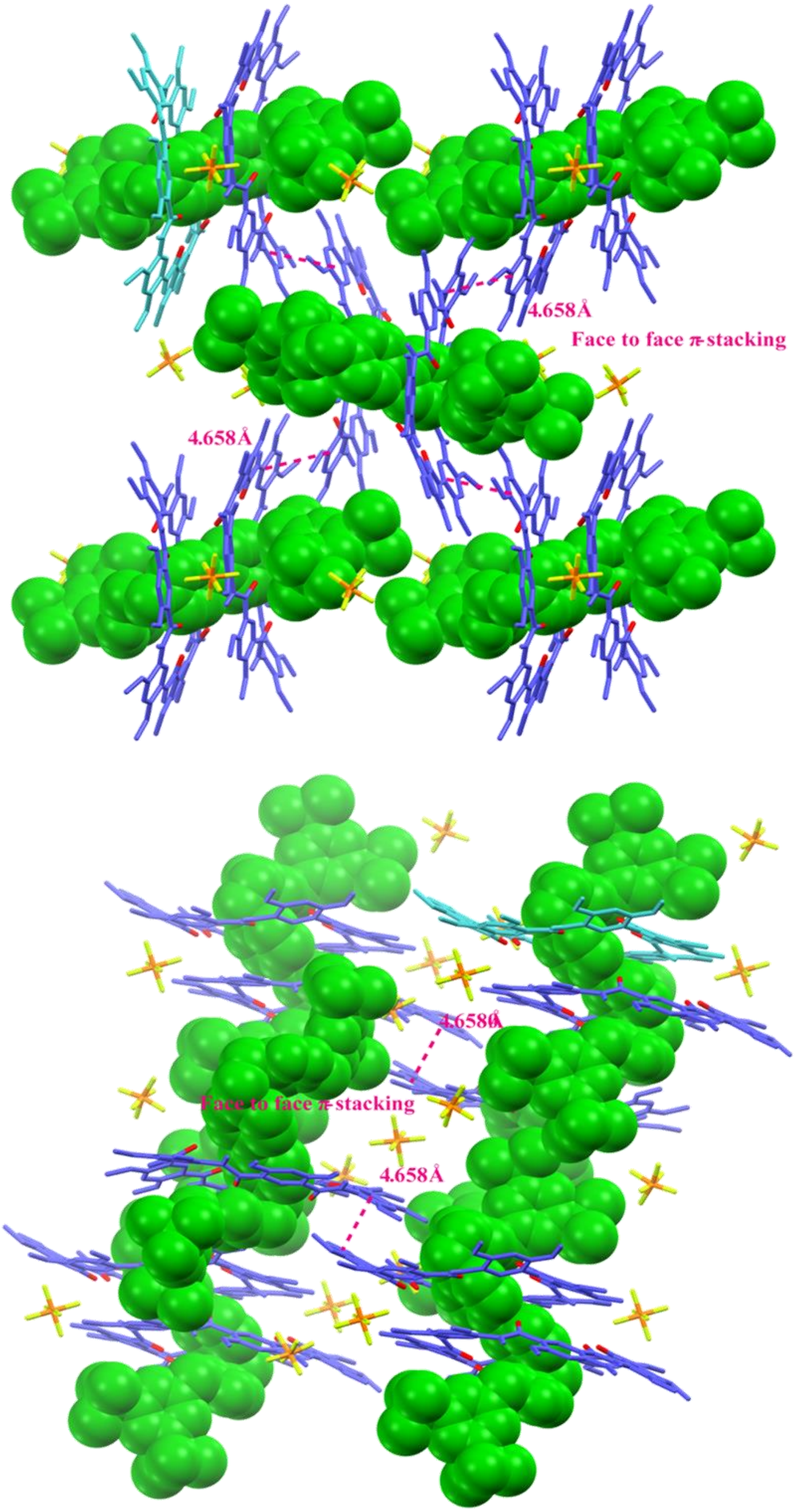

Figure S150 X-ray structure packing of [3] rotaxane **[3]CR-C$_6$**, (a) from the c axle and (b) from the b* axle. Cyclo[6]aramide **3** is shown in wire framing representations in cyan and purple and

Axle-1 is shown in space filling representations in green and oxygens in cavities are showing in red, hydrogens were omitted for clarity. The dashed white lines indicate the weak π-stacking parameters: centroid-centroid distance (Å), 4.658; ring plane-ring plane inclination (°), 2.96.

Table S6 Crystallographic data for [3]pseudorotaxane **3**$_2$ ⊃ **G1** and [3] rotaxane **[3]CR-C$_6$**

Identification code	**3**$_2$ ⊃ **G1**	**[3]CR-C$_6$**
CCDC	1475246	1475247
Empirical formula	$C_{90}H_{121}N_7O_{18}PF_6$	$C_{216}H_{292}N_{20}O_{36}P_2F_{12}$
Formula weight	1733.91	4034.64
Temperature/K	123 (2) K	150 (2) K
Wavelength	0.71073 Å	0.71073 Å
Crystal system	Monoclinic	Monoclinic
Space group	$P2_1/c$	$P2_1/n$
a/Å	22.630 (8)	22.928 (2)
b/Å	23.529 (9)	25.6042 (12)
c/Å	19.425 (7)	23.2131 (15)
α/°	90	90
β/°	112.979 (5)	111.030 (9)
γ/°	90	90
Volume/Å^3	9522 (6)	12719.6 (17)
Z	4	2
ρ_{calc} mg/mm^3	1.209	1.053
μ(Mo Kα) /mm^{-1}	0.107	0.089
F(000)	3692	4308
Crystal size/mm^3	0.31 × 0.18 × 0.18	0.28 × 0.21 × 0.18
2Θ range for data collection	0.98 to 25.60 °	2.89 to 25.51 °
Index ranges	$-27 \le h \le 27$, $-19 \le k \le 28$, $-23 \le l \le 23$	$-27 \le h \le 23$, $-30 \le k \le 31$, $-28 \le l \le 26$
Reflections collected	54751	52517
Independent reflections	17399 [R(int) = 0.0938]	23345 [R(int) = 0.0624]
Completeness to theta	98.4 % (25.51 °)	97.0 % (25.60 °)
Max. and min. transmission	0.9810 and 0.9675	0.9841 and 0.9755
Data/restraints/parameters	17399 / 288 / 1170	23345 / 465 / 1400
Goodness-of-fit on F^2	1.262	1.051
Final R indices [I > 2sigma (I)]	R^1 = 0.0896, wR^2 = 0.2339	R^1 = 0.1198, wR^2 = 0.3108
R indices (all data)	R^1 = 0.1534, wR^2 = 0.2667	R^1 = 0.1758, wR^2 = 0.3386
Largest diff. peak/hole / e Å^{-3}	0.728 / -0.558	1.055 / -0.622

10. Molecular Modeling

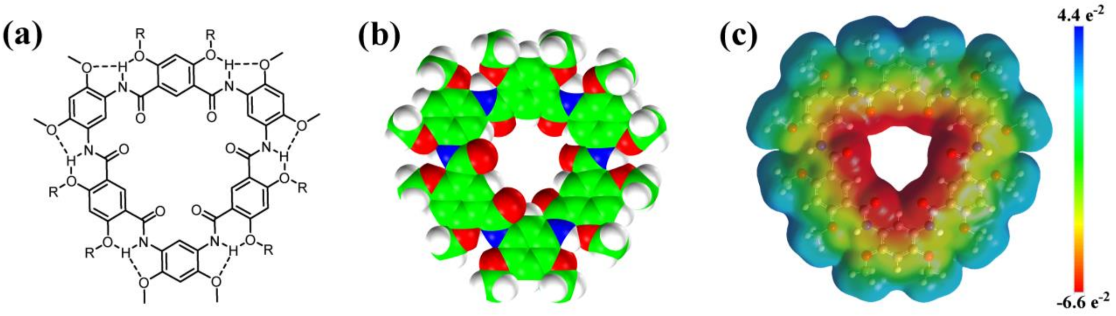

Figure S151 (a) Chemical structure of cyclo[6]aramide and (b) X-ray crystal structure of cyclo[6]aramide core and (c) Geometry optimized by B3PW91/6-31G (d, p) Electrostatic Potential Map of Cyclo[6]aramide **4** (red = −173 kJ mol^{-1}; blue = +115 kJ mol^{-1})

The structure of compact [3]rotaxane **[3]R-C$_1$** based on cyclo[6]aramide **4** were optimized by the density functional theory (DFT) method at the B3PW91/6-31G (d, p) level by employing the Gaussian09 program.[1] Corresponding atomic coordinates were listed in **Table S7** and optimized geometry structures of **[3]R-C$_1$** were displayed in **Figure S152**.

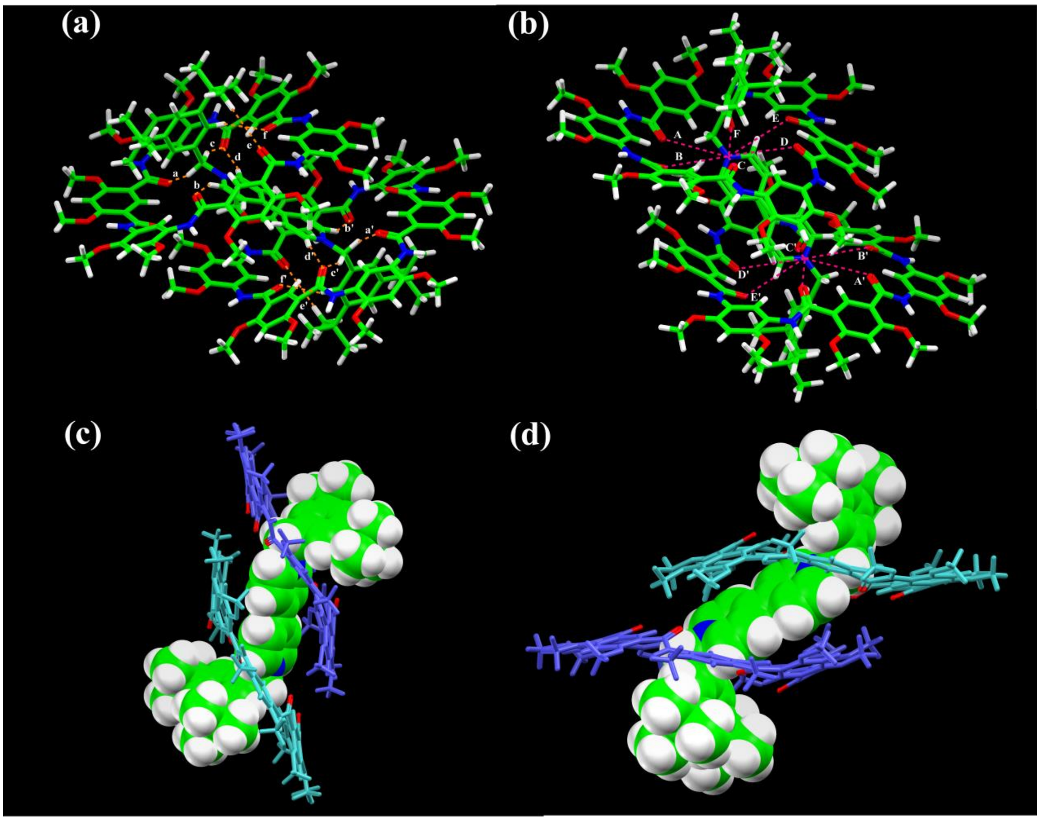

Figure S152 Two Side view (a) and (b) of optimized geometry of **[3]R-C$_1$** at the B3PW91/6-31G (d, p) level (green = carbon, white = hydrogen, red = oxygen and blue = nitrogen) and two side view (c) and (d) of optimized geometry of **[3]R-C$_1$** at the B3PW91/6-31G (d, p) level (green = C, white = H, blue = N for axle molecule in space filling models, red = O in the cavity of cyclo[6]aramides, cyclo[6]aramides are shown in cyan or light blue). All side chains are replaced by methyl groups for simplicity and the PF_6^- counterions are omitted. The dashed orange lines indicate intermolecular H-bonds a-f and a'-f' (a = 2.677 Å (120.42 °), b = 2.280 Å (144.02 °), c = 2.515 Å (155.49 °), d = 2.309 Å (162.90 °), e = 2.872 Å (169.83 °), f = 2.540 Å (156.44 °), a' = 2.677 Å (117.58 °), b' = 2.280 Å (144.02 °), c' = 2.515 Å (155.49 °), d' = 2.309 Å (162.90 °), e' = 2.872 Å (169.83 °), f' = 2.540 Å (156.44 °)) the dashed pink lines indicate the $N^+\cdots O$ interaction A-F and A'-F' (A = 4.365 Å, B = 4.808 Å, C = 4.373 Å, D = 4.574 Å, E = 3.675 Å, F = 3.924 Å, A = 4.365 Å, B = 4.808 Å, C = 4.373 Å, D = 4.574 Å, E = 3.675 Å, F = 3.924 Å).

Table S7 Atomic coordinates for the optimized structure of the rotaxane **[3]R-C$_1$**

Center Number	Atomic Number	Atomic Type	Coordinates (Angstroms)		
			X	Y	Z
1	6	0	2.84135	-0.66588	-1.01377
2	1	0	3.45211	-1.408	-1.51551
3	6	0	1.5256	-0.89311	-0.72944
4	1	0	1.13164	-1.861	-1.00849
5	6	0	0.69927	0.09487	-0.11314
6	6	0	1.39421	1.28648	0.25863
7	1	0	0.88929	2.09315	0.77559
8	6	0	2.71831	1.46192	-0.02583
9	1	0	3.26432	2.35487	0.25882
10	6	0	4.87711	0.74098	-0.98038
11	1	0	5.05649	1.8047	-0.80791
12	1	0	5.4656	0.19992	-0.23339
13	7	0	3.44324	0.51523	-0.69064
14	6	0	-2.84136	0.66588	1.01384
15	1	0	-3.45211	1.40799	1.51557
16	6	0	-1.52561	0.89311	0.7295
17	1	0	-1.13165	1.861	1.00854
18	6	0	-0.69927	-0.09487	0.1132
19	6	0	-1.39422	-1.28649	-0.25856
20	1	0	-0.88931	-2.09315	-0.77553
21	6	0	-2.71832	-1.46192	0.02591
22	1	0	-3.26433	-2.35488	-0.25875
23	6	0	-4.87712	-0.74099	0.98046
24	1	0	-5.0565	-1.80472	0.80801
25	1	0	-5.46561	-0.19995	0.23346
26	7	0	-3.44325	-0.51524	0.69071

27	6	0	8.23025	0.92981	1.77006
28	6	0	5.15675	5.10111	-0.00354
29	6	0	8.87867	3.31427	1.53755
30	6	0	9.95925	4.19898	1.71753
31	6	0	9.80908	5.5579	1.44119
32	1	0	10.64273	6.23228	1.58114
33	6	0	8.58971	6.04678	0.97399
34	6	0	7.50251	5.17353	0.78935
35	6	0	7.65661	3.81732	1.0876
36	1	0	6.82215	3.14632	0.96929
37	6	0	12.24911	4.46521	2.33469
38	1	0	12.55375	4.94268	1.3952
39	1	0	13.05352	3.81694	2.68363
40	1	0	12.05868	5.23574	3.0917
41	6	0	9.43663	8.26628	0.73819
42	1	0	9.79876	8.3613	1.7692
43	1	0	9.04637	9.22995	0.40905
44	1	0	10.267	7.9722	0.08469
45	6	0	4.15925	5.77244	-0.90425
46	6	0	3.26114	4.90262	-1.51033
47	1	0	3.37018	3.84468	-1.31945
48	6	0	2.23388	5.29068	-2.36241
49	6	0	2.0886	6.66886	-2.61073
50	6	0	2.99475	7.58026	-2.05266
51	1	0	2.89556	8.63442	-2.27161
52	6	0	4.03171	7.14079	-1.21924
53	6	0	4.89012	9.36794	-0.98072
54	1	0	3.95223	9.81987	-0.63836
55	1	0	5.01216	9.53618	-2.05651
56	6	0	0.91893	8.40822	-3.77562
57	1	0	1.80512	8.76421	-4.31216
58	1	0	0.73452	9.04167	-2.90013
59	6	0	1.44634	4.14543	-2.92295
60	6	0	-0.13015	-0.89694	-4.19552
61	6	0	-0.79146	3.41681	-3.64432
62	6	0	-2.15046	3.84761	-3.62896
63	6	0	-3.18236	2.93262	-3.80678
64	1	0	-4.21092	3.25793	-3.75112
65	6	0	-2.88659	1.5832	-3.98094
66	6	0	-1.53066	1.13931	-4.02739
67	6	0	-0.50173	2.073	-3.87649
68	1	0	0.52077	1.73581	-3.88895
69	6	0	-3.66603	5.64536	-3.23325
70	1	0	-4.23264	5.55266	-4.16685

71	1	0	-3.56886	6.69903	-2.97285
72	1	0	-4.17773	5.10813	-2.42801
73	6	0	-5.18687	0.95419	-3.92835
74	1	0	-5.33075	1.57672	-3.04084
75	1	0	-5.71864	0.01333	-3.79587
76	1	0	-5.55407	1.4775	-4.81871
77	6	0	-0.14966	-2.36101	-3.86598
78	6	0	1.08402	-2.88727	-3.48633
79	1	0	1.94563	-2.23079	-3.52961
80	6	0	1.27433	-4.17772	-2.99402
81	6	0	0.14179	-5.02159	-2.96854
82	6	0	-1.11122	-4.54022	-3.36155
83	1	0	-1.9733	-5.19082	-3.32254
84	6	0	-1.2702	-3.21635	-3.78314
85	6	0	-3.65465	-3.48826	-3.97219
86	1	0	-3.80661	-3.77293	-2.92609
87	1	0	-3.62297	-4.37583	-4.61335
88	6	0	-0.78784	-7.18343	-2.49595
89	1	0	-1.22089	-7.32941	-3.49186
90	1	0	-1.5573	-6.82246	-1.8045
91	6	0	2.64711	-4.44708	-2.43583
92	6	0	6.89373	-3.67963	1.02875
93	6	0	4.12447	-6.12432	-1.38574
94	6	0	4.42902	-7.49778	-1.43975
95	6	0	5.60541	-7.98235	-0.86782
96	1	0	5.84016	-9.03636	-0.92749
97	6	0	6.48543	-7.10735	-0.22775
98	6	0	6.17783	-5.73871	-0.1387
99	6	0	5.00022	-5.26389	-0.72105
100	1	0	4.77007	-4.2119	-0.67196
101	6	0	3.79447	-9.65281	-2.2466
102	1	0	4.71789	-9.79795	-2.82013
103	1	0	2.95762	-10.06914	-2.80858
104	1	0	3.8773	-10.1751	-1.28487
105	6	0	8.0751	-8.83407	0.19677
106	1	0	7.37544	-9.51256	0.70068
107	1	0	9.04965	-8.90939	0.68039
108	1	0	8.1708	-9.12619	-0.85626
109	6	0	8.08235	-2.82062	1.36009
110	6	0	7.81083	-1.46156	1.49578
111	1	0	6.78576	-1.13627	1.37313
112	6	0	8.76451	-0.47887	1.75068
113	6	0	10.09759	-0.91453	1.90605
114	6	0	10.41597	-2.27191	1.7898

115	1	0	11.44044	-2.59227	1.91691
116	6	0	9.42771	-3.2189	1.50426
117	6	0	11.04334	-4.99495	1.49515
118	1	0	11.43411	-4.79356	2.49858
119	1	0	11.69361	-4.53641	0.74187
120	6	0	12.39425	-0.35377	2.33443
121	1	0	12.78887	-0.81622	1.42287
122	1	0	12.51527	-1.03634	3.18274
123	7	0	9.1351	1.94828	1.75054
124	1	0	10.11135	1.70083	1.86384
125	7	0	6.33877	5.72853	0.22101
126	1	0	6.4302	6.68187	-0.11006
127	7	0	0.16587	4.38829	-3.3574
128	1	0	-0.16952	5.34305	-3.30991
129	7	0	-1.33875	-0.22784	-4.13322
130	1	0	-2.17622	-0.79912	-4.06824
131	7	0	2.92488	-5.70632	-1.98931
132	1	0	2.24681	-6.43128	-2.18613
133	7	0	7.13383	-4.89254	0.45739
134	1	0	8.08651	-5.23611	0.48178
135	8	0	7.0109	1.11292	1.75257
136	8	0	4.89488	3.97893	0.44603
137	8	0	11.12054	3.63104	2.15144
138	8	0	8.35913	7.35268	0.65013
139	8	0	4.93854	7.98358	-0.66683
140	8	0	1.0583	7.04358	-3.40684
141	8	0	1.93404	3.02097	-2.91498
142	8	0	0.92645	-0.32329	-4.41683
143	8	0	-2.33464	5.16432	-3.40224
144	8	0	-3.80971	0.61332	-4.09126
145	8	0	-2.47292	-2.70586	-4.13309
146	8	0	0.31976	-6.29695	-2.54171
147	8	0	3.46028	-3.52603	-2.36017
148	8	0	5.75337	-3.24481	1.21757
149	8	0	3.50606	-8.27909	-2.0707
150	8	0	7.67308	-7.48356	0.3271
151	8	0	9.7038	-4.53936	1.36652
152	8	0	11.03527	0.02682	2.17312
153	6	0	-8.23021	-0.9298	-1.77007
154	6	0	-5.15671	-5.10109	0.00353
155	6	0	-8.87862	-3.31427	-1.5376
156	6	0	-9.95919	-4.19898	-1.71761
157	6	0	-9.80902	-5.55791	-1.44128
158	1	0	-10.64266	-6.23229	-1.58125

159	6	0	-8.58965	-6.04678	-0.97406
160	6	0	-7.50246	-5.17353	-0.78941
161	6	0	-7.65656	-3.81731	-1.08764
162	1	0	-6.82211	-3.14631	-0.96931
163	6	0	-12.24904	-4.46522	-2.33481
164	1	0	-12.55369	-4.9427	-1.39532
165	1	0	-13.05345	-3.81695	-2.68374
166	1	0	-12.0586	-5.23574	-3.09182
167	6	0	-9.43656	-8.26629	-0.73831
168	1	0	-9.79868	-8.3613	-1.76933
169	1	0	-9.0463	-9.22996	-0.40917
170	1	0	-10.26694	-7.97222	-0.08481
171	6	0	-4.15921	-5.77244	0.90422
172	6	0	-3.26112	-4.90262	1.51034
173	1	0	-3.37018	-3.84468	1.31949
174	6	0	-2.23386	-5.29069	2.36241
175	6	0	-2.08856	-6.66887	2.61069
176	6	0	-2.99469	-7.58027	2.0526
177	1	0	-2.89549	-8.63443	2.27152
178	6	0	-4.03165	-7.14079	1.21918
179	6	0	-4.89004	-9.36795	0.9806
180	1	0	-3.95214	-9.81986	0.63824
181	1	0	-5.01208	-9.53621	2.05639
182	6	0	-0.91887	-8.40824	3.77556
183	1	0	-1.80505	-8.76426	4.31208
184	1	0	-0.73444	-9.04167	2.90005
185	6	0	-1.44635	-4.14544	2.923
186	6	0	0.13013	0.89695	4.19554
187	6	0	0.79145	-3.4168	3.64436
188	6	0	2.15046	-3.84759	3.62899
189	6	0	3.18235	-2.93259	3.80679
190	1	0	4.21092	-3.2579	3.75111
191	6	0	2.88658	-1.58318	3.98095
192	6	0	1.53065	-1.1393	4.02742
193	6	0	0.50171	-2.07299	3.87654
194	1	0	-0.52079	-1.7358	3.88901
195	6	0	3.66603	-5.64533	3.23324
196	1	0	4.23266	-5.55262	4.16682
197	1	0	3.56886	-6.699	2.97284
198	1	0	4.17769	-5.1081	2.42798
199	6	0	5.18685	-0.95416	3.92831
200	1	0	5.33071	-1.57667	3.04079
201	1	0	5.71862	-0.01329	3.79584
202	1	0	5.55407	-1.47748	4.81866

203	6	0	0.14965	2.36102	3.86601
204	6	0	-1.08404	2.88729	3.48636
205	1	0	-1.94565	2.23082	3.52964
206	6	0	-1.27434	4.17774	2.99403
207	6	0	-0.1418	5.0216	2.96856
208	6	0	1.11121	4.54023	3.36157
209	1	0	1.9733	5.19083	3.32255
210	6	0	1.27019	3.21636	3.78316
211	6	0	3.65464	3.48826	3.97221
212	1	0	3.80661	3.77289	2.92611
213	1	0	3.62296	4.37585	4.61334
214	6	0	0.78785	7.18343	2.49594
215	1	0	1.2209	7.32942	3.49185
216	1	0	1.5573	6.82246	1.80449
217	6	0	-2.64711	4.4471	2.43583
218	6	0	-6.8937	3.67964	-1.02874
219	6	0	-4.12447	6.12435	1.38576
220	6	0	-4.42903	7.4978	1.43978
221	6	0	-5.60542	7.98236	0.86784
222	1	0	-5.84018	9.03637	0.92752
223	6	0	-6.48543	7.10736	0.22775
224	6	0	-6.17783	5.73873	0.1387
225	6	0	-5.00021	5.26391	0.72105
226	1	0	-4.77006	4.21192	0.67195
227	6	0	-3.7945	9.65282	2.24664
228	1	0	-4.71792	9.79795	2.82017
229	1	0	-2.95765	10.06916	2.80863
230	1	0	-3.87732	10.17512	1.28492
231	6	0	-8.07512	8.83407	-0.19675
231	1	0	-7.37545	9.51258	-0.70065
233	1	0	-9.04966	8.90939	-0.68037
234	1	0	-8.17082	9.12618	0.85628
235	6	0	-8.08232	2.82062	-1.36011
236	6	0	-7.81079	1.46156	-1.49578
237	1	0	-6.78572	1.13628	-1.37311
238	6	0	-8.76446	0.47888	-1.75071
239	6	0	-10.09754	0.91453	-1.90611
240	6	0	-10.41592	2.27192	-1.78987
241	1	0	-11.4404	2.59227	-1.91701
242	6	0	-9.42768	3.2189	-1.50431
243	6	0	-11.04331	4.99496	-1.49524
244	1	0	-11.43405	4.79357	-2.49868
245	1	0	-11.69359	4.53642	-0.74198
246	6	0	-12.39419	0.35377	-2.33454

247	1	0	-12.78883	0.81622	-1.42298
248	1	0	-12.51519	1.03634	-3.18285
249	7	0	-9.13505	-1.94828	-1.75058
250	1	0	-10.1113	-1.70083	-1.86391
251	7	0	-6.33872	-5.72852	-0.22105
252	1	0	-6.43013	-6.68188	0.10999
253	7	0	-0.16587	-4.38829	3.35744
254	1	0	0.16953	-5.34304	3.30994
255	7	0	1.33873	0.22786	4.13325
256	1	0	2.1762	0.79913	4.06826
257	7	0	-2.92488	5.70634	1.98932
258	1	0	-2.24682	6.4313	2.18615
259	7	0	-7.13382	4.89255	-0.4574
260	1	0	-8.0865	5.23611	-0.48179
261	8	0	-7.01085	-1.11291	-1.75255
262	8	0	-4.89486	-3.97889	-0.44601
263	8	0	-11.12048	-3.63104	-2.15153
264	8	0	-8.35906	-7.35269	-0.65022
265	8	0	-4.93847	-7.98358	0.66674
266	8	0	-1.05826	-7.04359	3.4068
267	8	0	-1.93408	-3.02099	2.91507
268	8	0	-0.92646	0.3233	4.41686
269	8	0	2.33463	-5.1643	3.40226
270	8	0	3.80969	-0.61329	4.09126
271	8	0	2.4729	2.70587	4.13312
272	8	0	-0.31976	6.29696	2.54171
273	8	0	-3.46028	3.52605	2.36018
274	8	0	-5.75334	3.24483	-1.21756
275	8	0	-3.50608	8.27911	2.07074
276	8	0	-7.67309	7.48357	-0.3271
277	8	0	-9.70377	4.53936	-1.36658
278	8	0	-11.03522	-0.02682	-2.1732
279	6	0	5.29346	0.33709	-2.37491
280	6	0	6.15227	-0.74977	-2.54625
281	6	0	4.85331	1.06232	-3.48722
282	6	0	6.58202	-1.13631	-3.82008
283	1	0	6.47797	-1.29777	-1.66795
284	6	0	5.26659	0.71128	-4.77557
285	1	0	4.17055	1.8925	-3.33542
286	6	0	6.12333	-0.38887	-4.9076
287	1	0	6.44239	-0.6781	-5.90488
288	6	0	-5.29348	-0.3371	2.37498
289	6	0	-6.15231	0.74975	2.54631
290	6	0	-4.85335	-1.06232	3.4873

291	6	0	-6.58209	1.13629	3.82013
292	1	0	-6.47802	1.29774	1.668
293	6	0	-5.26666	-0.71129	4.77564
294	1	0	-4.17059	-1.8925	3.33551
295	6	0	-6.12341	0.38885	4.90766
296	1	0	-6.44251	0.67806	5.90493
297	6	0	-4.80654	-1.4612	6.03395
298	6	0	-7.50237	2.34059	4.06143
299	6	0	-6.0284	-1.88629	6.87101
300	1	0	-5.70048	-2.42813	7.76507
301	1	0	-6.62076	-1.03054	7.20808
302	1	0	-6.68848	-2.5457	6.29758
303	6	0	-4.00528	-2.72373	5.69431
304	1	0	-4.59812	-3.42995	5.10225
305	1	0	-3.09444	-2.49313	5.13625
306	1	0	-3.71197	-3.23018	6.62015
307	6	0	-3.90969	-0.52573	6.86921
308	1	0	-3.56922	-1.03681	7.77735
309	1	0	-3.02995	-0.21646	6.29691
310	1	0	-4.44679	0.37705	7.1773
311	6	0	-8.75987	1.89192	4.83038
312	1	0	-8.51348	1.45484	5.80235
313	1	0	-9.4191	2.74834	5.01219
314	1	0	-9.3226	1.14394	4.26178
315	6	0	-6.73828	3.39471	4.88701
316	1	0	-5.83912	3.72114	4.35552
317	1	0	-7.37184	4.27085	5.06843
318	1	0	-6.42984	3.00013	5.85992
319	6	0	-7.95332	2.9964	2.75096
320	1	0	-7.10447	3.39285	2.18595
321	1	0	-8.5012	2.29526	2.11204
322	1	0	-8.62113	3.83589	2.97214
323	6	0	7.50224	-2.34066	-4.0614
324	6	0	4.80646	1.46119	-6.03387
325	6	0	8.75981	-1.89204	-4.83026
326	1	0	9.41901	-2.74849	-5.01205
327	1	0	8.5135	-1.45491	-5.80223
328	1	0	9.32255	-1.14411	-4.2616
329	6	0	6.02832	1.88629	-6.87093
330	1	0	6.62069	1.03055	-7.20799
331	1	0	5.70038	2.42811	-7.76501
332	1	0	6.68838	2.54572	-6.29751
333	6	0	3.90962	0.52572	-6.86913
334	1	0	3.02987	0.21644	-6.29683

335	1	0	3.56914	1.0368	-7.77727
336	1	0	4.44671	-0.37706	-7.17723
337	6	0	4.00519	2.7237	-5.69422
338	1	0	3.09433	2.49309	-5.13619
339	1	0	4.598	3.42992	-5.10213
340	1	0	3.7119	3.23018	-6.62006
341	6	0	6.73812	-3.39468	-4.88708
342	1	0	7.37163	-4.27085	-5.06852
343	1	0	5.83891	-3.72109	-4.35565
344	1	0	6.42974	-3.00002	-5.85997
345	6	0	7.95307	-2.99658	-2.75094
346	1	0	8.50094	-2.29549	-2.11194
347	1	0	7.10417	-3.39302	-2.186
348	1	0	8.62086	-3.83608	-2.97214
349	1	0	0.05589	8.44967	-4.43971
350	1	0	-11.00589	6.07174	-1.33312
351	1	0	-12.93664	-0.56939	-2.53529
352	1	0	-4.47482	-2.84558	-4.28888
353	1	0	-5.72326	-9.82215	0.44522
354	1	0	-0.3909	-8.13187	-2.13382
355	1	0	11.00593	-6.07174	1.33303
356	1	0	12.9367	0.56939	2.53518
357	1	0	4.4748	2.84558	4.28892
358	1	0	0.39091	8.13187	2.13381
359	1	0	5.72335	9.82215	-0.44536
360	1	0	-0.05584	-8.44969	4.43966

The total electronic energy is calculated to be -9208.29742799 a.u.

References

[1] (S1) Gaussian 09, Revision B.01, Frisch, M.J.; Trucks, G.W.; Schlegel, H.B.; Scuseria, G.E.; Robb, M.A.; Cheeseman, J.R.; Scalmani, G.; Barone, V.; Mennucci, B.; Petersson, G.A.; Nakatsuji, H.; Caricato, M.; Li, X.; Hratchian, H. P.; Izmaylov, A. F.; Bloino, J.; Zheng, G.; Sonnenberg,J.L.; Hada, M.; Ehara, M.; Toyota, K.; Fukuda, R.; Hasegawa, J.; Ishida, M.; Nakajima, T.; Honda, Y.; Kitao, O.; Nakai, H.; Vreven, T.; Montgomery, J.A., Jr.; Peralta, J.E.; Ogliaro, F.; Bearpark, M.; Heyd, J. J.; Brothers, E.; Kudin, K. N.; Staroverov, V. N.; Keith, T.; Kobayashi, R.; Normand, J.; Raghavachari, K.; Rendell, A.; Burant, J. C.; Iyengar, S.S.; Tomasi, J.; Cossi, M.; Rega, N.; Millam, J.M.; Klene, M.; Knox, J.E.; Cross, J.B.; Bakken, V.; Adamo, C.; Jaramillo, J.; Gomperts, R.; Stratmann, R.E.; Yazyev, O.; Austin, A.J.; Cammi, R.; Pomelli, C.; Ochterski, J.W.; Martin, R.L.; Morokuma, K.; Zakrzewski, V.G.; Voth, G.A.; Salvador, P.; Dannenberg, J.J.; Dapprich, S.; Daniels, A.D.; Farkas, O.J.; Foresman, B.; Ortiz, J.V.; Cioslowski, J.; Fox, D. J. Gaussian, Inc., Wallingford CT, **2010**.
